# Supplementary material for: Chiral Phosphine Catalyzed Allylic Alkylation of Benzylidene Succinimides with Morita–Baylis–Hillman Carbonates
Source: Molecules. 2023 Mar 21;28(6):2825. doi: 10.3390/molecules28062825 (PMC10053092; doi:10.3390/molecules28062825)
Supplement: Supplementary file 1 [file molecules-28-02825-s001.zip › molecules-2282436-supplementary.pdf]

Supporting Materials

Chiral phosphine catalyzed allylic alkylation of benzylidene succinimides with Morita-Baylis-Hillman carbonates

Chang Liu [1,2], Jianwei Sun [2,]* and Pengfei Li [1,3]*

[1]Shenzhen Grubbs Institute, Department of Chemistry, Guangdong Provincial Key Laboratory of Catalysis, College of Science, Southern University of Science and Technology (SUSTech), Shenzhen 518055, China
[2]Department of Chemistry, The Hong Kong University of Science and Technology, Clear Water Bay, Kowloon, Hong Kong SAR, China
[3]Southern University of Science and Technology Guangming Advanced Research Institute
*Correspondence: sunjw@ust.hk; lipf@sustech.edu.cn

I. General Information……………………………………………………………….S2

II. Substrate Preparation……………………………………………………………..S3

III. Chiral phosphine catalyzed allylic alkylations……………………………...…..S10

IV. Scale-up of the allylic alkylation…...…………………………...……...………S22

V. Determination of the Stereochemistry…………..………………...……...………S23

VI. Copies of NMR of substrates and products……………...………………...………S26

VII. Copies of Chiral HPLC analysis……………………………………………….S81

I. General Information

All chemicals were used without further purification as commercially available unless otherwise noted. Thin-layer chromatography (TLC) was performed on silica gel plates (60F-254) using UV-light (254 and 365 nm). Flash chromatography was conducted on silica gel (300 400 mesh). NMR (400, 500 or 600 MHz for ^{1}H NMR, 100 or 126 MHz for ^{13}C NMR, 376 MHz for ^{19}F NMR) spectra were recorded in $CDCl_3$ with TMS as the internal standard. Chemical shifts are reported in ppm and coupling constants are given in Hz. Data for ^{1}H NMR are recorded as follows: chemical shift (ppm), multiplicity (s, singlet; d, doublet; t, triplet; q, quartet; m, multiplet; dd, doublet-doublet), coupling constant (Hz), integration. Data for ^{13}C NMR are reported in terms of chemical shift (*δ*, ppm). Data for ^{19}F NMR are reported in terms of chemical shift (*δ*, ppm). High resolution mass spectral (HRMS) analyses were recorded on a Thermo Scientific Q Exactive Orbitrap mass spectrometer（Bremen，Germany）with ESI source. The crystal structure and data were recorded on a Rigaku Homelab diffractometer.

II. Substrate Preparation

Synthesis of α-benzylidene succinimides 1.

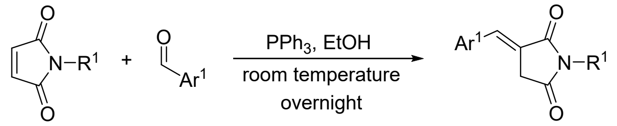

S1 S2 1

The procedure was according to the reported literature.[1] To a solution of pyrrole-2,5-dione **S1** (1.73 g, 10mmol, 1.0 equiv.) and aldehyde **S2** (11 mmol, 1.1 equiv.) in EtOH (100 mL), was added triphenylphosphine (PPh_3, 2.75 g, 10.5 mmol, 1.05 equiv.) at room temperature. The reaction mixture was stirred at room temperature overnight. When the reaction was completed (monitored by TLC), the reaction mixture was filtered, the precipitation was washed with ethanol and dried to afford **1**.

(*E*)-3-benzylidene-1-phenylpyrrolidine-2,5-dione (1a)

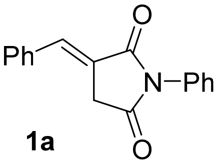

1a

white solid, 95% yield, 2.50g, mp:192.2 – 193.7°C. **^{1}H NMR** (400 MHz, $CDCl_3$): δ 7.75 (t, *J* = 2.4 Hz, 1H), 7.57 – 7.36 (m, 10H), 3.77 (d, *J* = 2.4 Hz, 2H). **^{13}C NMR** (101 MHz, $CDCl_3$): δ 173.2, 170.2, 135.6, 134.2, 132.1, 130.5, 130.4, 129.3, 129.3, 128.7, 126.6, 123.1, 34.4. **HRMS** (CI+) Calcd for $C_{17}H_{14}NO_2^+$ $[M + H]^+$: 264.1025, Found: 264.1016.

(*E*)-3-benzylidene-1-methylpyrrolidine-2,5-dione (1b)

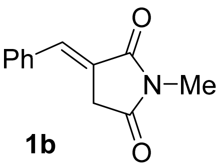

1b

white solid, 87% yield, 1.75g, mp:161.8 – 165.1°C. **^{1}H NMR** (500 MHz, $CDCl_3$): δ 7.62 (t, *J* = 2.4 Hz, 1H), 7.51 – 7.40 (m, 5H), 3.57 (d, *J* = 2.4 Hz, 2H), 3.12 (s, 3H). **^{13}C NMR** (126 MHz, $CDCl_3$): δ 174.3, 171.3, 134.4, 134.2, 130.3, 130.3, 129.3, 123.6, 34.2, 25.1. **HRMS** (CI+) Calcd for $C_{12}H_{12}NO_2^+$ $[M + H]^+$: 202.0868, Found: 202.0862.

(*E*)-1-benzyl-3-benzylidenepyrrolidine-2,5-dione (1c)

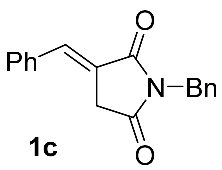

1c

white solid, 93% yield, 2.57g, mp:186.5 – 188.8°C. **^{1}H NMR** (500 MHz, $CDCl_3$): δ 7.62 (t, *J* = 2.4 Hz, 1H), 7.48 – 7.40 (m, 7H), 7.35 – 7.26 (m, 3H), 4.79 (s, 2H), 3.57 (d, *J* = 2.4 Hz, 2H).

[1] L. Yan, W. Yang, L. Li, Y. Sheng, Z. Jiang, *Chin. J. Chem.* **2011**, *29*, 1906-1910.

^{13}C NMR (126 MHz, $CDCl_3$): δ 173.8, 170.8, 136.0, 134.7, 134.2, 130.3, 130.3, 129.2, 129.0, 128.8, 128.1, 123.5, 42.6, 34.2. **HRMS** (CI+) Calcd for $C_{18}H_{16}NO_2^+$ [M + H]$^+$: 278.1181, Found: 278.1174.

(*E*)-3-benzylidene-1-butylpyrrolidine-2,5-dione (1d)

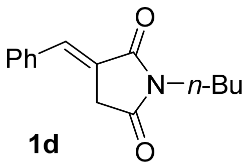

1d

white solid, 96% yield, 2.33g, mp:115.6 – 117.7°C. **^{1}H NMR** (500 MHz, $CDCl_3$): δ 7.60 (s, 1H), 7.49 – 7.41 (m, 5H), 3.63 (t, *J* = 7.4 Hz, 2H), 3.56 (s, 2H), 1.61 (p, *J* = 7.4 Hz, 2H), 1.35 (h, *J* = 7.4 Hz, 2H), 0.94 (t, *J* = 7.4 Hz, 3H). **^{13}C NMR** (126 MHz, $CDCl_3$): δ 174.2, 171.2, 134.3, 134.2, 130.2, 130.2, 129.2, 123.7, 38.9, 34.2, 30.0, 20.3, 13.8. **HRMS** (CI+) Calcd for $C_{15}H_{18}NO_2^+$ [M + H]$^+$: 244.1338, Found: 244.1330.

(*E*)-3-benzylidene-1-(tert-butyl)pyrrolidine-2,5-dione (1e)

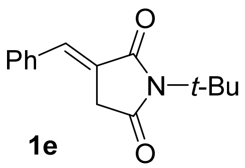

1e

white solid, 96% yield, 2.33g, mp:119.2 – 120.9°C. **^{1}H NMR** (500 MHz, $CDCl_3$): δ 7.51 (t, *J* = 2.5 Hz, 1H), 7.49 – 7.35 (m, 5H), 3.47 (d, *J* = 2.5 Hz, 2H), 1.65 (s, 9H). **^{13}C NMR** (126 MHz, $CDCl_3$): δ 175.2, 172.3, 134.5, 133.0, 130.1, 129.9, 129.1, 124.2, 58.8, 34.9, 28.7. **HRMS** (CI+) Calcd for $C_{15}H_{18}NO_2^+$ [M + H]$^+$: 244.1338, Found: 244.1330.

(*E*)-3-benzylidene-1-(naphthalen-1-ylmethyl)pyrrolidine-2,5-dione (1f)

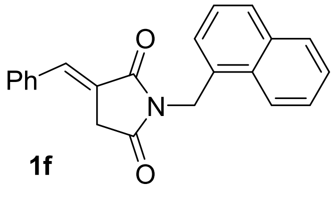

1f

white solid, 92% yield, 2.98g, mp:190.1 – 193.3°C. **^{1}H NMR** (500 MHz, $CDCl_3$): δ 8.37 (d, *J* = 8.5 Hz, 1H), 7.83 (dd, *J* = 26.0, 8.2 Hz, 2H), 7.67 – 7.56 (m, 3H), 7.53 – 7.40 (m, 7H), 5.28 (s, 2H), 3.60 (d, *J* = 2.4 Hz, 2H). **^{13}C NMR** (126 MHz, $CDCl_3$): δ 174.0, 171.0, 134.9, 134.2, 133.9, 131.4, 130.9, 130.4, 130.3, 129.2, 128.9, 128.8, 128.0, 126.7, 126.0, 125.4, 123.8, 123.4, 40.5, 34.3. **HRMS** (CI+) Calcd for $C_{22}H_{18}NO_2^+$ [M + H]$^+$: 328.1338, Found: 328.1328.

(*E*)-1-benzyl-3-(4-methylbenzylidene)pyrrolidine-2,5-dione (1g)

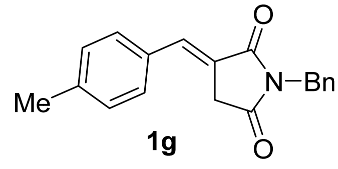

1g

white solid, 86% yield, 2.50g, mp:164.0 – 165.8°C. **^{1}H NMR** (400 MHz, $CDCl_3$): δ 7.62 (t, *J* = 2.4 Hz, 1H), 7.46 (d, *J* = 7.0 Hz, 2H), 7.39 (d, *J* = 7.8 Hz, 2H), 7.37 – 7.24 (m, 5H), 4.81 (s, 2H), 3.57 (d, *J* = 2.4 Hz, 2H), 2.41 (s, 3H). **^{13}C NMR** (101 MHz, $CDCl_3$): δ 173.2, 170.2, 135.6, 134.2, 132.1, 130.5, 130.4, 129.3, 129.3, 128.7, 126.6,

123.1, 34.4. **HRMS** (CI+) Calcd for $C_{19}H_{18}NO_2^+$ $[M + H]^+$: 292.1338, Found: 292.1328.

(*E*)-1-benzyl-3-(4-(methylthio)benzylidene)pyrrolidine-2,5-dione (1h)

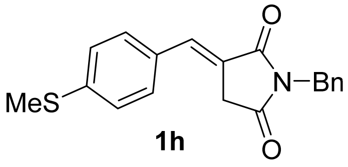

1h

white solid, 92% yield, 2.97g, mp:177.2 – 180.5°C. **^{1}H NMR** (500 MHz, $CDCl_3$): δ 7.55 (t, J = 2.4 Hz, 1H), 7.42 (d, J = 6.7 Hz, 2H), 7.37 (d, J = 8.4 Hz, 2H), 7.32 – 7.24 (m, 5H), 4.77 (s, 2H), 3.53 (d, J = 2.3 Hz, 2H), 2.50 (s, 3H). **^{13}C NMR** (126 MHz, $CDCl_3$): 173.8, 170.9, 142.5, 136.0, 134.2, 130.6, 130.5, 129.0, 128.8, 128.1, 126.0, 122.3, 42.6, 34.3, 15.1. **HRMS** (CI+) Calcd for $C_{19}H_{18}NO_2S^+$ $[M + H]^+$: 324.1058, Found: 324.1049.

(*E*)-1-benzyl-3-(4-(methylthio)benzylidene)pyrrolidine-2,5-dione (1i)

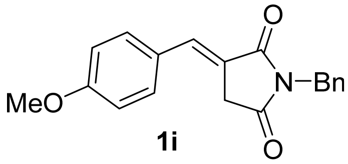

1i

white solid, 90% yield, 2.91g, mp:170.4 – 172.5°C. **^{1}H NMR** (500 MHz, $CDCl_3$): δ 7.57 (t, J = 2.3 Hz, 1H), 7.46 – 7.40 (m, 4H), 7.34 – 7.25 (m, 3H), 6.97 – 6.93 (m, 2H), 4.78 (s, 2H), 3.85 (s, 3H), 3.53 (d, J = 2.3 Hz, 2H). **^{13}C NMR** (126 MHz, $CDCl_3$): δ 174.0, 171.1, 161.3, 136.1, 134.4, 132.1, 129.0, 128.8, 128.0, 126.9, 120.7, 114.7, 55.5, 42.5, 34.3. **HRMS** (CI+) Calcd for $C_{19}H_{18}NO_3^+$ $[M + H]^+$: 308.1287, Found: 308.1277.

(*E*)-1-benzyl-3-(3,5-dimethoxybenzylidene)pyrrolidine-2,5-dione (1j)

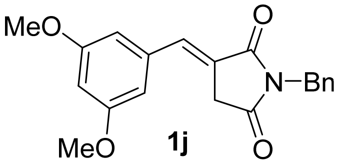

1j

white solid, 95% yield, 3.20g, mp:134.7– 136.2°C. **^{1}H NMR** (500 MHz, $CDCl_3$): δ 7.53 (t, J = 2.4 Hz, 1H), 7.47 – 7.39 (m, 2H), 7.33 – 7.28 (m, 3H), 6.59 (d, J = 2.2 Hz, 2H), 6.51 (t, J = 2.3 Hz, 1H), 4.78 (s, 2H), 3.81 (s, 6H), 3.57 (d, J = 2.4 Hz, 2H). **^{13}C NMR** (126 MHz, $CDCl_3$): δ 173.8, 170.8, 161.2, 135.9, 135.9, 134.8, 129.0, 128.8, 128.1, 124.0, 108.3, 102.3, 55.6, 42.6, 34.2. **HRMS** (CI+) Calcd for $C_{20}H_{20}NO_4^+$ $[M + H]^+$: 338.1392, Found: 338.1381.

(*E*)-1-benzyl-3-(2-methoxybenzylidene)pyrrolidine-2,5-dione (1k)

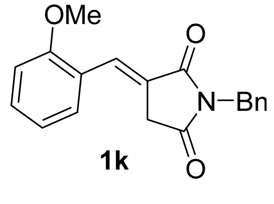

1k

white solid, 90% yield, 2.91g, mp:151.9 – 154.0°C. **^{1}H NMR** (400 MHz, $CDCl_3$): δ 8.04 (t, J = 2.4 Hz, 1H), 7.50 – 7.42 (m,

2H), 7.40 – 7.26 (m, 5H), 7.04 – 6.97 (m, 1H), 6.94 (d, *J* = 8.2 Hz, 1H), 4.79 (s, 2H), 3.88 (s, 3H), 3.51 (d, *J* = 2.4 Hz, 2H). **^{13}C NMR** (101 MHz, $CDCl_3$): δ 174.2, 170.9, 158.6, 136.1, 131.8, 129.8, 129.3, 129.0, 128.7, 128.0, 123.3, 123.2, 120.7, 111.3, 55.7, 42.5, 34.3. **HRMS** (CI+) Calcd for $C_{19}H_{18}NO_3^+$ [M + H]$^+$: 308.1287, Found: 308.1277.

(*E*)-1-benzyl-3-(4-fluorobenzylidene)pyrrolidine-2,5-dione (1l)

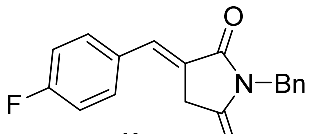

1l

white solid, 87% yield, 2.56g, mp:161.4 – 162.6°C. **^{1}H NMR** (500 MHz, $CDCl_3$): δ 7.59 (t, *J* = 2.4 Hz, 1H), 7.50 – 7.41 (m, 4H), 7.35 – 7.27 (m, 3H), 7.17 – 7.10 (m, 2H), 4.79 (s, 2H), 3.54 (d, *J* = 2.4 Hz, 2H). **^{13}C NMR** (101 MHz, $CDCl_3$): δ 173.6, 170.8, 164.7, 162.7, 135.9, 133.5, 132.3(d, *J* = 8.7), 130.5 (d, *J* = 3.3), 128.9 (d, *J* = 27.8), 128.1, 123.1, 123.1, 116.5 (d, *J* = 22.2), 42.7, 34.1. **^{19}F NMR** (376 MHz, $CDCl_3$): δ -108.6. **HRMS** (CI+) Calcd for $C_{18}H_{15}FNO_2^+$ [M + H]$^+$: 296.1087, Found: 296.1069.

(*E*)-1-benzyl-3-(4-chlorobenzylidene)pyrrolidine-2,5-dione (1m)

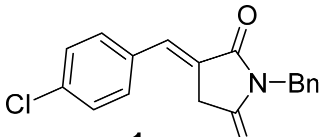

1m

white solid, 96% yield, 2.99g, mp:186.8 – 187.4°C. **^{1}H NMR** (500 MHz, $CDCl_3$): δ 7.56 (t, *J* = 2.4 Hz, 1H), 7.47 – 7.36 (m, 6H), 7.36 – 7.21 (m, 3H), 4.78 (s, 2H), 3.53 (d, *J* = 2.4 Hz, 2H). **^{13}C NMR** (126 MHz, $CDCl_3$): δ 173.5, 170.6, 136.4, 135.8, 133.3, 132.6, 131.4, 129.5, 129.0, 128.8, 128.1, 124.1, 42.7, 34.1. **HRMS** (CI+) Calcd for $C_{18}H_{15}ClNO_2^+$ [M + H]$^+$: 312.0791, Found: 312.0782.

(*E*)-1-benzyl-3-(4-bromobenzylidene)pyrrolidine-2,5-dione (1n)

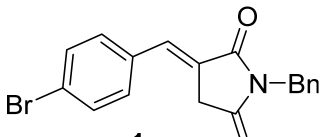

1n

white solid, 95% yield, 3.30g, mp:206.5 – 210.1°C. **^{1}H NMR** (500 MHz, $CDCl_3$): δ 7.61 – 7.53 (m, 3H), 7.46 – 7.40 (m, 2H), 7.37 – 7.27 (m, 5H), 4.79 (s, 2H), 3.53 (d, *J* = 2.4 Hz, 2H). **^{13}C NMR** (126 MHz, $CDCl_3$): δ 173.5, 170.6, 135.8, 133.4, 133.0, 132.5, 131.5, 129.1, 128.8, 128.2, 124.8, 124.2, 42.7, 34.1. **HRMS** (CI+) Calcd for $C_{18}H_{15}BrNO_2^+$ [M + H]$^+$: 356.0286, Found: 356.0275.

(*E*)-1-benzyl-3-(naphthalen-2-ylmethylene)pyrrolidine-2,5-dione (1o)

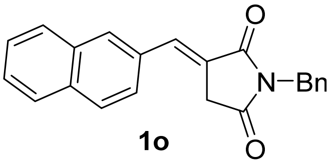

1o

white solid, 96% yield, 3.13g, mp:214.7 – 217.5°C. **^{1}H NMR** (500 MHz, $CDCl_3$): δ 7.96 (d, J = 1.7 Hz, 1H), 7.91 – 7.82 (m, 3H), 7.78 (t, J = 2.5 Hz, 1H), 7.59 – 7.51 (m, 3H), 7.50 – 7.43 (m, 2H), 7.37 – 7.26 (m, 3H), 4.82 (s, 2H), 3.68 (d, J = 2.4 Hz, 2H). **^{13}C NMR** (126 MHz, $CDCl_3$): δ 173.9, 170.9, 136.0, 134.9, 133.9, 133.3, 131.7, 131.6, 129.1, 129.0, 128.8, 128.8, 128.1, 127.9, 127.1, 126.1, 123.5, 42.7, 34.4. **HRMS** (CI+) Calcd for $C_{22}H_{18}NO_2^+$ $[M + H]^+$: 328.1338, Found: 328.1328.

Synthesis of MBH carbonates 2.

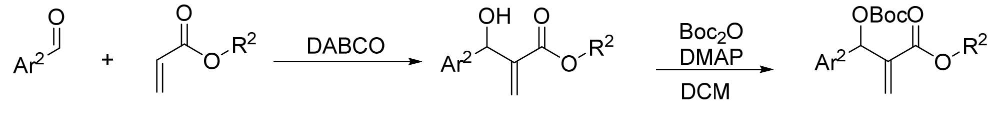

The procedure was according to the reported literature.[2] To a solution of aldehyde **S3** (10 mmol) in acrylate **S4** (20 mL), was added 1,4-diazabicyclo[2.2.2]octane (DABCO, 10.5 mmol) at room temperature. The reaction mixture was stirred at room temperature for 3-7 days. When the reaction was completed (monitored by TLC), the reaction mixture was purified by silica gel column chromatography to afford MBH alcohol **S5**.

To a solution of MBH alcohol **S5** and Boc-anhydride (15 mmol) in CH_2Cl_2 (30 mL) was added 4-(dimethylamino)pyridine (DMAP, 2.08 mmol) in batches. When the reaction was completed (monitored by TLC), the organic phase was washed with distilled water (2 x 20 mL), dried over anhydrous Na_2SO_4 and the solvent was removed under reduced pressure. The residue was purified by silica gel column chromatography affording MBH carbonate **2**.

Methyl 2-(((tert-butoxycarbonyl)oxy)(phenyl)methyl)acrylate (2a)

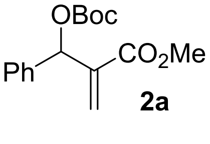

2a

white solid, 81% yields, 2.37g. **^{1}H NMR** (600 MHz, $CDCl_3$): δ 7.41 – 7.29 (m, 5H), 6.48 (s, 1H), 6.41 (s, 1H), 5.91 (s, 1H), 3.71 (s, 3H), 1.46 (s, 9H). **^{13}C NMR** (151 MHz, $CDCl_3$): δ 165.5, 152.5, 139.8, 137.6, 128.6, 128.6, 127.8, 126.0, 82.8, 75.9, 52.1, 27.9.

Methyl 2-(((tert-butoxycarbonyl)oxy)(p-tolyl)methyl)acrylate (2b)

---

[2] J. T. M. Correia, L.V. Acconcia, F. Coelho, *Eur. J. Org. Chem.* **2016**, *11*, 1972-1976.

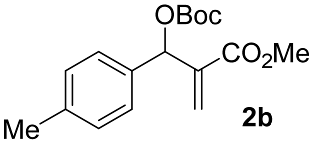
2b

white solid, 72% yields, 2.20g. **^{1}H NMR** (500 MHz, $CDCl_3$): δ 7.28 (d, J = 8.2 Hz, 2H), 7.14 (d, J = 7.9 Hz, 2H), 6.45 (s, 1H), 6.39 (t, J = 1.0 Hz, 1H), 5.91 (t, J = 1.3 Hz, 1H), 3.70 (s, 3H), 2.33 (s, 3H), 1.46 (s, 9H). **^{13}C NMR** (126 MHz, $CDCl_3$): δ 165.58, 152.57, 139.85, 138.40, 134.61, 129.29, 127.75, 125.72, 82.67, 75.85, 52.11, 27.89, 21.33.

Methyl 2-(((tert-butoxycarbonyl)oxy)(4-methoxyphenyl)methyl)acrylate (2c)

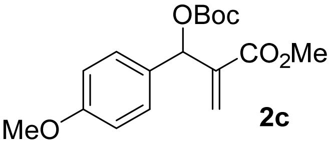
2c

white solid, 75% yields, 2.42g. **^{1}H NMR** (600 MHz, $CDCl_3$): δ 7.35 – 7.29 (m, 2H), 6.89 – 6.82 (m, 2H), 6.43 (s, 1H), 6.38 (s, 1H), 5.92 (s, 1H), 3.79 (s, 3H), 3.70 (s, 3H), 1.45 (s, 9H). **^{13}C NMR** (126 MHz, $CDCl_3$): δ 165.6, 159.8, 152.6, 139.9, 129.6, 129.3, 125.3, 114.0, 82.7, 75.7, 55.4, 52.1, 27.9.

Methyl 2-(((tert-butoxycarbonyl)oxy)(4-fluorophenyl)methyl)acrylate (2d)

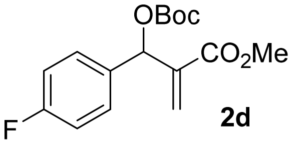
2d

yellow solid, 59% yields, 1.83g. **^{1}H NMR** (500 MHz, $CDCl_3$): δ 7.42 – 7.35 (m, 2H), 7.02 (t, J = 8.7 Hz, 2H), 6.44 (d, J = 1.1 Hz, 1H), 6.40 (t, J = 0.9 Hz, 1H), 5.94 (dd, J = 1.5, 0.9 Hz, 1H), 3.71 (s, 3H), 1.46 (s, 9H). **^{13}C NMR** (126 MHz, $CDCl_3$): δ 165.3, 162.7 (d, J = 248.2 Hz), 152.3, 139.5, 133.4 (d, J = 3.2 Hz), 129.6 (d, J = 8.6), 125.6, 115.4 (d, J = 21.4), 82.9, 52.1, 27.7. ^{19}F NMR (565 MHz, $CDCl_3$):δ -113.4.

Methyl 2-(((tert-butoxycarbonyl)oxy)(4-chlorophenyl)methyl)acrylate (2e)

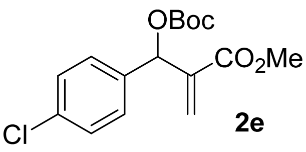
2e

white solid, 62% yields, 2.02g. **^{1}H NMR** (500 MHz, $CDCl_3$): δ 7.36 – 7.29 (m, 4H), 6.43 (d, J = 1.2 Hz, 1H), 6.41 (t, J = 0.9 Hz, 1H), 5.94 (dd, J = 1.5, 0.8 Hz, 1H), 3.71 (s, 3H), 1.46 (s, 9H). **^{13}C NMR** (126 MHz, $CDCl_3$): δ 165.3, 152.4, 139.4, 136.3, 134.5, 129.2, 128.8, 126.1, 83.1, 75.2, 52.2, 27.9.

Methyl 2-(((tert-butoxycarbonyl)oxy)(naphthalen-2-yl)methyl)acrylate (2f)

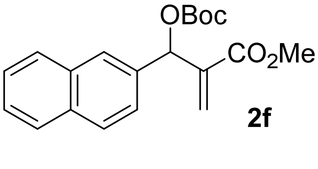
2f

white solid, 77% yields, 2.63g. **^{1}H NMR** (500 MHz, $CDCl_3$): δ 7.90 – 7.78 (m, 4H), 7.54 – 7.45 (m, 3H), 6.66 (s, 1H), 6.45 (d, J = 0.9 Hz, 1H), 6.04 – 5.97 (m, 1H), 3.71 (s, 3H), 1.47 (s, 9H). **^{13}C NMR** (126 MHz, $CDCl_3$): δ 165.6, 152.6, 139.7, 135.0,

133.4, 133.2, 128.4, 128.4, 127.8, 127.2, 126.5, 126.3, 126.3, 125.3, 82.9, 76.0, 52.2, 27.9.

Methyl 2-(((tert-butoxycarbonyl)oxy)(thiophen-2-yl)methyl)acrylate (2g)

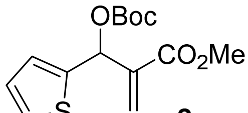

2g

Colorless oil, 75% yields, 2.24g. **^{1}H NMR** (500 MHz, $CDCl_3$): δ 7.31 – 7.27 (m, 1H), 7.09 – 7.08 (m, 1H), 6.97 – 6.94 (m, 1H), 6.73 (s, 1H), 6.44 (s, 1H), 6.07 – 6.03 (m, 1H), 3.73 (s, 3H), 1.47 (s, 9H). **^{13}C NMR** (126 MHz, $CDCl_3$): δ 165.3, 152.3, 140.5, 139.4, 127.5, 126.9, 126.6, 125.9, 83.1, 71.0, 52.3, 27.9.

Ethyl 2-(((tert-butoxycarbonyl)oxy)(phenyl)methyl)acrylate (2h)

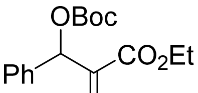

2h

Colorless oil, 80% yields, 2.45g. **^{1}H NMR** (500 MHz, $CDCl_3$): δ 7.41 – 7.29 (m, 5H), 6.49 – 6.47 (m, 1H), 6.40 (t, J = 1.0 Hz, 1H), 5.90 – 5.88 (m, 1H), 4.22 – 4.10 (m, 2H), 1.46 (s, 9H), 1.22 (t, J = 7.1 Hz, 3H). **^{13}C NMR** (126 MHz, $CDCl_3$): δ 165.1, 152.6, 140.0, 137.7, 128.6, 128.5, 127.9, 125.7, 82.8, 76.0, 61.1, 27.9, 14.1.

Benzyl 2-(((tert-butoxycarbonyl)oxy)(phenyl)methyl)acrylate (2i)

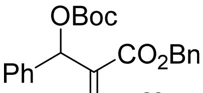

2i

Colorless oil, 67% yields, 2.47g. **^{1}H NMR** (600 MHz, $CDCl_3$): δ 7.41 – 7.28 (m, 8H), 7.25 – 7.20 (m, 2H), 6.49 (s, 1H), 6.46 (s, 1H), 5.94 (s, 1H), 5.20 – 5.08 (m, 2H), 1.44 (s, 9H). **^{13}C NMR** (151 MHz, $CDCl_3$): δ 164.9, 152.5, 139.7, 137.6, 135.6, 128.64, 128.61, 128.3, 128.2, 127.9, 127.1, 126.3, 82.8, 76.0, 66.9, 27.9.

Butyl 2-(((tert-butoxycarbonyl)oxy)(phenyl)methyl)acrylate (2j)

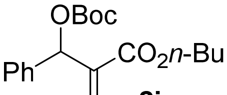

2j

Colorless oil, 71% yields, 2.37g. **^{1}H NMR** (500 MHz, $CDCl_3$): δ 7.41 – 7.37 (m, 2H), 7.35 – 7.29 (m, 3H), 6.47 (s, 1H), 6.41 (t, J = 1.1 Hz, 1H), 5.91 – 5.85 (m, 1H), 4.14 – 4.07 (m, 2H), 1.59 – 1.55 (m, 2H), 1.46 (s, 9H), 1.29 – 1.25 (m, 2H), 0.88 (t, J = 7.4 Hz, 3H). **^{13}C NMR** (126 MHz, $CDCl_3$): δ 165.2, 152.5, 139.9, 137.7, 128.6, 128.1, 127.9, 125.8, 82.8, 76.0, 65.0, 30.6, 27.9, 19.2, 13.8.

Isobutyl 2-(((tert-butoxycarbonyl)oxy)(phenyl)methyl)acrylate (2k)

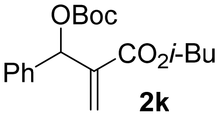

2k

Colorless oil, 76% yields, 2.54g. **^{1}H NMR** (500 MHz, $CDCl_3$): δ

7.42 – 7.29 (m, 5H), 6.48 (s, 1H), 6.43 (t, J = 1.0 Hz, 1H), 5.89 (t, J = 1.3 Hz, 1H), 3.94 – 3.83 (m, 2H), 1.95 – 1.83 (m, 1H), 1.46 (s, 9H), 0.86 (dd, J = 6.8, 2.4 Hz, 6H). **^{13}C NMR** (126 MHz, $CDCl_3$): δ165.2, 152.6, 139.9, 137.7, 128.6 (2C), 127.9, 126.0, 82.7, 76.0, 71.2, 27.9, 19.2.

III. Chiral phosphine catalyzed allylic alkylations

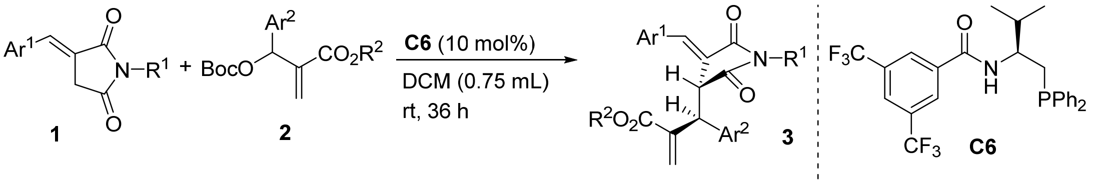

To a solution of DCM (0.75 mL) were added *N*-protected succinimide substrate **1** (1 equiv, 0.05 mmol), MBH carbonate **2** (1.2 equiv, 0.06 mmol) and catalyst **C6** (2.5mg, 10 mol%, 0.005 mmol). The reaction mixture was stirred at r.t. for 36h. Then the mixture was purified by preparative TLC (eluent: PE/EA = 2:1) to yield the desired product **3**.

Methyl 2-((*S*)-((*R*)-4-((*E*)-benzylidene)-2,5-dioxo-1-phenylpyrrolidin-3-yl)(phenyl) methyl)acrylate (3aa)

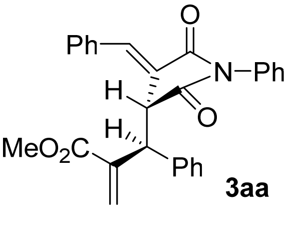

3aa

pale yellow solid, 96% yield (21.0 mg, 11:1 dr), mp: 160.6 – 161.6°C. $[\alpha]_D^{25}$: +110 (c = 1.0, $CHCl_3$). HPLC analysis of the product: Daicel CHIRALPAK IF-3 column; 20% ^{i}PrOH in hexanes; 1.0 mL/min; retention times: 12.52 min (major), 14.788 min (minor). 81% ee. **^{1}H NMR** (500 MHz, $CDCl_3$): δ 7.74 (d, J = 2.2 Hz, 1H), 7.73 – 7.68 (m, 2H), 7.59 – 7.49 (m, 3H), 7.43 – 7.40 (m, 2H), 7.38 – 7.36 (m, 1H), 7.28 – 7.21 (m, 3H), 6.97 – 6.90 (m, 4H), 6.63 (d, J = 1.2 Hz, 1H), 6.29 (d, J = 1.8 Hz, 1H), 4.87 (t, J = 2.7 Hz, 1H), 4.66 – 4.61 (m, 1H), 3.75 (s, 3H). **^{13}C NMR** (126 MHz, $CDCl_3$): δ 174.7, 169.4, 167.3, 140.0, 136.3, 136.0, 133.5, 131.8, 130.63, 130.62, 129.4, 129.1, 129.0, 128.73, 128.71, 128.65, 128.1, 127.7, 126.5, 52.3, 46.5, 46.5. **HRMS** (CI+) Calcd for $C_{28}H_{24}NO_4^+$ $[M + H]^+$: 438.1705, Found: 438.1700.

Methyl 2-((*S*)-((*R*)-4-((*E*)-benzylidene)-1-methyl-2,5-dioxopyrrolidin-3-yl)(phenyl) methyl)acrylate (3ba)

white solid, 91% yield (17.1 mg, 5:1 dr), mp: 74.0 – 76.0 °C. $[\alpha]_D^{25}$: +240 (c = 1.0, $CHCl_3$). HPLC analysis of the product: Daicel CHIRALPAK IE-3 column; 10% ^{i}PrOH in hexanes; 1.0 mL/min; retention times: 18.694 min (minor), 19.832 min (major). 81% ee. **^{1}H NMR** (400 MHz, $CDCl_3$): δ 7.63 (dd, J = 7.1, 1.8 Hz, 2H), 7.56

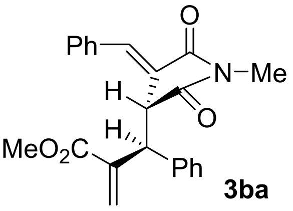

3ba

(d, J = 2.1 Hz, 1H), 7.54 – 7.27 (m, 5H), 7.21 – 7.09 (m, 3H), 6.80 – 6.71 (m, 2H), 6.55 (d, J = 1.4 Hz, 1H), 6.10 (d, J = 1.8 Hz, 1H), 4.70 (m, 1H), 4.52 – 4.46 (m, 1H), 3.74 (s, 3H), 2.82 (s, 3H). **^{13}C NMR** (101 MHz, $CDCl_3$): δ 175.9, 170.4, 167.4, 140.0, 136.2, 134.9, 133.6, 130.5, 129.3, 129.0, 128.7, 128.5, 128.4, 127.9, 127.8, 52.3, 46.24, 46.18, 24.6. **HRMS** (CI+) Calcd for $C_{23}H_{22}NO_4^+$ [M + H]$^+$: 376.1549, Found: 376.1540.

Methyl 2-((*S*)-((*R*)-1-benzyl-4-((*E*)-benzylidene)-2,5-dioxopyrrolidin-3-yl)(phenyl) methyl)acrylate (3ca)

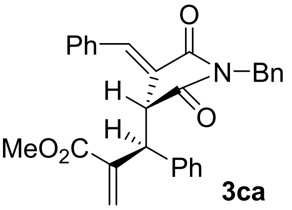

3ca

white solid, 96% yield (21.7 mg, 11:1 dr), mp: 75.6 – 77.0 °C. $[\alpha]_D^{25}$: +158 (c = 1.0, $CHCl_3$). HPLC analysis of the product: Daicel CHIRALPAK IE-3 column; 10% ^{i}PrOH in hexanes; 1.0 mL/min; retention times: 13.358 min (minor), 14.456 min (major). 92% ee. **^{1}H NMR** (500 MHz, $CDCl_3$): δ 7.57 – 7.52 (m, 2H), 7.48 (d, J = 2.3 Hz, 1H), 7.46 – 7.36 (m, 3H), 7.21 – 7.18 (m, 5H), 7.00 – 6.94 (m, 1H), 6.84 – 6.77 (m, 2H), 6.50 – 6.44 (m, 2H), 6.41 (d, J = 1.6 Hz, 1H), 5.78 (d, J = 1.8 Hz, 1H), 4.67 (dd, J = 4.0, 2.3 Hz, 1H), 4.50 (s, 2H), 4.44 – 4.40 (m, 1H), 3.68 (s, 3H). **^{13}C NMR** (126 MHz, $CDCl_3$): δ 175.5, 170.1, 167.5, 140.2, 135.9, 133.7, 130.4, 130.3, 129.24, 129.23, 128.8, 128.7, 128.5, 128.4, 127.9, 127.7, 127.6, 52.3, 45.8, 45.6, 42.5. **HRMS** (CI+) Calcd for $C_{29}H_{26}NO_4^+$ [M + H]$^+$: 452.1862, Found: 452.1857.

Methyl 2-((*S*)-((*R)*-4-((*E*)-benzylidene)-1-butyl-2,5-dioxopyrrolidin-3-yl)(phenyl) methyl)acrylate (3da)

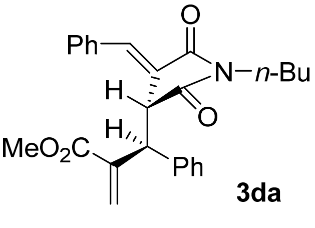

3da

white solid, 91% yield (19.0 mg, 11:1 dr), mp: 71.6 – 73.0 °C. $[\alpha]_D^{25}$: +315 (c = 1.0, $CHCl_3$). HPLC analysis of the product: Daicel CHIRALPAK IE-3 column; 10% ^{i}PrOH in hexanes; 1.0 mL/min; retention times: 13.312 min (minor), 14.771 min (major). 82% ee. **^{1}H NMR** (400 MHz, $CDCl_3$): δ 7.63 – 7.55 (m, 3H), 7.52 – 7.42 (m, 3H), 7.19 – 7.07 (m, 3H), 6.84 – 6.76 (m, 2H), 6.59 (d, J = 1.2

Hz, 1H), 6.23 (d, J = 1.7 Hz, 1H), 4.63 (t, J = 2.7 Hz, 1H), 4.52 – 4.50 (m, 1H), 3.70 (s, 3H), 3.35 (td, J = 7.1, 4.0 Hz, 2H), 1.21 (dt, J = 14.3, 7.8 Hz, 2H), 1.16 – 1.02 (m, 2H), 0.83 (t, J = 7.2 Hz, 3H). **^{13}C NMR** (126 MHz, $CDCl_3$): δ 175.7, 170.4, 167.3, 140.4, 136.3, 134.8, 133.6, 130.5, 130.3, 129.3, 128.9, 128.49, 128.47, 128.0, 127.9, 52.3, 46.2, 45.9, 38.6, 29.7, 20.2, 13.8. **HRMS** (CI+) Calcd for $C_{26}H_{28}NO_4^+$ $[M + H]^+$: 418.2018, Found: 418.2008.

Methyl 2-((*S*)-((*R*)-4-((*E*)-benzylidene)-1-(tert-butyl)-2,5-dioxopyrrolidin-3-yl)(phenyl)methyl)acrylate (3ea)

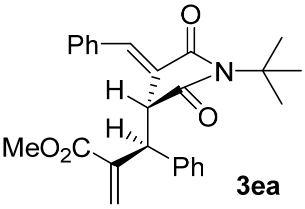

3ea

white solid, 94% yield (19.6 mg, 14:1 dr), mp: 63.2 – 64.7 °C. $[\alpha]_D^{25}$: +310 (c = 1.0, $CHCl_3$). HPLC analysis of the product: Daicel CHIRALPAK IE-3 column; 10% ^{i}PrOH in hexanes; 1.0 mL/min; retention times: 7.653 min (minor), 8.803 min (major). 92% ee. **^{1}H NMR** (400 MHz, $CDCl_3$): δ 7.62 – 7.39 (m, 6H), 7.19 – 7.17 (m, 3H), 6.93 – 6.83 (m, 2H), 6.59 (t, J = 0.9 Hz, 1H), 6.29 (d, J = 1.6 Hz, 1H), 4.49 (t, J = 2.6 Hz, 1H), 4.47 – 4.45 (m, 1H), 3.68 (s, 3H), 1.35 (s, 9H). **^{13}C NMR** (101 MHz, $CDCl_3$): δ 176.6, 171.5, 167.3, 140.6, 136.4, 133.8, 133.7, 130.4, 130.1, 130.0, 129.2, 129.0, 128.51, 128.47, 128.3, 127.8, 58.5, 52.3, 46.5, 46.4, 28.3. **HRMS** (CI+) Calcd for $C_{26}H_{28}NO_4^+$ $[M + H]^+$: 418.2018, Found: 418.2013.

Methyl 2-((*S*)-((*R*)-4-((*E*)-benzylidene)-1-(naphthalen-1-ylmethyl)-2,5-dioxopyrrolidin-3-yl)(phenyl)methyl)acrylate (3fa)

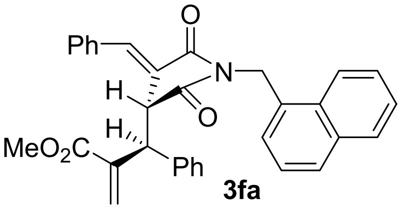

3fa

white solid, 91% yield (22.8 mg, 13:1 dr), mp: 97.6 – 99.8 °C. $[\alpha]_D^{25}$: +120 (c = 1.0, $CHCl_3$). HPLC analysis of the product: Daicel CHIRALPAK IE-3 column; 10% ^{i}PrOH in hexanes; 1.0 mL/min; retention times: 23.246 min (minor), 24.53 min (major). 81% ee. **^{1}H NMR** (500 MHz, $CDCl_3$): δ 8.33 – 8.21 (m, 1H), 7.87 – 7.85 (m, 1H), 7.80 – 7.78 (m, 1H), 7.67 – 7.58 (m, 3H), 7.52 – 7.47 (m, 3H), 7.47 – 7.42 (m, 1H), 7.38 – 7.32 (m, 2H), 6.82 (tt, J = 7.2, 1.2 Hz, 1H), 6.61 (t, J = 7.8 Hz, 2H), 6.53 – 6.45 (m, 3H), 5.95 (d, J = 1.8 Hz, 1H), 5.04 (q, J = 12.4 Hz, 2H), 4.72 (t, J = 3.0 Hz, 1H), 4.49 – 4.48 (m, 1H), 3.72 (s,

3H). **^{13}C NMR** (126 MHz, $CDCl_3$): δ 175.7, 170.2, 167.4, 140.1, 135.7, 135.6, 133.9, 133.6, 131.6, 130.52, 130.47, 130.4, 129.3, 128.72, 128.66, 128.63, 128.56, 128.4, 128.2, 127.6, 127.5, 126.6, 125.9, 125.4, 124.0, 52.3, 46.1, 45.7, 40.4. **HRMS** (CI+) Calcd for $C_{33}H_{28}NO_4^+$ $[M + H]^+$: 502.2018, Found: 502.2012.

Methyl 2-((*S*)-((*R*)-1-benzyl-4-((*E*)-4-methylbenzylidene)-2,5-dioxopyrrolidin-3-yl)(phenyl)methyl) acrylate (3ga)

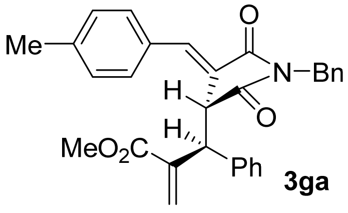

3ga

white solid, 86% yield (20.0 mg, 10:1 dr), mp: 76.7 – 77.8 °C. $[\alpha]_D^{25}$: +152 (c = 1.0, $CHCl_3$). HPLC analysis of the product: Daicel CHIRALPAK IE-3 column; 10% ^{i}PrOH in hexanes; 1.0 mL/min; retention times: 18.464 min (minor), 23.692 min (major). 90% ee. **^{1}H NMR** (500 MHz, $CDCl_3$): δ 7.49 – 7.41 (m, 3H), 7.23 (d, *J* = 7.9 Hz, 2H), 7.20 -7.16 (m, 5H), 6.97 (t, *J* = 7.4 Hz, 1H), 6.81 (t, *J* = 7.8 Hz, 2H), 6.49 (dd, *J* = 8.2, 1.3 Hz, 2H), 6.41 (d, *J* = 1.4 Hz, 1H), 5.82 (d, *J* = 1.8 Hz, 1H), 4.63 (dd, *J* = 4.0, 2.3 Hz, 1H), 4.48 (s, 2H), 4.47 - 4.46 (m, 1H), 3.69 (s, 3H), 2.34 (s, 3H). **^{13}C NMR** (126 MHz, $CDCl_3$): δ 175.6, 170.3, 167.6, 140.9, 140.2, 136.0, 135.5, 130.8, 130.6, 130.4, 130.0, 129.2, 128.8, 128.7, 128.4, 128.3, 127.9, 127.7, 126.5, 52.3, 45.9, 45.6, 42.5, 21.7. **HRMS** (CI+) Calcd for $C_{30}H_{28}NO_4^+$ $[M + H]^+$: 466.2018, Found: 466.2008.

Methyl 2-((*S*)-((*R*)-1-benzyl-4-((*E*)-4-(methylthio)benzylidene)-2,5-dioxopyrrolidin-3-yl)(phenyl)methyl)acrylate (3ha)

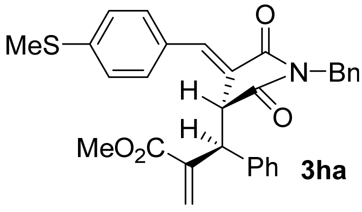

3ha

white solid, 93% yield (23.1 mg, 11:1 dr), mp: 91.6 – 92.3 °C. $[\alpha]_D^{25}$: +165 (c = 1.0, $CHCl_3$). HPLC analysis of the product: Daicel CHIRALPAK IE-3 column; 10% ^{i}PrOH in hexanes; 1.0 mL/min; retention times: 27.053 min (minor), 29.227 min (major). 84% ee. **^{1}H NMR** (500 MHz, $CDCl_3$): δ 7.54 (d, *J* = 8.5 Hz, 2H), 7.46 (d, *J* = 2.1 Hz, 1H), 7.31 (d, *J* = 8.5 Hz, 2H), 7.25 (d, *J* = 3.7 Hz, 5H), 7.06 – 6.99 (m, 1H), 6.87 (t, *J* = 7.8 Hz, 2H), 6.55 (dd, *J* = 8.1, 1.4 Hz, 2H), 6.47 (d, *J* = 1.5 Hz, 1H), 5.83 (d, *J* = 1.9 Hz, 1H), 4.69 (dd, *J* = 4.1, 2.2 Hz, 1H), 4.55 (s, 2H), 4.51 (dd, *J* = 4.0, 1.9 Hz, 1H), 3.77 (s, 3H), 2.53 (s, 3H). **^{13}C NMR** (126 MHz,

$CDCl_3$): δ 175.5, 170.2, 167.6, 142.4, 140.1, 135.9, 135.5, 134.9, 130.9, 129.9, 129.3, 128.8, 128.7, 128.5, 128.4, 127.9, 127.8, 126.4, 126.0, 52.4, 45.8, 45.6, 42.5, 15.1. **HRMS** (CI+) Calcd for $C_{30}H_{28}NO_4S^+$ [M + H]$^+$: 498.1739, Found: 498.1731.

Methyl 2-((*S*)-((*R*)-1-benzyl-4-((*E*)-4-methoxybenzylidene)-2,5-dioxopyrrolidin-3-yl)(phenyl)methyl)acrylate (3ia)

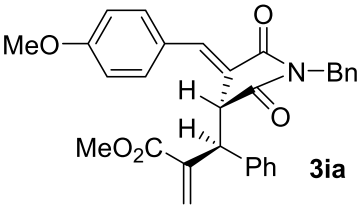

3ia

white solid, 92% yield (22.1 mg, 9:1 dr), mp: 102.9 – 103.6 °C. $[\alpha]_D^{25}$: +168 (c = 1.0, $CHCl_3$). HPLC analysis of the product: Daicel CHIRALPAK IE-3 column; 10% ^{i}PrOH in hexanes; 1.0 mL/min; retention times: 35.278 min (minor), 37.542 min (major). 91% ee. **^{1}H NMR** (500 MHz, $CDCl_3$): δ 7.59 (d, *J* = 8.8 Hz, 2H), 7.50 (d, *J* = 2.2 Hz, 1H), 7.29 – 7.24 (m, 5H), 7.07 – 6.97 (m, 3H), 6.89 (t, *J* = 7.8 Hz, 2H), 6.63 – 6.56 (m, 2H), 6.50 (d, *J* = 1.4 Hz, 1H), 5.93 (d, *J* = 1.8 Hz, 1H), 4.68 – 4.64 (m, 1H), 4.57 – 4.55 (m, 1H), 4.54 (s, 2H), 3.88 (s, 3H), 3.76 (s, 3H). **^{13}C NMR** (126 MHz, $CDCl_3$): δ 175.6, 170.4, 167.6, 161.3, 140.3, 135.9, 135.6, 135.2, 132.5, 129.2, 128.8, 128.7, 128.40, 128.36, 127.8, 127.7, 126.2, 124.9, 114.7, 55.6, 52.3, 45.9, 45.6, 42.4. **HRMS** (CI+) Calcd for $C_{30}H_{28}NO_5^+$ [M + H]$^+$: 482.1967, Found: 482.1958.

Methyl 2-((*S*)-((*R*)-1-benzyl-4-((*E*)-3,5-dimethoxybenzylidene)-2,5-dioxopyrrolidin-3-yl)(phenyl)methyl)acrylate (3ja)

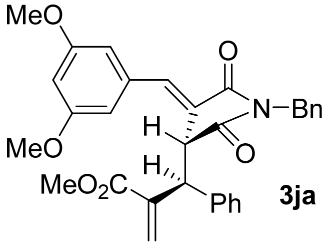

3ja

white solid, 90% yield (23.0 mg, 11:1 dr), mp: 84.5 – 86.7 °C. $[\alpha]_D^{25}$: +101 (c = 1.0, $CHCl_3$). HPLC analysis of the product: Daicel CHIRALPAK IF-3 column; 10% ^{i}PrOH in hexanes; 1.0 mL/min; retention times: 18.95 min (minor), 20.149 min (major). 98% ee. **^{1}H NMR** (500 MHz, $CDCl_3$): δ 7.47 (d, *J* = 2.3 Hz, 1H), 7.29 – 7.26 (m, 5H), 7.05 (t, *J* = 7.4 Hz, 1H), 6.90 (t, *J* = 7.6 Hz, 2H), 6.72 (d, *J* = 2.3 Hz, 2H), 6.61 – 6.56 (m, 2H), 6.55 (t, *J* = 2.3 Hz, 1H), 6.47 (d, *J* = 1.5 Hz, 1H), 5.84 (d, *J* = 1.9 Hz, 1H), 4.77 (dd, *J* = 4.2, 2.3 Hz, 1H), 4.56 (s, 3H), 3.85 (s, 6H), 3.74 (s, 3H). **^{13}C NMR** (126 MHz, $CDCl_3$): δ 175.3, 170.0, 167.3, 161.1, 140.4, 135.9, 135.5, 135.4, 129.1, 128.7, 128.6, 128.3,

128.2, 127.9, 127.8, 127.6, 107.9, 103.0, 55.6, 52.2, 45.9, 45.6, 42.4. **HRMS** (CI+) Calcd for $C_{31}H_{30}NO_6^+$ [M + H]$^+$: 512.2073, Found: 512.2065.

Methyl 2-((*S*)-((*R*)-1-benzyl-4-((*E*)-2-methoxybenzylidene)-2,5-dioxopyrrolidin-3-yl)(phenyl)methyl)acrylate (3ka)

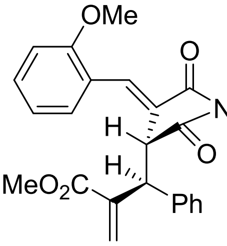

3ka

white solid, 94% yield (22.6 mg, 3:1 dr), mp: 59.9 – 62.3 °C. $[\alpha]_D^{25}$: +40 (c = 1.0, $CHCl_3$). HPLC analysis of the product: Daicel CHIRALPAK IE-3 column; 10% ^{i}PrOH in hexanes; 1.0 mL/min; retention times: 25.547 min (minor), 31.706 min (major). 28% ee. **^{1}H NMR** (500 MHz, $CDCl_3$): δ 7.86 (d, J = 2.4 Hz, 1H), 7.57 – 7.55 (m, 1H), 7.44 – 7.40 (m, 1H), 7.25 – 7.14 (m, 5H), 7.08 – 7.02 (m, 2H), 6.97 (d, J = 8.3 Hz, 1H), 6.88 (t, J = 7.7 Hz, 2H), 6.55 (d, J = 7.8 Hz, 2H), 6.44 (d, J = 1.3 Hz, 1H), 5.89 (d, J = 1.8 Hz, 1H), 4.69 – 4.66 (m, 1H), 4.58 (d, J = 4.0 Hz, 2H), 4.33 – 4.29 (m, 1H), 3.88 (s, 3H), 3.69 (s, 3H). **^{13}C NMR** (126 MHz, $CDCl_3$): δ 175.6, 170.0, 167.3, 158.3, 140.6, 136.1, 135.5, 131.7, 131.5, 130.0, 129.1, 129.0, 128.7, 128.5, 128.2, 127.9, 127.7, 127.4, 122.7, 120.7, 111.0, 55.6, 52.1, 46.2, 45.4, 42.3. **HRMS** (CI+) Calcd for $C_{30}H_{28}NO_5^+$ [M + H]$^+$: 482.1967, Found: 482.1958.

Methyl 2-((*S*)-((*R*)-1-benzyl-4-((*E*)-4-fluorobenzylidene)-2,5-dioxopyrrolidin-3-yl)(phenyl)methyl)acrylate (3la)

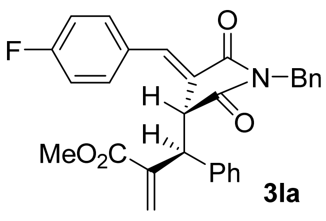

3la

white solid, 92% yield (21.6 mg, 9:1 dr), mp: 62.8 – 63.5 °C. $[\alpha]_D^{25}$: +102 (c = 1.0, $CHCl_3$). HPLC analysis of the product: Daicel CHIRALPAK IE-3 column; 10% ^{i}PrOH in hexanes; 1.0 mL/min; retention times: 13.027 min (minor), 14.371 min (major). 76% ee. **^{1}H NMR** (500 MHz, $CDCl_3$): δ 7.67 – 7.60 (m, 2H), 7.50 (d, J = 2.4 Hz, 1H), 7.28 – 7.27 (m, 4H), 7.23 – 7.15 (m, 2H), 7.07 – 7.01 (m, 1H), 6.88 (t, J = 7.8 Hz, 2H), 6.53 (dt, J = 8.3, 1.4 Hz, 2H), 6.47 (d, J = 1.5 Hz, 1H), 5.79 (d, J = 1.8 Hz, 1H), 4.70 (dd, J = 4.2, 2.3 Hz, 1H), 4.58 (s, 2H), 4.47 – 4.44 (m, 1H), 3.78 (s, 3H). **^{13}C NMR** (126 MHz, $CDCl_3$): δ 175.3, 169.9, 167.5, 163.6 (d ,J = 253.1 Hz), 139.9, 135.6, 135.3, 134.1, 132.4 (d, J = 8.6 Hz) , 129.79, 129.76,

129.2, 129.1, 128.64, 128.62, 128.41, 128.36, 127.9, 127.7, 127.1 (d, *J* = 2.0 Hz), 116.4 (d, *J* = 21.9 Hz), 52.3, 45.5, 45.4, 42.4. **^{19}F NMR** (376 MHz, $CDCl_3$): *δ* -108.8. **HRMS** (CI+) Calcd for $C_{29}H_{25}FNO_4^+$ [M + H]$^+$: 470.1768, Found: 470.1757.

Methyl 2-((*S*)-((*R*)-1-benzyl-4-((*E*)-4-chlorobenzylidene)-2,5-dioxopyrrolidin-3-yl)(phenyl)methyl)acrylate (3ma)

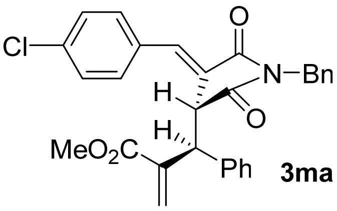

3ma

white solid, 93% yield (22.6 mg, 13:1 dr), mp: 89.4 – 92.0 °C. $[\alpha]_D^{25}$: +85 (c = 1.0, $CHCl_3$). HPLC analysis of the product: Daicel CHIRALPAK IE-3 column; 10% ^{i}PrOH in hexanes; 1.0 mL/min; retention times: 13.149 min (minor), 14.72 min (major). 78% ee. **^{1}H NMR** (500 MHz, $CDCl_3$): *δ* 7.60 – 7.55 (m, 2H), 7.50 – 7.45 (m, 3H), 7.29 (s, 5H), 7.05 (tt, *J* = 7.4, 1.2 Hz, 1H), 6.87 (t, *J* = 7.8 Hz, 2H), 6.54 – 6.49 (m, 2H), 6.46 (d, *J* = 1.7 Hz, 1H), 5.75 (d, *J* = 1.9 Hz, 1H), 4.76 – 4.68 (m, 1H), 4.59 (d, *J* = 1.6 Hz, 2H), 4.45 – 4.43 (m, 1H), 3.79 (s, 3H). **^{13}C NMR** (126 MHz, $CDCl_3$): *δ* 175.3, 169.9, 167.6, 139.9, 136.4, 135.7, 135.4, 134.1, 132.1, 131.6, 129.6, 129.3, 128.8, 128.7, 128.54, 128.50, 128.1, 128.0, 127.8, 52.4, 45.63, 45.58, 42.6. **HRMS** (CI+) Calcd for $C_{29}H_{25}ClNO_4^+$ [M + H]$^+$: 486.1472, Found: 486.1464.

Methyl 2-((*S*)-((*R*)-1-benzyl-4-((*E*)-4-bromobenzylidene)-2,5-dioxopyrrolidin-3-yl) (phenyl)methyl)acrylate (3na)

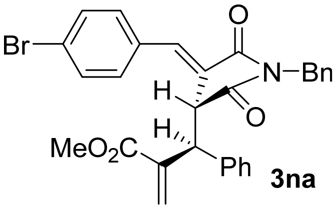

3na

white solid, 92% yield (24.4 mg, 11:1 dr), mp: 90.4 – 93.4 °C. $[\alpha]_D^{25}$: +98 (c = 1.0, $CHCl_3$). HPLC analysis of the product: Daicel CHIRALPAK IE-3 column; 10% ^{i}PrOH in hexanes; 1.0 mL/min; retention times: 13.869 min (minor), 15.455 min (major). 79% ee. **^{1}H NMR** (500 MHz, $CDCl_3$): *δ* 7.68 – 7.62 (m, 2H), 7.54 – 7.48 (m, 2H), 7.45 (d, *J* = 2.3 Hz, 1H), 7.29 – 7.29 (m, 4H), 7.11 – 7.00 (m, 1H), 6.90 – 6.81 (m, 2H), 6.54 – 6.49 (m, 2H), 6.46 (d, *J* = 1.5 Hz, 1H), 5.74 (d, *J* = 1.9 Hz, 1H), 4.74 – 4.69 (m, 1H), 4.59 (d, *J* = 1.9 Hz, 2H), 4.46 – 4.41 (m, 1H), 3.79 (s, 3H). **^{13}C NMR** (126 MHz, $CDCl_3$): *δ* 175.3, 169.9, 167.6, 139.9, 135.7, 135.4, 134.1, 132.55, 132.52, 131.8, 129.4, 128.8, 128.7, 128.54, 128.50, 128.3,

128.0, 127.8, 124.8, 52.4, 45.57, 45.56, 42.6. **HRMS** (CI+) Calcd for $C_{29}H_{25}BrNO_4^+$ $[M + H]^+$: 530.0967, Found: 530.0956.

Methyl 2-((*S*)-((*R*,*E*)-1-benzyl-4-(naphthalen-2-ylmethylene)-2,5-dioxopyrrolidin-3-yl)(phenyl)methyl)acrylate (3oa)

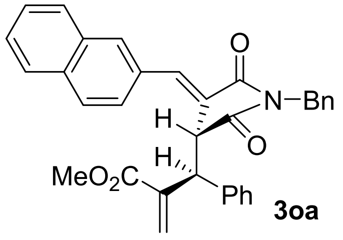

3oa

white solid, 93% yield (23.3 mg, 11:1 dr), mp: 81.3 – 82.1 °C. $[\alpha]_D^{25}$: +158 (c = 1.0, $CHCl_3$). HPLC analysis of the product: Daicel CHIRALPAK IE-3 column; 10% ^{i}PrOH in hexanes; 1.0 mL/min; retention times: 20.734 min (minor), 24.482 min (major). 85% ee. **^{1}H NMR** (500 MHz, $CDCl_3$): δ 8.20 (s, 1H), 7.99 – 7.94 (m, 2H), 7.91 – 7.86 (m, 1H), 7.73 – 7.66 (m, 2H), 7.61 – 7.52 (m, 2H), 7.29 (s, 5H), 7.04 (tt, J = 7.2, 1.2 Hz, 1H), 6.86 (t, J = 7.8 Hz, 2H), 6.56 – 6.44 (m, 3H), 5.80 (d, J = 1.9 Hz, 1H), 4.89 (dd, J = 4.1, 2.3 Hz, 1H), 4.60 (d, J = 5.7 Hz, 3H), 3.76 (s, 3H). **^{13}C NMR** (126 MHz, $CDCl_3$): δ 175.6, 170.2, 167.6, 140.2, 135.8, 135.54, 135.51, 133.9, 133.4, 131.4, 131.1, 129.3, 129.00, 128.96, 128.8, 128.7, 128.5, 128.4, 127.95, 127.87, 127.8, 127.7, 127.6, 127.0, 126.8, 52.3, 46.0, 45.7, 42.6. **HRMS** (CI+) Calcd for $C_{33}H_{28}NO_4^+$ $[M + H]^+$: 502.2018, Found: 502.2007.

Methyl 2-((*S*)-((*R*)-4-((*E*)-benzylidene)-1-(tert-butyl)-2,5-dioxopyrrolidin-3-yl)(p-tolyl)methyl)acrylate (3eb)

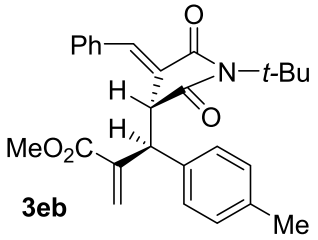

3eb

white solid, 91% yield (19.6 mg, 15:1 dr), mp: 57.5 – 59.1 °C. $[\alpha]_D^{25}$: +323 (c = 1.0, $CHCl_3$). HPLC analysis of the product: Daicel CHIRALPAK IE-3 column; 1% ^{i}PrOH in hexanes; 1.0 mL/min; retention times: 9.767 min (minor), 17.787 min (major). 92% ee. **^{1}H NMR** (500 MHz, $CDCl_3$): δ 7.60 – 7.55 (m, 2H), 7.51 (d, J = 2.3 Hz, 1H), 7.49 – 7.39 (m, 3H), 6.97 (d, J = 7.8 Hz, 2H), 6.77 – 6.71 (m, 2H), 6.55 (d, J = 1.8 Hz, 1H), 6.21 (d, J = 2.1 Hz, 1H), 4.50 – 4.47 (m, 1H), 4.44 – 4.41 (m, 1H), 3.70 (s, 3H), 2.25 (s, 3H), 1.37 (s, 9H). **^{13}C NMR** (126 MHz, $CDCl_3$): δ 176.7, 171.6, 167.4, 140.7, 137.4, 133.9, 133.6, 133.3, 130.4, 130.0, 129.20, 129.18, 128.9, 128.5, 128.1, 58.5, 52.2, 46.4, 46.1, 28.3, 21.1. **HRMS** (CI+) Calcd for $C_{27}H_{30}NO_4^+$ $[M + H]^+$: 432.2175, Found: 432.2164.

Methyl 2-((*S*)-((*R*)-4-((*E*)-benzylidene)-1-(tert-butyl)-2,5-dioxopyrrolidin-3-yl)(4-

methoxyphenyl)methyl)acrylate (3ec)

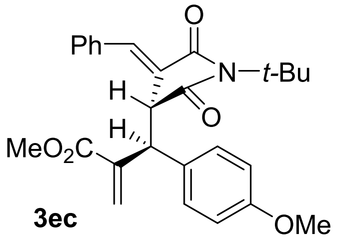

3ec

white solid, 94% yield (21.0 mg, 10:1 dr), mp: 58.8 – 60.1 °C. $[\alpha]_D^{25}$: +287 (c = 1.0, $CHCl_3$). HPLC analysis of the product: Daicel CHIRALPAK IE-3 column; 10% ^{i}PrOH in hexanes; 1.0 mL/min; retention times: 9.515 min (minor), 11.816 min (major). 93% ee. **^{1}H NMR** (500 MHz, $CDCl_3$): δ 7.59 – 7.56 (m, 2H), 7.53 – 7.39 (m, 4H), 6.81 – 6.75 (m, 2H), 6.72 – 6.67 (m, 2H), 6.55 (s, 1H), 6.21 (d, J = 2.0 Hz, 1H), 4.50 – 4.46 (m, 1H), 4.43 – 4.39 (m, 1H), 3.73 (s, 3H), 3.70 (s, 3H), 1.38 (s, 9H). **^{13}C NMR** (101 MHz, $CDCl_3$): δ 176.7, 171.6, 167.4, 159.2, 140.9, 133.9, 133.7, 130.4, 130.09, 130.08, 129.2, 128.6, 128.4, 128.0, 113.9, 58.5, 55.3, 52.3, 46.5, 45.7, 28.4. **HRMS** (CI+) Calcd for $C_{27}H_{30}NO_5^+$ $[M + H]^+$: 448.2124, Found: 448.2114.

Methyl 2-((*R*)-((*S*)-4-((*E*)-benzylidene)-1-(tert-butyl)-2,5-dioxopyrrolidin-3-yl)(4-fluorophenyl)methyl)acrylate (3ed)

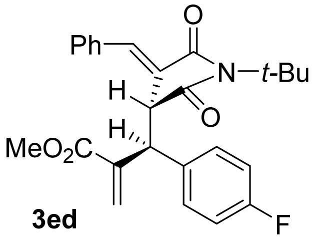

3ed

white solid, 90% yield (19.6 mg, 10:1 dr), mp: 64.7 – 68.1 °C. $[\alpha]_D^{25}$: +246 (c = 1.0, $CHCl_3$). HPLC analysis of the product: Daicel CHIRALPAK IE-3 column; 10% ^{i}PrOH in hexanes; 1.0 mL/min; retention times: 6.348 min (minor), 7.008 min (major). 94% ee. **^{1}H NMR** (400 MHz, $CDCl_3$): δ 7.59 – 7.52 (m, 3H), 7.51 – 7.38 (m, 3H), 6.90 – 6.83 (m, 4H), 6.59 (d, J = 1.1 Hz, 1H), 6.29 (d, J = 1.8 Hz, 1H), 4.48 (t, J = 2.5 Hz, 1H), 4.45 – 4.43 (m, 1H), 3.68 (s, 3H), 1.37 (s, 9H). **^{13}C NMR** (101 MHz, $CDCl_3$): δ 176.4, 171.3, 167.1, 162.4 (d, J = 247.7 Hz), 140.5, 133.9, 133.7, 132.2 (d, J = 3.3 Hz), 130.65, 130.57, 130.4, 130.2, 129.3, 128.3 (d, J = 7.9 Hz), 115.4 (d, J = 21.4 Hz), 58.6, 52.3, 46.3, 45.7, 28.3. **^{19}F NMR** (376 MHz, $CDCl_3$): δ -114.7. **HRMS** (CI+) Calcd for $C_{26}H_{27}FNO_4^+$ $[M + H]^+$: 436.1924, Found: 436.1914.

Methyl 2-((*S*)-((*R*)-4-((*E*)-benzylidene)-1-(tert-butyl)-2,5-dioxopyrrolidin-3-yl)(4-chlorophenyl)methyl)acrylate (3ee)

white solid, 92% yield (20.7 mg, 11:1 dr), mp: 72.7 – 73.8 °C. $[\alpha]_D^{25}$: +287 (c = 1.0, $CHCl_3$). HPLC analysis of the product: Daicel CHIRALPAK IE-3 column; 10%

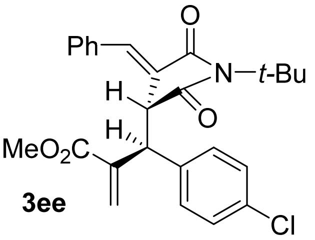

3ee

^{i}PrOH in hexanes; 1.0 mL/min; retention times: 6.412 min (minor), 7.338 min (major). 93% ee. **^{1}H NMR** (500 MHz, $CDCl_3$): *δ* 7.56 – 7.53 (m, 3H), 7.50 – 7.41 (m, 4H), 7.16 (d, *J* = 8.4 Hz, 2H), 6.81 (d, *J* = 8.4 Hz, 2H), 6.60 (s, 1H), 6.26 (d, *J* = 1.7 Hz, 1H), 4.51 – 4.47 (m, 1H), 4.45 – 4.40 (m, 1H), 3.69 (s, 3H), 1.38 (s, 9H). **^{13}C NMR** (126 MHz, $CDCl_3$): *δ* 176.2, 171.2, 167.0, 140.1, 135.0, 133.9, 133.7, 133.6, 130.3, 130.2, 130.1, 130.0, 129.2, 128.6, 128.0, 58.6, 52.2, 46.0, 45.7, 28.2. **HRMS** (CI+) Calcd for $C_{26}H_{27}ClNO_4^+$ $[M + H]^+$: 452.1629, Found: 452.1620.

Methyl 2-((*S*)-((*R*)-4-((*E*)-benzylidene)-1-(tert-butyl)-2,5-dioxopyrrolidin-3-yl)(naphthalen-2-yl)methyl)acrylate (3ef)

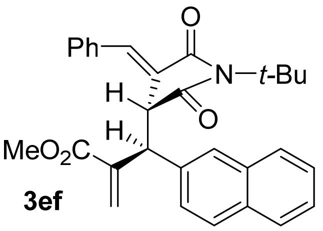

3ef

white solid, 91% yield (21.0 mg, 14:1 dr), mp: 106.3 – 108.1 °C. $[\alpha]_D^{25}$: +424 (c = 1.0, $CHCl_3$). HPLC analysis of the product: Daicel CHIRALPAK IE-3 column; 10% ^{i}PrOH in hexanes; 1.0 mL/min; retention times: 8.056 min (minor), 9.841 min (major). 95% ee. **^{1}H NMR** (400 MHz, $CDCl_3$): *δ* 7.78 – 7.72 (m, 1H), 7.71 – 7.33 (m, 11H), 6.99 (dd, *J* = 8.5, 1.8 Hz, 1H), 6.63 – 6.56 (m, 1H), 6.21 (d, *J* = 1.8 Hz, 1H), 4.65 – 4.63 (m, 1H), 4.62 – 4,61(m, 1H), 3.71 (s, 3H), 1.27 (s, 9H). **^{13}C NMR** (101 MHz, $CDCl_3$): *δ* 176.7, 171.3, 167.4, 140.7, 134.0, 133.9, 133.3, 132.9, 130.4, 130.1, 129.3,128.9, 128.4, 128.3, 128.2, 128.0, 127.9, 127.7, 127.0, 126.3, 126.1, 58.5, 52.3, 46.5, 46.3, 28.2. **HRMS** (CI+) Calcd for $C_{30}H_{30}NO_4^+$ $[M + H]^+$: 468.2175, Found: 468.2166.

Methyl 2-((*R*)-((*R*)-4-((*E*)-benzylidene)-1-(tert-butyl)-2,5-dioxopyrrolidin-3-yl)(thiophen-2-yl)methyl)acrylate (3eg)

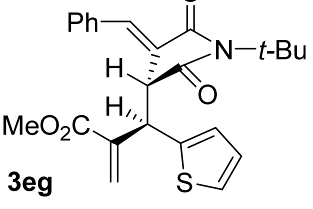

3eg

white solid, 91% yield (19.2 mg, >20:1 dr), mp: 76.2 – 79.3 °C. $[\alpha]_D^{25}$: +190 (c = 1.0, $CHCl_3$). HPLC analysis of the product: Daicel CHIRALPAK IE-3 column; 5% ^{i}PrOH in hexanes; 1.0 mL/min; retention times: 12.029 min (minor), 13.145 min (major). 92% ee. **^{1}H NMR** (500 MHz, $CDCl_3$): *δ* 7.61 – 7.54 (m, 3H), 7.50 – 7.43 (m, 2H), 7.45 – 7.39 (m, 1H), 7.10 (dd, *J* = 5.2, 1.2 Hz, 1H), 6.86 –

6.82 (m, 1H), 6.56 – 6.51 (m, 2H), 6.20 (d, J = 1.7 Hz, 1H), 4.78 – 4.72 (m, 1H), 4.54 – 4.46 (m, 1H), 3.75 (s, 3H), 1.48 (s, 9H). **^{13}C NMR** (126 MHz, $CDCl_3$): δ 176.3, 171.5, 167.2, 140.3, 138.5, 134.3, 133.8, 130.3, 129.7, 129.2, 128.5, 128.0, 126.9, 124.9, 58.7, 52.4, 46.4, 41.1, 28.4. **HRMS** (CI+) Calcd for $C_{24}H_{26}NO_4S^+$ $[M + H]^+$: 424.1583, Found: 424.1576.

Ethyl 2-((*S*)-((*R*)-4-((*E*)-benzylidene)-1-(tert-butyl)-2,5-dioxopyrrolidin-3-yl)(phenyl)methyl)acrylate (3eh)

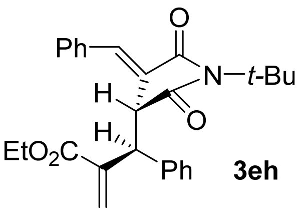

3eh

white solid, 88% yield (19.0 mg, 7:1 dr), mp: 63.2 – 64.1 °C. $[\alpha]_D^{25}$: +247 (c = 1.0, $CHCl_3$). HPLC analysis of the product: Daicel CHIRALPAK IE-3 column; 10% ^{i}PrOH in hexanes; 1.0 mL/min; retention times: 6.883 min (minor), 7.641 min (major). 91% ee. **^{1}H NMR** (500 MHz, $CDCl_3$): δ 7.60 – 7.52 (m, 3H), 7.49 – 7.36 (m, 4H), 7.20 – 7.14 (m, 3H), 6.89 (dd, J = 7.1, 2.5 Hz, 2H), 6.61 (s, 1H), 6.31 (d, J = 1.7 Hz, 1H), 4.48 (t, J = 2.4 Hz, 1H), 4.45 (d, J = 2.7 Hz, 1H), 4.15 – 4.07 (m, 2H), 1.34 (s, 9H), 1.14 (t, J = 7.1 Hz, 3H). **^{13}C NMR** (126 MHz, $CDCl_3$): δ 176.5, 171.4, 166.8, 140.9, 136.6, 133.9, 133.6, 130.4, 130.1, 129.2, 129.0, 128.6, 128.5, 128.0, 127.7, 61.0, 58.5, 46.5, 46.4, 28.3, 14.1. **HRMS** (CI+) Calcd for $C_{27}H_{30}NO_4^+$ $[M + H]^+$: 432.2175, Found: 432.2165.

Benzyl 2-((*S*)-((*R*)-4-((*E*)-benzylidene)-1-(tert-butyl)-2,5-dioxopyrrolidin-3-yl)(phenyl)methyl)acrylate (3ei)

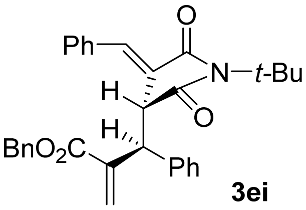

3ei

white solid, 94% yield (23.2 mg, 15:1 dr), mp: 65.5 – 68.7 °C. $[\alpha]_D^{25}$: +232 (c = 1.0, $CHCl_3$). HPLC analysis of the product: Daicel CHIRALPAK IE-3 column; 10% ^{i}PrOH in hexanes; 1.0 mL/min; retention times: 8.376 min (minor), 8.871 min (major). 92% ee. **^{1}H NMR** (500 MHz, $CDCl_3$): δ 7.56 – 7.50 (m, 3H), 7.43 – 7.37 (m, 3H), 7.29 – 7.26 (m, 3H), 7.20 – 7.15 (m, 3H), 7.14 – 7.09 (m, 2H), 6.92 – 6.87 (m, 2H), 6.69 (s, 1H), 6.43 (d, J = 1.7 Hz, 1H), 5.14 – 5.03 (m, 2H), 4.49 – 4.42 (m, 2H), 1.32 (s, 9H). **^{13}C NMR** (126 MHz, $CDCl_3$): δ 176.4, 171.4, 166.5, 140.6, 136.4, 135.9, 133.7, 133.6, 130.4, 130.1, 129.3, 129.0, 128.8, 128.7, 128.55, 128.53, 128.1, 127.9, 127.8, 66.7, 58.5, 46.6, 46.5, 28.2. **HRMS** (CI+) Calcd

for $C_{32}H_{32}NO_4^+$ [M + H]$^+$: 494.2331, Found: 494.2321.

Butyl 2-((*S*)-((*R*)-4-((*E*)-benzylidene)-1-(tert-butyl)-2,5-dioxopyrrolidin-3-yl)(phenyl)methyl)acrylate (3ej)

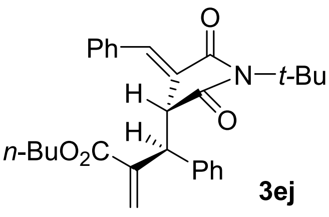

white solid, 91% yield (20.9 mg, 7:1 dr), mp: 52.0 – 54.4 °C. [α]$_D^{25}$: +280 (c = 1.0, $CHCl_3$). HPLC analysis of the product: Daicel CHIRALPAK IE-3 column; 5% ^{i}PrOH in hexanes; 1.0 mL/min; retention times: 9.202 min (minor), 10.105 min (major). 93% ee. **^{1}H NMR** (500 MHz, $CDCl_3$): *δ* 7.58 – 7.53 (m, 3H), 7.48 – 7.40 (m, 3H), 7.21 – 7.14 (m, 3H), 6.92 – 6.87 (m, 2H), 6.63 (s, 1H), 6.42 – 6.37 (m, 1H), 4.47 – 4.41 (m, 2H), 4.06 – 3.99 (m, 2H), 1.48 – 1.42 (m, 2H), 1.32 (s, 9H), 1.21 – 1.12 (m, 2H), 0.81 (t, *J* = 7.4 Hz, 3H). **^{13}C NMR** (126 MHz, $CDCl_3$): *δ* 176.5, 171.4, 166.8, 140.9, 136.6, 133.8, 133.6, 130.4, 130.1, 129.3, 129.0, 128.7, 128.5, 128.1, 127.7, 64.9, 58.5, 46.7, 46.5, 30.6, 28.3, 19.1, 13.8. **HRMS** (CI+) Calcd for $C_{29}H_{34}NO_4^+$ [M + H]$^+$: 460.2488, Found: 460.2480.

Isobutyl 2-((*S*)-((*R*)-4-((*E*)-benzylidene)-1-(tert-butyl)-2,5-dioxopyrrolidin-3-yl)(phenyl)methyl)acrylate (3ek)

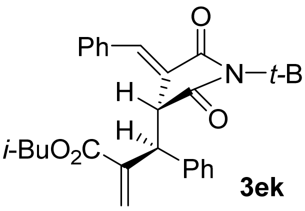

white solid, 91% yield (20.9 mg, 8:1 dr), mp: 53.1 – 55.5 °C. [α]$_D^{25}$: +330 (c = 1.0, $CHCl_3$). HPLC analysis of the product: Daicel CHIRALPAK IE-3 column; 5% ^{i}PrOH in hexanes; 1.0 mL/min; retention times: 8.803 min (minor), 9.844 min (major). 92% ee. **^{1}H NMR** (500 MHz, $CDCl_3$): *δ* 7.59 – 7.52 (m, 3H), 7.49 – 7.37 (m, 3H), 7.21 – 7.12 (m, 3H), 6.95 – 6.88 (m, 2H), 6.66 (d, *J* = 1.0 Hz, 1H), 6.44 (d, *J* = 1.2 Hz, 1H), 4.44 (d, *J* = 1.7 Hz, 2H), 3.84 – 3.71 (m, 2H), 1.76 (hept, *J* = 13.3, 6.7 Hz, 1H), 1.30 (s, 9H), 0.73 (d, *J* = 6.7 Hz, 6H). **^{13}C NMR** (126 MHz, $CDCl_3$): *δ* 176.4, 171.4, 166.7, 140.9, 136.6, 133.8, 133.6, 130.4, 130.1, 129.3, 129.0, 128.9, 128.8, 128.5, 128.2, 127.7, 71.1, 58.5, 46.7, 28.2, 27.7, 19.0. **HRMS** (CI+) Calcd for $C_{29}H_{34}NO_4^+$ [M + H]$^+$: 460.2488, Found: 460.2481.

IV. Scale-up of the allylic alkylation

Scale -up reaction:

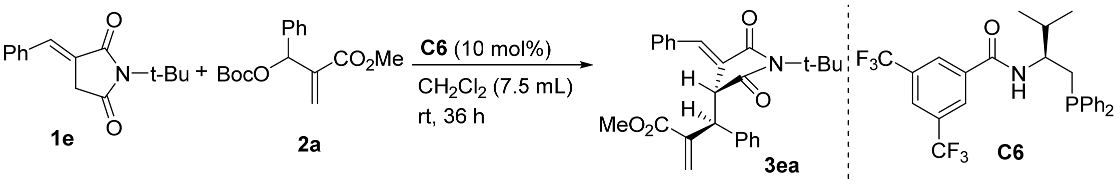

To a solution of DCM (7.5 mL) were added *(E)*-3-benzylidene-1-(tert-butyl)pyrrolidine-2,5-dione **1e** (122mg, 1equiv, 0.5 mmol), MBH carbonate **2a** (175mg, 1.2 equiv, 0.6 mmol) and catalyst **C6** (25mg, 10 mol%, 0.05 mmol). The reaction mixture was stirred at r.t. for 36h. Then the mixture was purified by silica gel column chromatography (eluent: PE/EA = 2:1) to yield the desired product **3ea** in 92% yield (196 mg, 94% ee, dr: 14:1).

V. Determination of the Stereochemistry

The absolute stereochemistry of products **3la** was determined by X-ray diffraction (CCDC 2244711 for **3la**).The stereochemistry of other products was assumed by analogy.

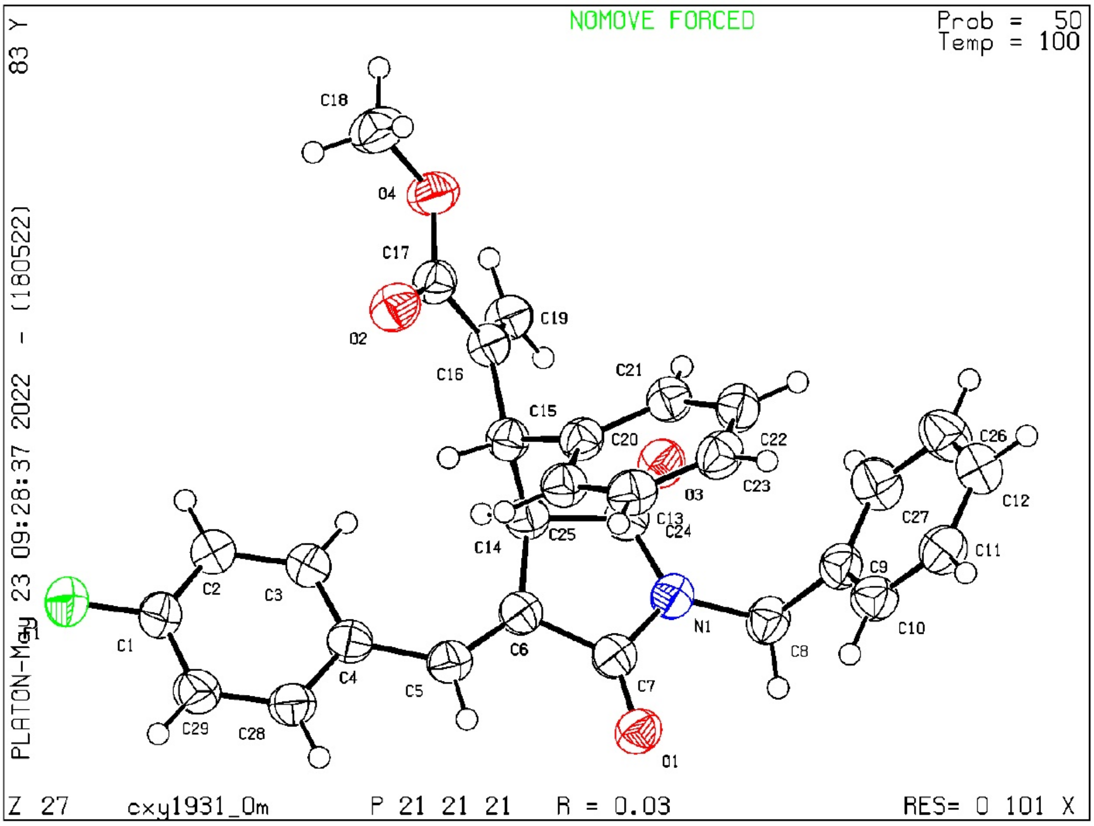

Table S1. Crystal data and structure refinement for 3la.

Identification code	cxy1931_0m
Empirical formula	$C_{29}H_{24}FNO_4$
Formula weight	469.49
Temperature/K	100(2)
Crystal system	orthorhombic
Space group	$P2_12_12_1$
a/Å	9.0198(3)
b/Å	13.0367(4)
c/Å	20.2824(6)
α/°	90
β/°	90
γ/°	90
Volume/Å^3	2384.98(13)
Z	4
ρ_{calc}g/cm^3	1.308
μ/mm^{-1}	0.756
F(000)	984.0
Crystal size/mm^3	0.36 × 0.33 × 0.29
Radiation	CuKα (λ = 1.54178)
2Θ range for data collection/°	8.062 to 148.94
Index ranges	-11 ≤ h ≤ 10, -15 ≤ k ≤ 16, -22 ≤ l ≤ 24
Reflections collected	22241
Independent reflections	4844 [R_{int} = 0.0403, R_{sigma} = 0.0250]
Data/restraints/parameters	4844/0/318
Goodness-of-fit on F^2	1.056
Final R indexes [I>=2σ (I)]	R_1 = 0.0277, wR_2 = 0.0725
Final R indexes [all data]	R_1 = 0.0292, wR_2 = 0.0737
Largest diff. peak/hole / e Å^{-3}	0.14/-0.13
Flack parameter	0.01(5)

VI. Copies of NMR of substrates

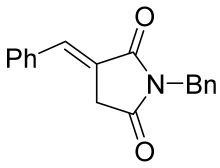

1a

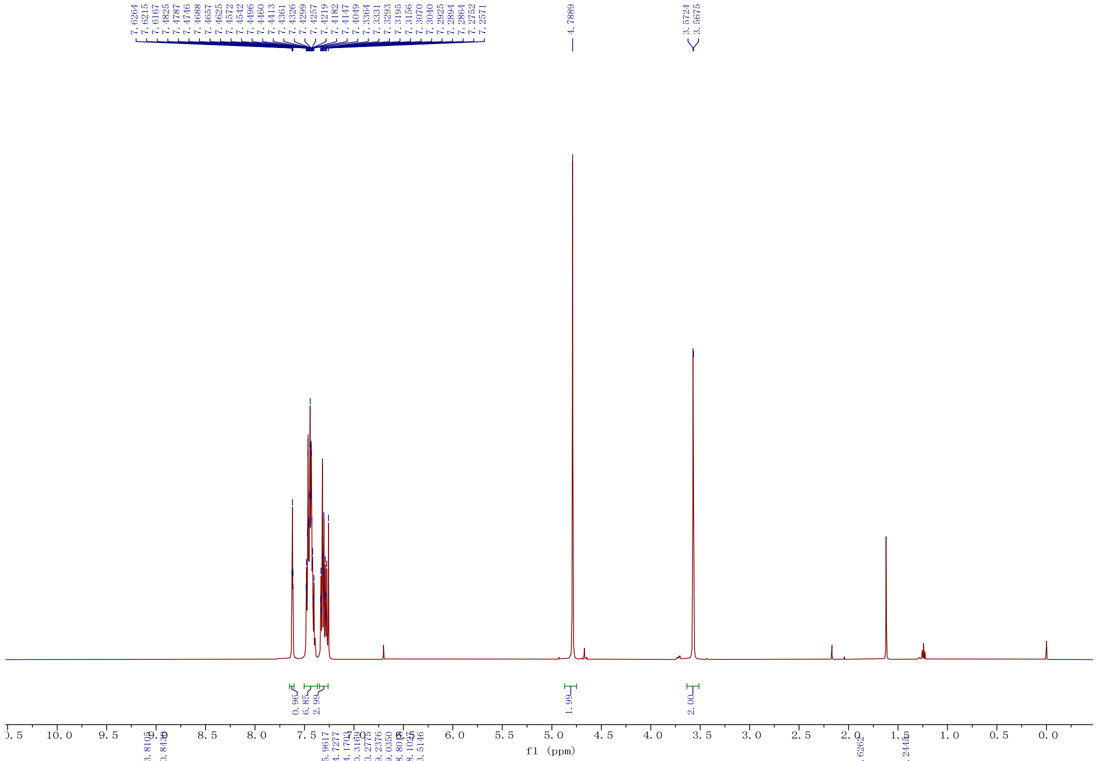

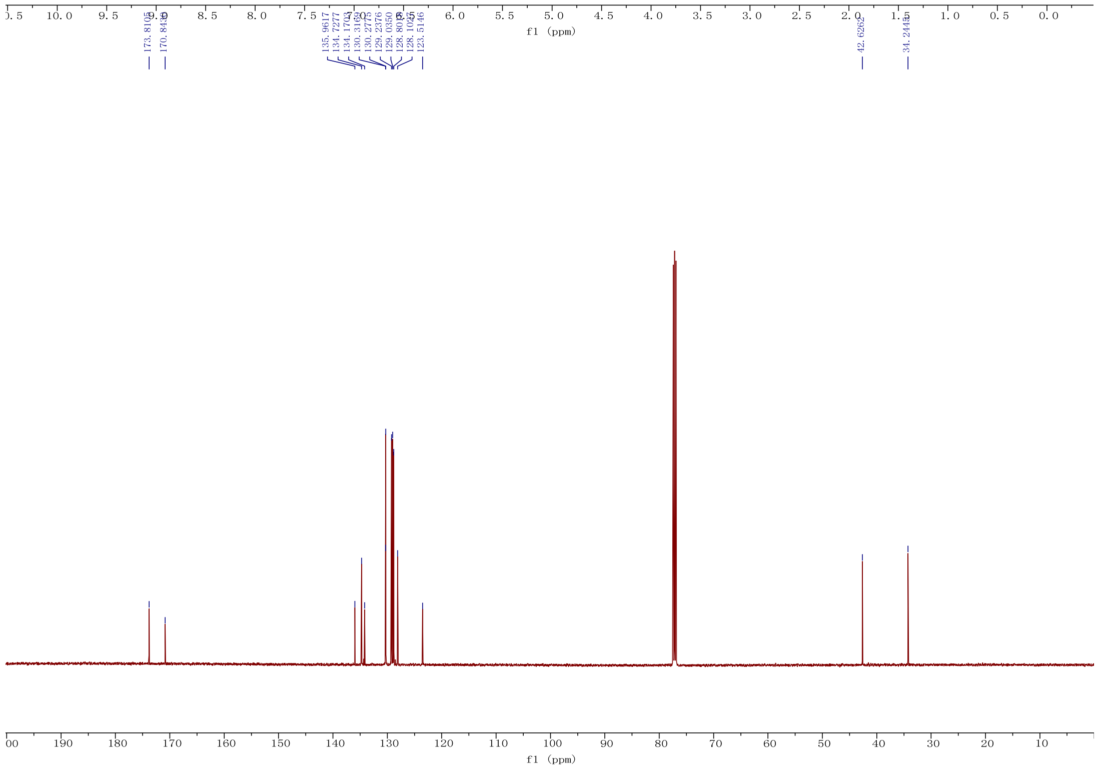

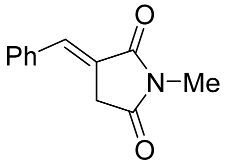
Ph
O
N−Me
O

1b

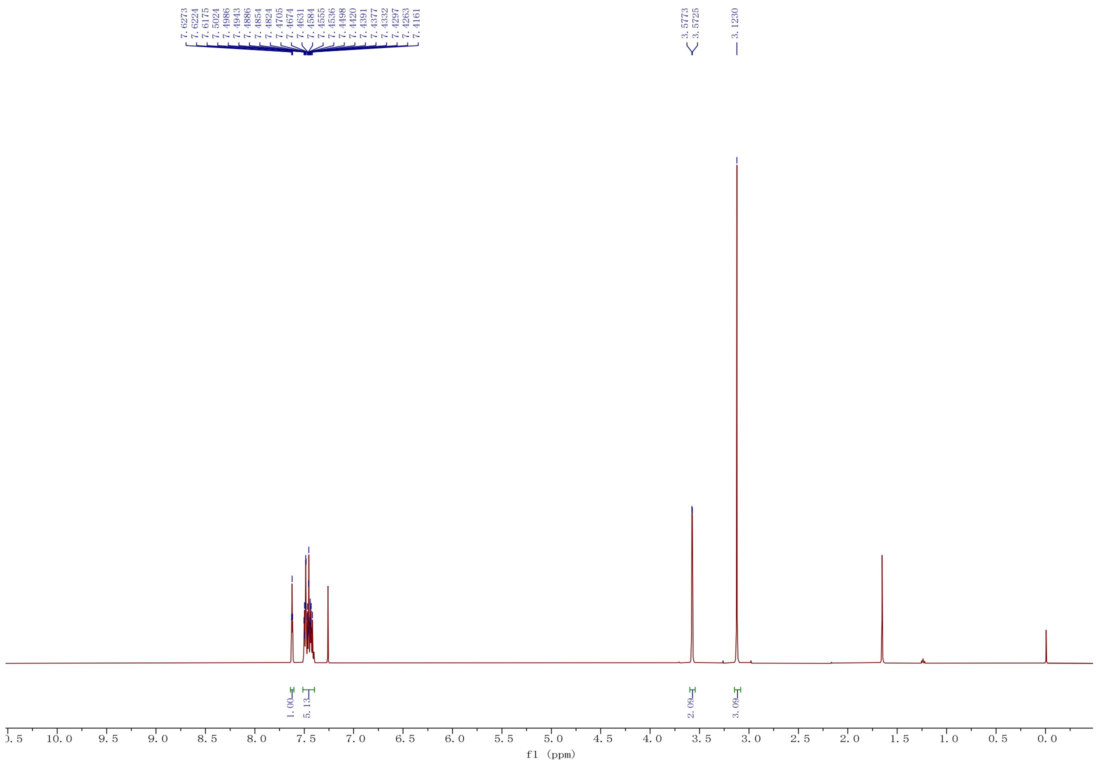
7.6273
7.6224
7.6175
7.5024
7.4986
7.4943
7.4886
7.4854
7.4824
7.4705
7.4674
7.4631
7.4584
7.4555
7.4536
7.4498
7.4420
7.4391
7.4377
7.4332
7.4297
7.4263
7.4161
3.5773
3.5725
3.1230
1.00
5.13
2.09
3.09
f1 (ppm)

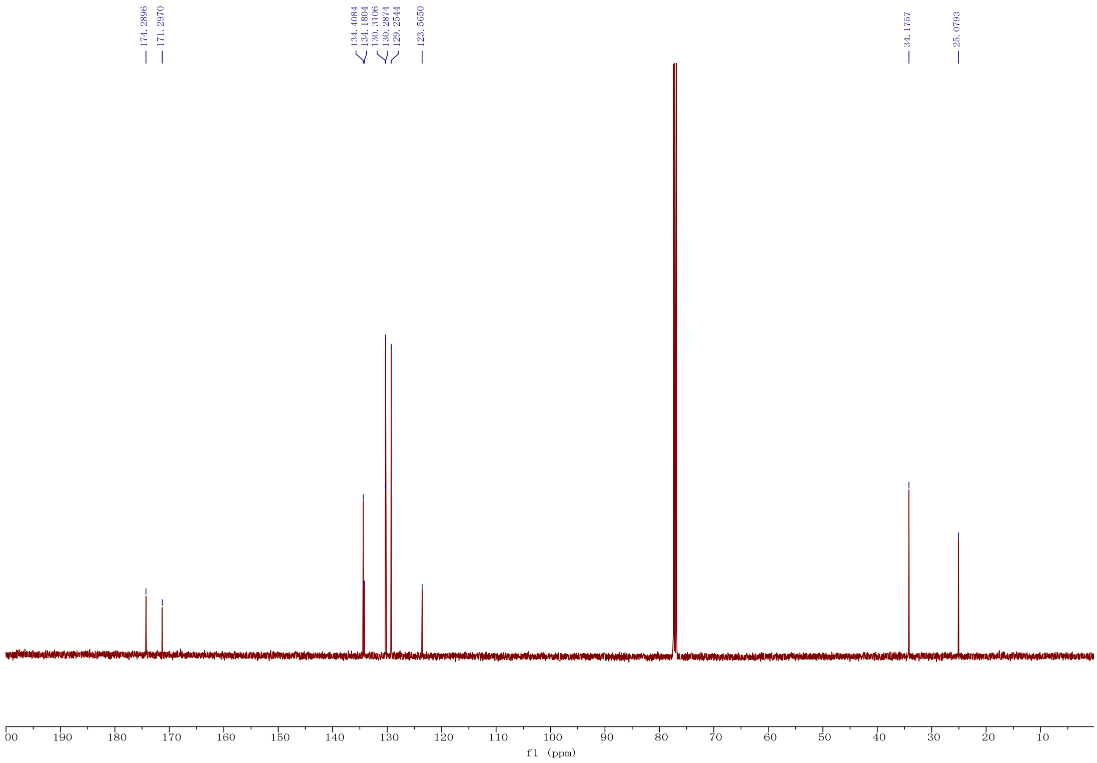
174.2896
171.2970
134.4084
134.1804
130.3106
130.2874
129.2544
123.5650
34.1757
25.0793
f1 (ppm)

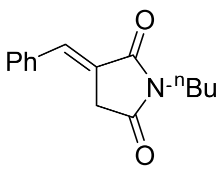
Ph
O
N-nBu
O

1c

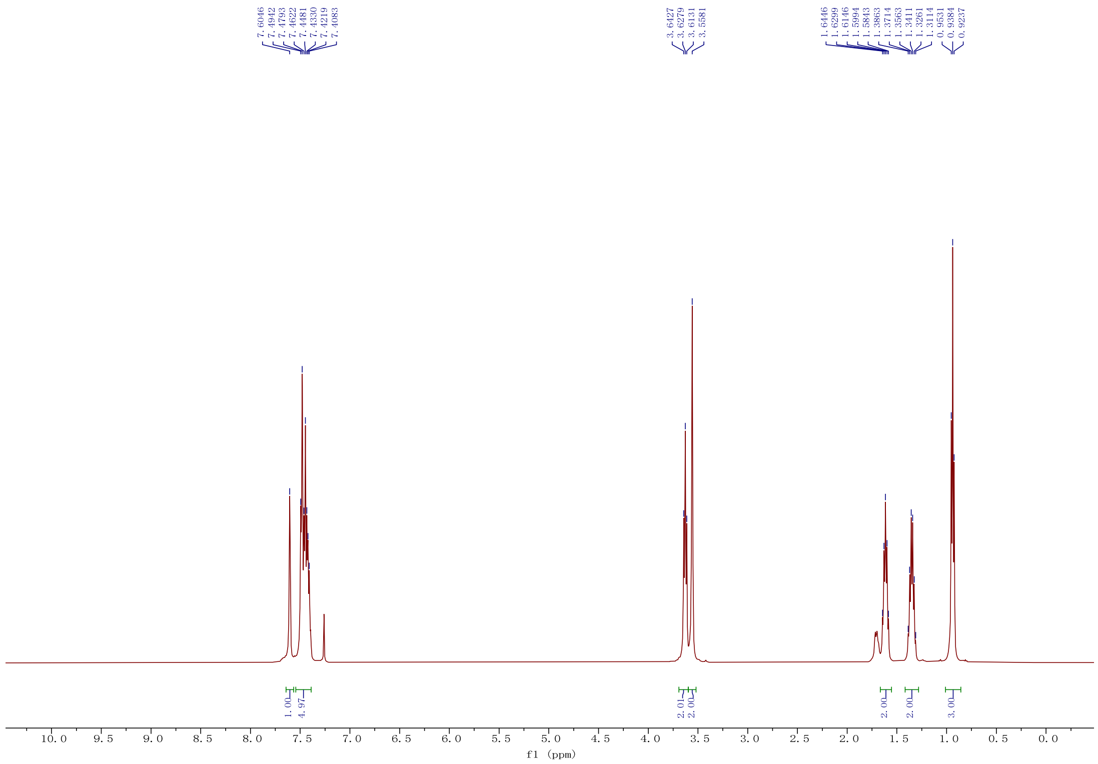
7.6046
7.4942
7.4793
7.4622
7.4481
7.4330
7.4219
7.4083
3.6427
3.6279
3.6131
3.5581
1.6446
1.6299
1.6146
1.5994
1.5843
1.3863
1.3714
1.3563
1.3411
1.3261
1.3114
0.9531
0.9384
0.9237
1.00
4.97
2.01
2.00
2.00
2.00
3.00
10.0 9.5 9.0 8.5 8.0 7.5 7.0 6.5 6.0 5.5 5.0 4.5 4.0 3.5 3.0 2.5 2.0 1.5 1.0 0.5 0.0
f1 (ppm)

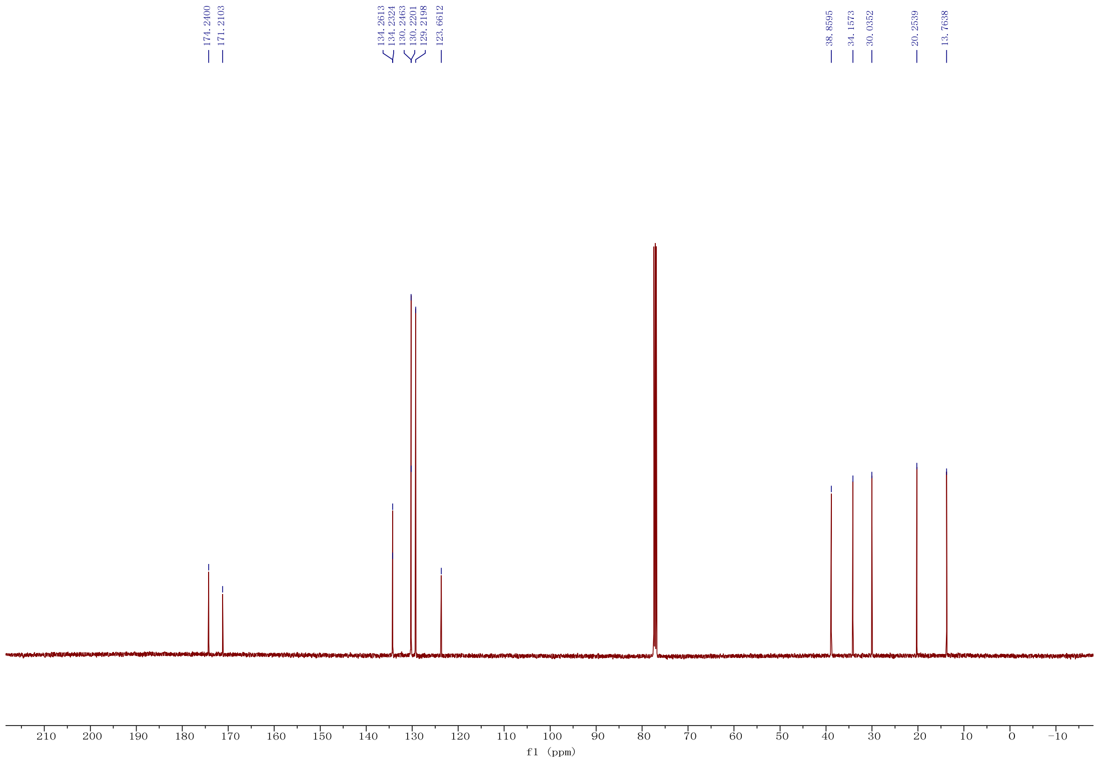
174.2400
171.2103
134.2613
134.2324
130.2463
130.2201
129.2198
123.6612
38.8595
34.1573
30.0352
20.2539
13.7638
210 200 190 180 170 160 150 140 130 120 110 100 90 80 70 60 50 40 30 20 10 0 -10
f1 (ppm)

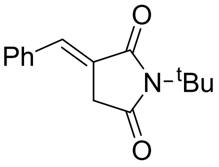
Ph
O
N
tBu
O

1d

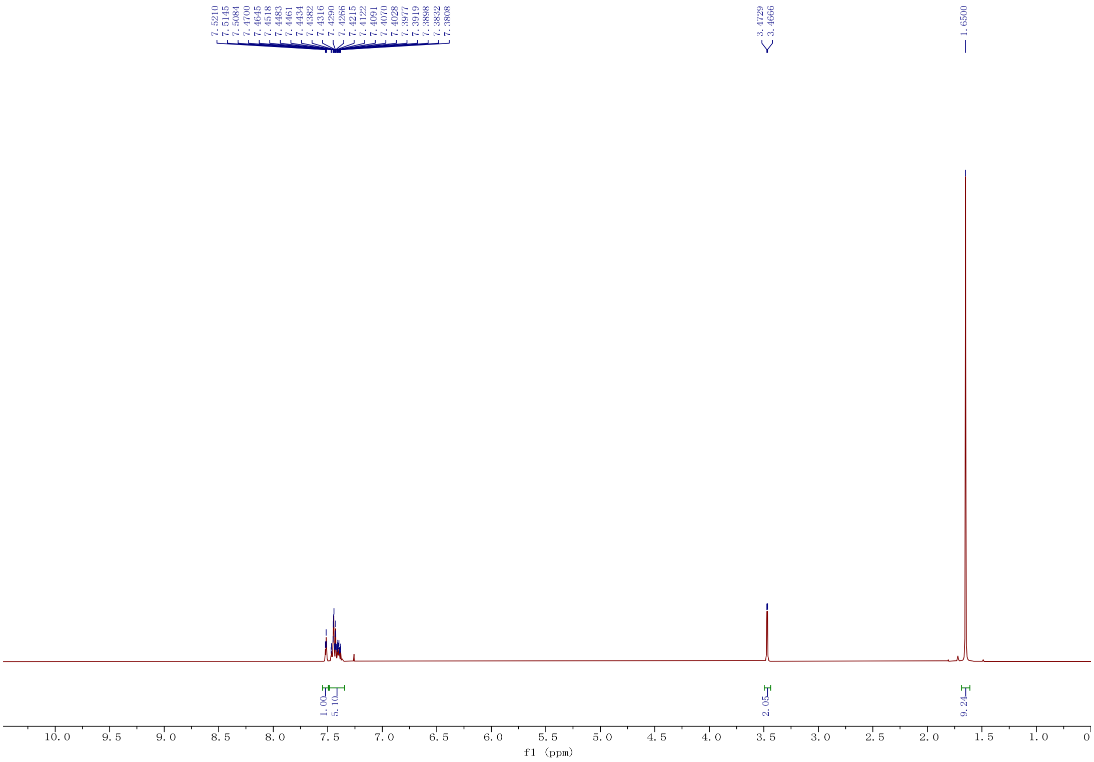
7.5210
7.5145
7.5084
7.4700
7.4645
7.4518
7.4483
7.4461
7.4434
7.4382
7.4316
7.4290
7.4266
7.4215
7.4122
7.4091
7.4070
7.4028
7.3977
7.3919
7.3898
7.3832
7.3808
3.4729
3.4666
1.6500
1.00
5.10
2.05
9.24
f1 (ppm)

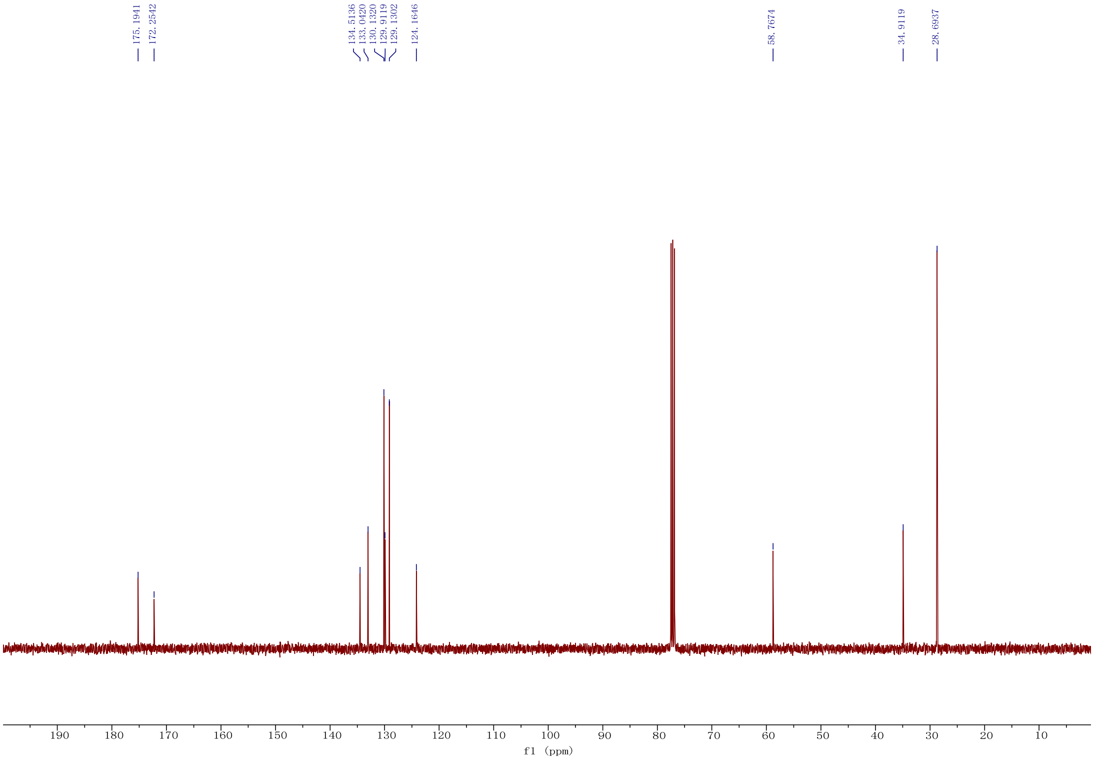
175.1941
172.2542
134.5136
133.0420
130.1320
129.9119
129.1302
124.1646
58.7674
34.9119
28.6937
f1 (ppm)

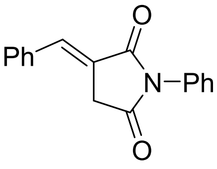
Ph
N−Ph
O
O

1e

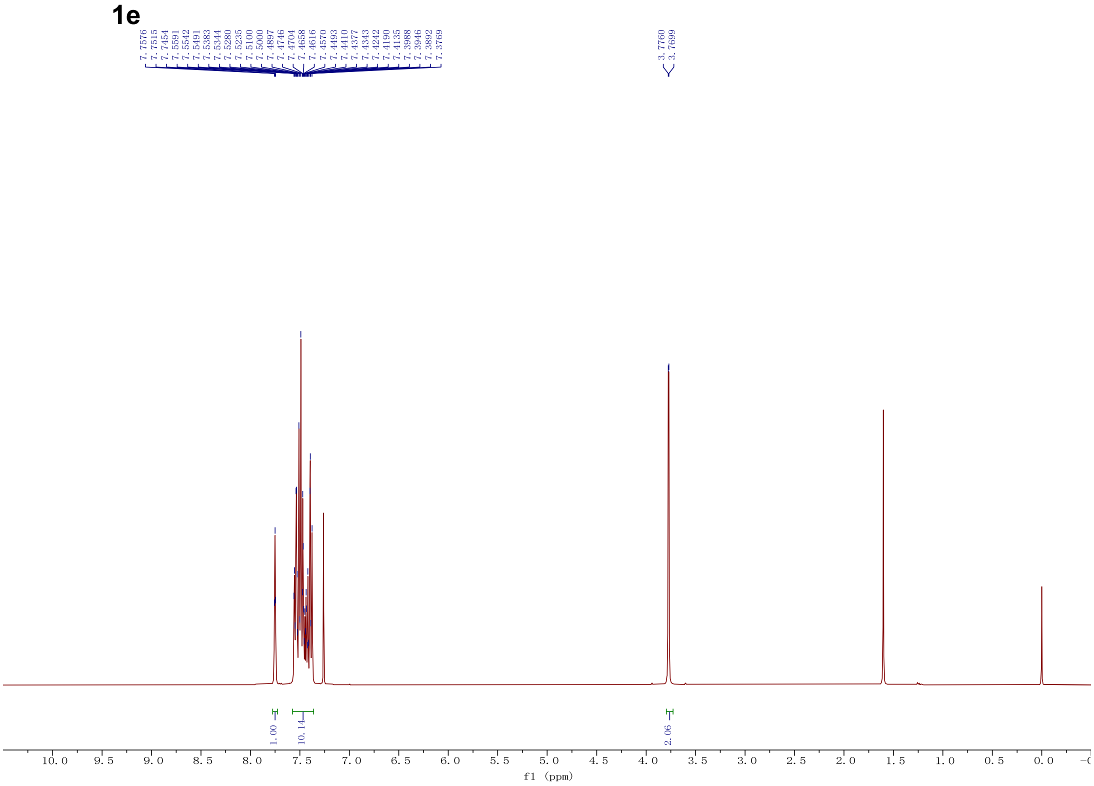
7.7576
7.7515
7.7454
7.5591
7.5542
7.5491
7.5383
7.5344
7.5280
7.5235
7.5100
7.5000
7.4897
7.4746
7.4704
7.4658
7.4616
7.4570
7.4493
7.4410
7.4377
7.4313
7.4242
7.4190
7.4135
7.3988
7.3946
7.3892
7.3769
3.7760
3.7699
1.00
10.14
2.06
10.0 9.5 9.0 8.5 8.0 7.5 7.0 6.5 6.0 5.5 5.0 4.5 4.0 3.5 3.0 2.5 2.0 1.5 1.0 0.5 0.0
f1 (ppm)

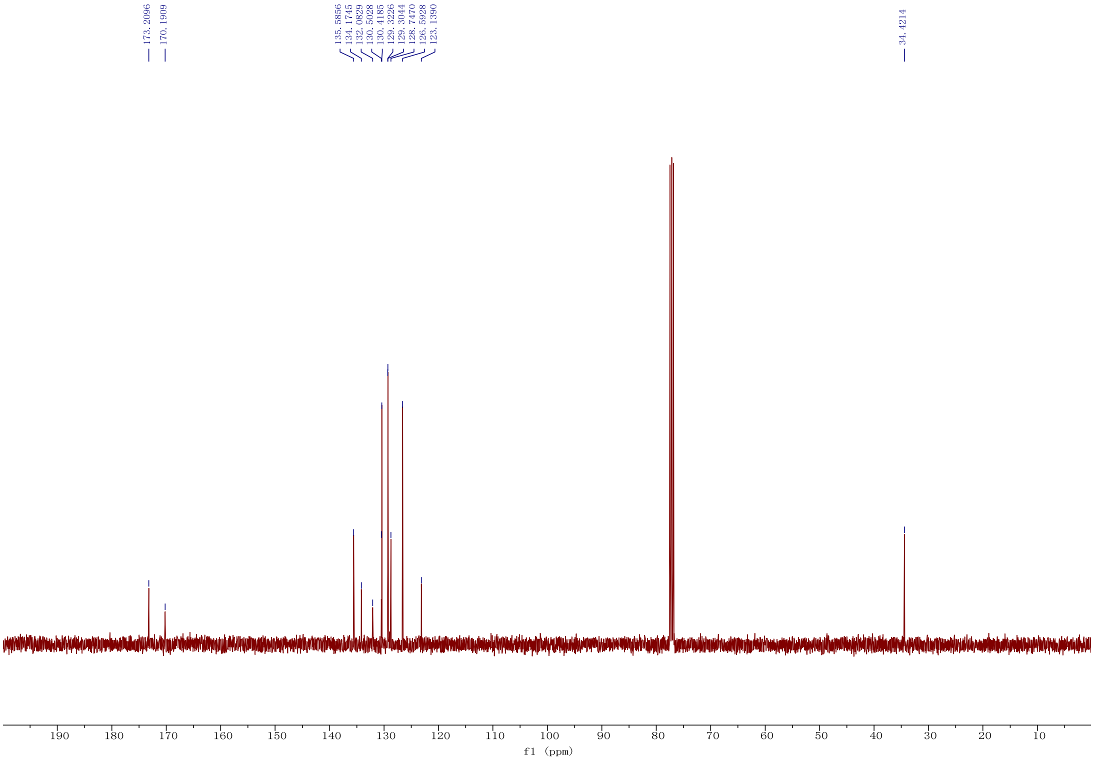
173.2096
170.1909
135.5856
134.1745
132.0829
130.5028
130.4185
129.3226
129.3044
128.7470
126.5928
123.1390
34.4214
190 180 170 160 150 140 130 120 110 100 90 80 70 60 50 40 30 20 10
f1 (ppm)

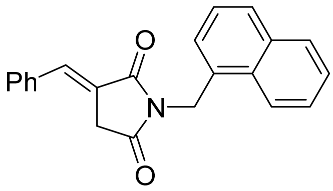

1f

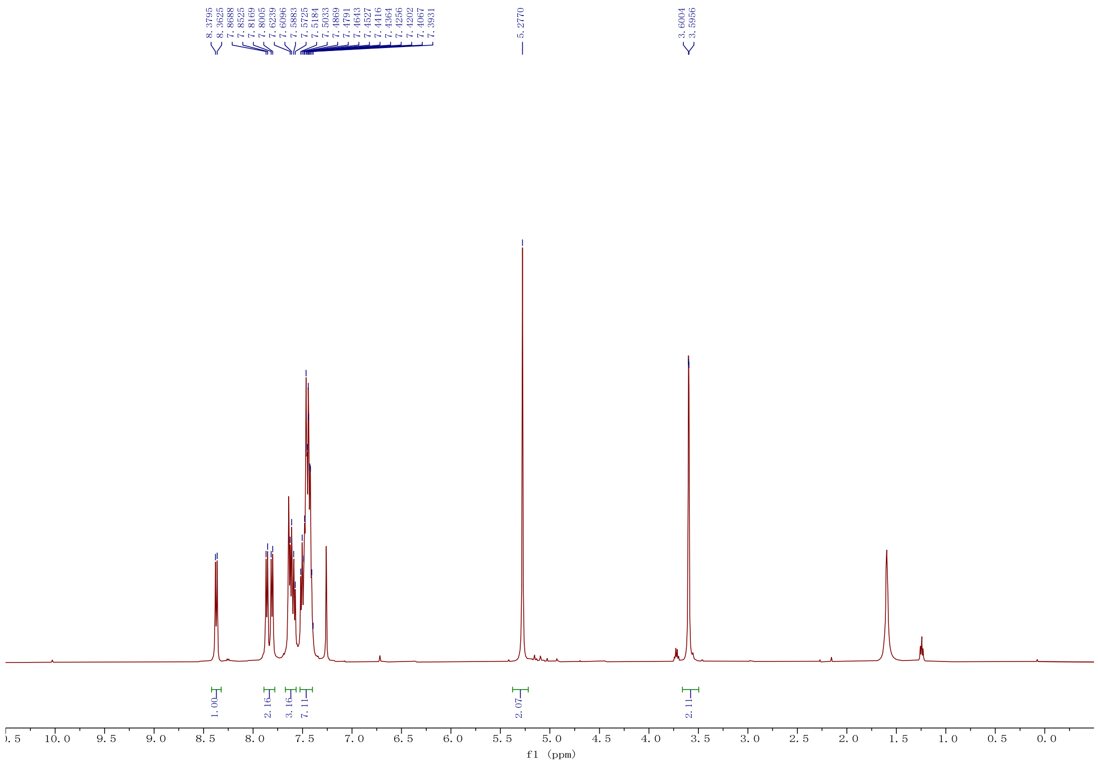

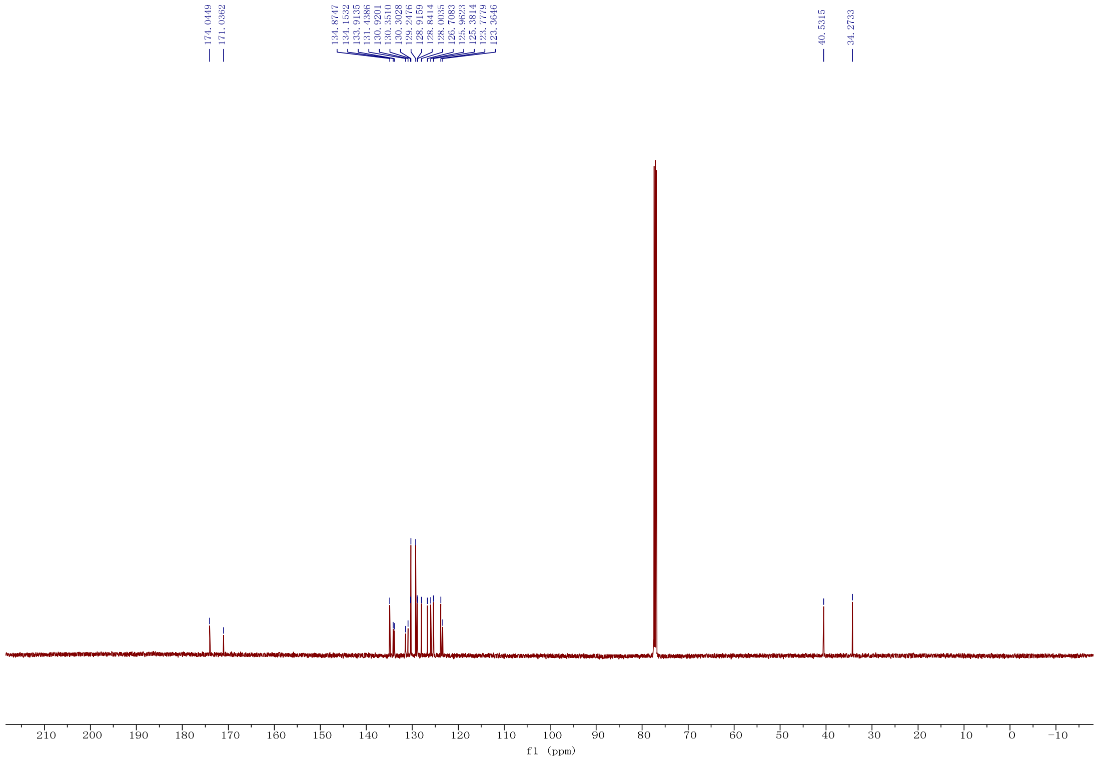

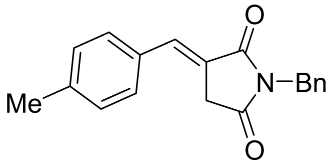
O
N−Bn
O
Me

1g

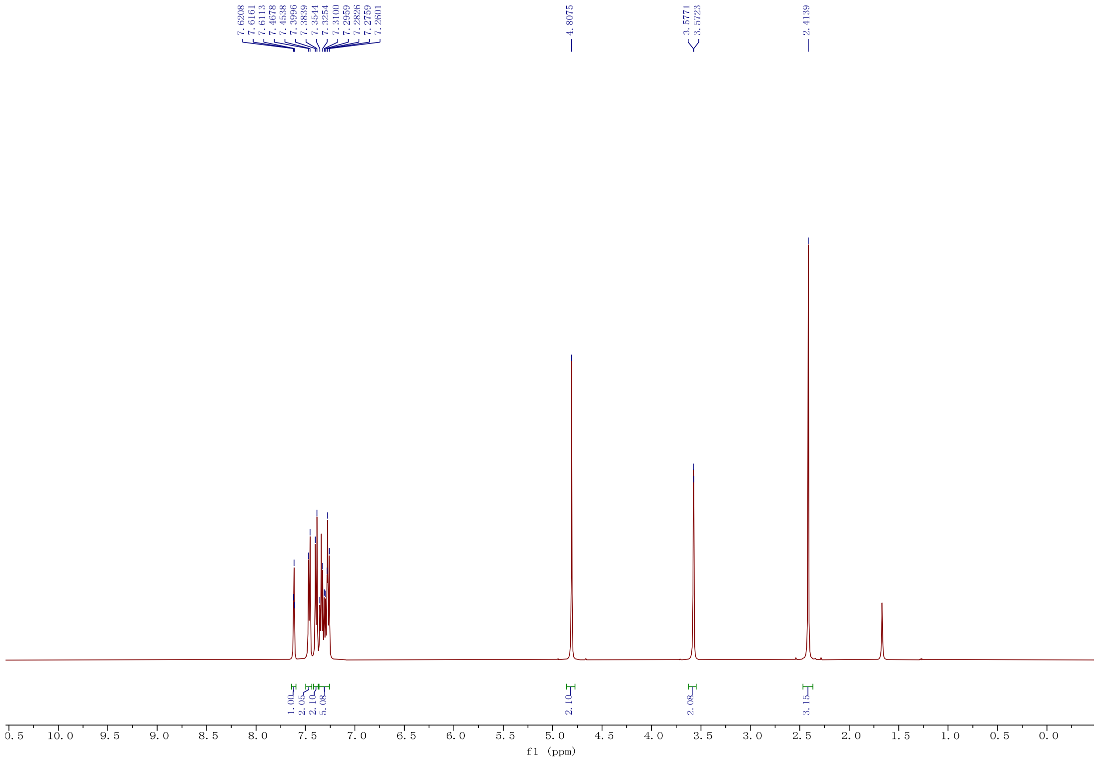
7.6208
7.6161
7.6113
7.4678
7.4538
7.3996
7.3839
7.3544
7.3254
7.3100
7.2959
7.2826
7.2759
7.2601
4.8075
3.5771
3.5723
2.4139
1.00
2.05
2.10
5.08
2.10
2.08
3.15
f1 (ppm)

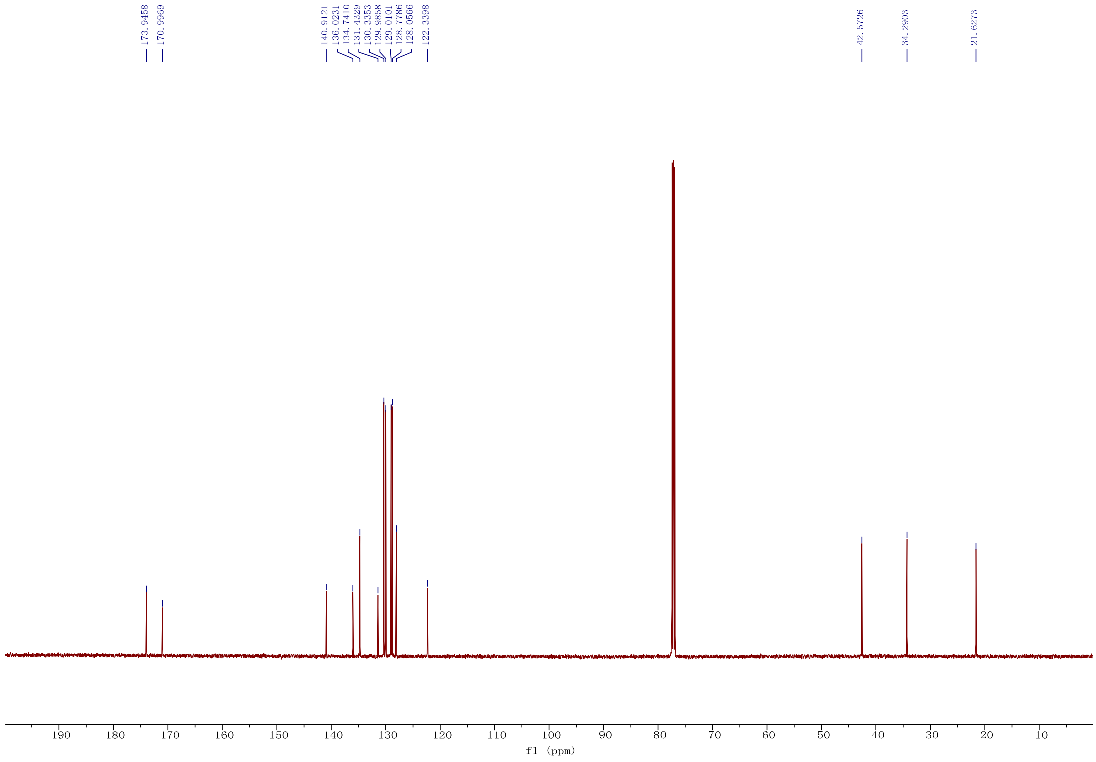
173.9458
170.9969
140.9121
136.0231
134.7410
131.4329
130.3353
129.9858
129.0101
128.7786
128.0566
122.3398
42.5726
34.2903
21.6273
f1 (ppm)

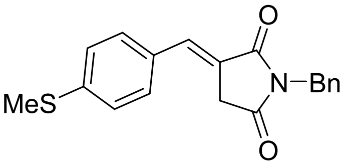
O
MeS
N−Bn
O

1h

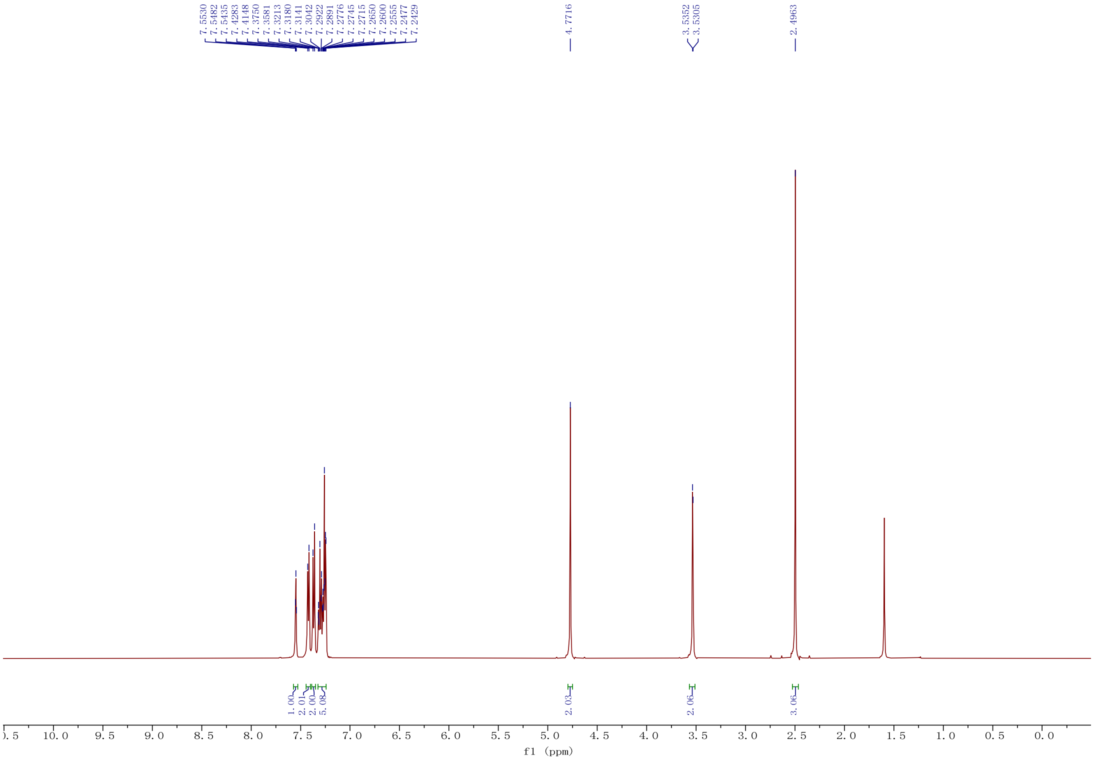
7.5530
7.5482
7.5435
7.4283
7.4148
7.3750
7.3581
7.3213
7.3180
7.3141
7.3042
7.2922
7.2891
7.2776
7.2745
7.2715
7.2650
7.2600
7.2555
7.2477
7.2429
4.7716
3.5352
3.5305
2.4963
1.00
2.01
2.00
5.08
2.03
2.06
3.06
f1 (ppm)

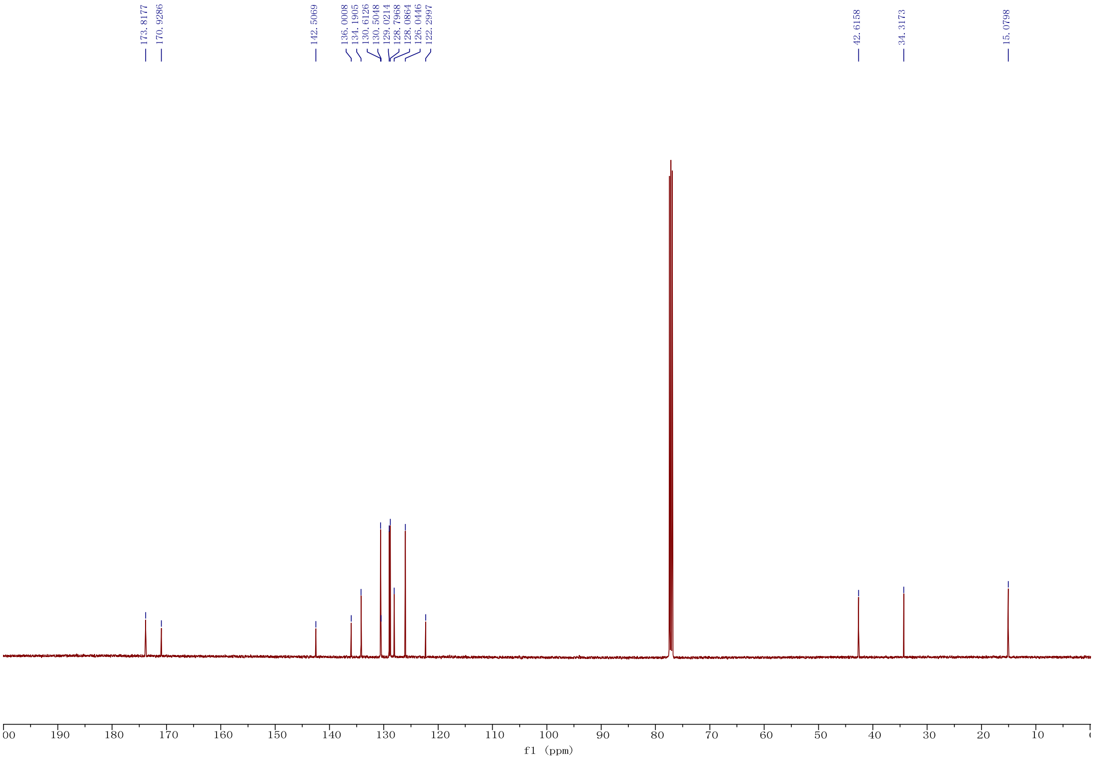
173.8177
170.9286
142.5069
136.0008
134.1905
130.6126
130.5048
129.0214
128.7968
128.0864
126.0446
122.2997
42.6158
34.3173
15.0798
f1 (ppm)

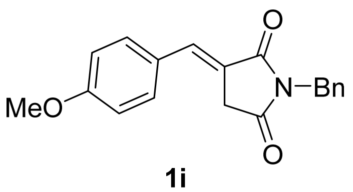
MeO
O
N−Bn
O

1i

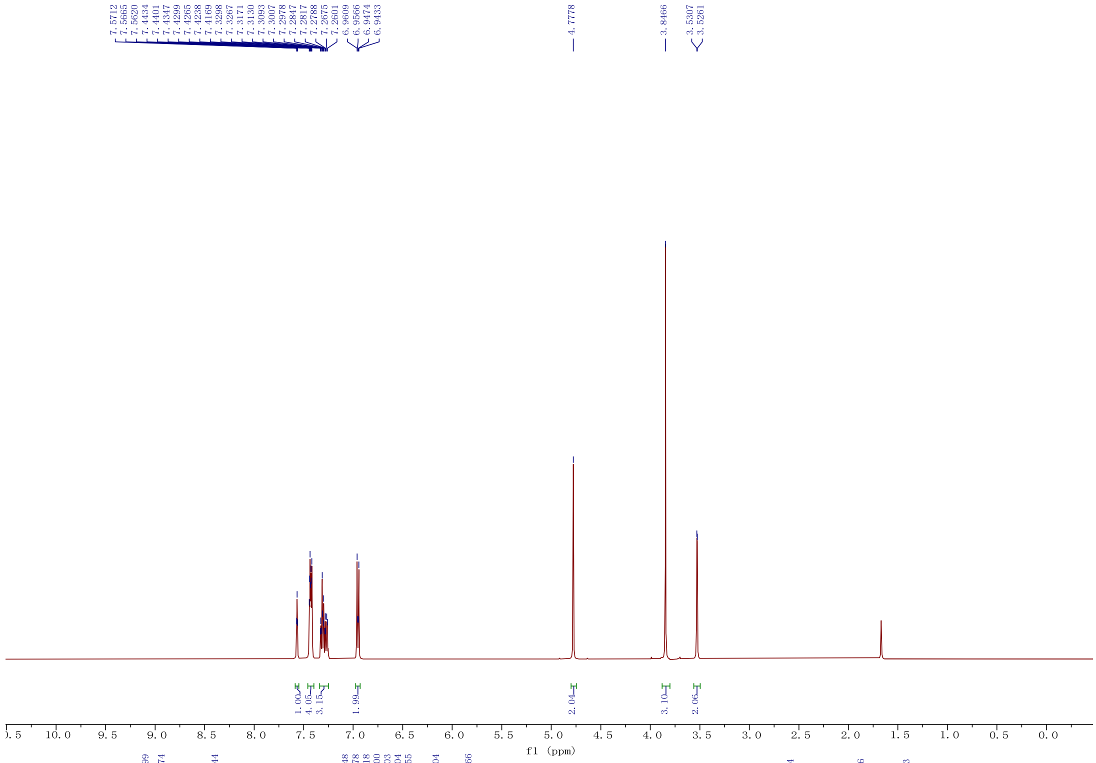
7.5712
7.5665
7.5620
7.4434
7.4401
7.4347
7.4299
7.4265
7.4238
7.4169
7.3298
7.3267
7.3171
7.3130
7.3093
7.3007
7.2978
7.2847
7.2817
7.2788
7.2675
7.2601
6.9609
6.9566
6.9474
6.9433
4.7778
3.8466
3.5307
3.5261
1.00
4.05
3.15
1.99
2.04
3.10
2.06
f1 (ppm)

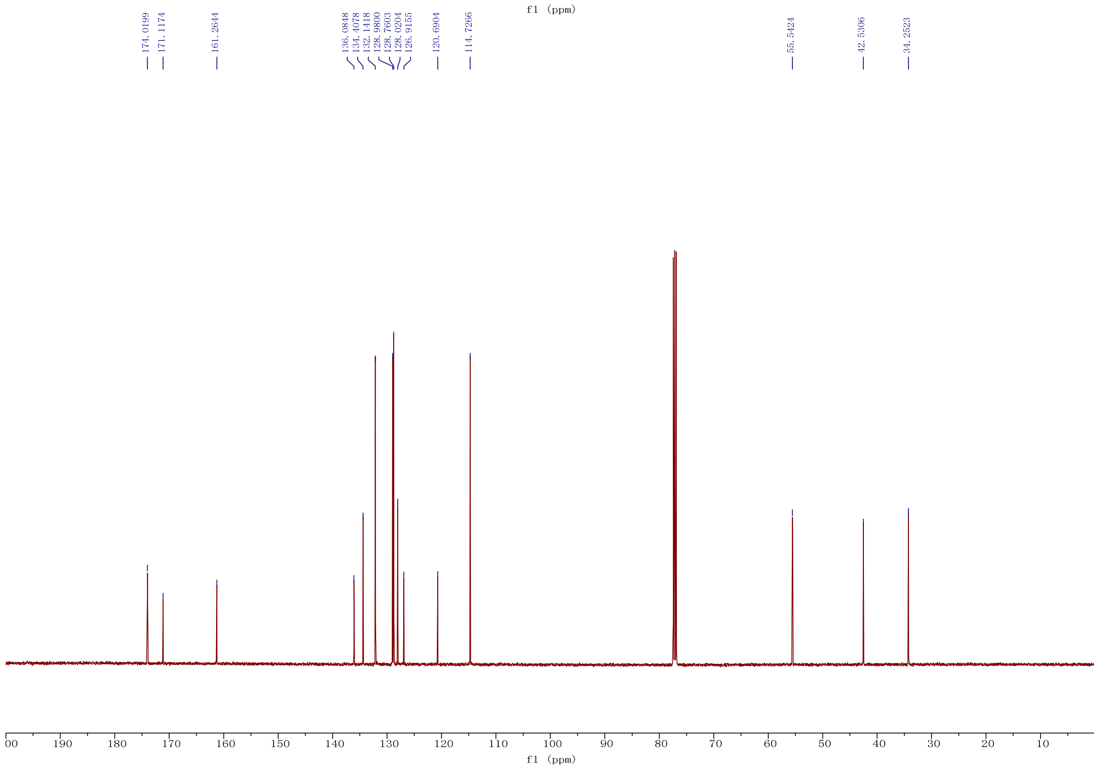
174.0199
171.1174
161.2644
136.0848
134.4078
132.1418
128.9800
128.7603
128.0204
126.9155
120.6904
114.7266
55.5424
42.5306
34.2523
f1 (ppm)

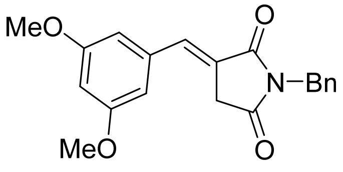
MeO
O
N−Bn
MeO
O

1j

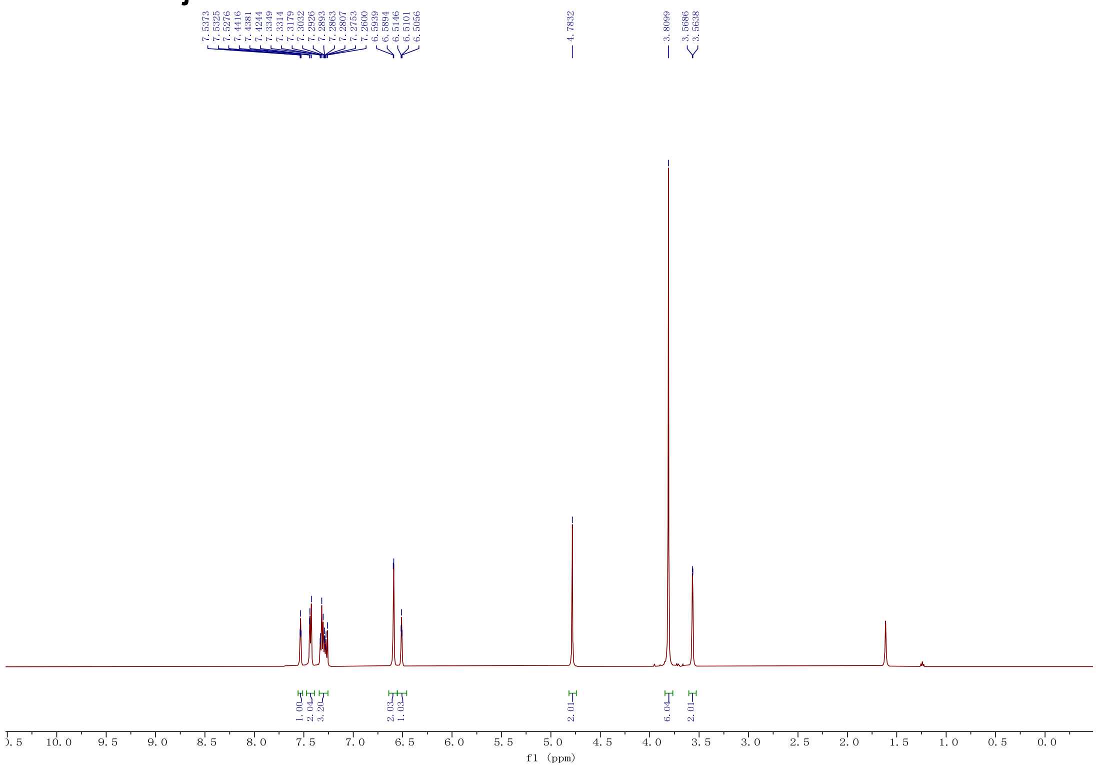
7.5373
7.5325
7.5276
7.4416
7.4381
7.4244
7.3349
7.3314
7.3179
7.3032
7.2926
7.2893
7.2863
7.2807
7.2753
7.2600
6.5939
6.5894
6.5146
6.5101
6.5056
4.7832
3.8099
3.5686
3.5638
1.00
2.04
3.20
2.03
1.03
2.01
6.04
2.01
f1 (ppm)

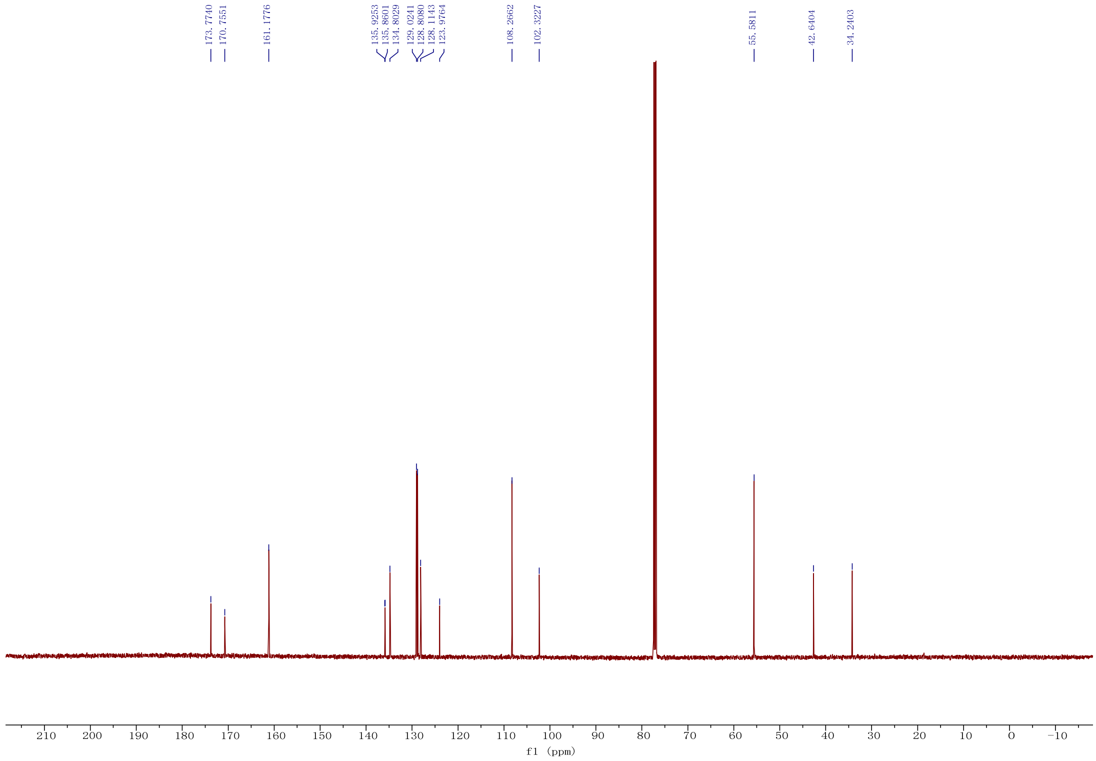
173.7740
170.7551
161.1776
135.9253
135.8601
134.8029
129.0241
128.8080
128.1143
123.9764
108.2662
102.3227
55.5811
42.6404
34.2403
f1 (ppm)

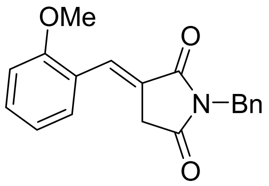
OMe
O
N−Bn
O

1k

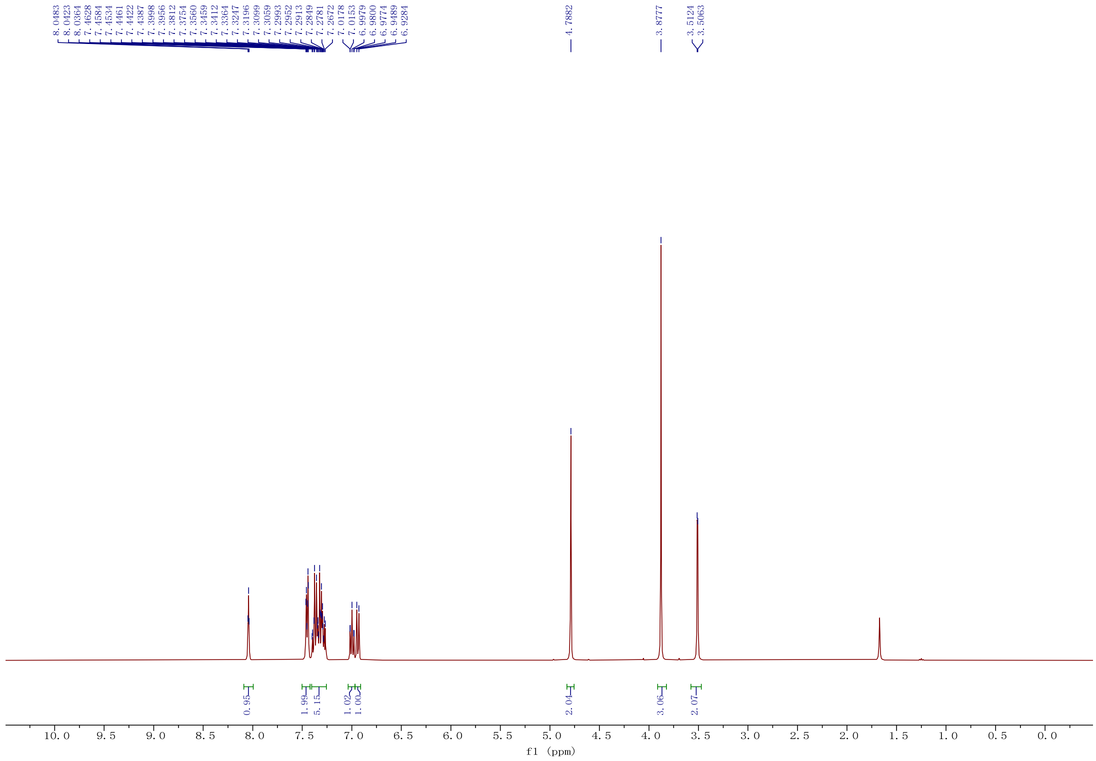
8.0483
8.0423
8.0364
7.4628
7.4581
7.4534
7.4461
7.4422
7.4387
7.3998
7.3956
7.3812
7.3754
7.3560
7.3459
7.3412
7.3364
7.3247
7.3196
7.3099
7.3059
7.2993
7.2952
7.2913
7.2849
7.2781
7.2672
7.0178
7.0153
6.9979
6.9800
6.9774
6.9489
6.9284
4.7882
3.8777
3.5124
3.5063
0.95
1.99
5.15
1.02
1.00
2.04
3.06
2.07
10.0 9.5 9.0 8.5 8.0 7.5 7.0 6.5 6.0 5.5 5.0 4.5 4.0 3.5 3.0 2.5 2.0 1.5 1.0 0.5 0.0
f1 (ppm)

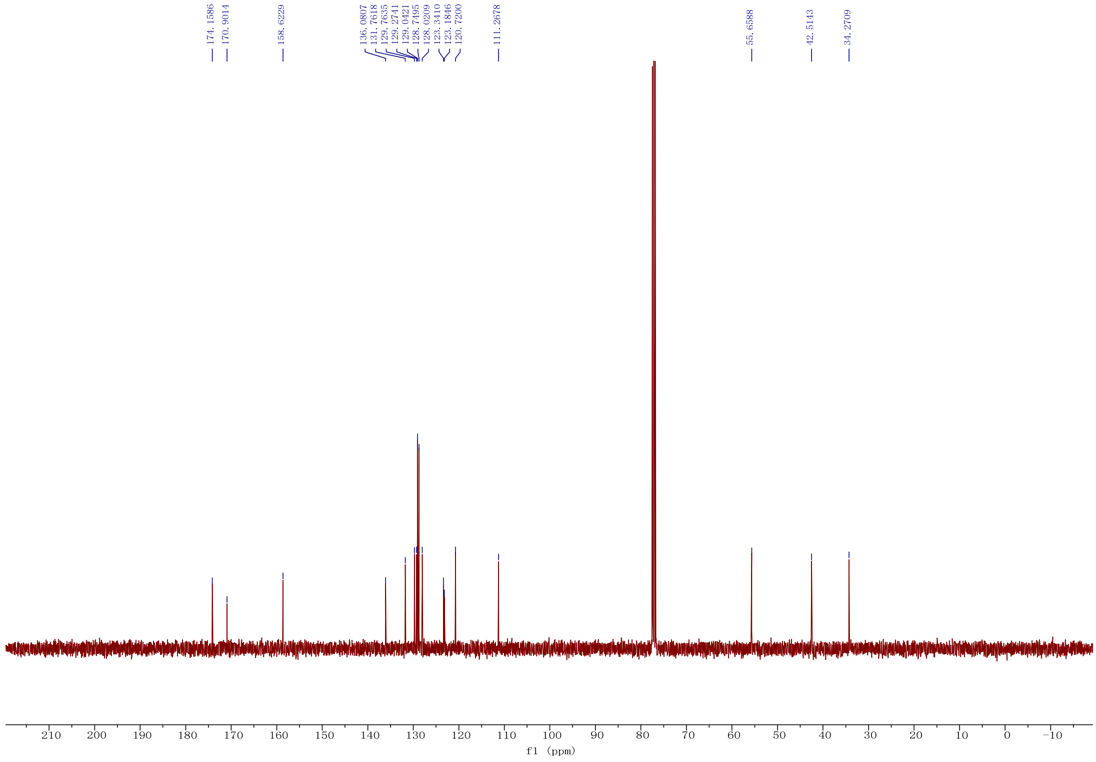
174.1586
170.9014
158.6229
136.0807
131.7618
129.7635
129.2741
129.0421
128.7495
128.0209
123.3410
123.1846
120.7200
111.2678
55.6588
42.5143
34.2709
210 200 190 180 170 160 150 140 130 120 110 100 90 80 70 60 50 40 30 20 10 0 −10
f1 (ppm)

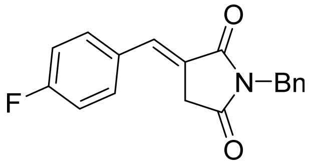

1l

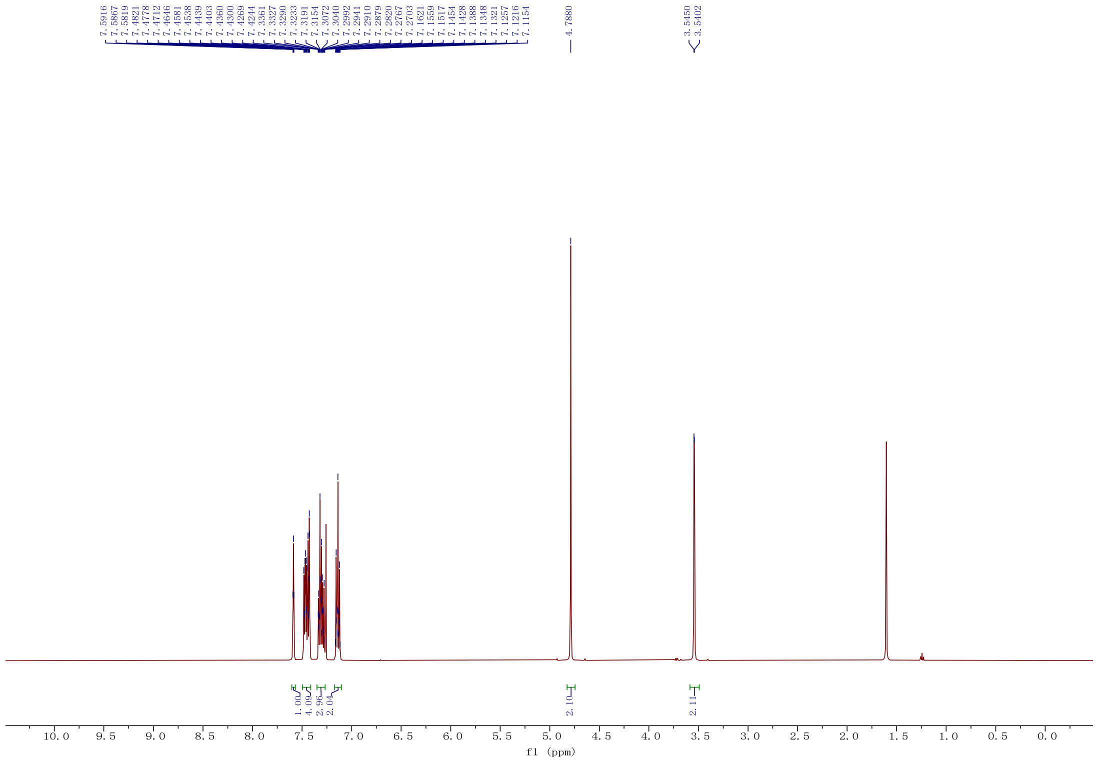

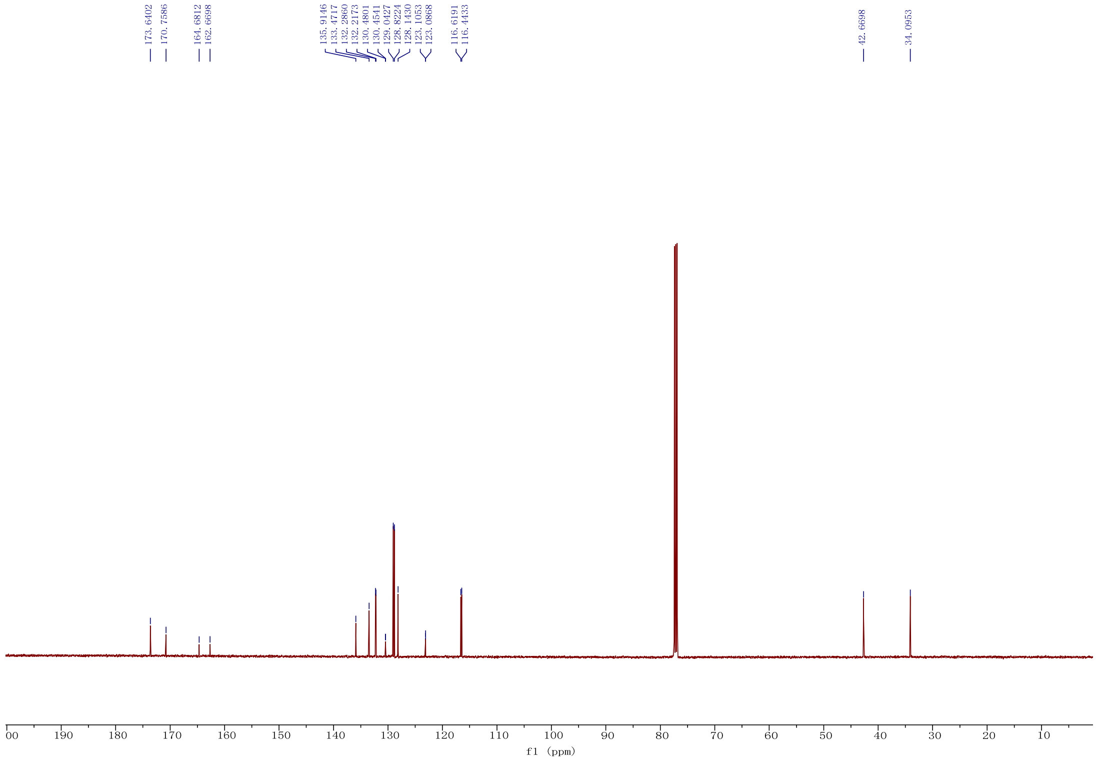

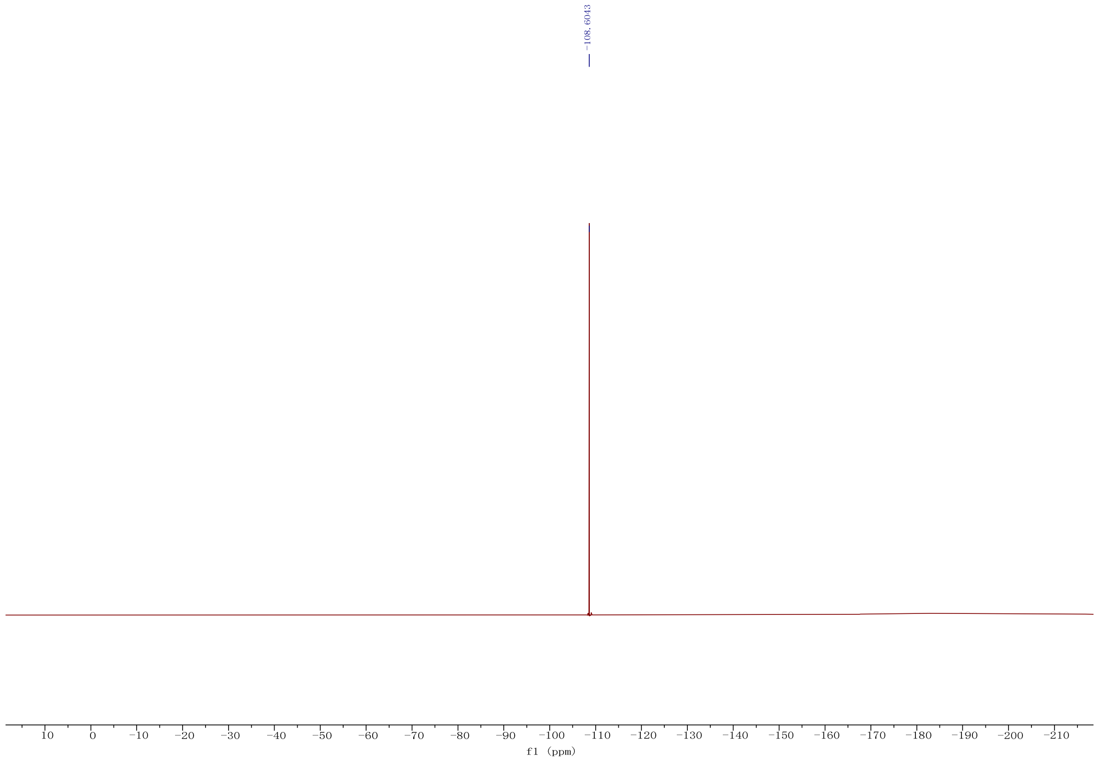

-108.6043
10
0
-10
-20
-30
-40
-50
-60
-70
-80
-90
-100
-110
-120
-130
-140
-150
-160
-170
-180
-190
-200
-210
f1 (ppm)

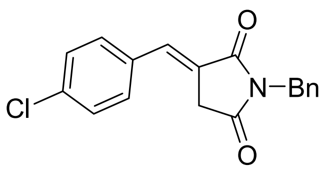
Cl
O
N−Bn
O

1m

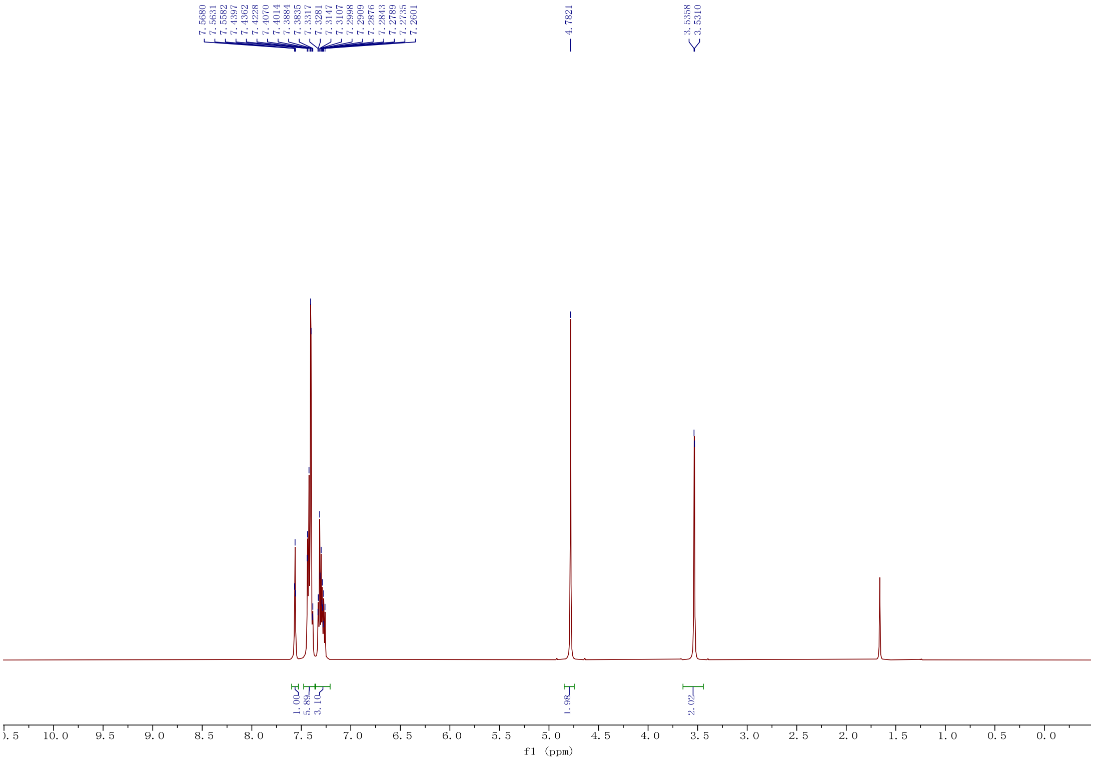
7.5680
7.5631
7.5582
7.4397
7.4362
7.4228
7.4070
7.4014
7.3884
7.3855
7.3317
7.3281
7.3147
7.3107
7.2968
7.2909
7.2876
7.2843
7.2789
7.2735
7.2601
4.7821
3.5358
3.5310
1.00
5.89
3.10
1.98
2.02
f1 (ppm)

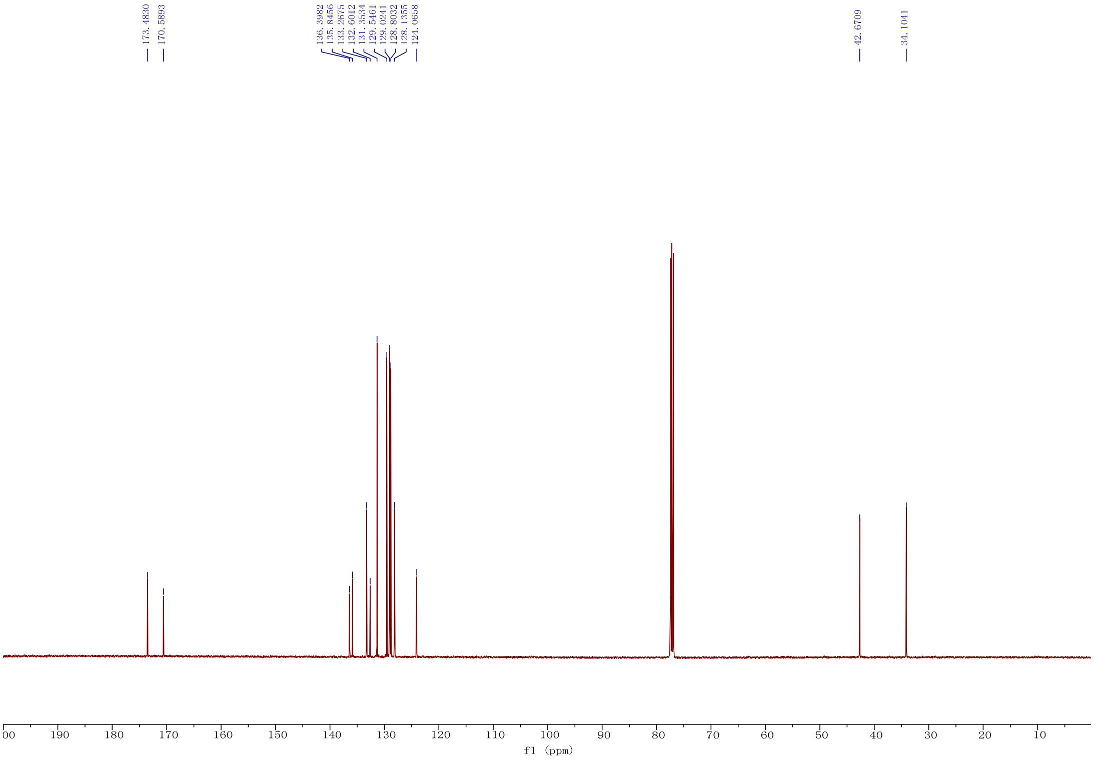
173.4830
170.5893
136.3982
135.8456
133.2675
132.6012
131.3534
129.5461
129.0241
128.8632
128.1355
124.0658
42.6709
34.1041
f1 (ppm)

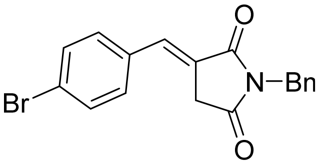
O
N−Bn
Br
O

1n

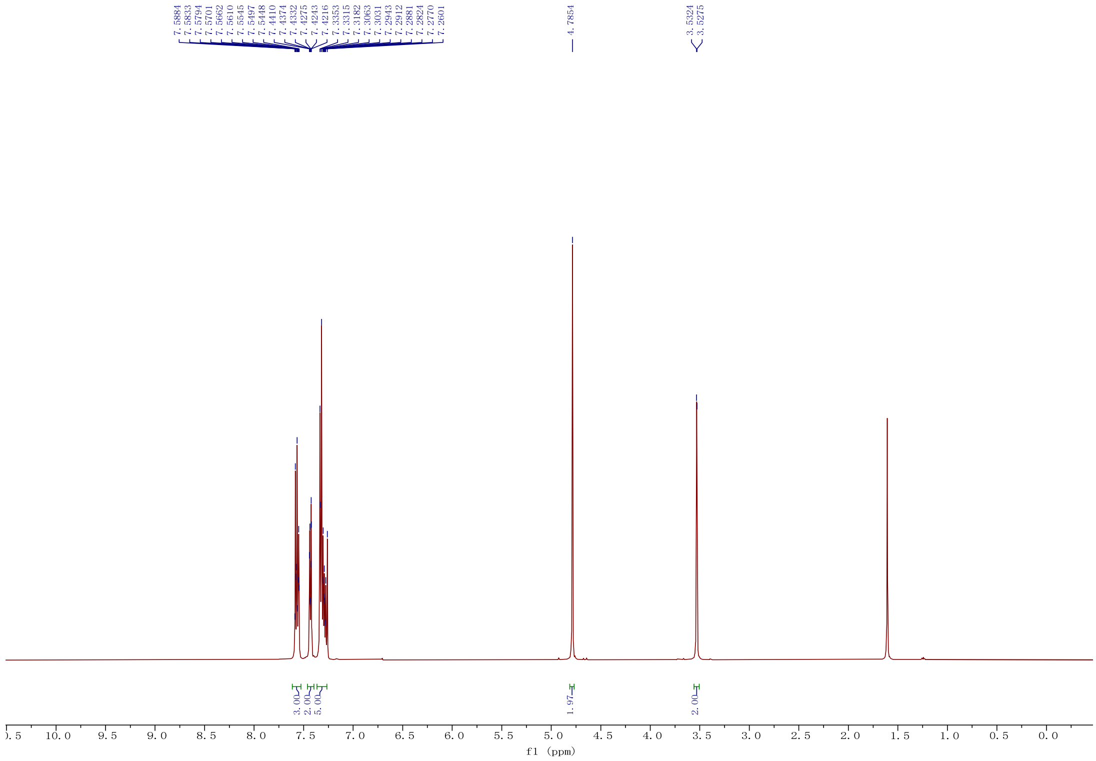
7.5884
7.5833
7.5794
7.5701
7.5662
7.5610
7.5545
7.5497
7.5448
7.4410
7.4374
7.4332
7.4275
7.4243
7.4216
7.3353
7.3315
7.3182
7.3063
7.3031
7.2943
7.2912
7.2881
7.2824
7.2770
7.2601
4.7854
3.5324
3.5275
3.00
2.00
5.00
1.97
2.00
f1 (ppm)

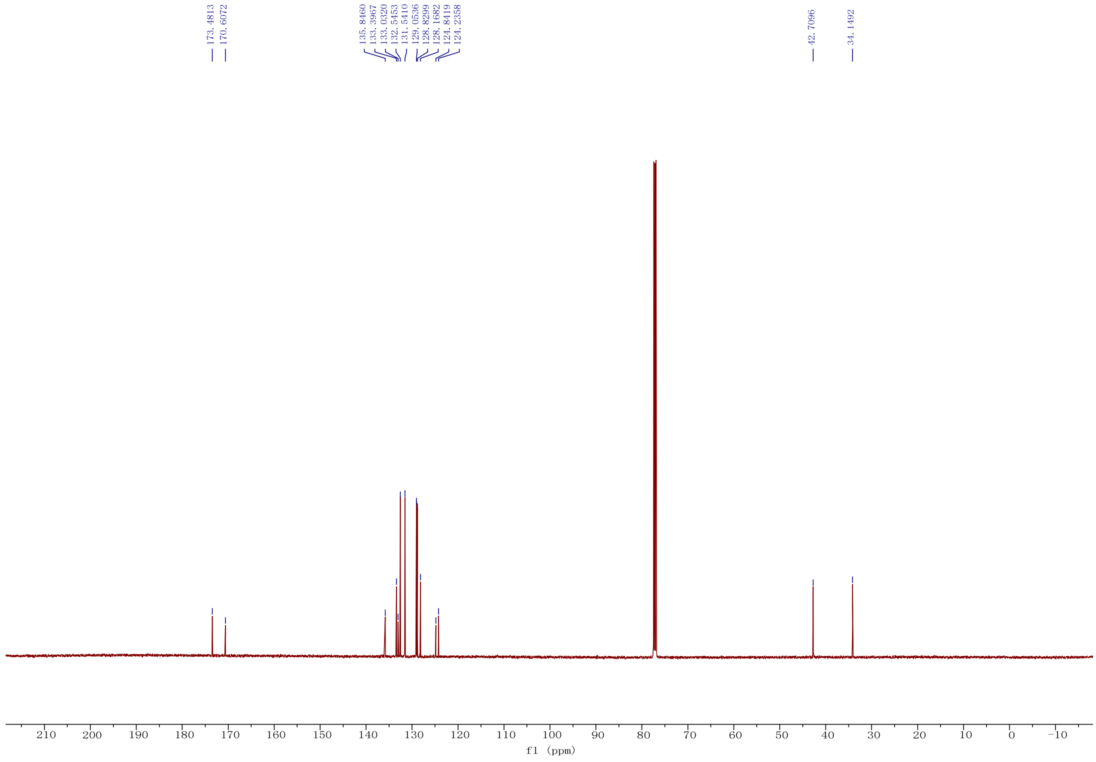
173.4813
170.6072
135.8460
133.3967
133.0320
132.5453
131.5410
129.0536
128.8299
128.1682
124.8419
124.2358
42.7096
34.1492
f1 (ppm)

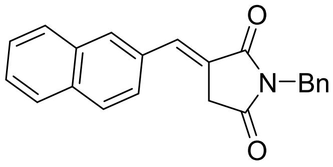
O
N−Bn
O

1o

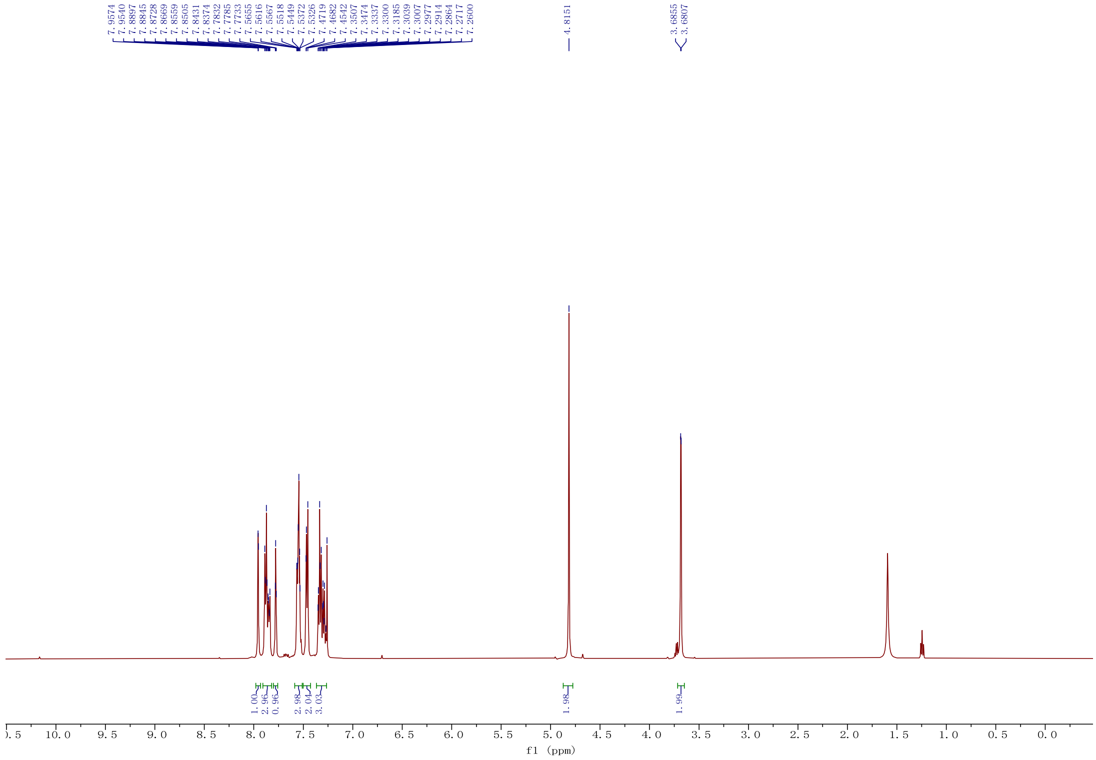
7.9574
7.9540
7.8897
7.8845
7.8728
7.8669
7.8559
7.8505
7.8431
7.8374
7.7832
7.7785
7.7733
7.5655
7.5616
7.5567
7.5518
7.5449
7.5372
7.5326
7.4719
7.4682
7.4542
7.3507
7.3474
7.3337
7.3300
7.3185
7.3039
7.3007
7.2977
7.2914
7.2864
7.2717
7.2600
4.8151
3.6855
3.6807
1.00
2.96
0.96
2.98
2.04
3.03
1.98
1.99
f1 (ppm)

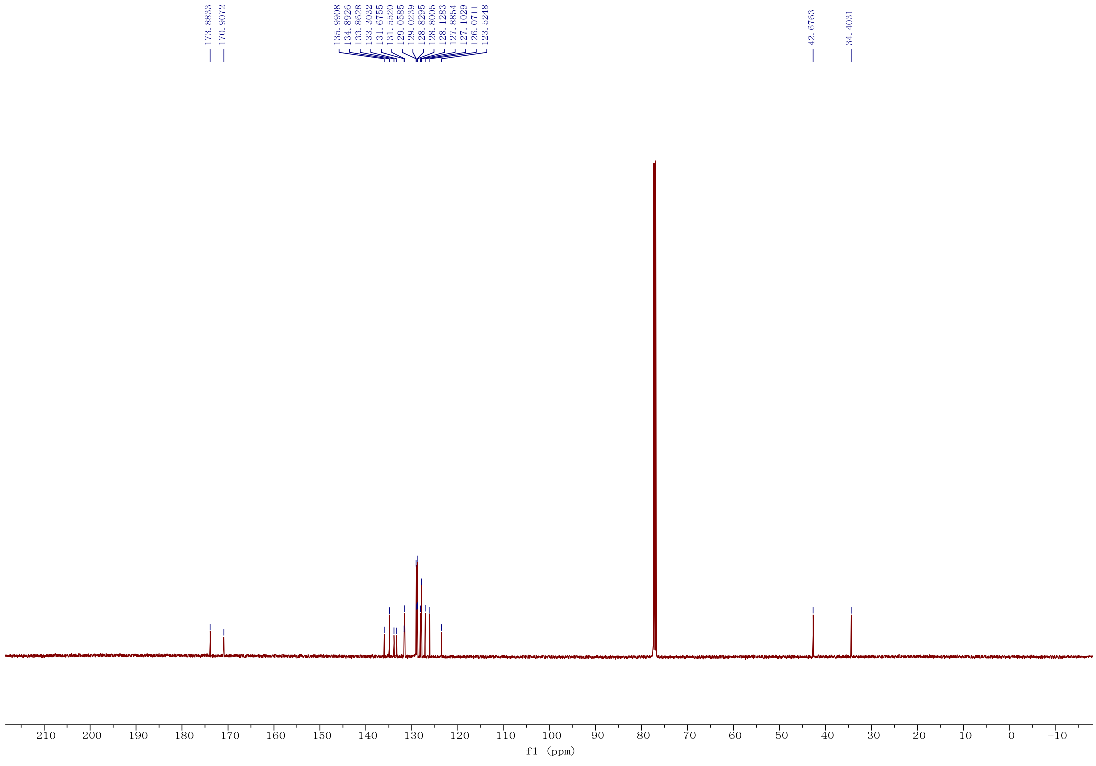
173.8833
170.9072
135.9908
134.8926
133.8628
133.3032
131.6755
131.5520
129.0585
129.0239
128.8295
128.8005
128.1283
127.8854
127.1029
126.0711
123.5248
42.6763
34.4031
f1 (ppm)

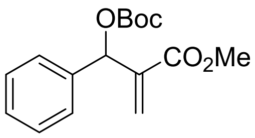
OBoc
CO_2Me

2a

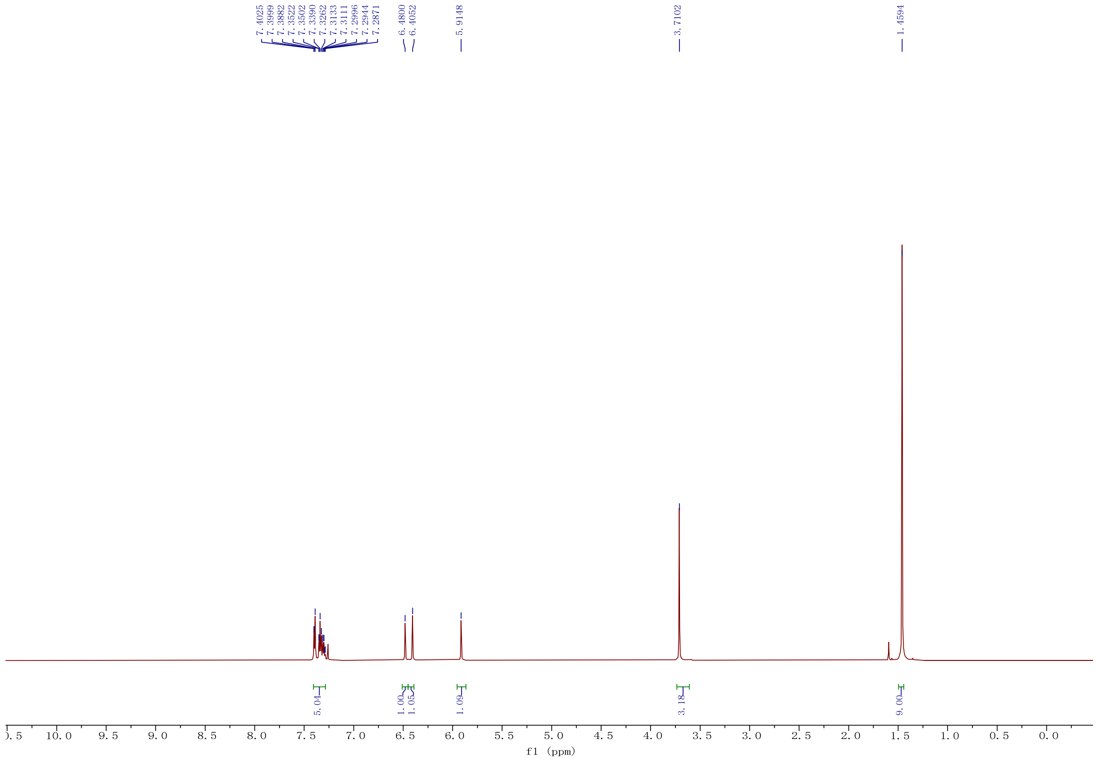
7.4025
7.3999
7.3882
7.3522
7.3502
7.3380
7.3262
7.3133
7.3111
7.2966
7.2944
7.2871
6.4800
6.4052
5.9148
3.7102
1.4594
5.04
1.00
1.05
1.09
3.18
9.00
10.0 9.5 9.0 8.5 8.0 7.5 7.0 6.5 6.0 5.5 5.0 4.5 4.0 3.5 3.0 2.5 2.0 1.5 1.0 0.5 0.0
f1 (ppm)

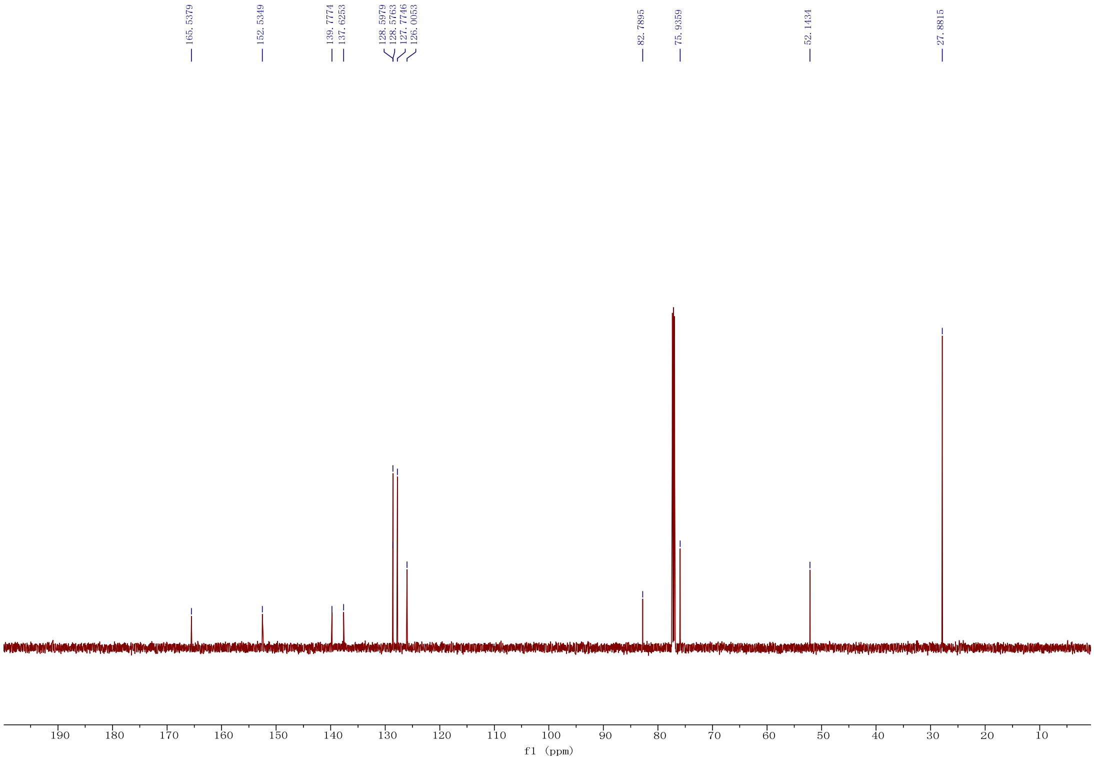
165.5379
152.5349
139.7774
137.6253
128.5979
128.5763
127.7746
126.0053
82.7895
75.9359
52.1434
27.8815
190 180 170 160 150 140 130 120 110 100 90 80 70 60 50 40 30 20 10
f1 (ppm)

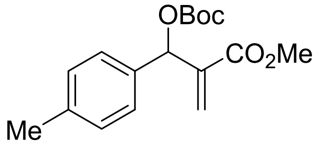
OBoc
CO2Me
Me

2b

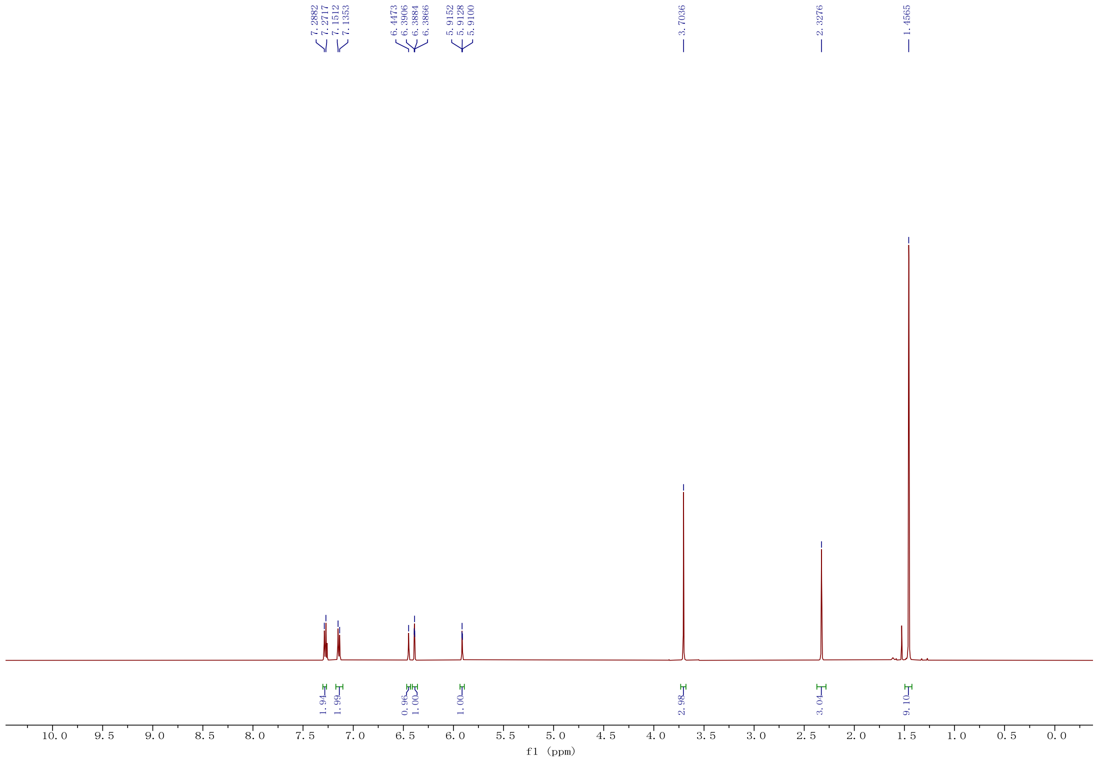
7.2882
7.2717
7.1512
7.1353
6.4473
6.3906
6.3884
6.3866
5.9152
5.9128
5.9100
3.7036
2.3276
1.4565
1.94
1.99
0.96
1.00
1.00
2.98
3.04
9.10
f1 (ppm)

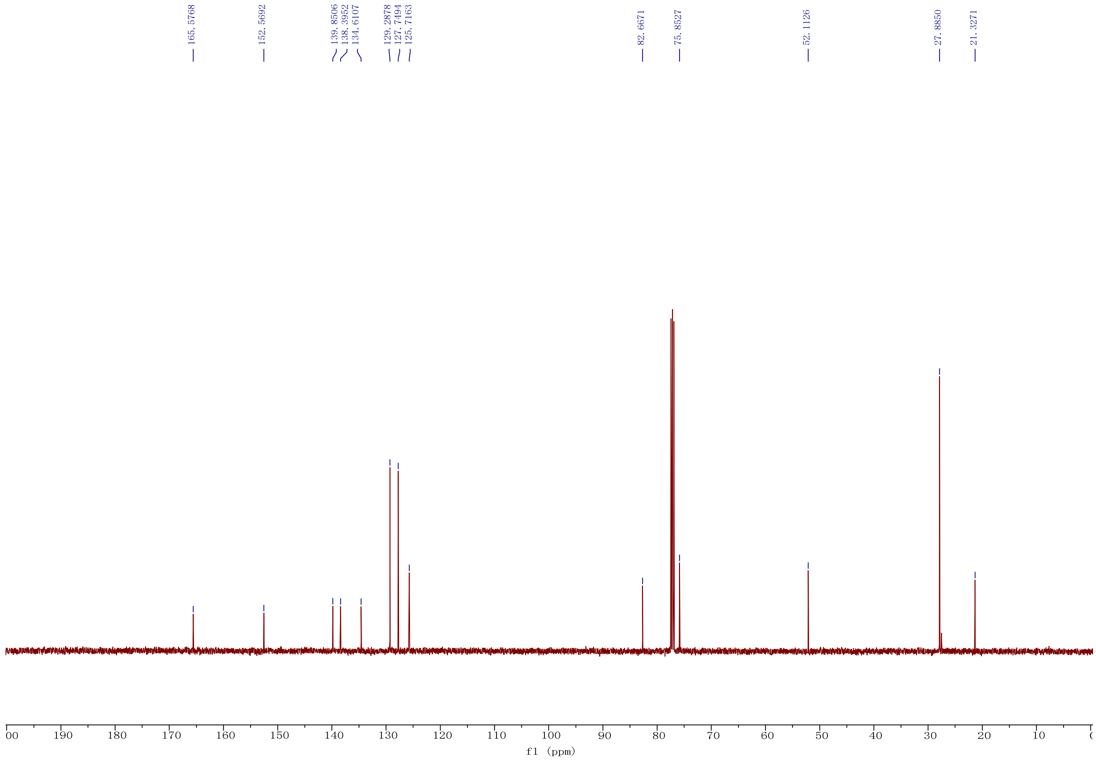
165.5768
152.5692
139.8506
138.3952
134.6107
129.2878
127.7494
125.7163
82.6671
75.8527
52.1126
27.8850
21.3271
f1 (ppm)

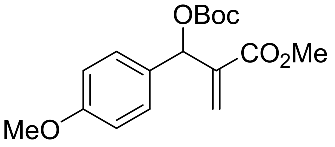
OBoc
CO2Me
MeO

2c

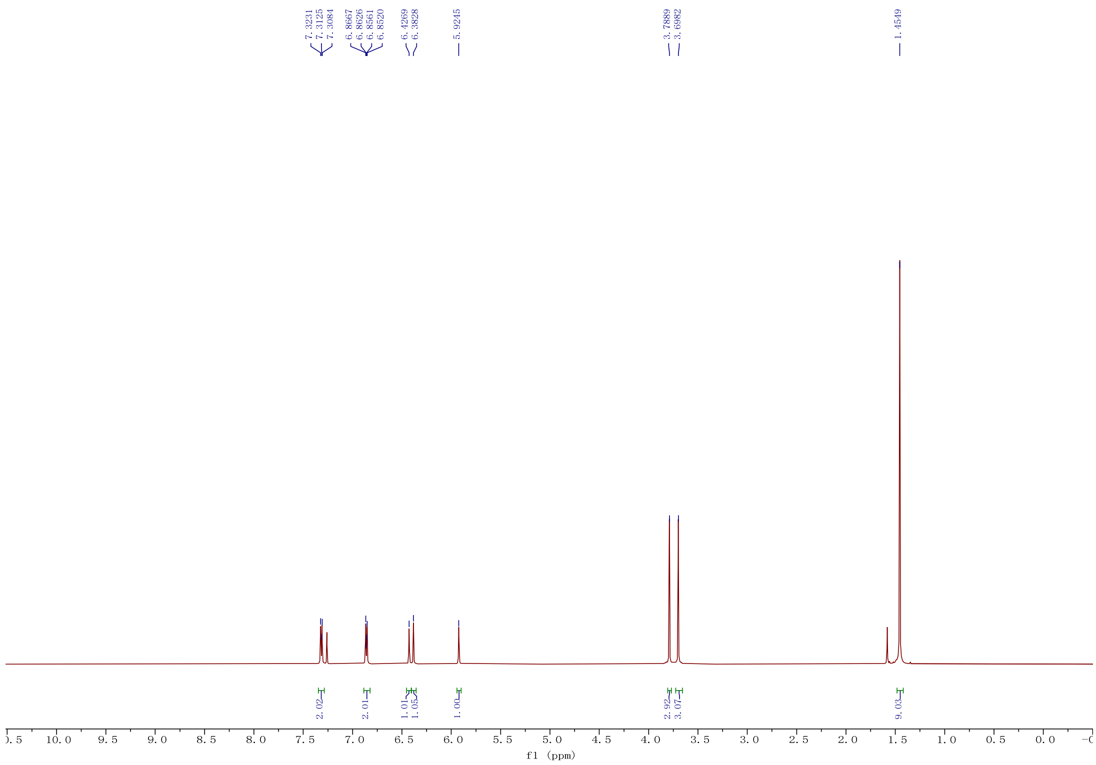
7.3231
7.3125
7.3084
6.8667
6.8626
6.8561
6.8520
6.4269
6.3828
5.9245
3.7889
3.6982
1.4549
2.02
2.01
1.01
1.05
1.00
2.92
3.07
9.03
f1 (ppm)

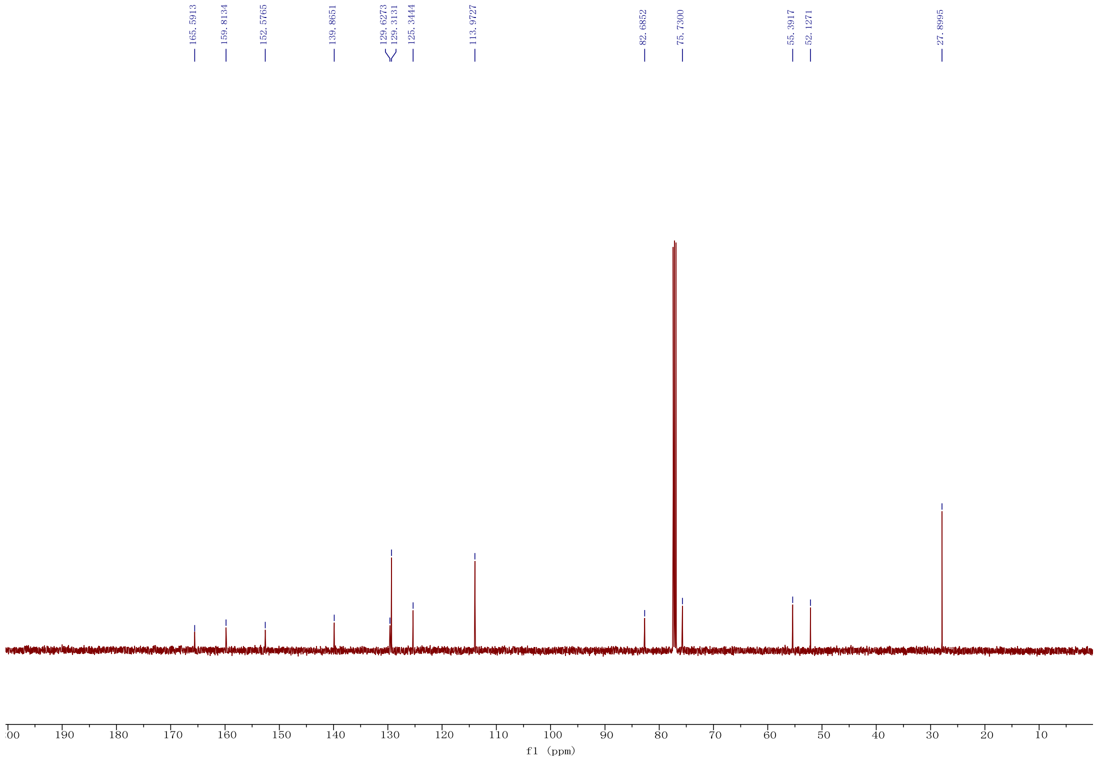
165.5913
159.8134
152.5765
139.8651
129.6273
129.3131
125.3444
113.9727
82.6852
75.7300
55.3917
52.1271
27.8995
f1 (ppm)

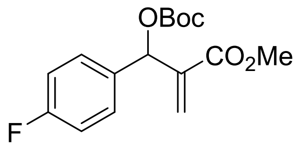

2d

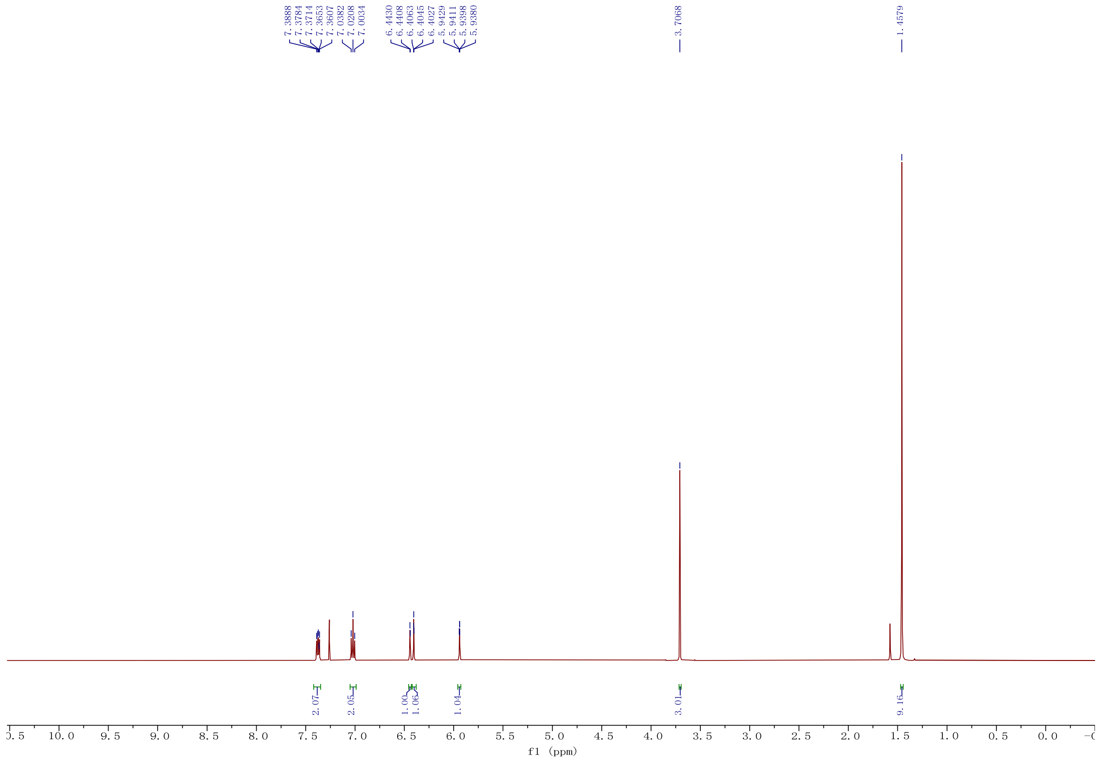

7.3888
7.3784
7.3714
7.3653
7.3607
7.0382
7.0208
7.0034
6.4430
6.4408
6.4063
6.4045
6.4027
5.9429
5.9411
5.9398
5.9380
3.7068
1.4579
2.07
2.05
1.00
1.06
1.04
3.01
9.16
f1 (ppm)

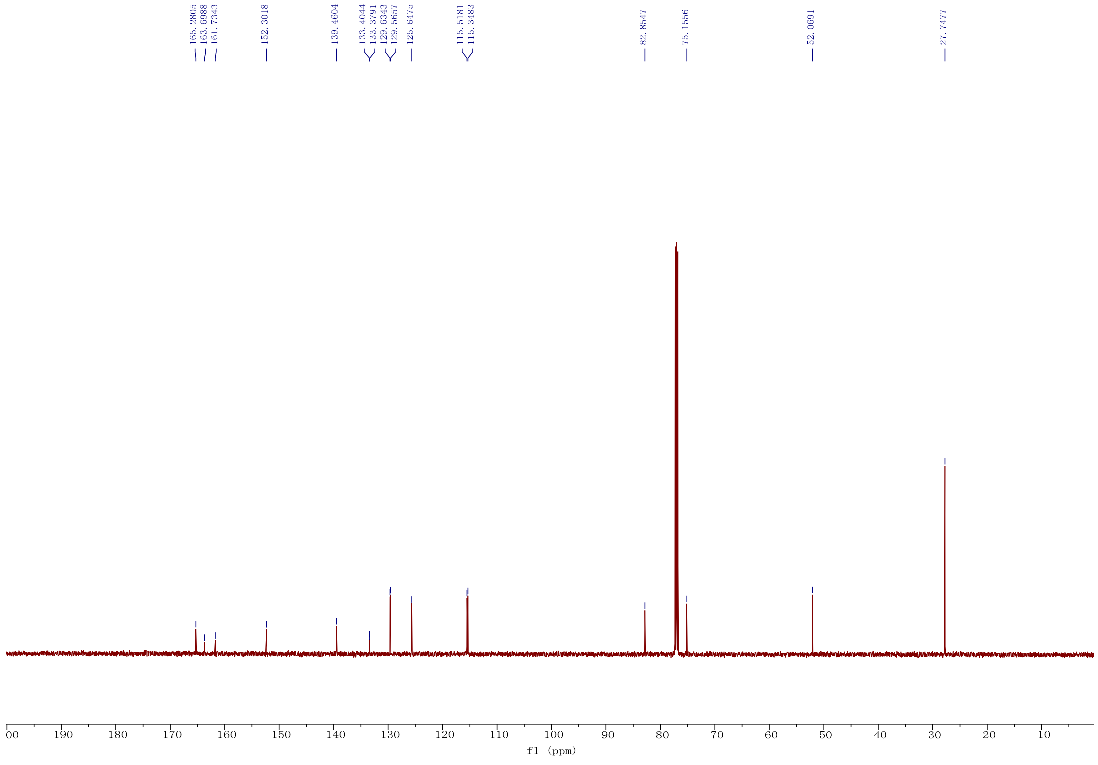

165.2805
163.6988
161.7343
152.3018
139.4604
133.4044
133.3791
129.6343
129.5657
125.6475
115.5181
115.3483
82.8547
75.1556
52.0691
27.7477
f1 (ppm)

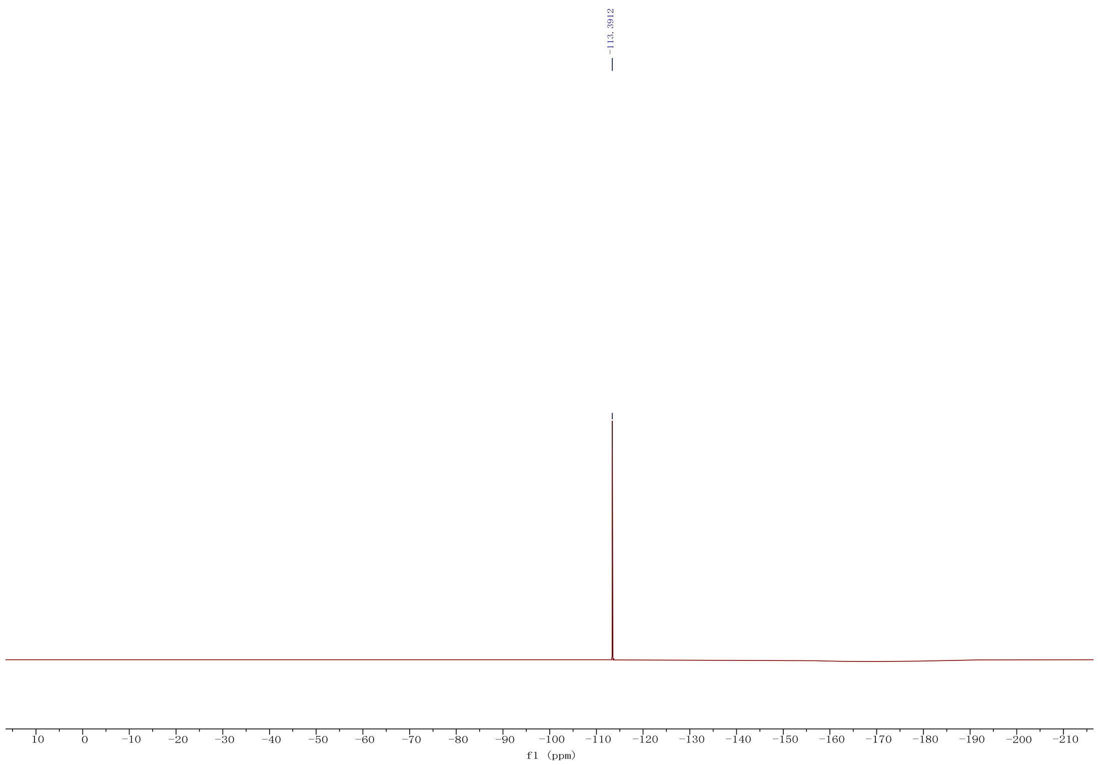
-113.3912
10
0
-10
-20
-30
-40
-50
-60
-70
-80
-90
-100
-110
-120
-130
-140
-150
-160
-170
-180
-190
-200
-210
f1 (ppm)

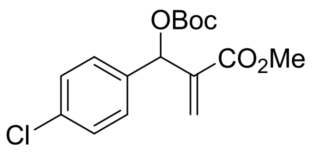
OBoc
CO2Me
Cl

2e

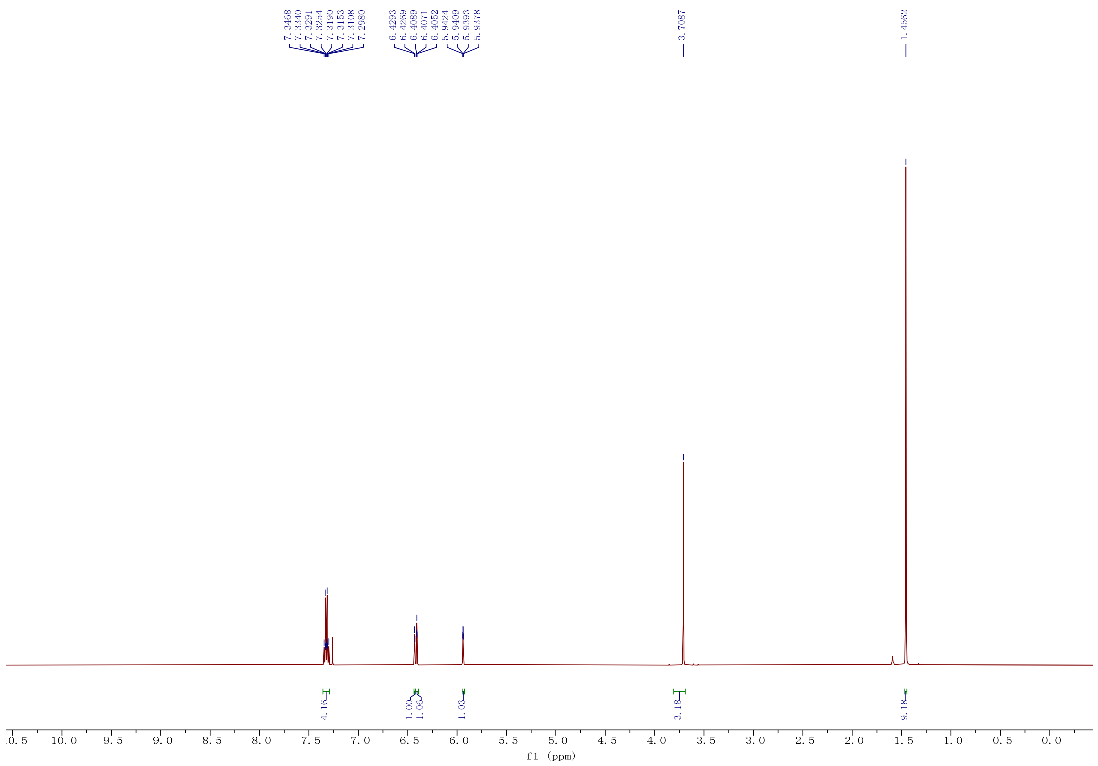
7.3468
7.3340
7.3291
7.3254
7.3190
7.3153
7.3108
7.2980
6.4293
6.4269
6.4089
6.4071
6.4052
5.9424
5.9409
5.9393
5.9378
3.7087
1.4562
4.16
1.00
1.06
1.03
3.18
9.18
f1 (ppm)

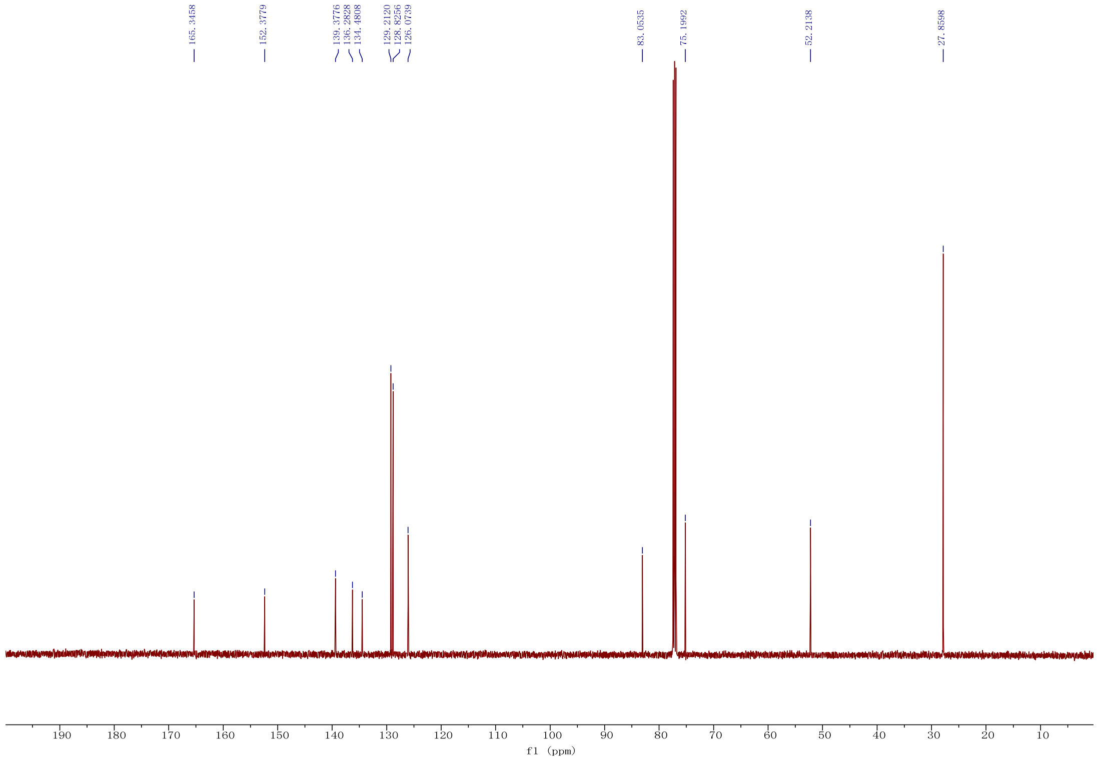
165.3458
152.3779
139.3776
136.2828
134.4808
129.2120
128.8256
126.0739
83.0535
75.1992
52.2138
27.8598
f1 (ppm)

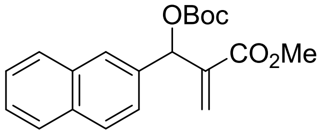
OBoc
CO2Me

2f

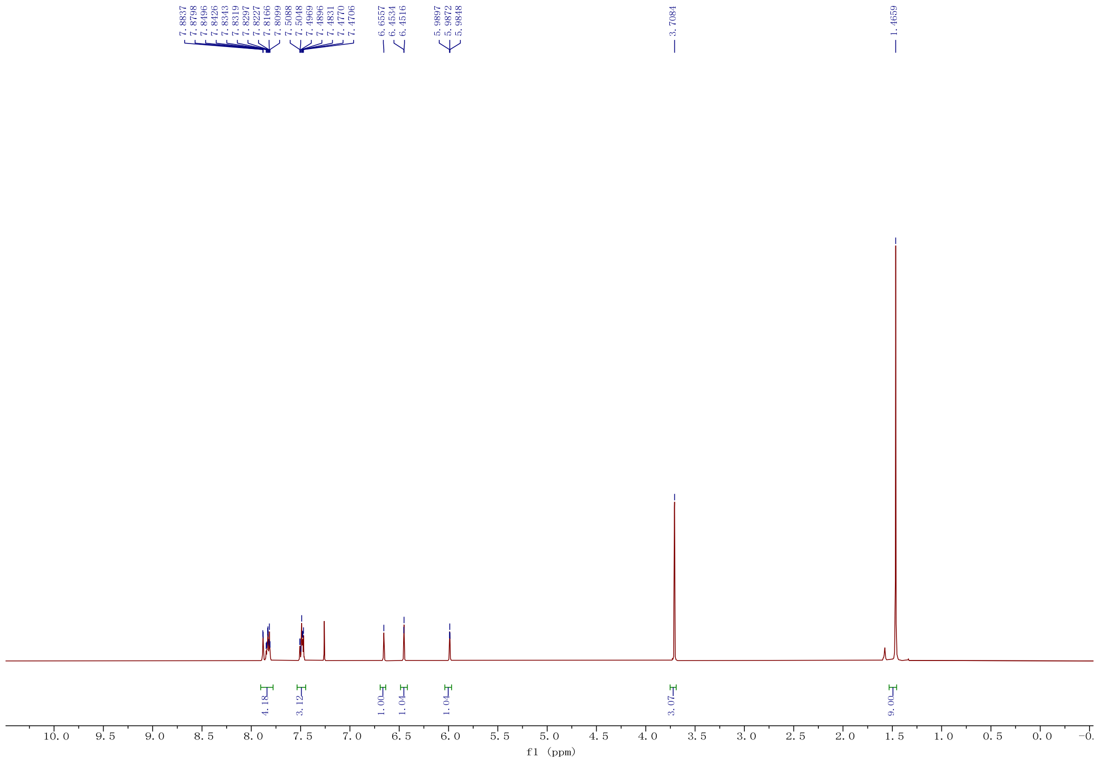
7.8837 7.8798 7.8496 7.8426 7.8343 7.8319 7.8297 7.8227 7.8166 7.8099 7.5088 7.5048 7.4969 7.4896 7.4831 7.4770 7.4706
6.6557 6.4534 6.4516
5.9897 5.9872 5.9848
3.7084
1.4659
4.18 3.12 1.00 1.04 1.04 3.07 9.00
f1 (ppm)

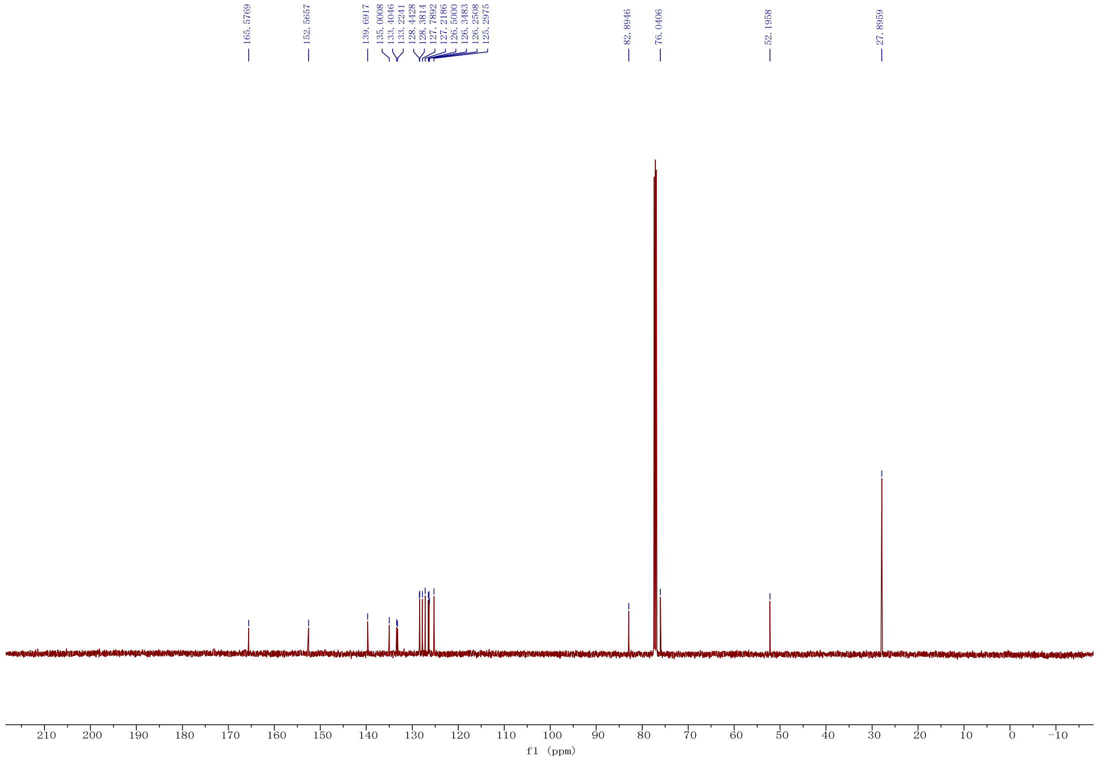
165.5769
152.5657
139.6917 135.0008 133.4046 133.2241 128.4428 128.3814 127.7892 127.2186 126.5000 126.3483 126.2508 125.2975
82.8946
76.0406
52.1958
27.8959
f1 (ppm)

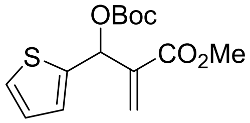
OBoc
S
CO2Me

2g

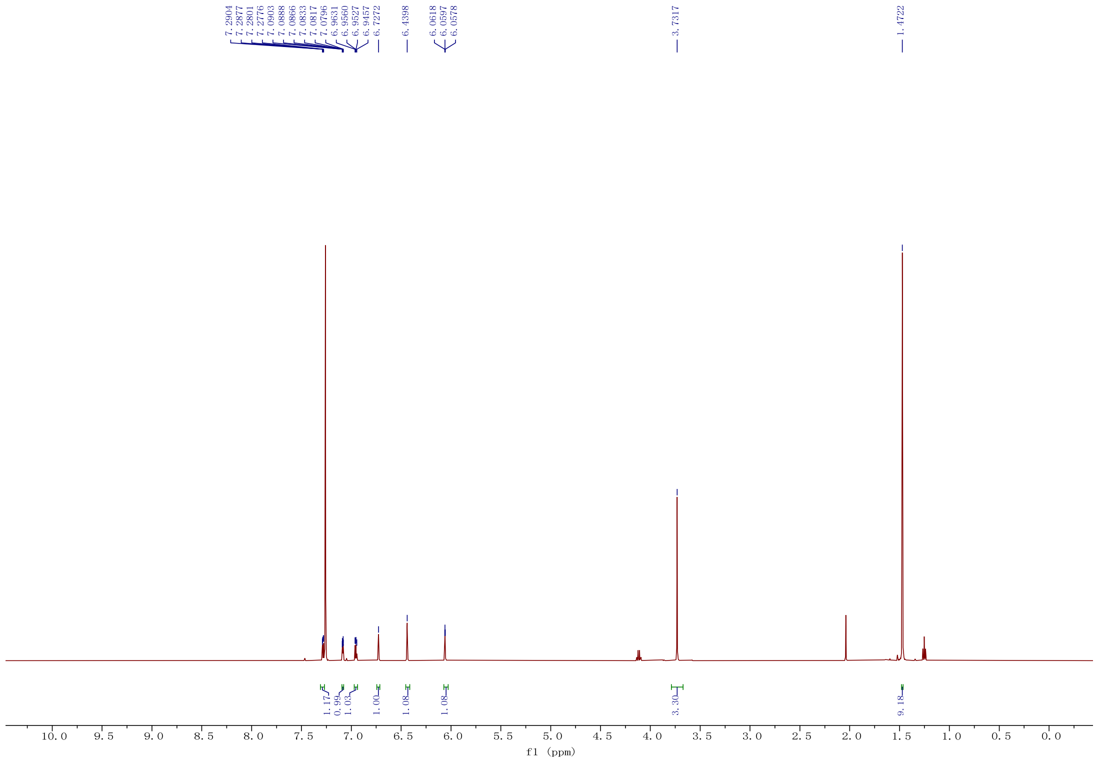
7.2904
7.2877
7.2801
7.2776
7.0903
7.0888
7.0866
7.0833
7.0847
7.0796
6.9631
6.9560
6.9527
6.9457
6.7272
6.4398
6.0618
6.0597
6.0578
3.7317
1.4722
1.17
0.99
1.03
1.00
1.08
1.08
3.30
9.18
10.0
9.5
9.0
8.5
8.0
7.5
7.0
6.5
6.0
5.5
5.0
4.5
4.0
3.5
3.0
2.5
2.0
1.5
1.0
0.5
0.0
f1 (ppm)

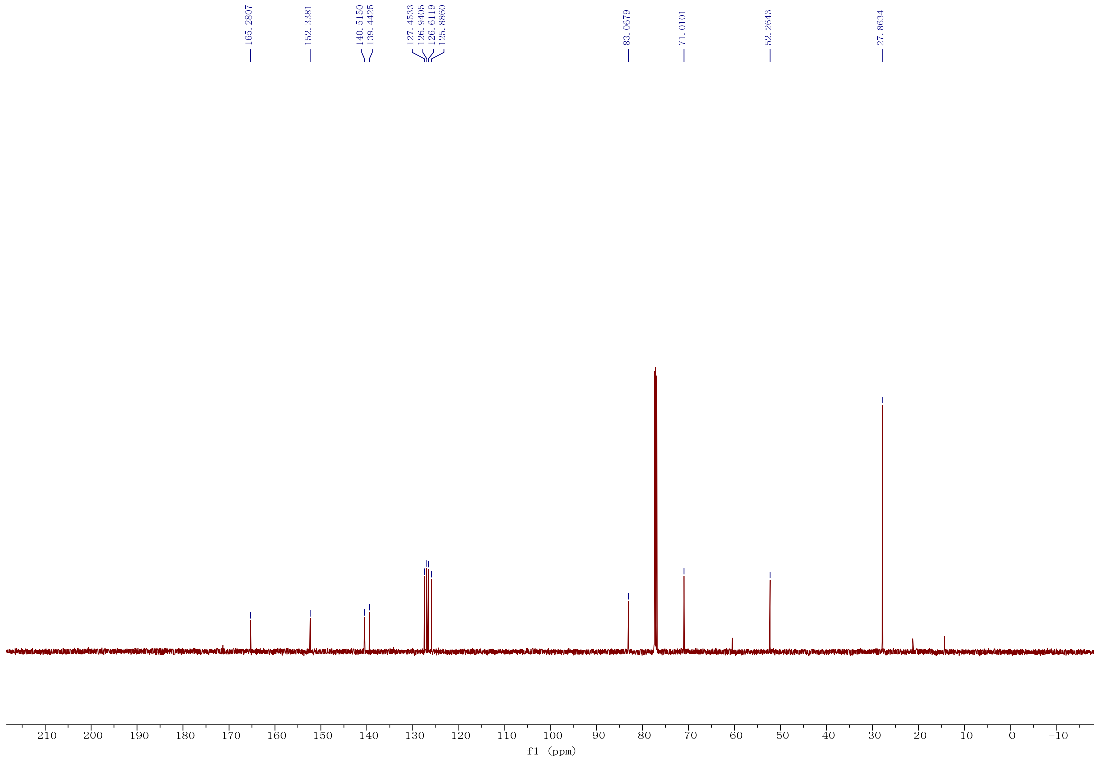
165.2807
152.3381
140.5150
139.4425
127.4533
126.9405
126.6119
125.8860
83.0679
71.0101
52.2643
27.8634
210
200
190
180
170
160
150
140
130
120
110
100
90
80
70
60
50
40
30
20
10
0
−10
f1 (ppm)

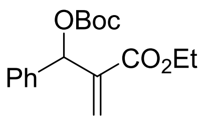
OBoc
Ph
CO2Et

2h

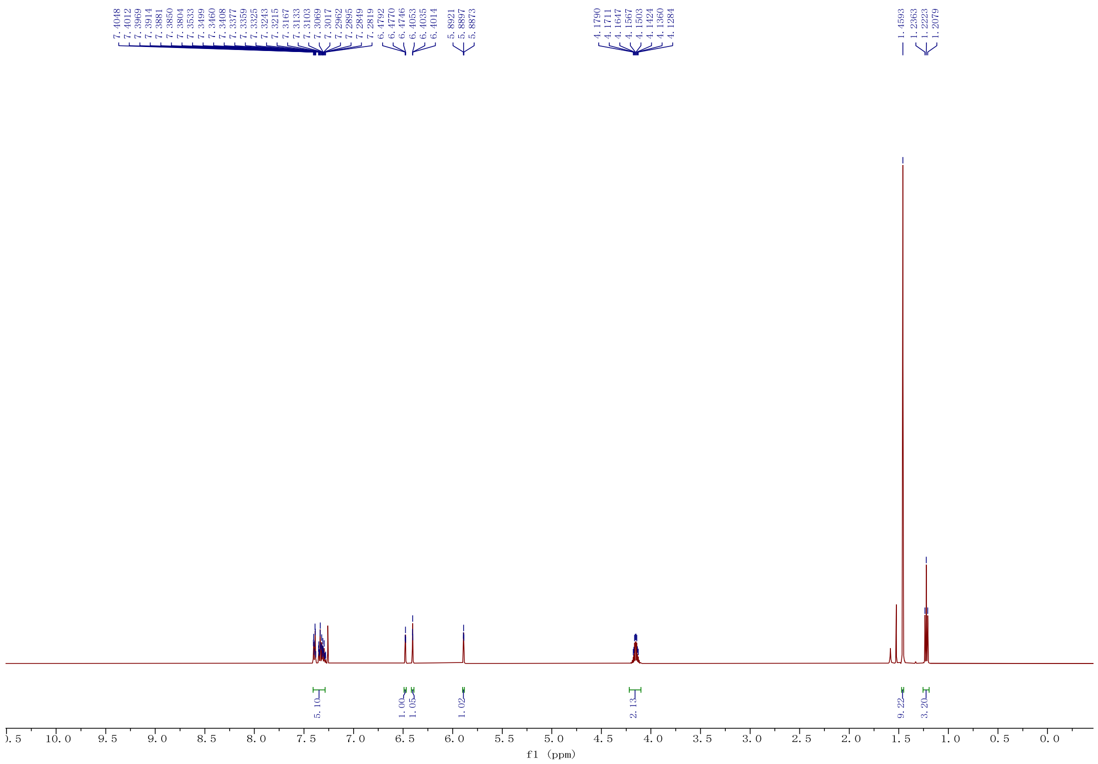
7.4048 7.4012 7.3969 7.3914 7.3881 7.3850 7.3804 7.3533 7.3499 7.3460 7.3408 7.3377 7.3359 7.3325 7.3243 7.3215 7.3167 7.3133 7.3103 7.3069 7.3017 7.2962 7.2895 7.2849 7.2819 6.4792 6.4770 6.4746 6.4053 6.4035 6.4014 5.8921 5.8897 5.8873
4.1790 4.1711 4.1647 4.1567 4.1503 4.1424 4.1360 4.1284
1.4593 1.2363 1.2223 1.2079
5.10 1.00 1.05 1.02 2.13 9.22 3.20
10.0 9.5 9.0 8.5 8.0 7.5 7.0 6.5 6.0 5.5 5.0 4.5 4.0 3.5 3.0 2.5 2.0 1.5 1.0 0.5 0.0
f1 (ppm)

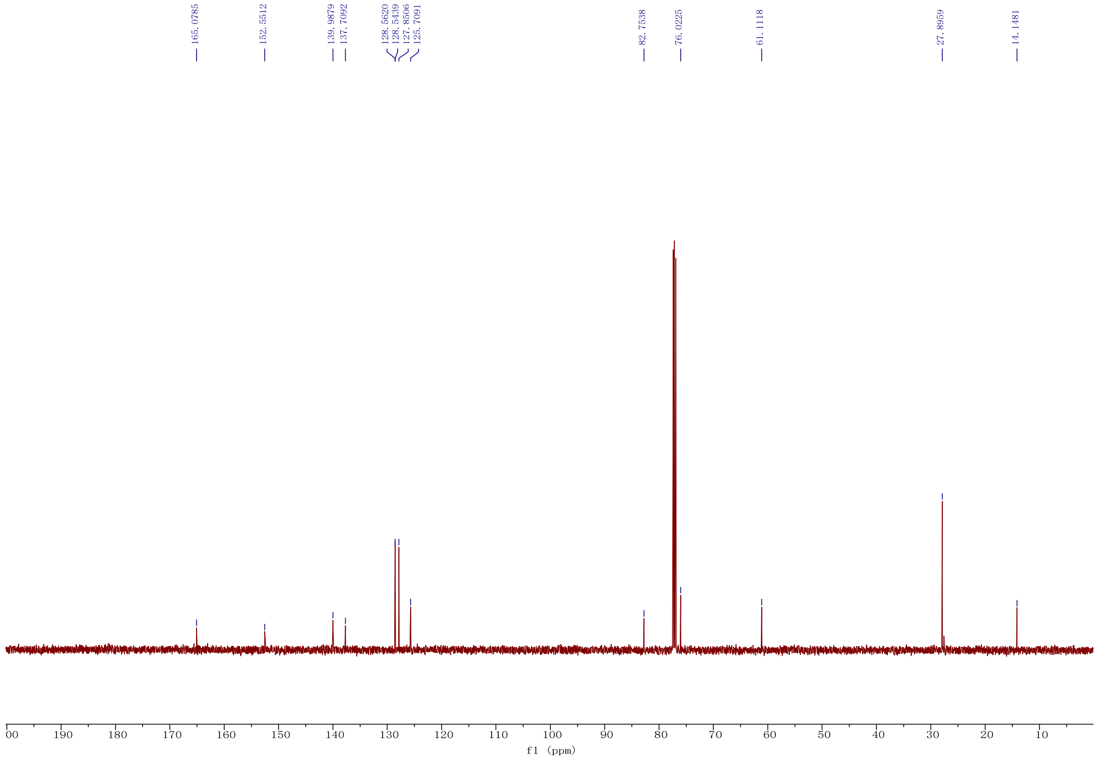
165.0785
152.5512
139.9879 137.7092
128.5620 128.5439 127.8506 125.7091
82.7538
76.0225
61.1118
27.8959
14.1481
190 180 170 160 150 140 130 120 110 100 90 80 70 60 50 40 30 20 10
f1 (ppm)

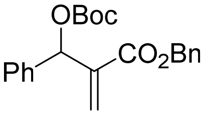
OBoc
Ph
CO2Bn

2i

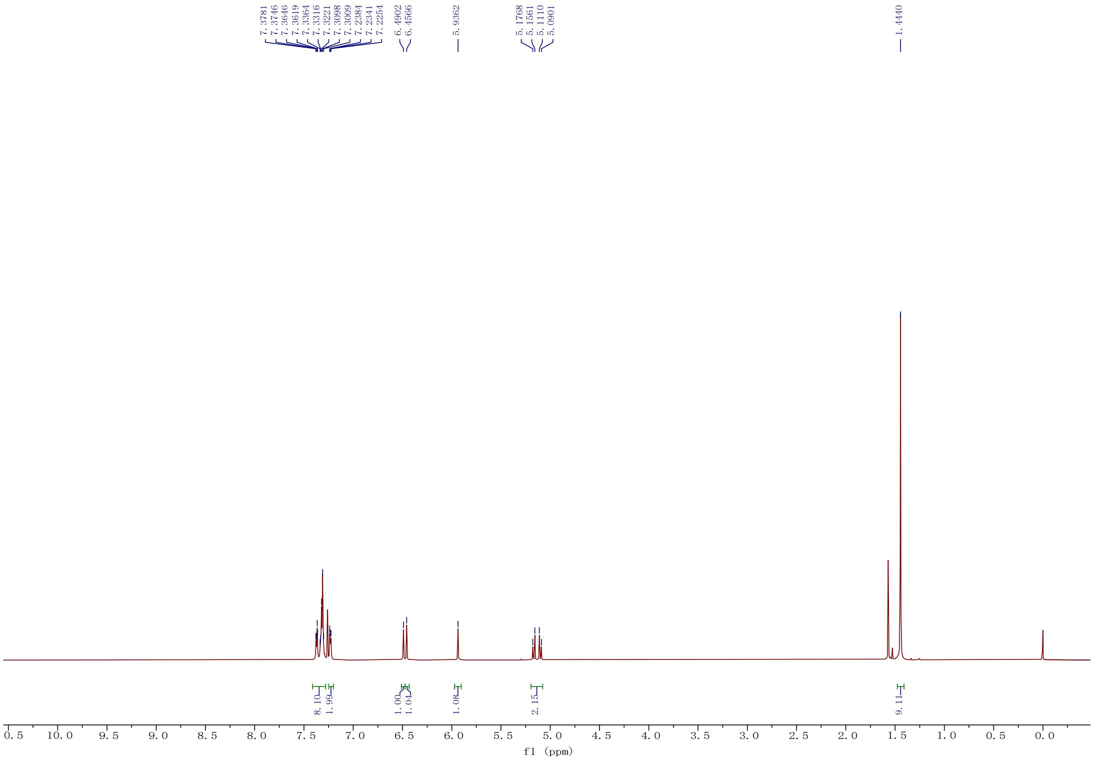
7.3781
7.3746
7.3646
7.3619
7.3364
7.3316
7.3221
7.3098
7.3009
7.2384
7.2341
7.2254
6.4902
6.4566
5.9362
5.1768
5.1561
5.1110
5.0901
1.4440
8.10
1.95
1.00
1.04
1.08
2.15
9.11
f1 (ppm)

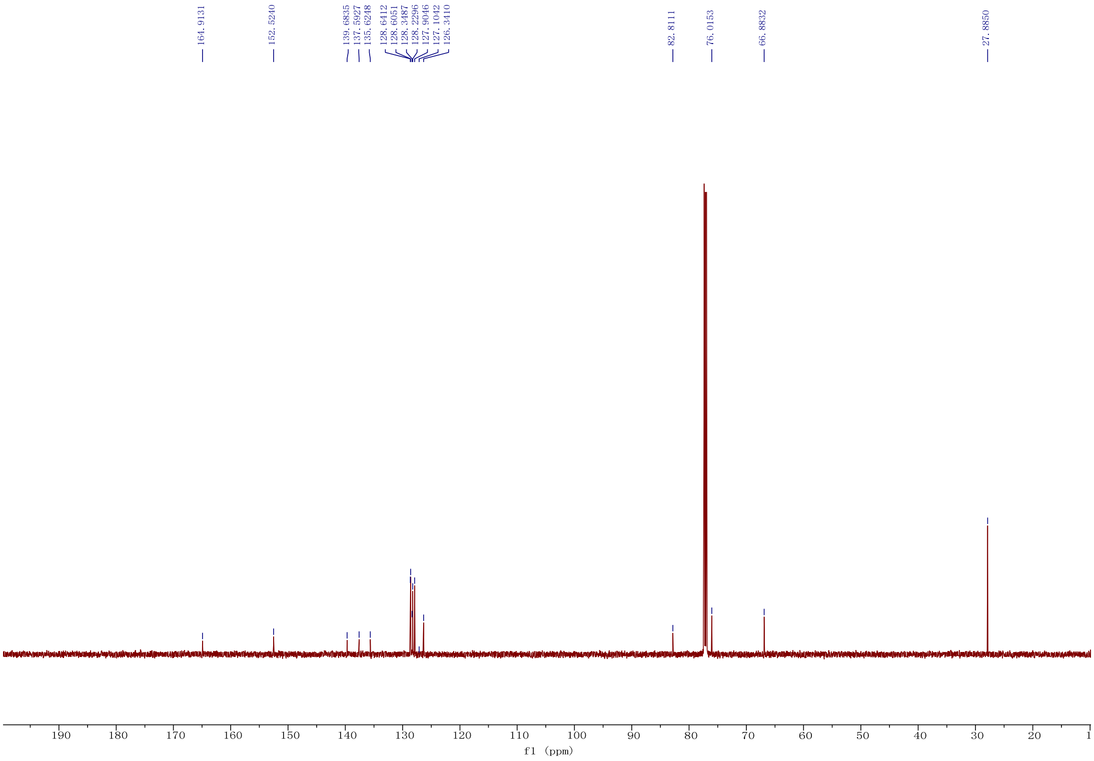
164.9131
152.5240
139.6835
137.5927
135.6248
128.6412
128.6051
128.3487
128.2296
127.9046
127.1042
126.3410
82.8111
76.0153
66.8832
27.8850
f1 (ppm)

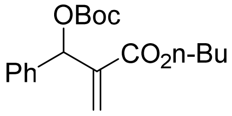
OBoc
Ph
CO2n-Bu

2j

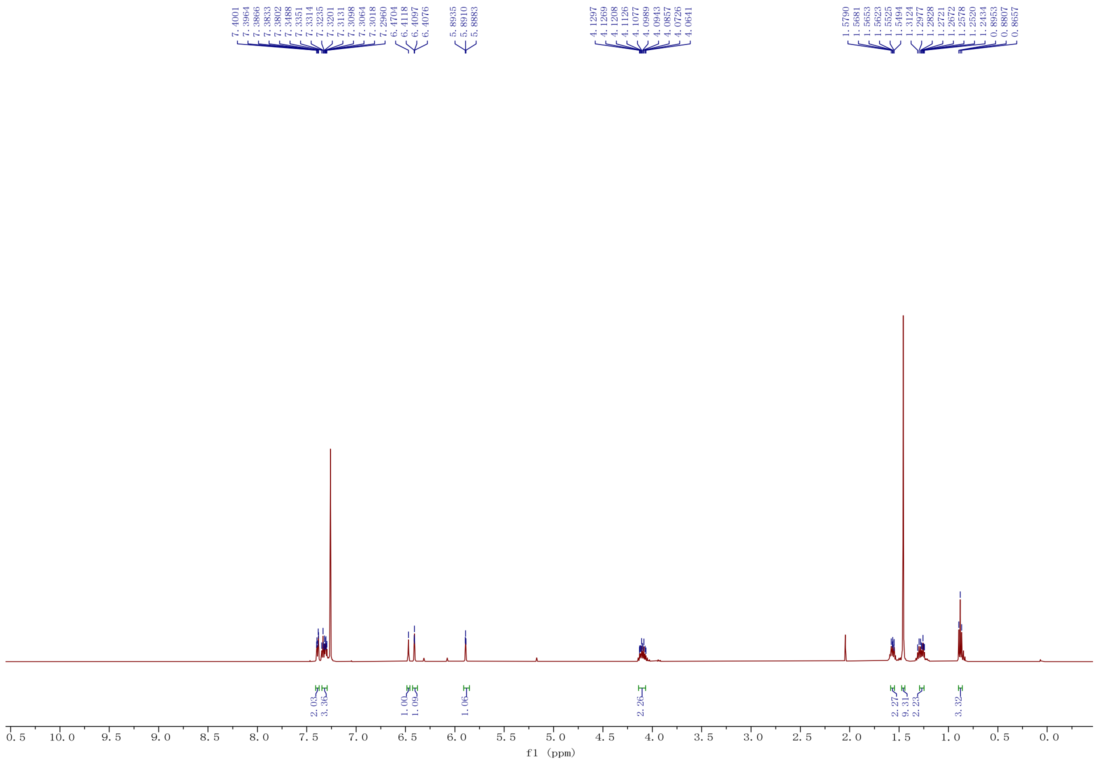
f1 (ppm)

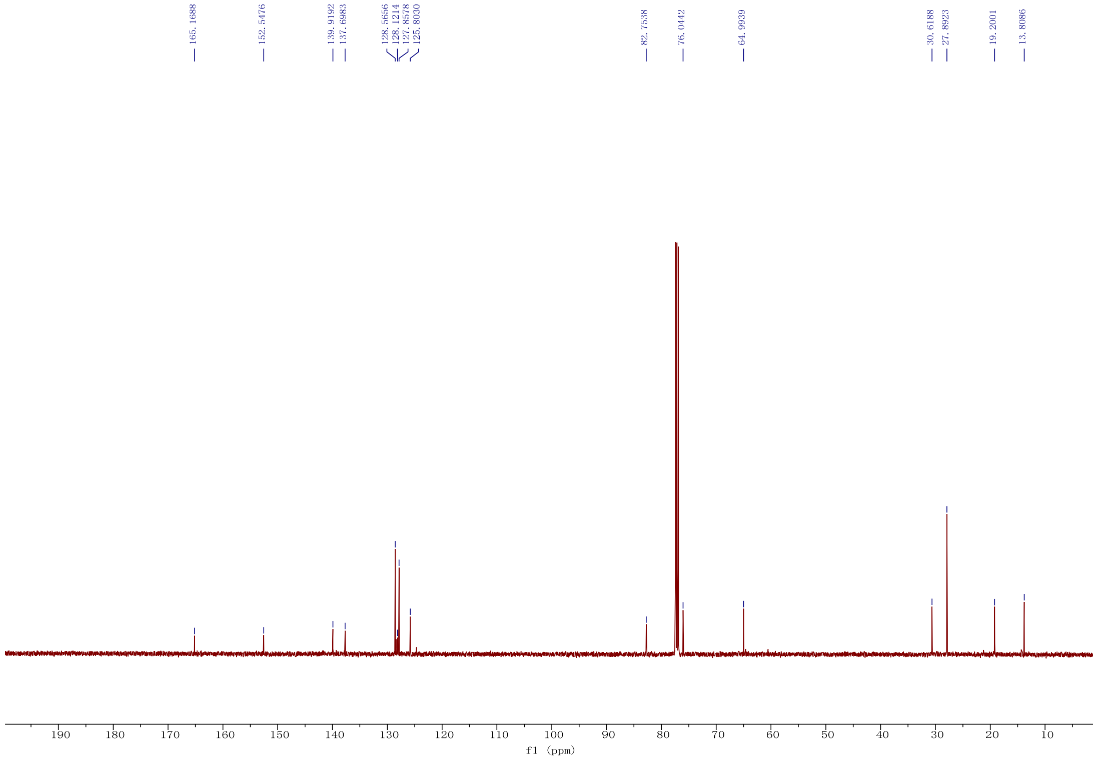
165.1688
152.5476
139.9192
137.6983
128.5656
128.1214
127.8578
125.8030
82.7538
76.0442
64.9939
30.6188
27.8923
19.2001
13.8086
f1 (ppm)

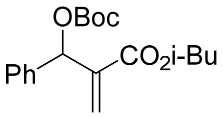
OBoc
Ph
CO2i-Bu

2k

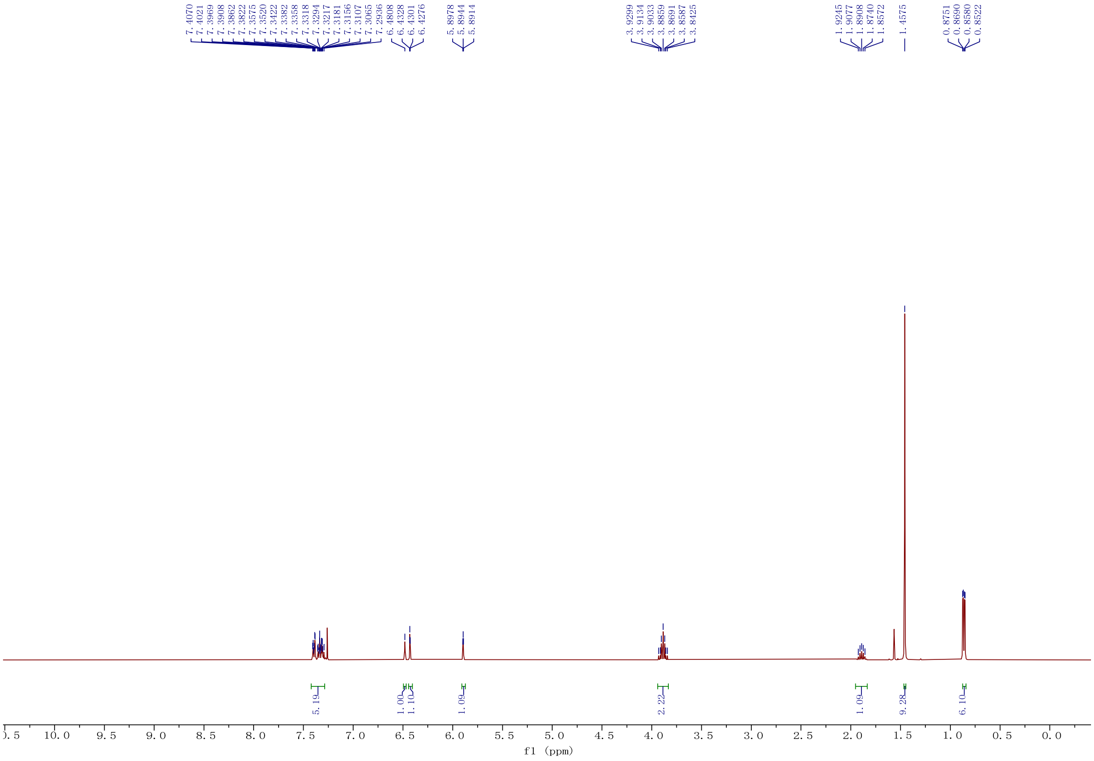
7.4070
7.4021
7.3969
7.3908
7.3862
7.3822
7.3575
7.3520
7.3422
7.3382
7.3358
7.3318
7.3294
7.3217
7.3181
7.3156
7.3107
7.3065
7.2956
6.4808
6.4328
6.4301
6.4276
5.8978
5.8944
5.8914
3.9299
3.9134
3.9033
3.8859
3.8691
3.8587
3.8425
1.9245
1.9077
1.8908
1.8740
1.8572
1.4575
0.8751
0.8690
0.8580
0.8522
5.19
1.00
1.10
1.09
2.22
1.09
9.28
6.10
f1 (ppm)

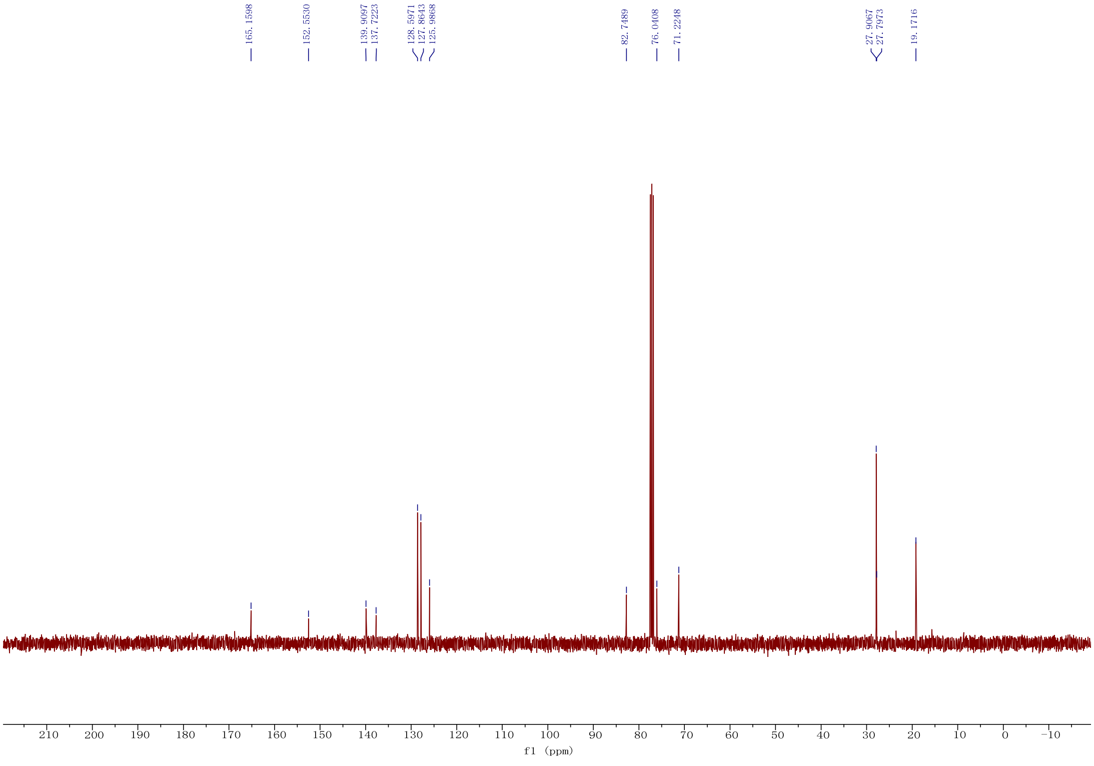
165.1598
152.5530
139.9097
137.7223
128.5971
127.8643
125.9868
82.7489
76.0408
71.2248
27.9067
27.7973
19.1716
f1 (ppm)

3aa

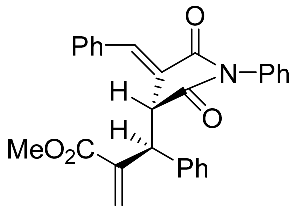
Ph
O
N−Ph
H
H
O
MeO2C
Ph

3aa

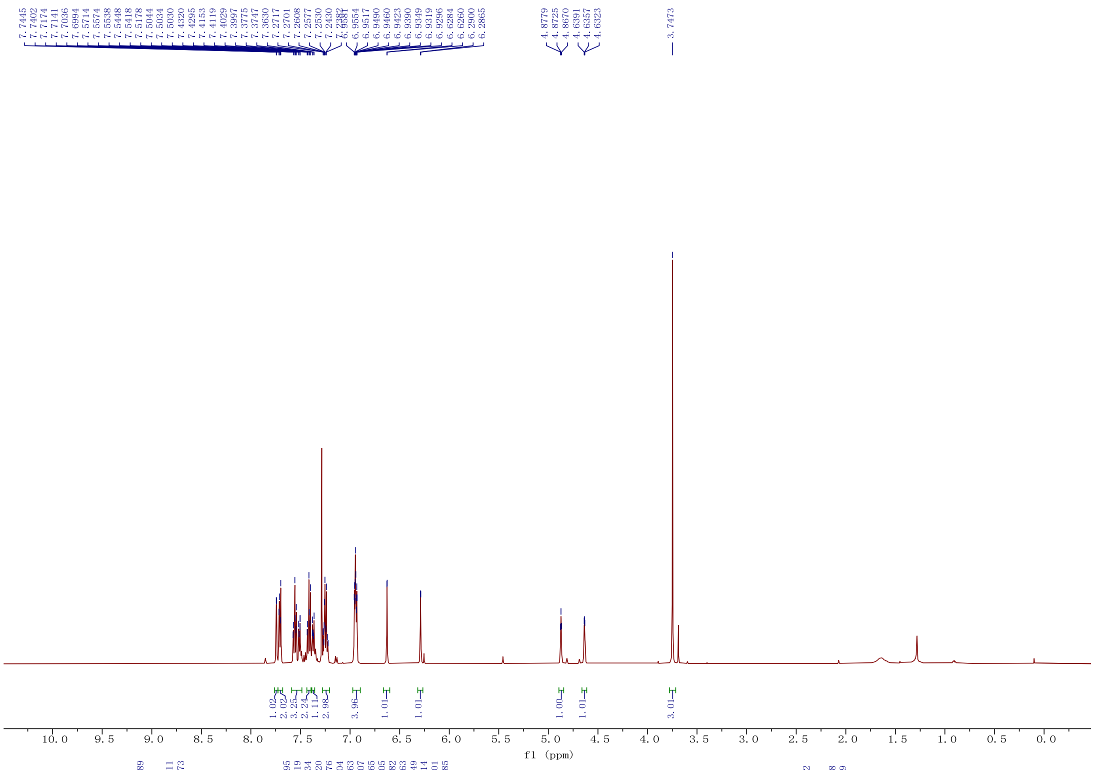
7.7445 7.7402 7.7174 7.7141 7.7036 7.6994 7.5714 7.5574 7.5538 7.5448 7.5418 7.5178 7.5044 7.5034 7.5030 7.4320 7.4295 7.4153 7.4119 7.4029 7.3997 7.3775 7.3747 7.3630 7.2717 7.2701 7.2608 7.2577 7.2530 7.2430 7.2382
6.9581 6.9554 6.9517 6.9490 6.9460 6.9423 6.9390 6.9349 6.9319 6.9296 6.6284 6.6260 6.2900 6.2865
4.8779 4.8725 4.8670 4.6391 4.6357 4.6323
3.7473
1.02 2.02 3.25 2.24 1.11 2.98 3.96 1.01 1.01 1.00 1.01 3.01
10.0 9.5 9.0 8.5 8.0 7.5 7.0 6.5 6.0 5.5 5.0 4.5 4.0 3.5 3.0 2.5 2.0 1.5 1.0 0.5 0.0
f1 (ppm)

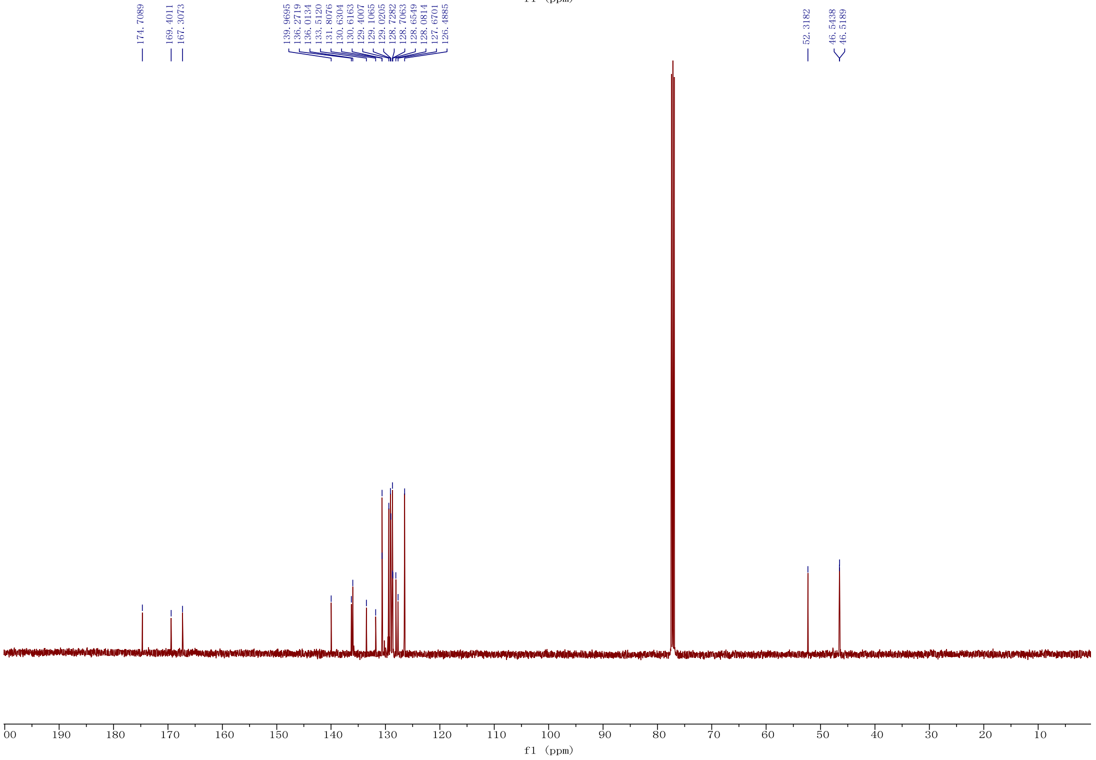
174.7089
169.4011
167.3073
139.9695 136.2719 136.0134 133.5120 131.8076 130.6304 130.6163 129.4007 129.1065 129.0205 128.7282 128.7063 128.6549 128.0814 127.6701 126.4885
52.3182
46.5438 46.5189
00 190 180 170 160 150 140 130 120 110 100 90 80 70 60 50 40 30 20 10
f1 (ppm)

3ba

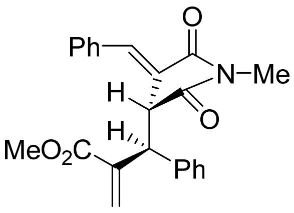

3ba

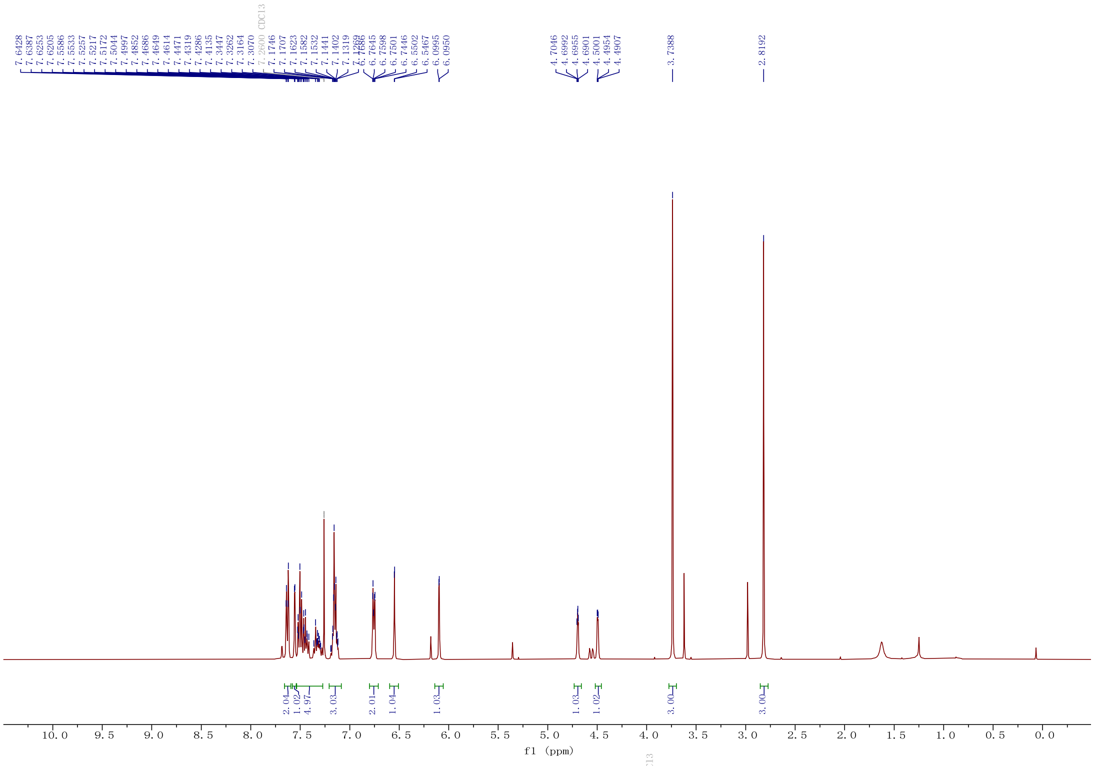

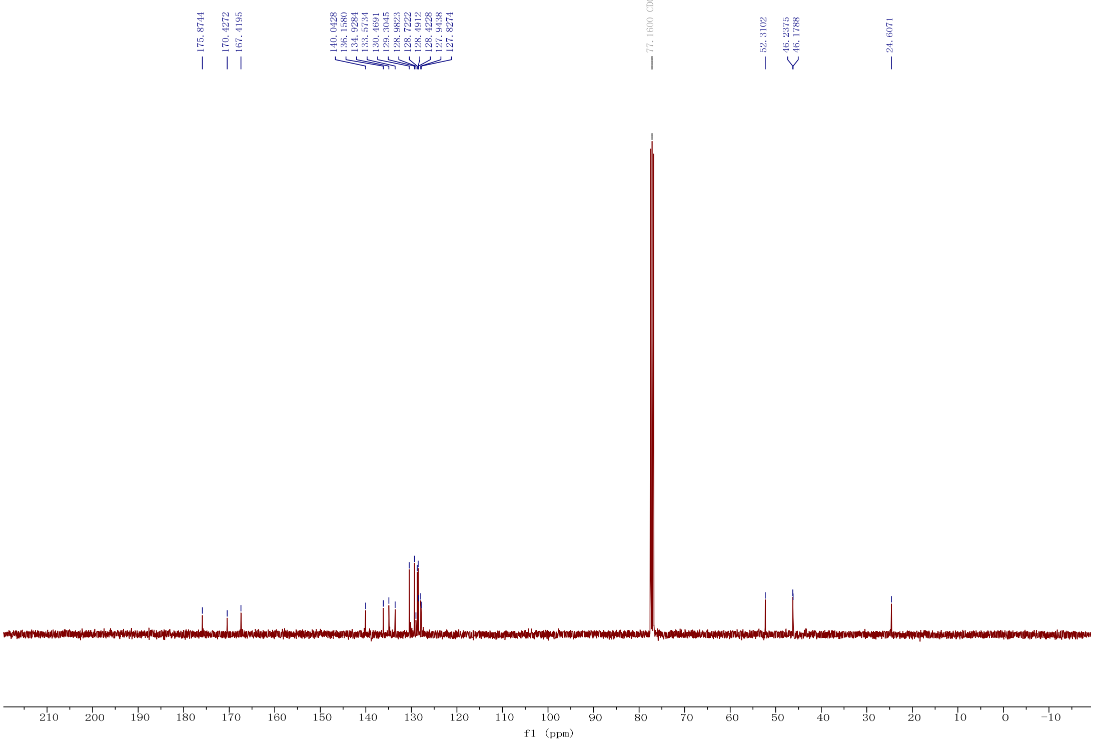

3ca

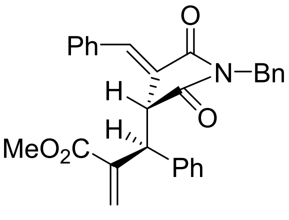

3ca

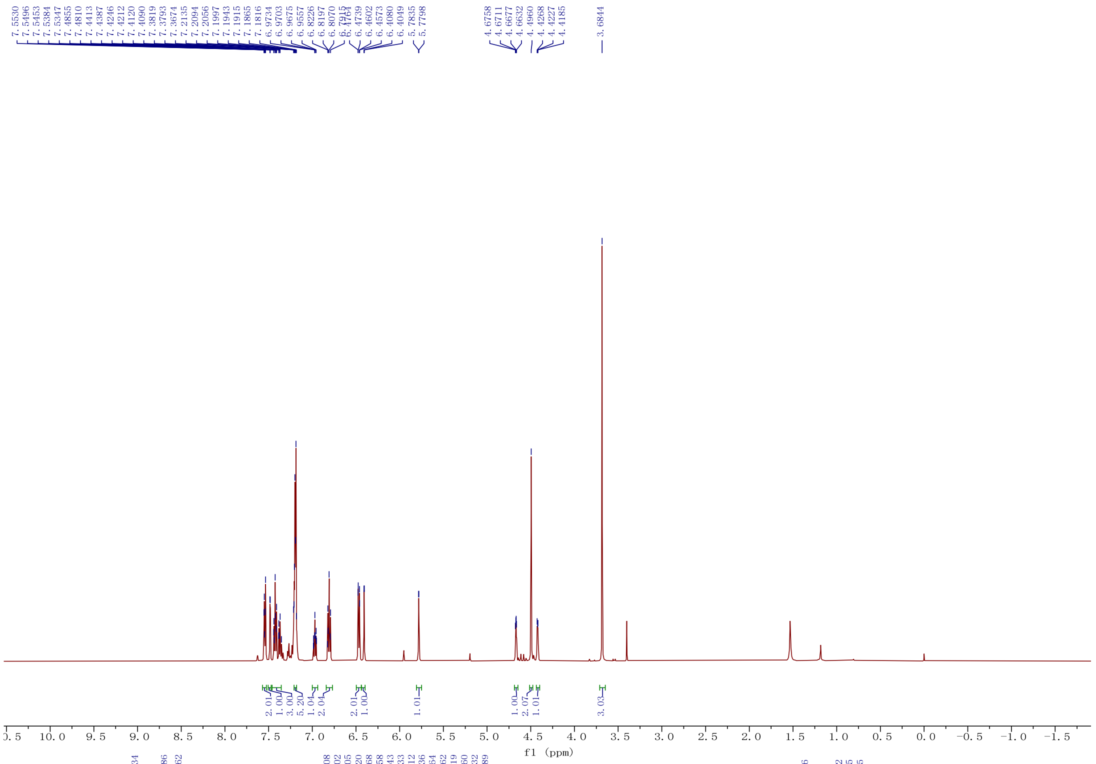

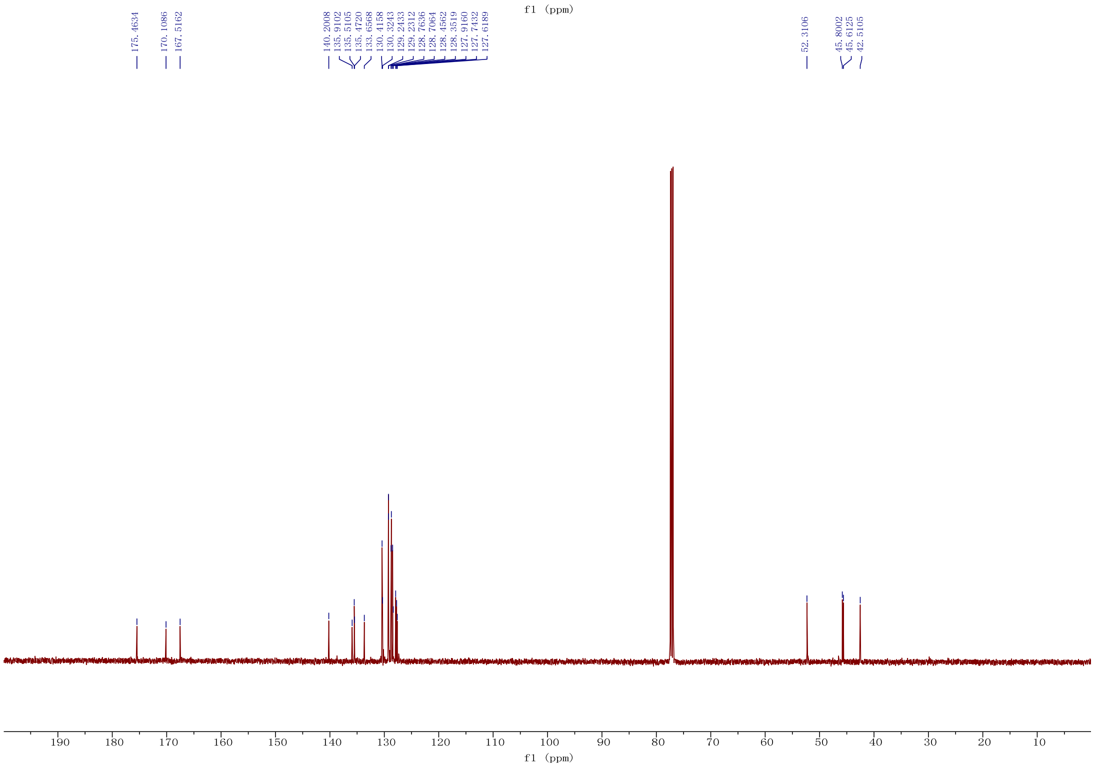

3da

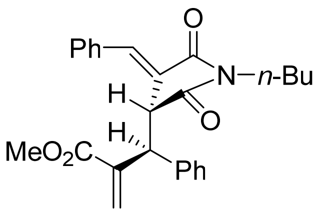

Ph
O
N
n-Bu
H
O
H
MeO2C
Ph

3da

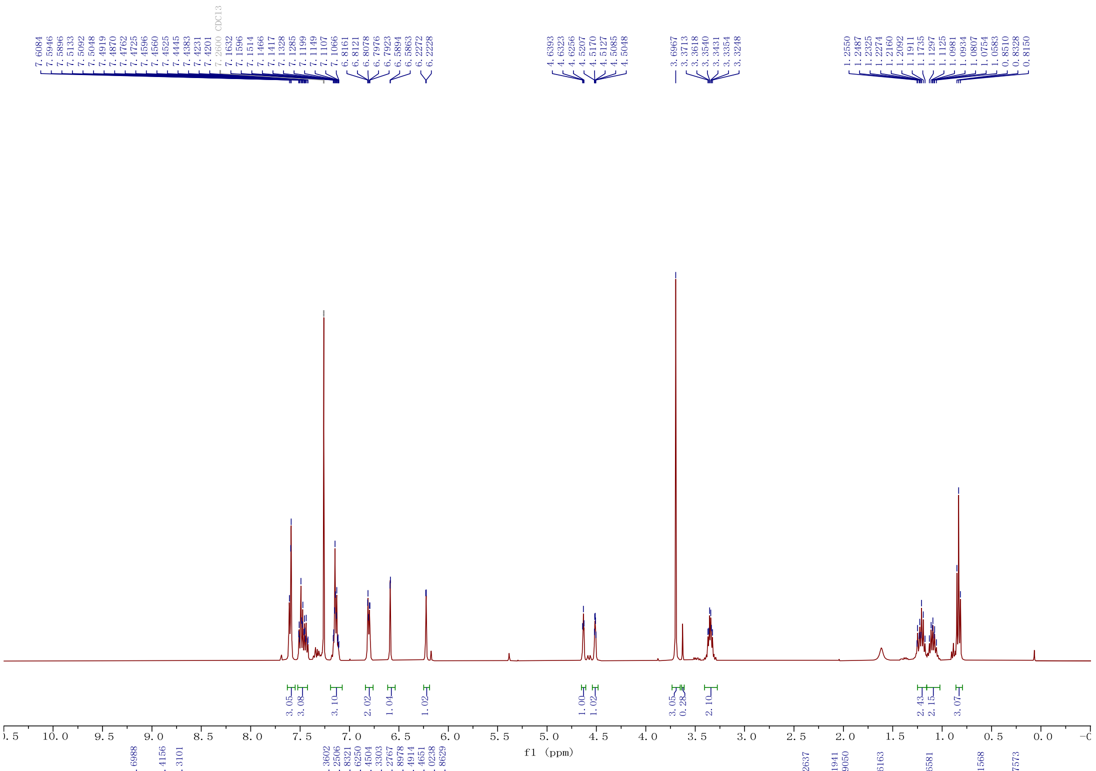

7.6084 7.5946 7.5896 7.5133 7.5092 7.5048 7.4919 7.4870 7.4762 7.4725 7.4596 7.4560 7.4525 7.4445 7.4383 7.4231 7.4201 7.2600 CDCl3 7.1632 7.1596 7.1514 7.1466 7.1417 7.1328 7.1285 7.1199 7.1149 7.1107 7.1066 6.8161 6.8121 6.8078 6.7976 6.7923 6.5894 6.5863 6.2272 6.2228
4.6393 4.6323 4.6256 4.5207 4.5170 4.5127 4.5085 4.5048
3.6967 3.3713 3.3618 3.3540 3.3431 3.3354 3.3248
1.2550 1.2487 1.2325 1.2274 1.2160 1.2092 1.1911 1.1735 1.1297 1.1125 1.0981 1.0934 1.0807 1.0754 1.0583 0.8510 0.8328 0.8150
3.05 3.08 3.10 2.02 1.04 1.02 1.00 1.02 3.05 0.28 2.10 2.43 2.15 3.07
f1 (ppm)

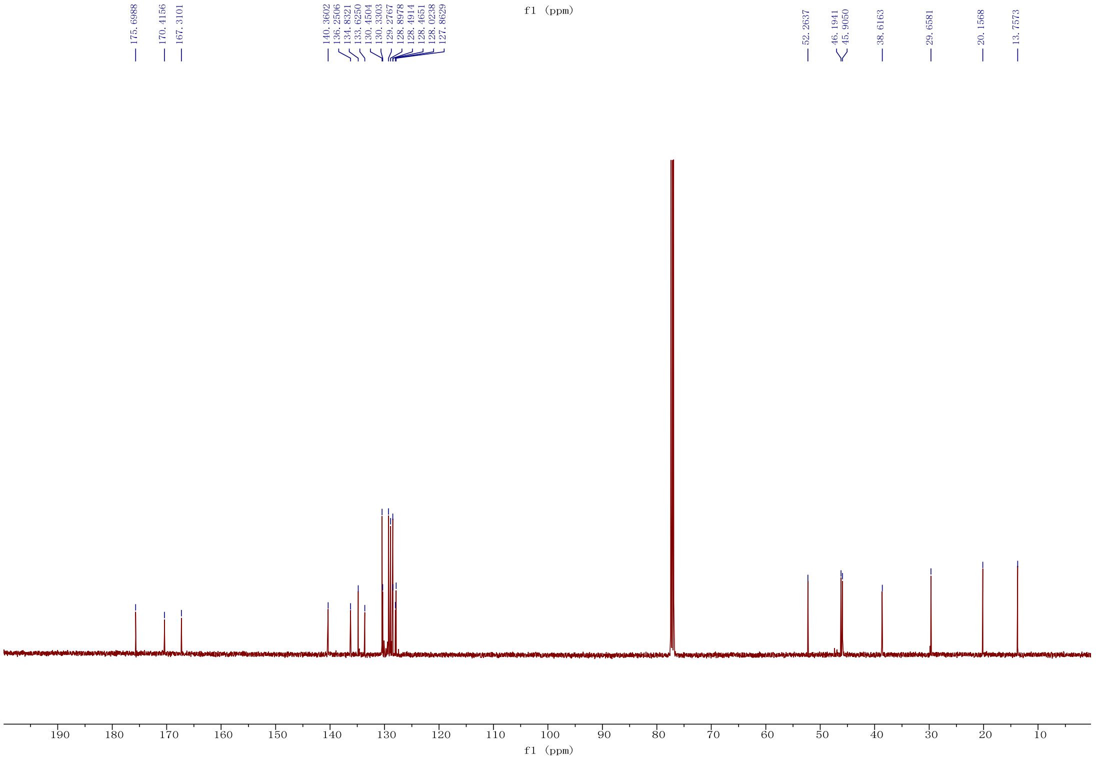

175.6988 170.4156 167.3101
140.3602 136.2506 134.8321 133.6250 130.4504 130.3303 129.2767 128.8978 128.4914 128.4651 128.0238 127.8629
52.2637 46.1941 45.9050 38.6163 29.6581 20.1568 13.7573
f1 (ppm)

3ea

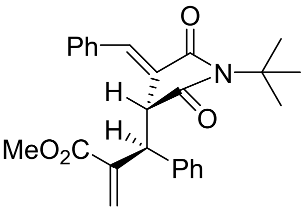
Ph
O
N
H
O
H
MeO2C
Ph

3ea

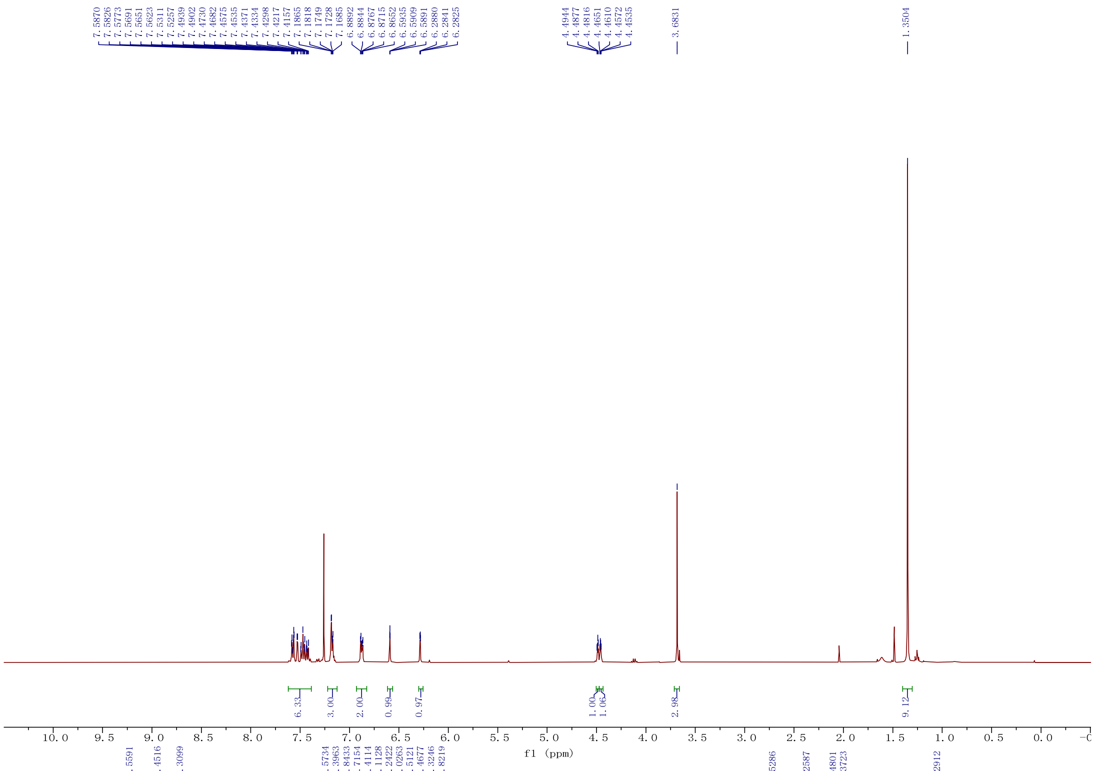
7.5870
7.5826
7.5773
7.5691
7.5651
7.5623
7.5311
7.5257
7.4939
7.4902
7.4730
7.4682
7.4575
7.4535
7.4371
7.4334
7.4298
7.4217
7.4157
7.1865
7.1818
7.1749
7.1728
7.1685
6.8892
6.8844
6.8767
6.8715
6.8652
6.5935
6.5909
6.5891
6.2880
6.2841
6.2825
4.4944
4.4877
4.4816
4.4651
4.4610
4.4572
4.4535
3.6831
1.3504
6.33
3.00
2.00
0.99
0.97
1.00
1.06
2.98
9.12
f1 (ppm)

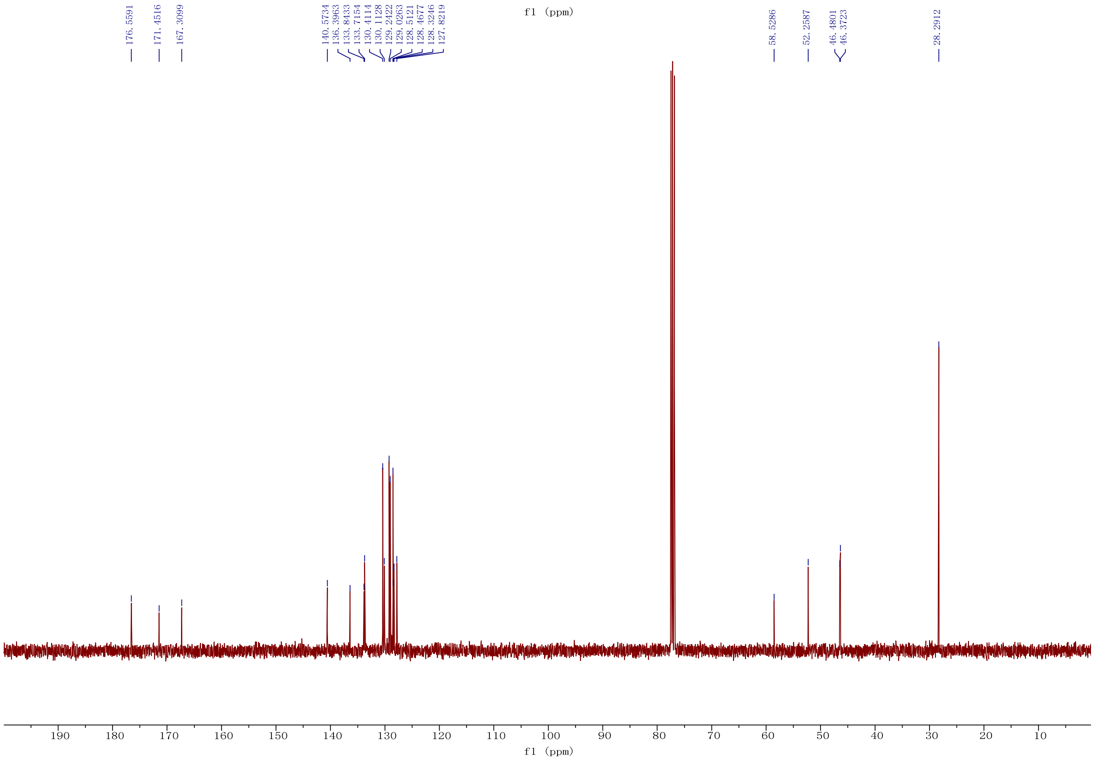
176.5591
171.4516
167.3099
140.5734
136.3963
133.8433
133.7154
130.4114
130.1128
129.2422
129.0263
128.5121
128.4677
128.3246
127.8219
58.5286
52.2587
46.4801
46.3723
28.2912
f1 (ppm)

3fa

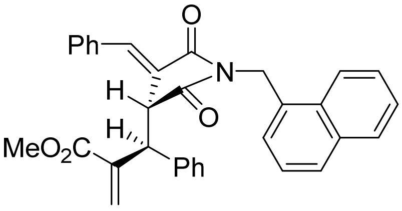
Ph
O
N
H
O
H
MeO2C
Ph

3fa

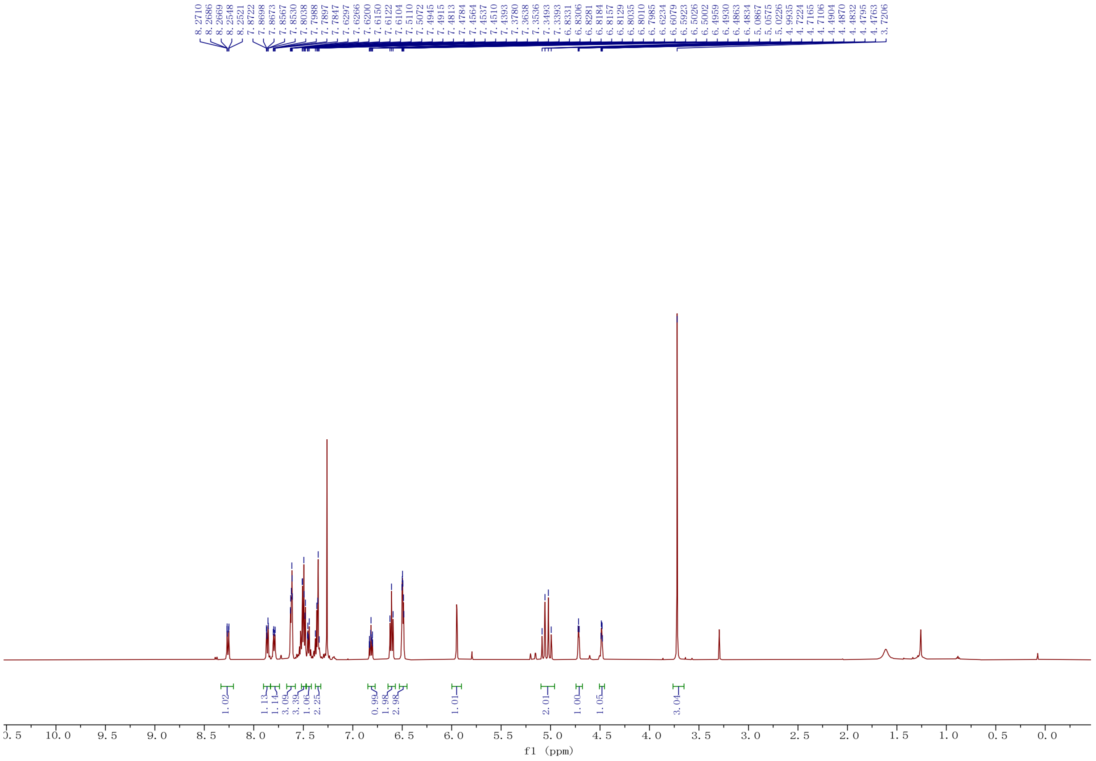
8.2710
8.2686
8.2569
8.2548
8.2521
7.8722
7.8698
7.8673
7.8567
7.8530
7.8038
7.7988
7.7897
7.7847
7.6297
7.6266
7.6200
7.6150
7.6122
7.6104
7.5110
7.5072
7.4945
7.4915
7.4813
7.4784
7.4564
7.4537
7.4510
7.4393
7.3780
7.3638
7.3536
7.3493
7.3393
6.8331
6.8306
6.8281
6.8184
6.8157
6.8129
6.8035
6.8010
6.7985
6.6234
6.6079
6.5923
6.5026
6.5002
6.4959
6.4930
6.4863
6.4834
5.0867
5.0575
5.0226
4.9935
4.7224
4.7165
4.7106
4.4904
4.4870
4.4832
4.4795
4.4763
3.7206
1.02
1.13
1.14
3.09
3.39
1.06
2.23
0.99
1.98
2.98
1.01
2.01
1.00
1.05
3.04
f1 (ppm)

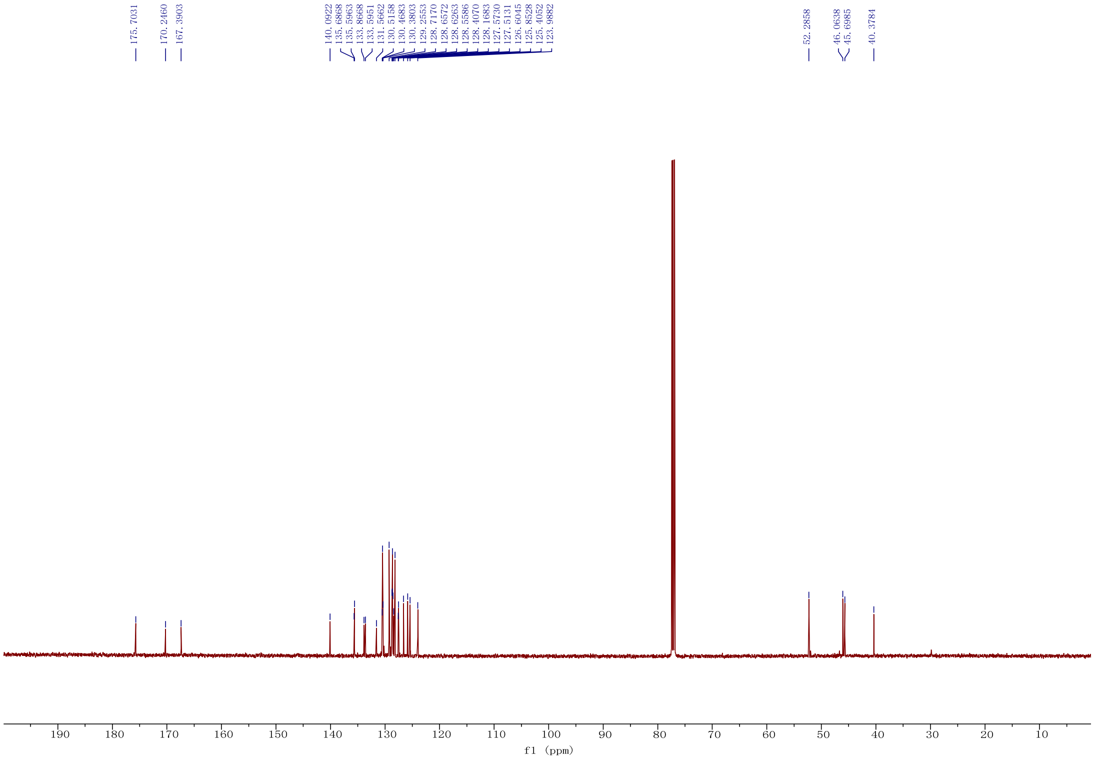
175.7031
170.2460
167.3903
140.0922
135.6868
135.5963
133.8668
133.5951
131.5662
130.5158
130.4683
130.3803
129.2553
128.7170
128.6572
128.6263
128.5586
128.4070
128.1683
127.5730
126.6045
125.8528
125.4052
123.9882
52.2858
46.0638
45.6985
40.3784
f1 (ppm)

3ga

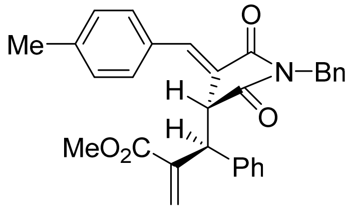
Me
O
N−Bn
H
H,
O
MeO2C
Ph

3ga

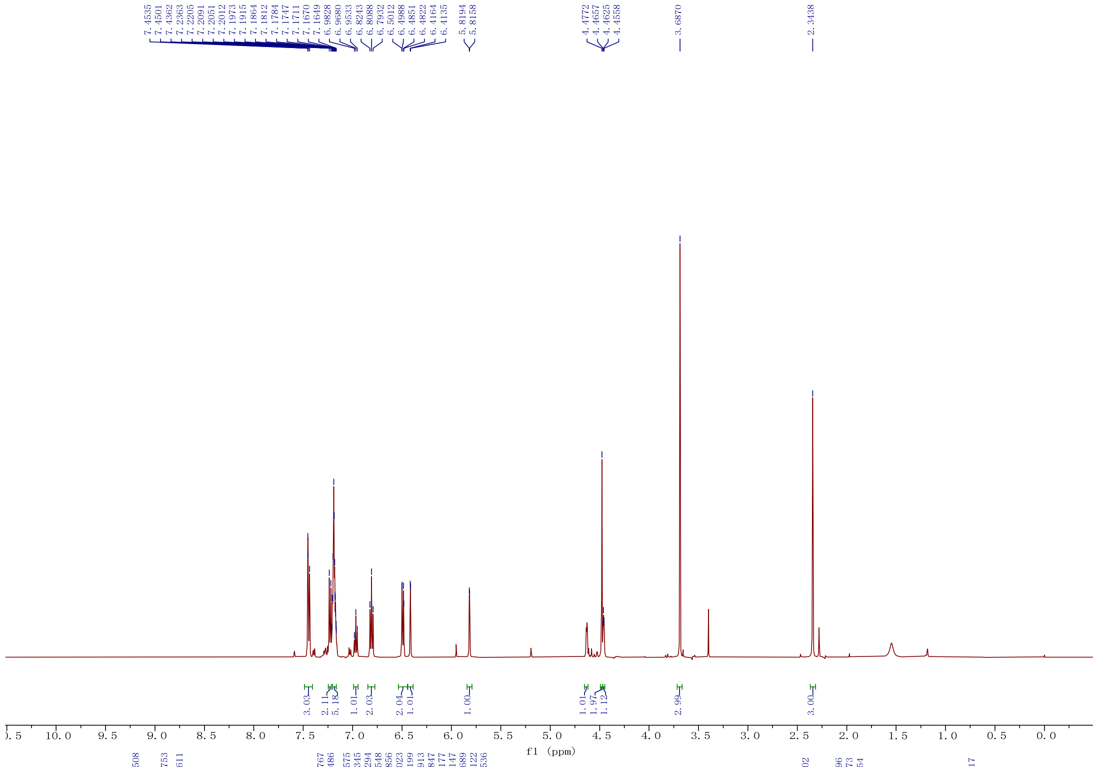
7.4535
7.4501
7.4362
7.2363
7.2205
7.2091
7.2051
7.2012
7.1973
7.1915
7.1864
7.1812
7.1784
7.1747
7.1711
7.1670
7.1649
6.9828
6.9680
6.9533
6.8243
6.8088
6.7932
6.5012
6.4988
6.4851
6.4822
6.4164
6.4135
5.8194
5.8158
4.4772
4.4657
4.4625
4.4558
3.6870
2.3438
3.02
2.11
5.18
1.01
2.03
2.04
1.01
1.00
1.01
1.97
1.12
2.99
3.00
f1 (ppm)

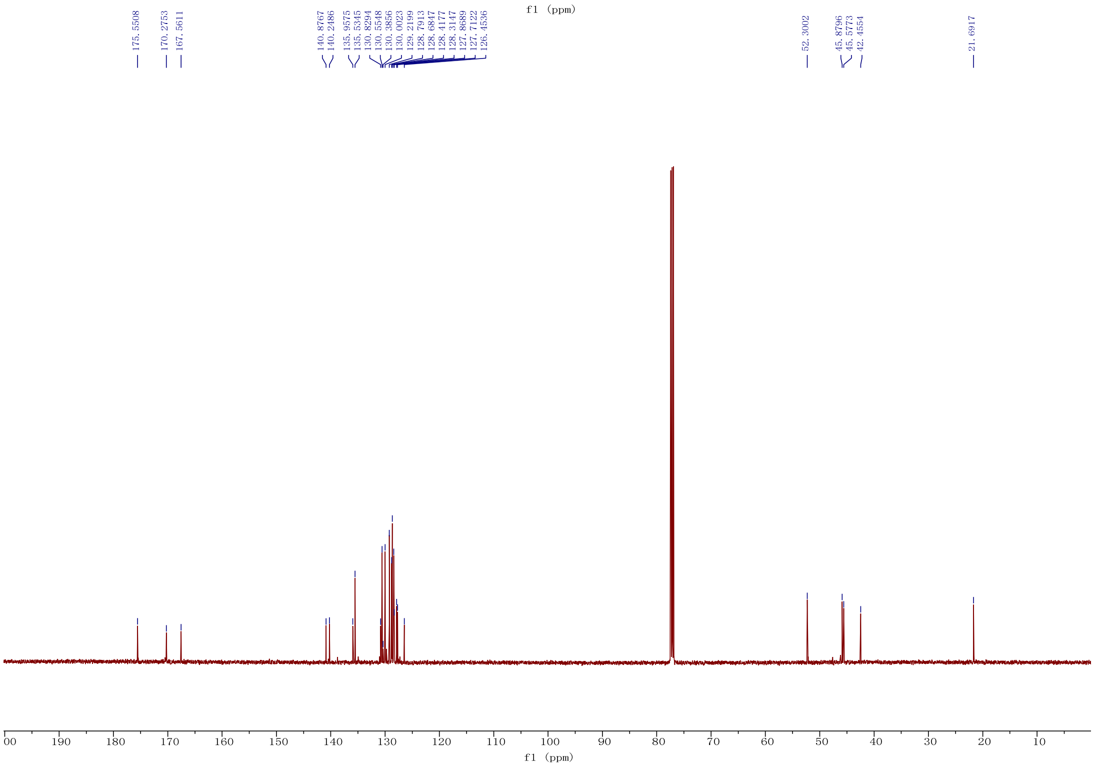
175.5508
170.2753
167.5611
140.8767
140.2486
135.9575
135.5345
130.8294
130.3856
130.5548
130.0023
129.2199
128.7913
128.6847
128.4177
128.3147
127.8689
127.7122
126.4536
52.3002
45.8796
45.5773
42.4554
21.6917
f1 (ppm)

3ha

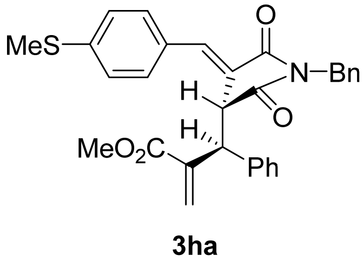

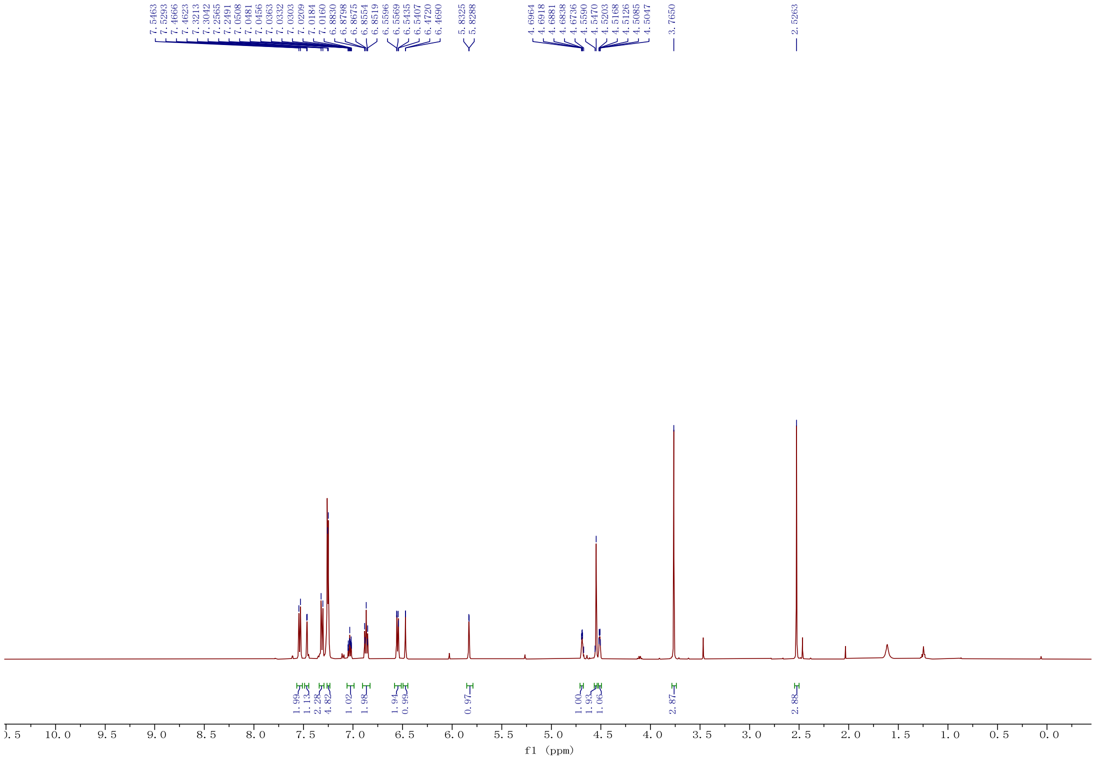

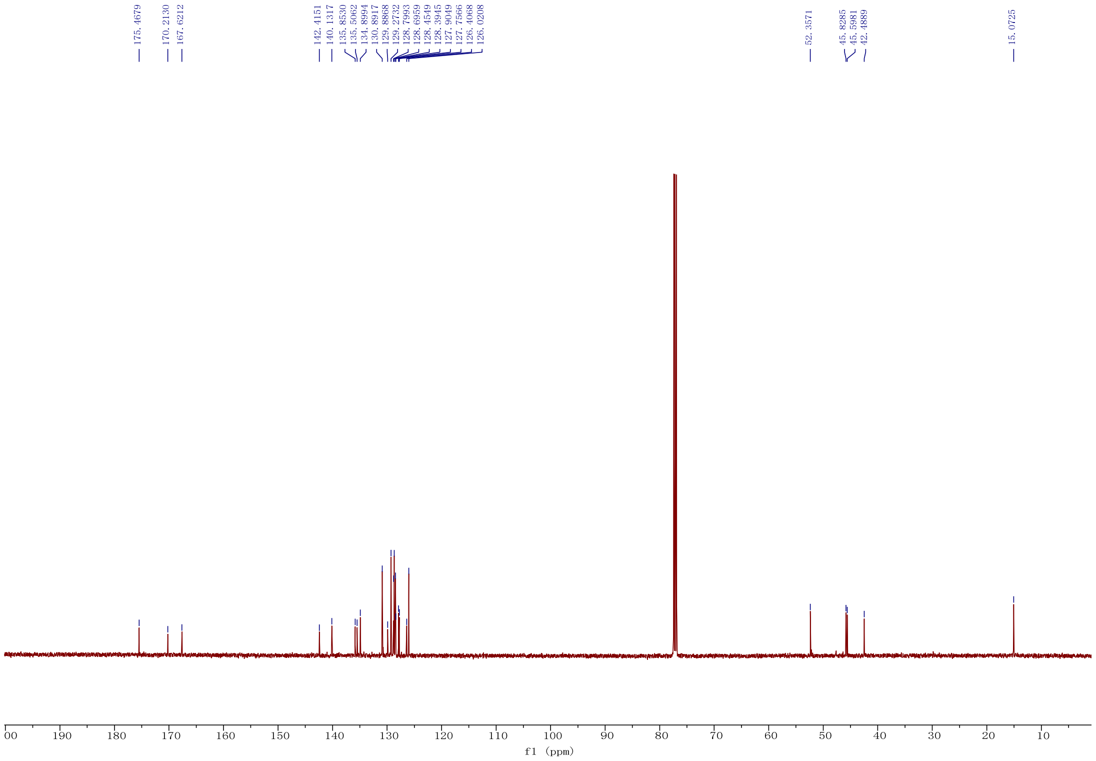

3ia

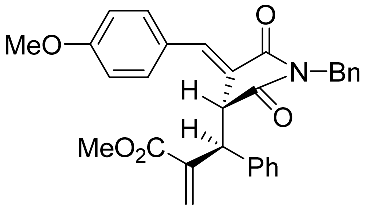

3ia

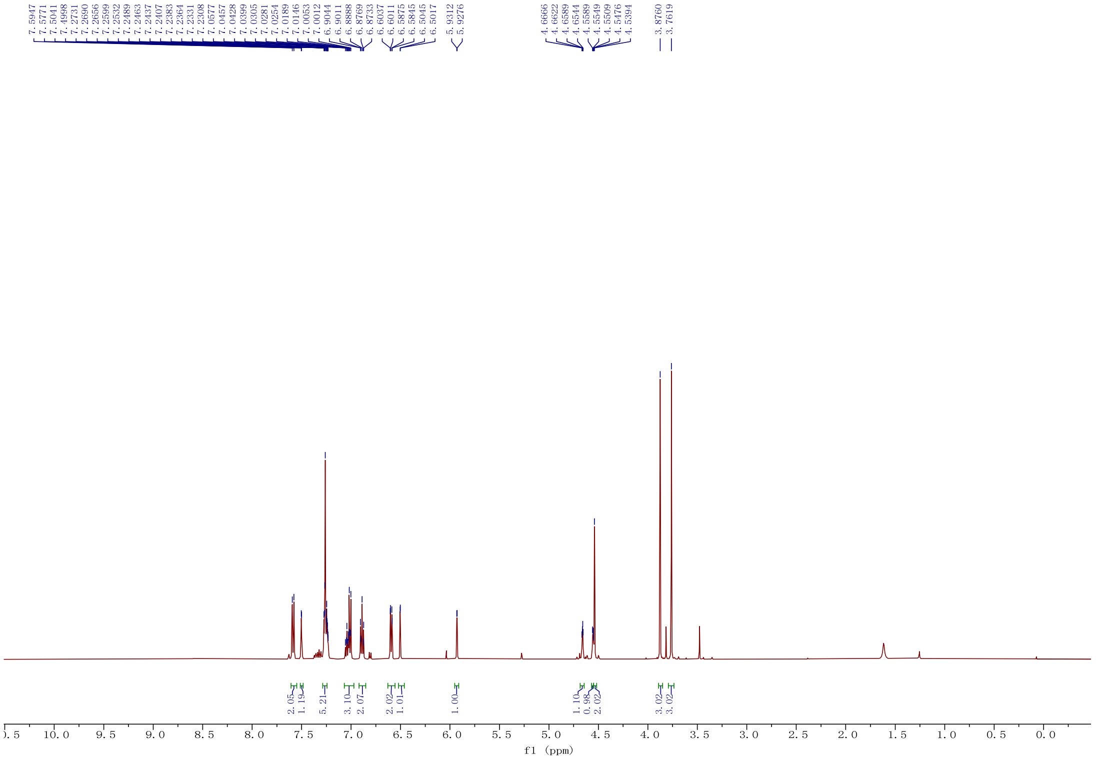

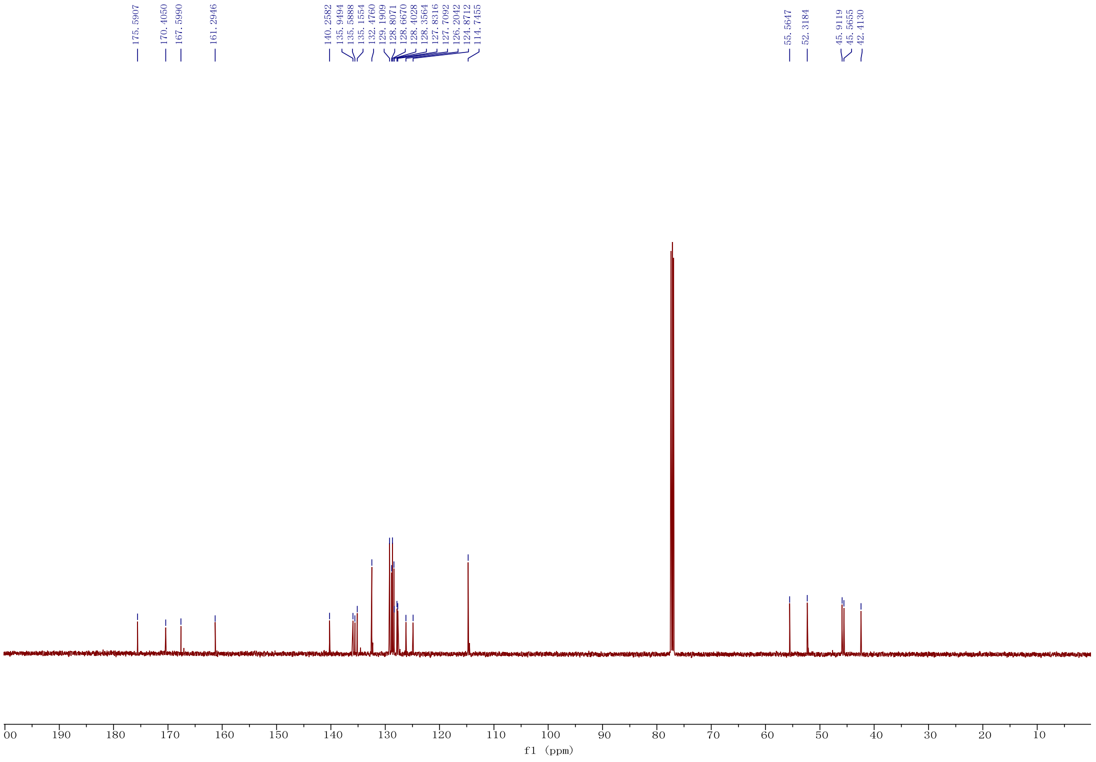

3ja

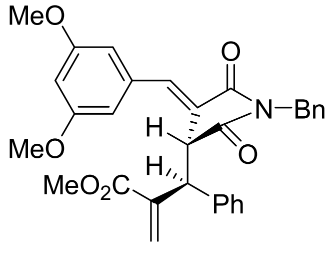

MeO
MeO
O
O
N−Bn
H
H
MeO2C
Ph

3ja

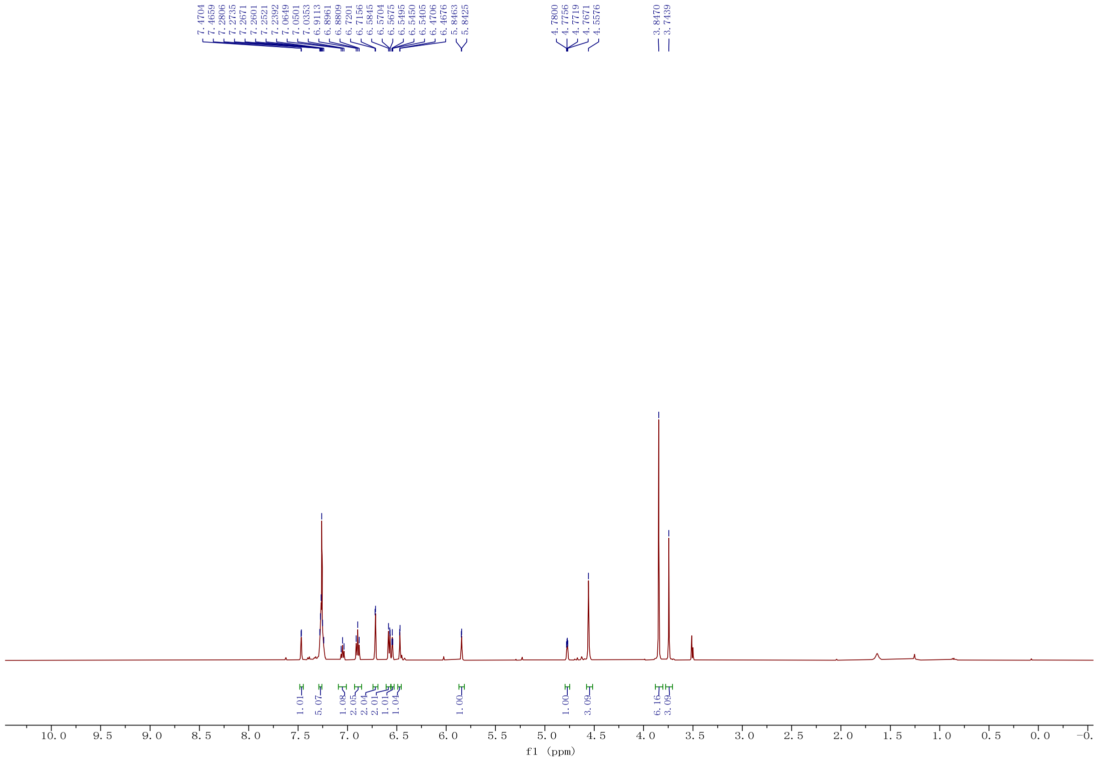

7.4704
7.4659
7.2806
7.2735
7.2671
7.2601
7.2521
7.2392
7.0649
7.0501
7.0353
6.9113
6.8961
6.8809
6.7201
6.7156
6.5845
6.5704
6.5675
6.5495
6.5450
6.5405
6.4706
6.4676
5.8463
5.8425
4.7800
4.7756
4.7719
4.7671
4.5576
3.8470
3.7439
1.01
5.07
1.08
2.05
2.04
2.01
1.01
1.04
1.00
1.00
3.09
6.16
3.09
f1 (ppm)

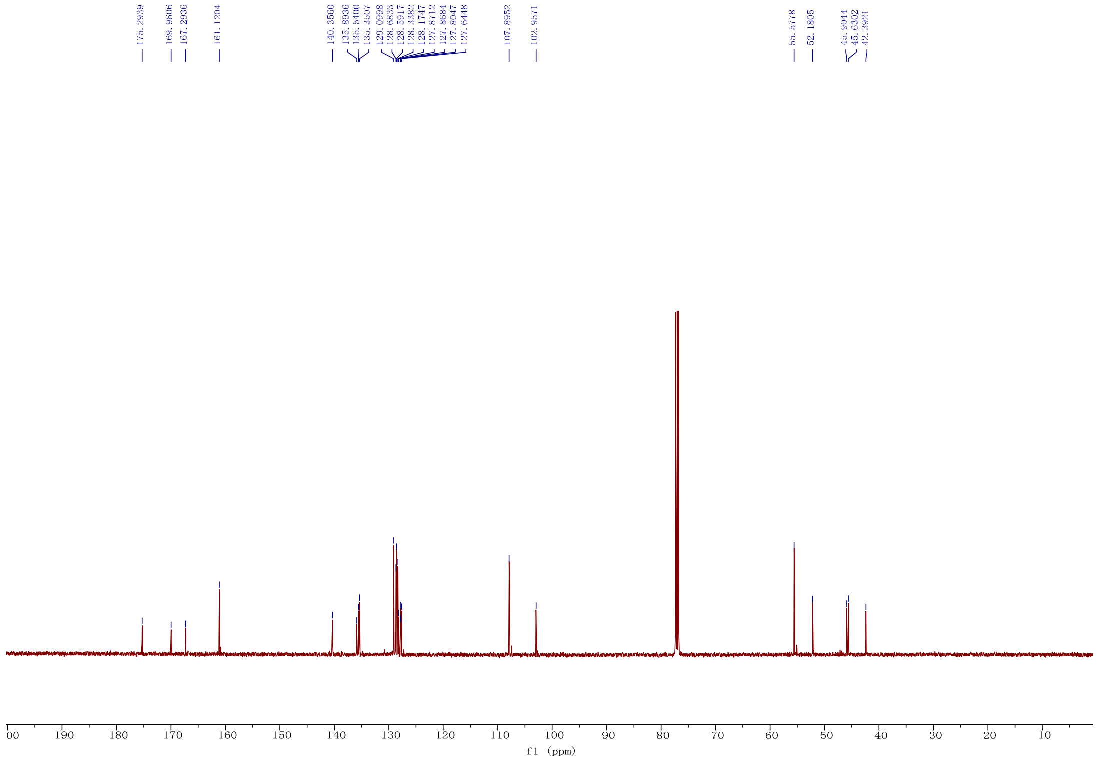

175.2939
169.9606
167.2936
161.1204
140.3560
135.8936
135.5400
135.3507
129.0998
128.6833
128.5917
128.3382
128.1747
127.8712
127.8684
127.8047
127.6448
107.8952
102.9571
55.5778
52.1805
45.9044
45.6302
42.3921
f1 (ppm)

3ka

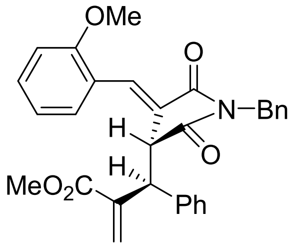

OMe
O
N−Bn
H
O
H
MeO2C
Ph

3ka

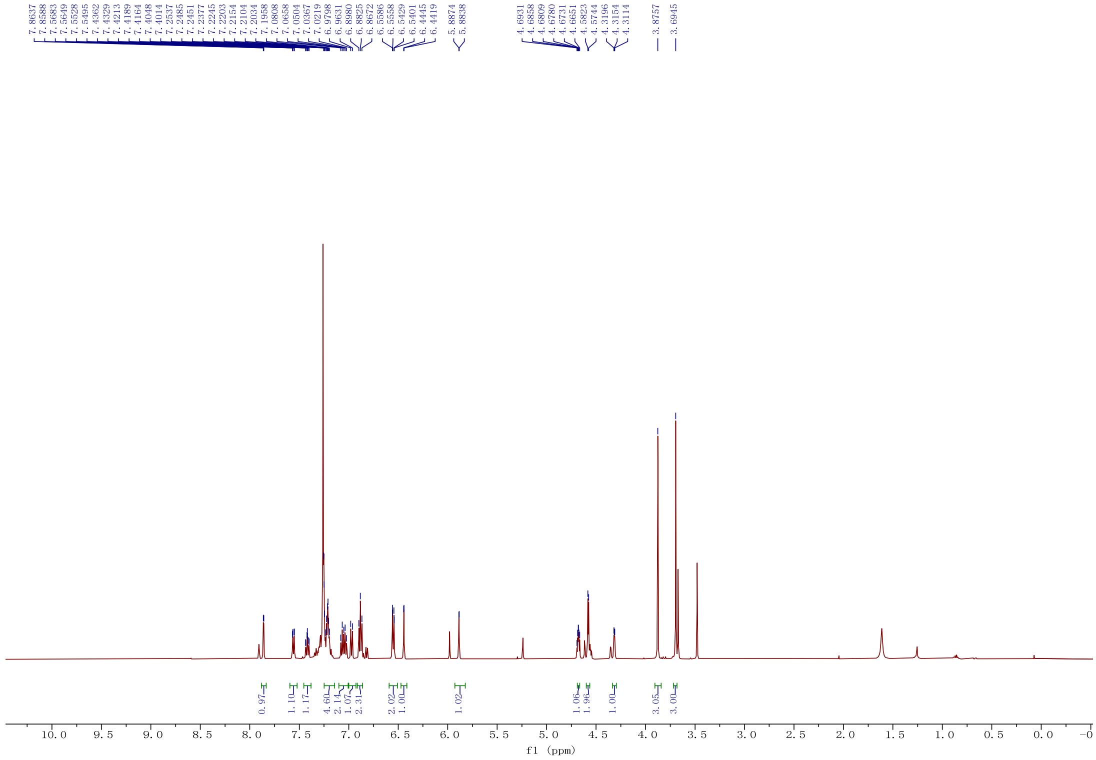

7.8637
7.8588
7.5683
7.5649
7.5528
7.5495
7.4362
7.4329
7.4213
7.4189
7.4164
7.4048
7.4014
7.2537
7.2485
7.2451
7.2377
7.2245
7.2203
7.2154
7.2104
7.2034
7.1958
7.0808
7.0658
7.0504
7.0367
7.0219
6.9798
6.9631
6.8980
6.8825
6.8672
6.5586
6.5558
6.5429
6.5401
6.4445
6.4419
5.8874
5.8838
4.6931
4.6858
4.6809
4.6780
4.6731
4.6651
4.5823
4.5744
4.3196
4.3154
4.3114
3.8757
3.6945
0.97
1.10
1.17
4.60
2.14
1.07
2.31
2.02
1.00
1.02
1.06
1.96
1.00
3.05
3.00
10.0 9.5 9.0 8.5 8.0 7.5 7.0 6.5 6.0 5.5 5.0 4.5 4.0 3.5 3.0 2.5 2.0 1.5 1.0 0.5 0.0
f1 (ppm)

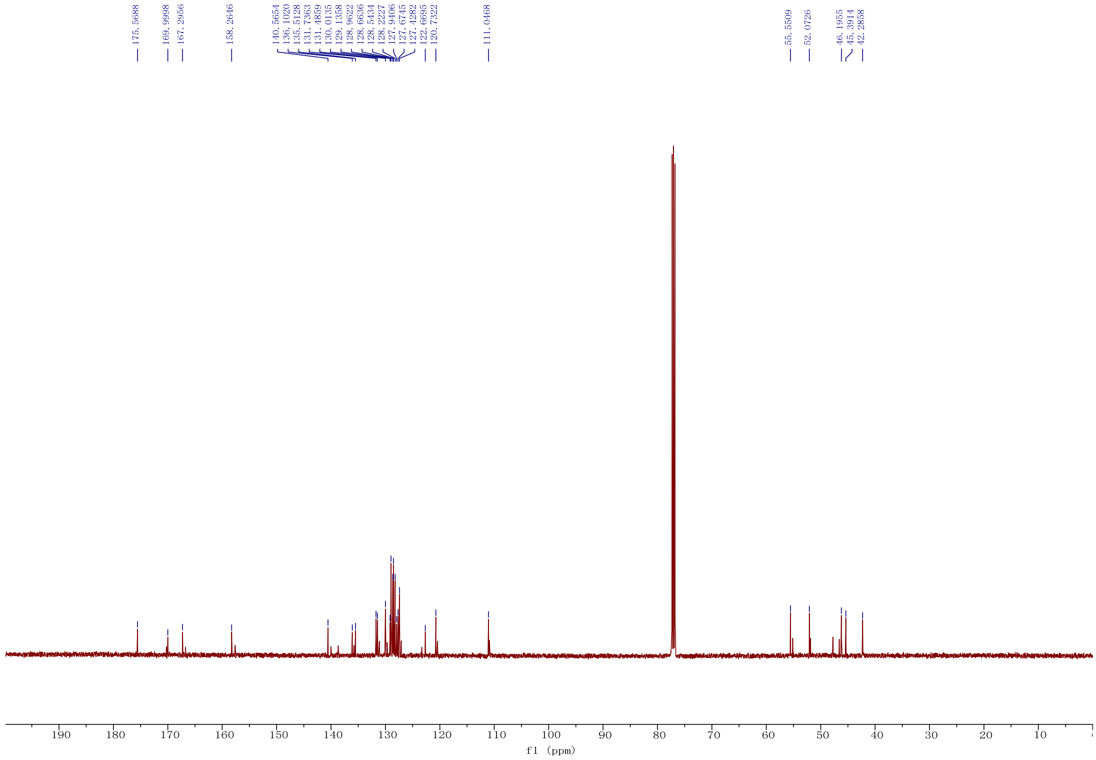

175.5688
169.9998
167.2956
158.2646
140.5654
136.1020
135.5128
131.7363
131.4859
130.0135
129.1358
128.9622
128.6636
128.5434
128.2227
127.9406
127.6745
127.4282
122.4695
120.7322
111.0468
55.5509
52.0726
46.1955
45.3914
42.2858
190 180 170 160 150 140 130 120 110 100 90 80 70 60 50 40 30 20 10
f1 (ppm)

3la

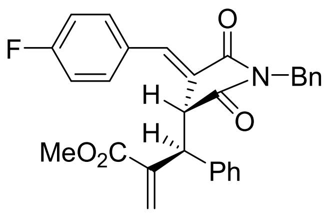

F
O
N–Bn
H
H
O
MeO2C
Ph

3la

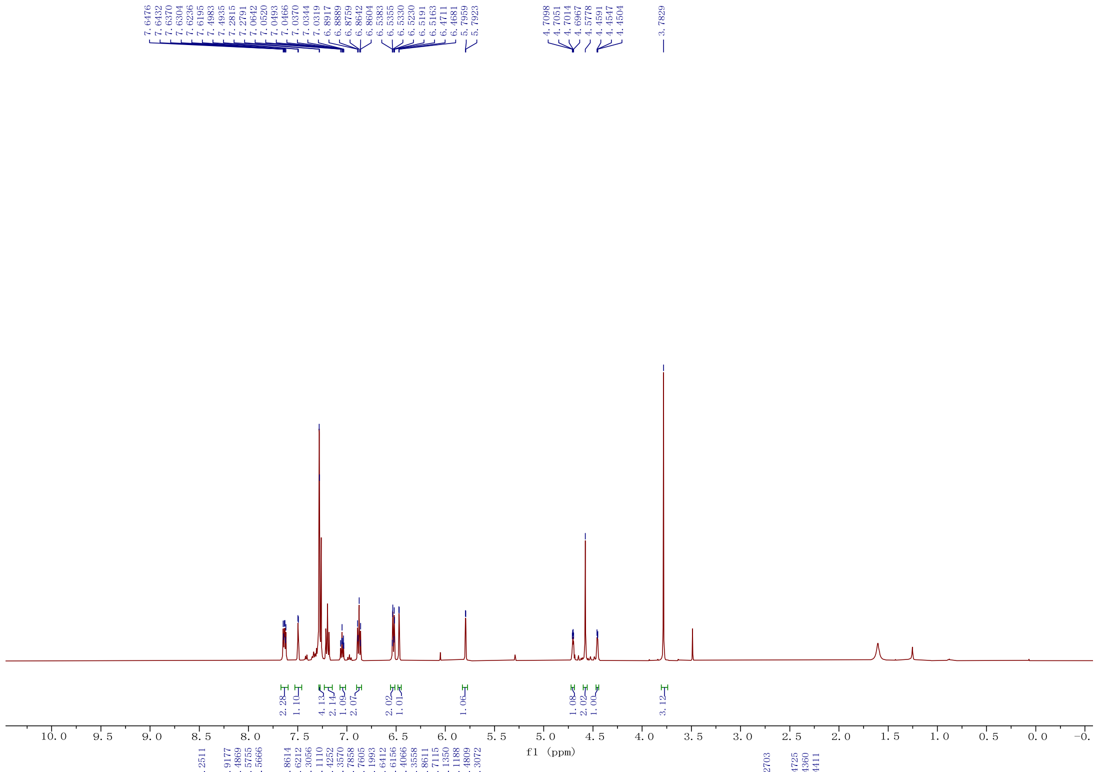

7.6476
7.6432
7.6370
7.6304
7.6236
7.6195
7.4983
7.4935
7.2815
7.2791
7.0642
7.0520
7.0493
7.0466
7.0370
7.0344
7.0319
6.8917
6.8889
6.8759
6.8642
6.8604
6.5383
6.5355
6.5330
6.5230
6.5191
6.5163
6.4711
6.4681
5.7959
5.7923
4.7098
4.7051
4.7014
4.6967
4.5778
4.4591
4.4547
4.4504
3.7829
2.28
1.10
4.13
2.14
1.04
2.07
2.02
1.01
1.06
1.08
2.02
1.00
3.12
f1 (ppm)

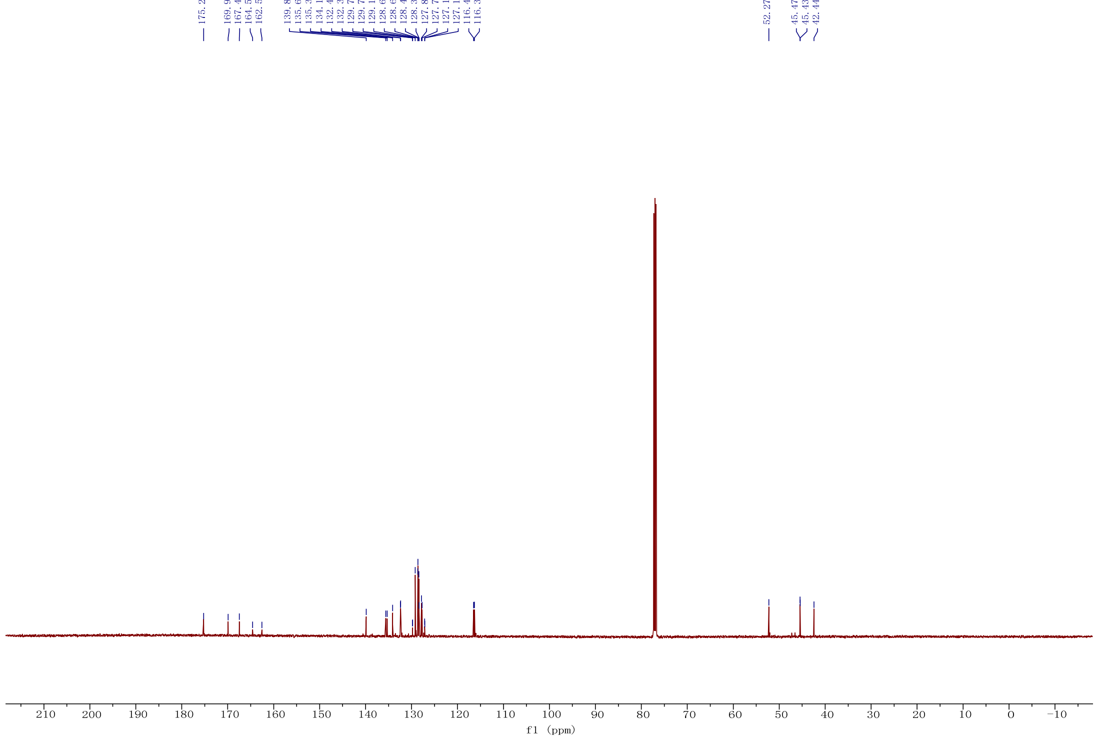

175.2511
169.9177
167.4869
164.5755
162.5666
139.8614
135.6212
135.3056
134.1110
132.4252
132.3570
129.7858
129.7605
129.1993
128.6412
128.6156
128.4066
128.3558
127.8611
127.7115
127.1350
127.1188
116.4809
116.3072
52.2703
45.4725
45.4360
42.4411
f1 (ppm)

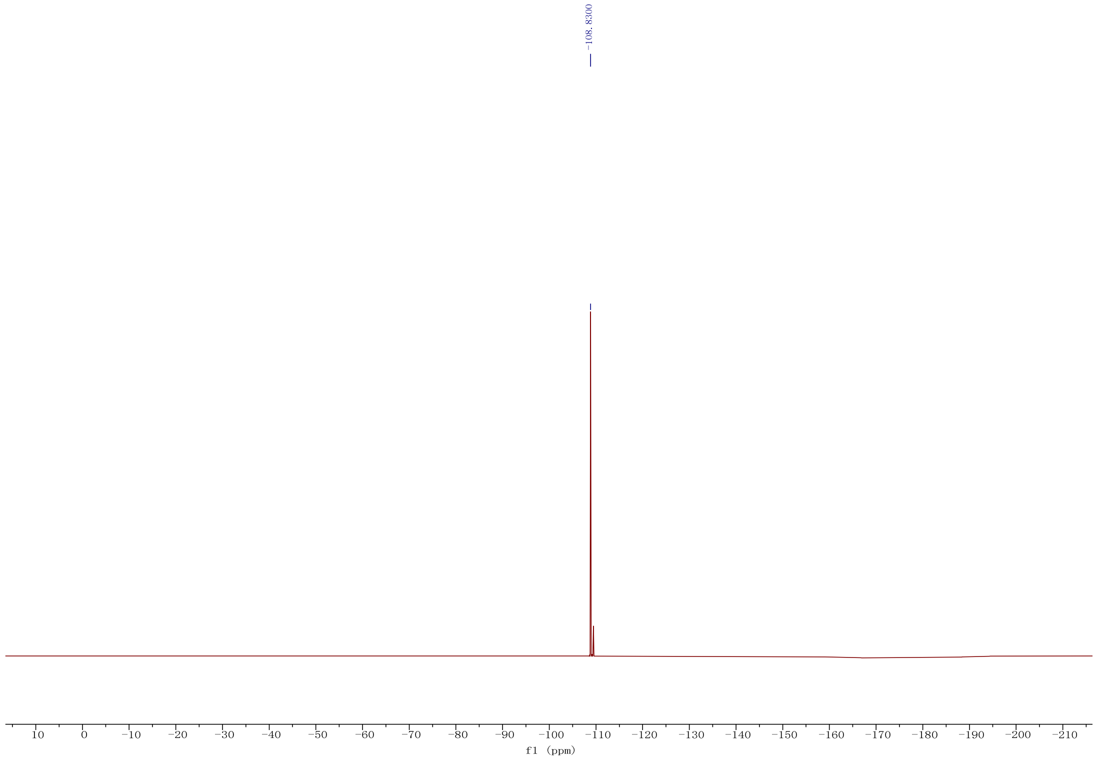

-108.8300
10
0
-10
-20
-30
-40
-50
-60
-70
-80
-90
-100
-110
-120
-130
-140
-150
-160
-170
-180
-190
-200
-210
f1 (ppm)

3ma

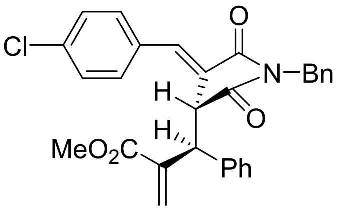

Cl
O
N−Bn
H
O
H
MeO2C
Ph

3ma

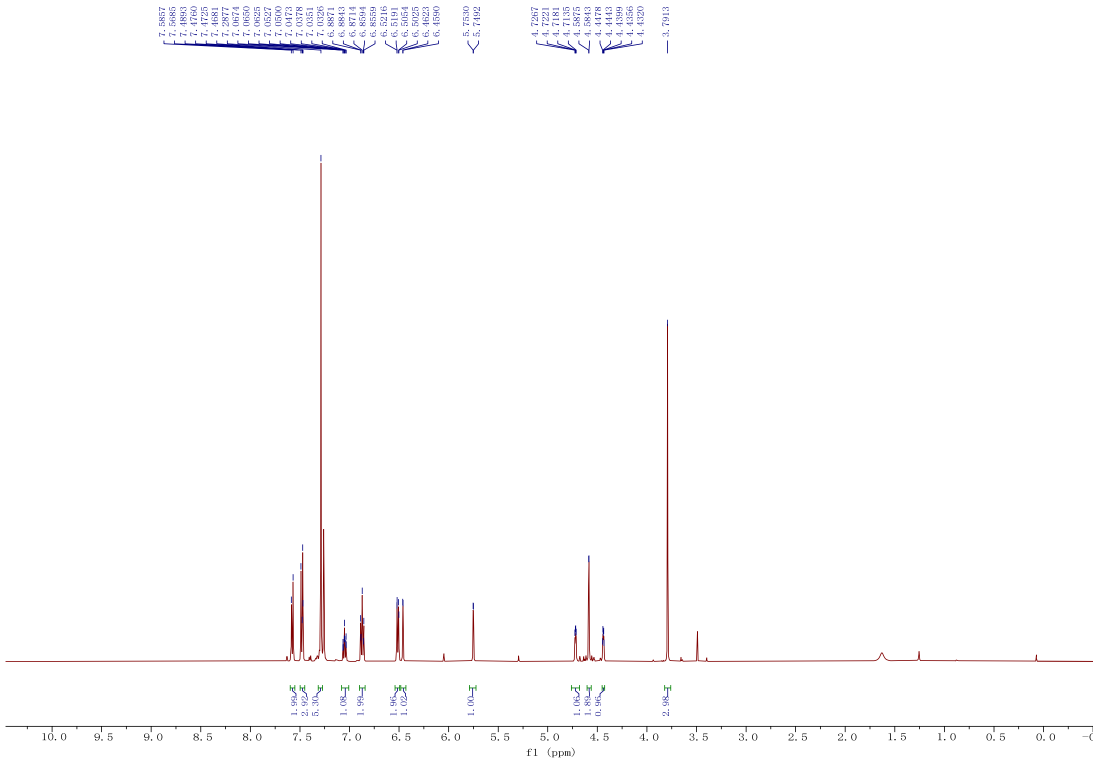

7.5857 7.5685 7.4893 7.4760 7.4725 7.4681 7.2877 7.0674 7.0650 7.0625 7.0527 7.0500 7.0473 7.0378 7.0351 7.0326 6.8871 6.8843 6.8714 6.8594 6.8559 6.5216 6.5191 6.5054 6.5025 6.4623 6.4590 5.7530 5.7492 4.7267 4.7221 4.7181 4.7135 4.5875 4.5843 4.4478 4.4443 4.4399 4.4356 4.4320 3.7913
1.99 2.92 5.30 1.08 1.99 1.96 1.02 1.00 1.06 1.89 0.96 2.98
10.0 9.5 9.0 8.5 8.0 7.5 7.0 6.5 6.0 5.5 5.0 4.5 4.0 3.5 3.0 2.5 2.0 1.5 1.0 0.5 0.0
f1 (ppm)

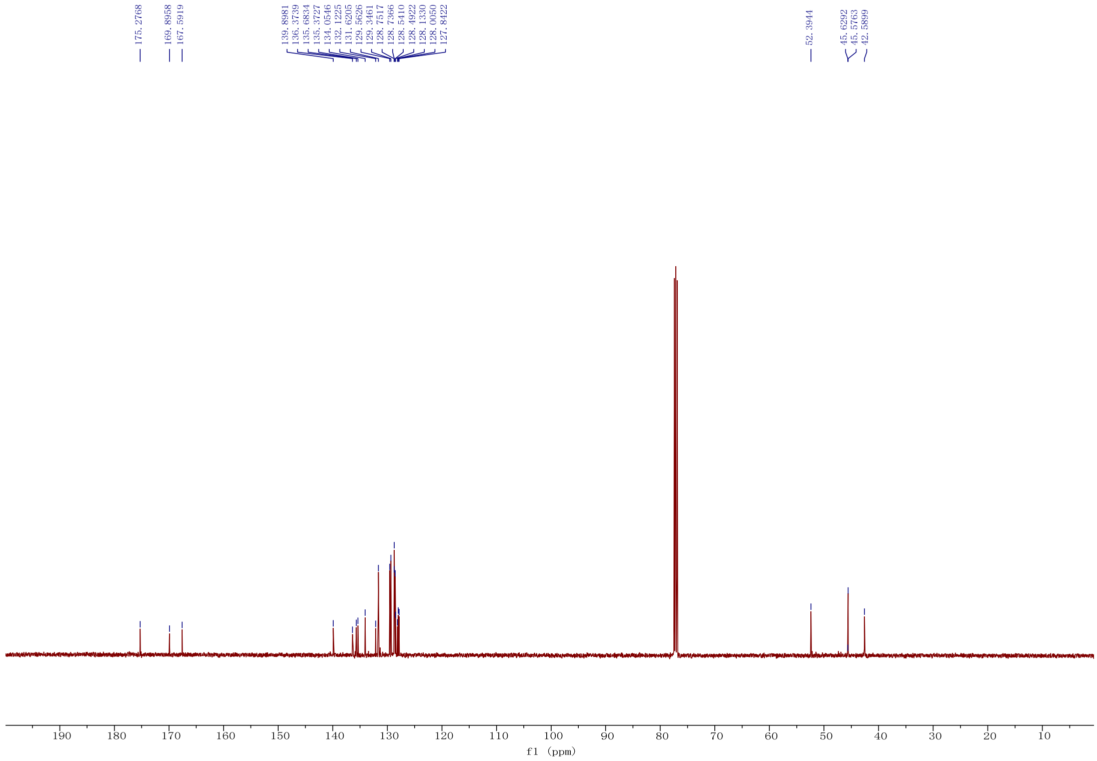

175.2768 169.8958 167.5919
139.8981 136.3739 135.6834 135.3727 134.0546 132.1225 131.6205 129.5626 129.3461 128.7517 128.7366 128.5410 128.4922 128.1330 128.0050 127.8422
52.3944 45.6292 45.5763 42.5899
190 180 170 160 150 140 130 120 110 100 90 80 70 60 50 40 30 20 10
f1 (ppm)

3na

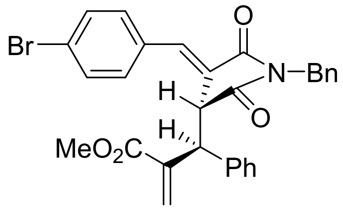

3na

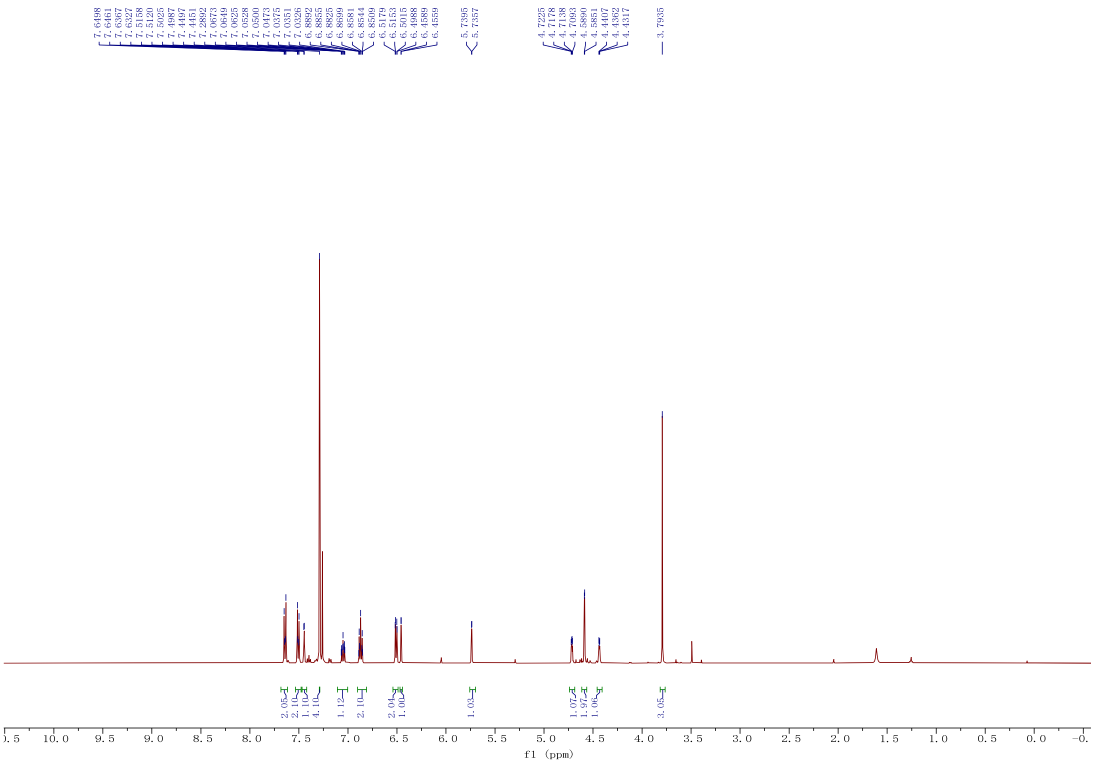

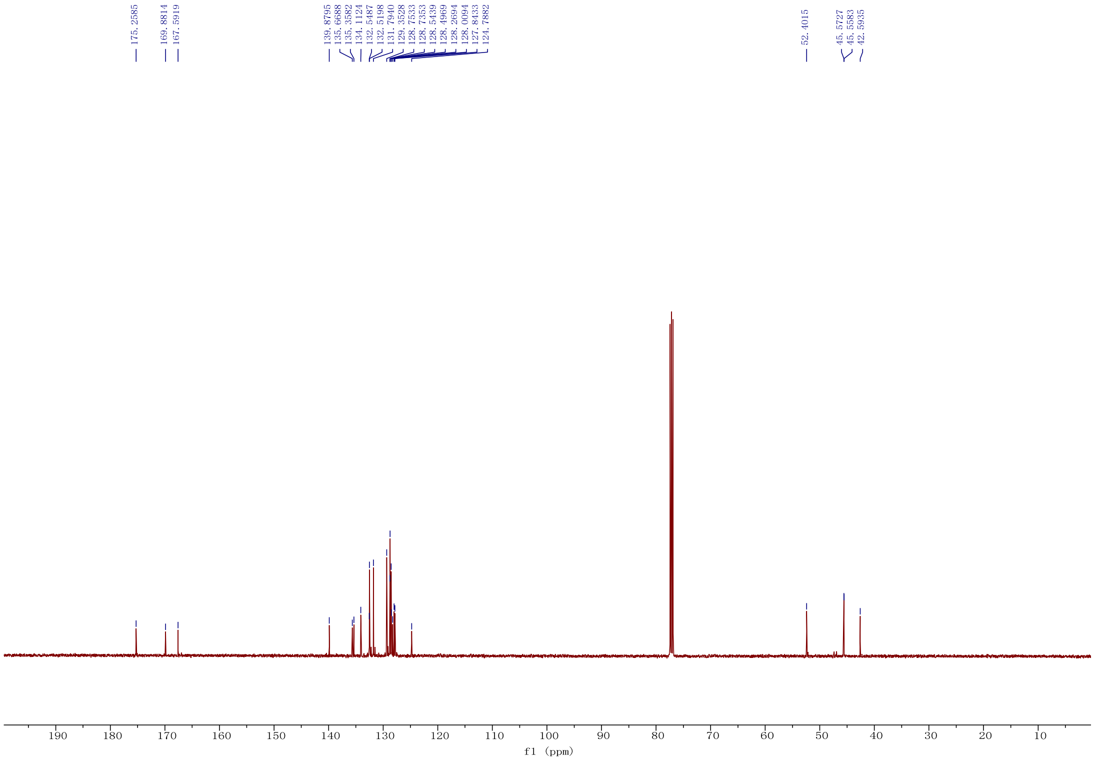

3oa

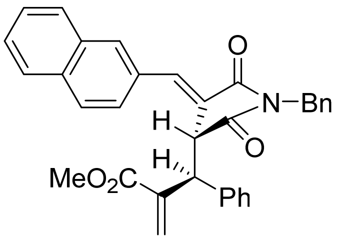

3oa

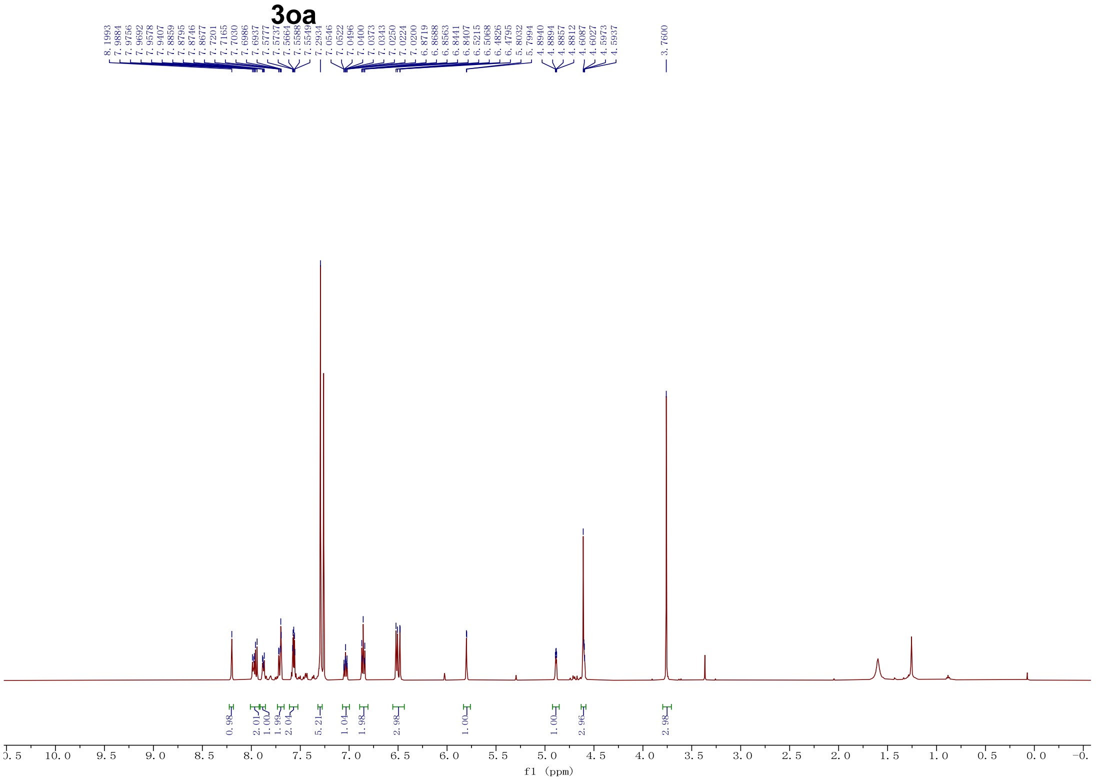

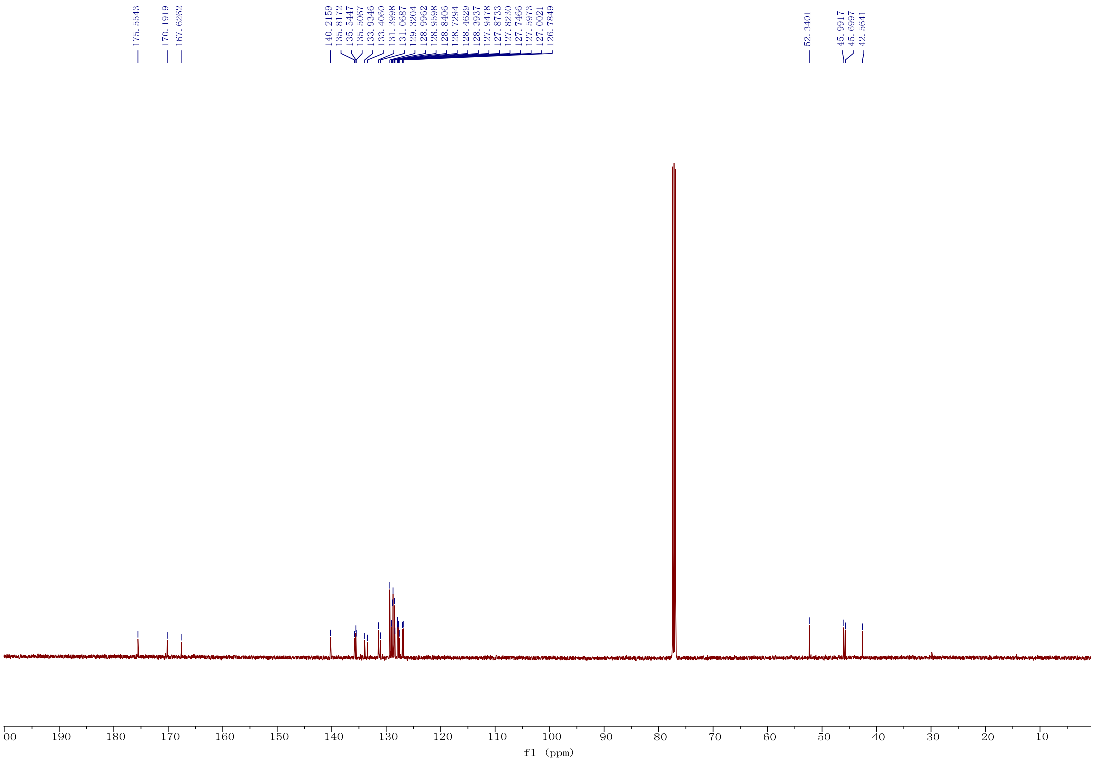

3eb

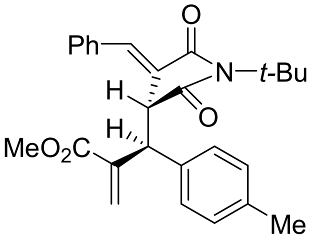

Ph
O
N
t-Bu
H
O
H
MeO2C
Me

3eb

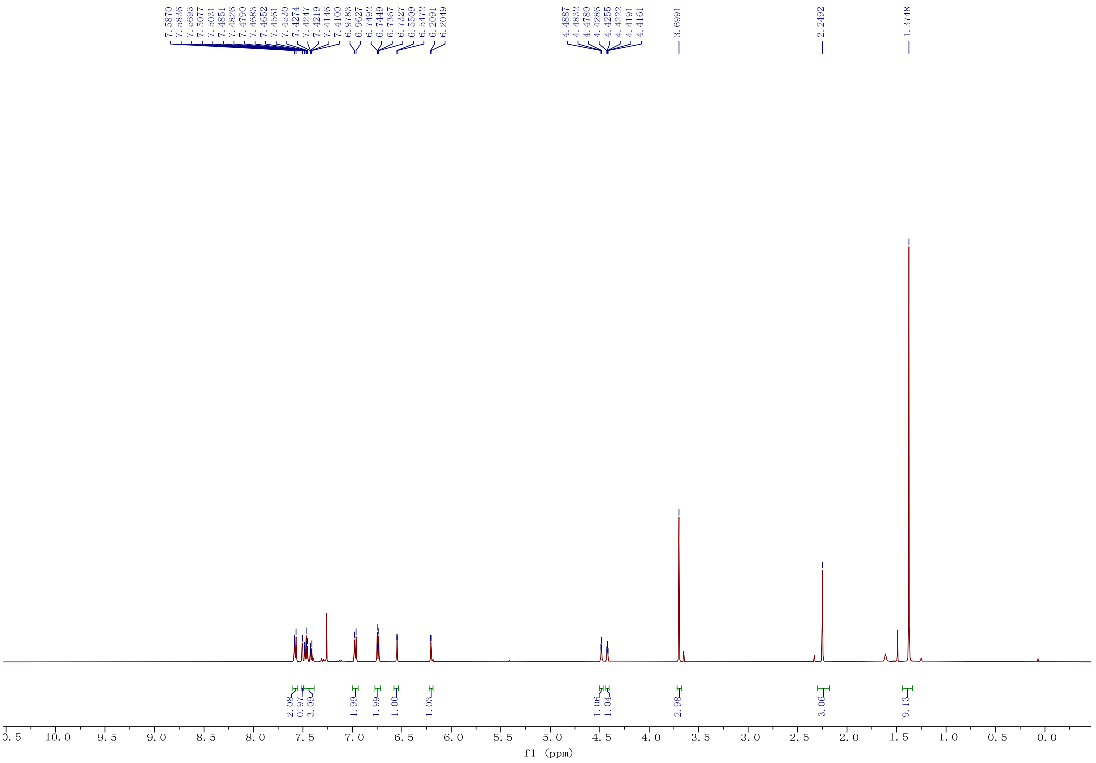

7.5870
7.5836
7.5693
7.5077
7.5031
7.4851
7.4826
7.4790
7.4683
7.4652
7.4561
7.4530
7.4274
7.4247
7.4219
7.4146
7.4100
6.9783
6.9627
6.7492
6.7449
6.7367
6.7327
6.5509
6.5472
6.2091
6.2049
4.4887
4.4832
4.4790
4.4286
4.4255
4.4222
4.4191
4.4161
3.6991
2.2492
1.3748
2.08
0.97
3.09
1.99
1.99
1.00
1.03
1.06
1.04
2.98
3.06
9.13
f1 (ppm)

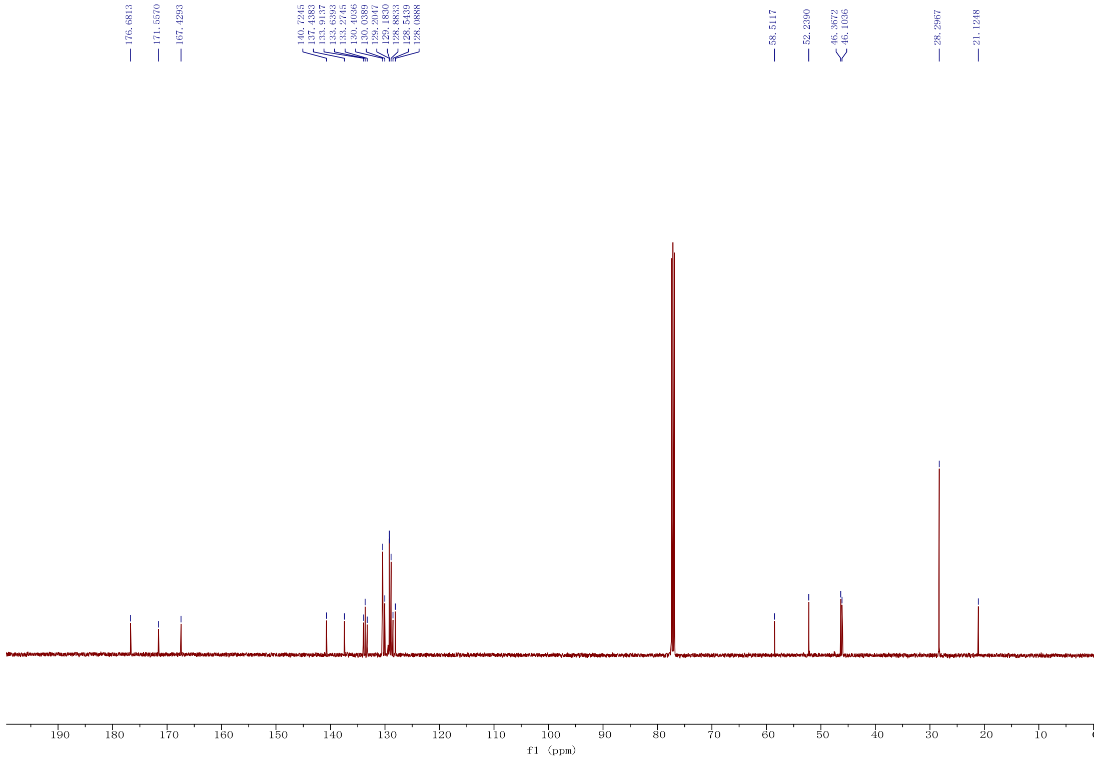

176.6813
171.5570
167.4293
140.7245
137.4383
133.9137
133.6393
133.2745
130.4036
130.0389
129.2047
129.1830
128.8833
128.5439
128.0888
58.5117
52.2390
46.3672
46.1036
28.2967
21.1248
f1 (ppm)

3ec

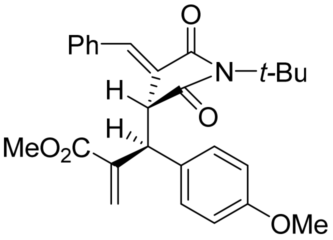
Ph
O
N—t-Bu
H
H
O
MeO2C
OMe

3ec

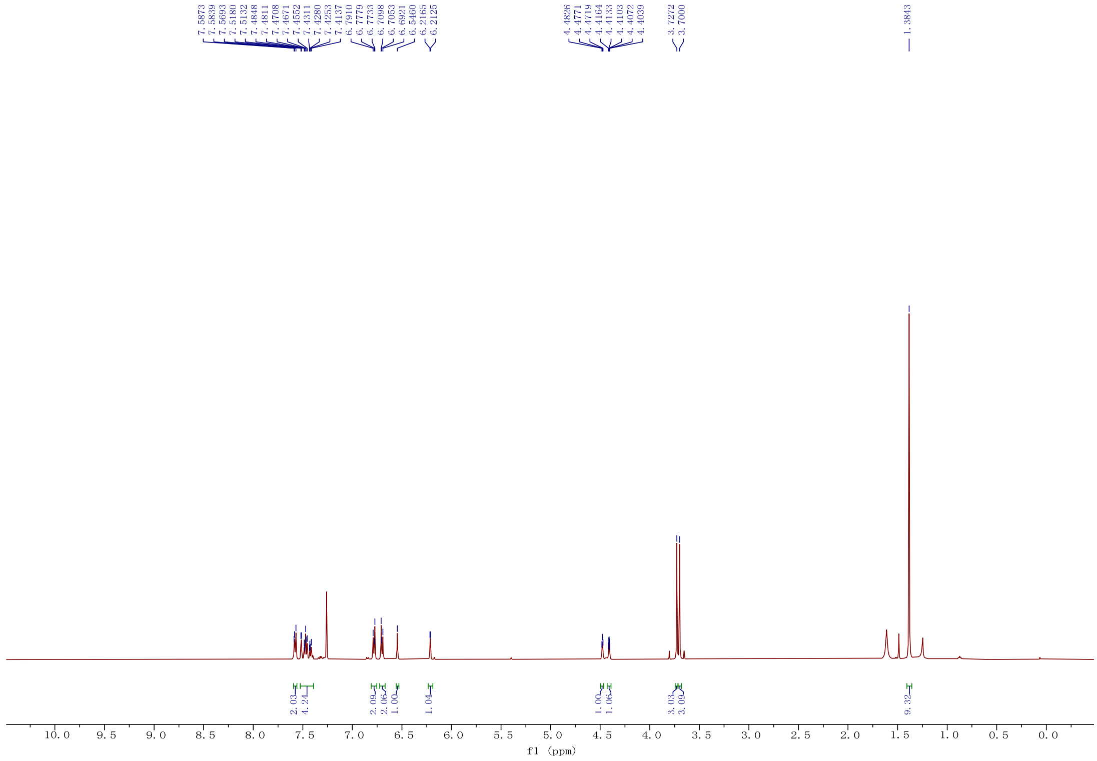
7.5873
7.5839
7.5693
7.5180
7.5132
7.4848
7.4811
7.4708
7.4671
7.4552
7.4311
7.4280
7.4253
7.4137
6.7910
6.7779
6.7733
6.7098
6.7053
6.6921
6.5460
6.2165
6.2125
4.4826
4.4771
4.4719
4.4164
4.4133
4.4103
4.4072
4.4039
3.7272
3.7000
1.3843
2.03
4.21
2.09
2.06
1.00
1.04
1.00
1.06
3.03
3.05
9.32
10.0 9.5 9.0 8.5 8.0 7.5 7.0 6.5 6.0 5.5 5.0 4.5 4.0 3.5 3.0 2.5 2.0 1.5 1.0 0.5 0.0
f1 (ppm)

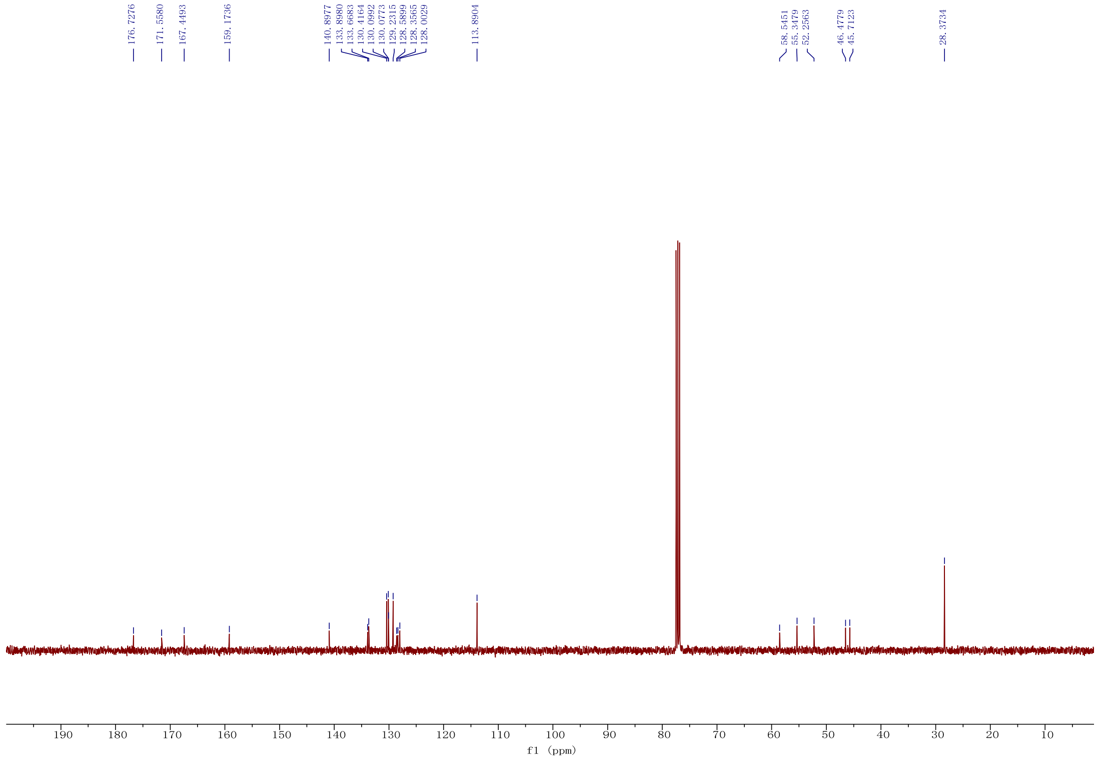
176.7276
171.5580
167.4493
159.1736
140.8977
133.8980
133.6683
130.4164
130.0992
130.0773
129.2315
128.5899
128.3565
128.0029
113.8904
58.5451
55.3479
52.2563
46.4779
45.7123
28.3734
190 180 170 160 150 140 130 120 110 100 90 80 70 60 50 40 30 20 10
f1 (ppm)

3ed

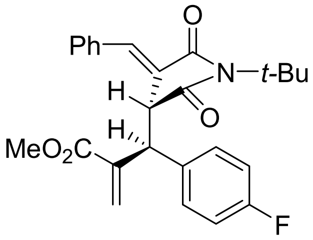
Ph
O
N—t-Bu
H
O
H
MeO2C
F

3ed

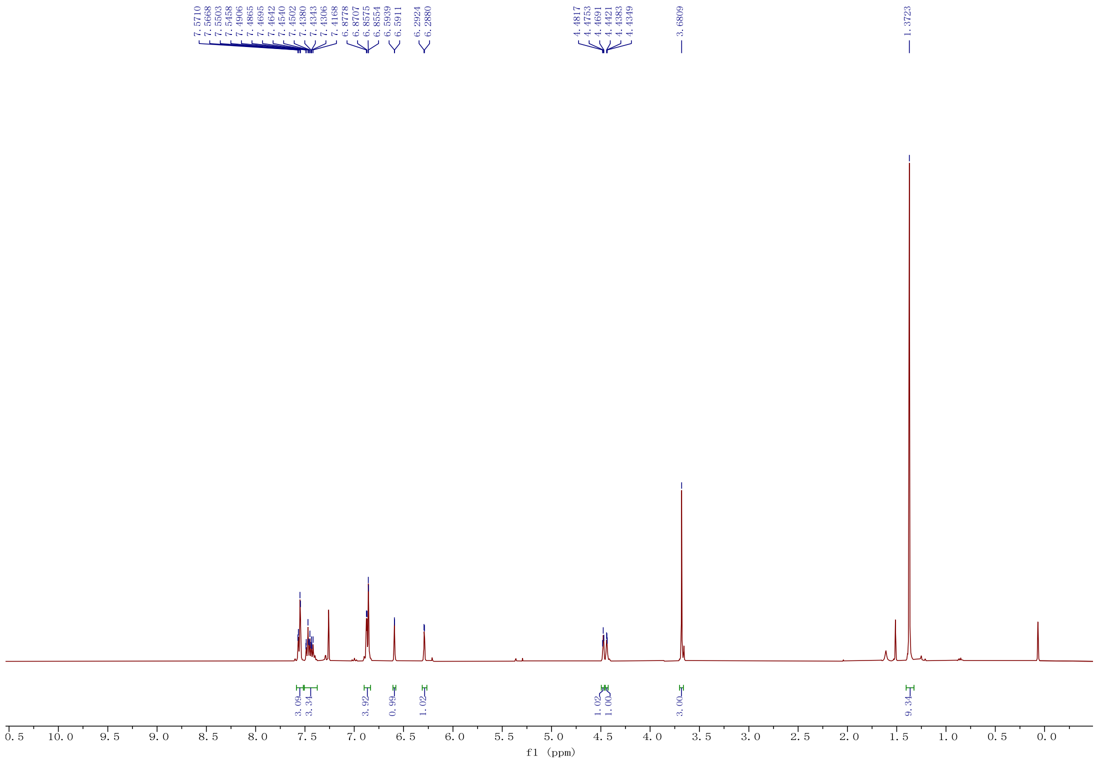
7.5710
7.5668
7.5503
7.5458
7.4906
7.4865
7.4695
7.4642
7.4540
7.4502
7.4380
7.4343
7.4306
7.4168
6.8778
6.8707
6.8575
6.8554
6.5939
6.5911
6.2924
6.2880
4.4817
4.4753
4.4691
4.4421
4.4383
4.4349
3.6809
1.3723
3.09
3.34
3.92
0.99
1.02
1.02
1.00
3.00
9.34
f1 (ppm)

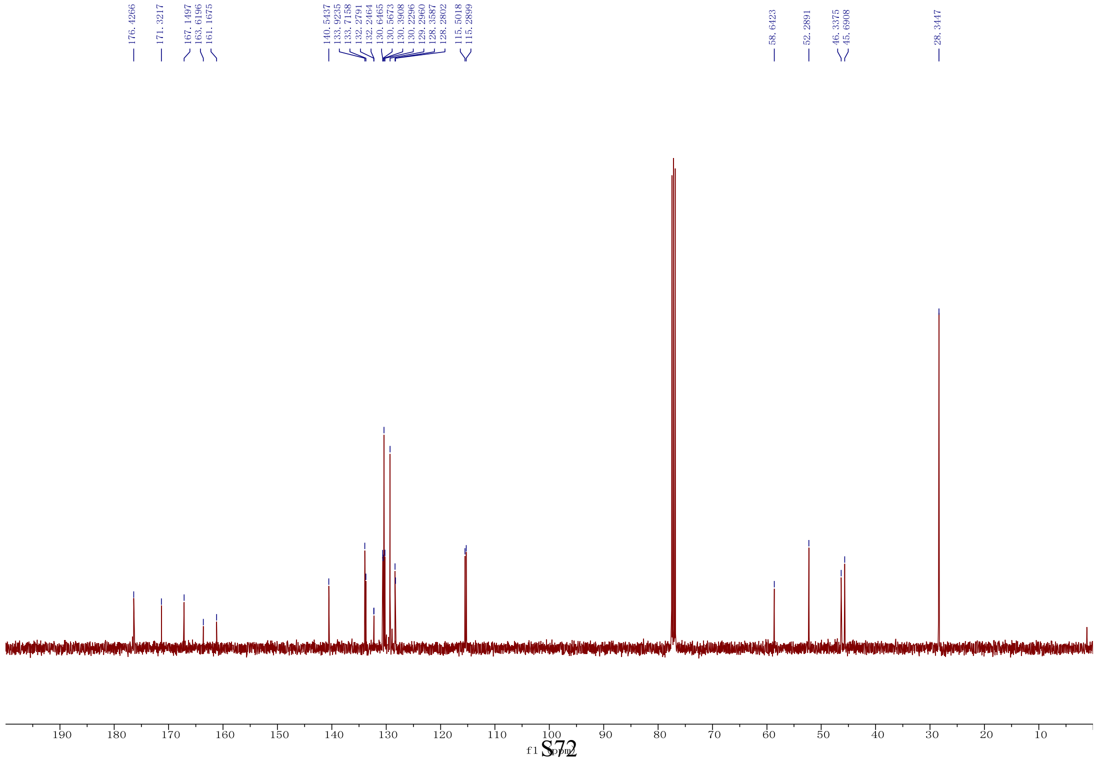
176.4266
171.3217
167.1497
163.6196
161.1675
140.5437
133.9235
133.7158
132.2791
132.2464
130.6465
130.5673
130.3908
130.2296
129.2960
128.3587
128.2802
115.5018
115.2899
58.6423
52.2891
46.3375
45.6908
28.3447
f1 (ppm)

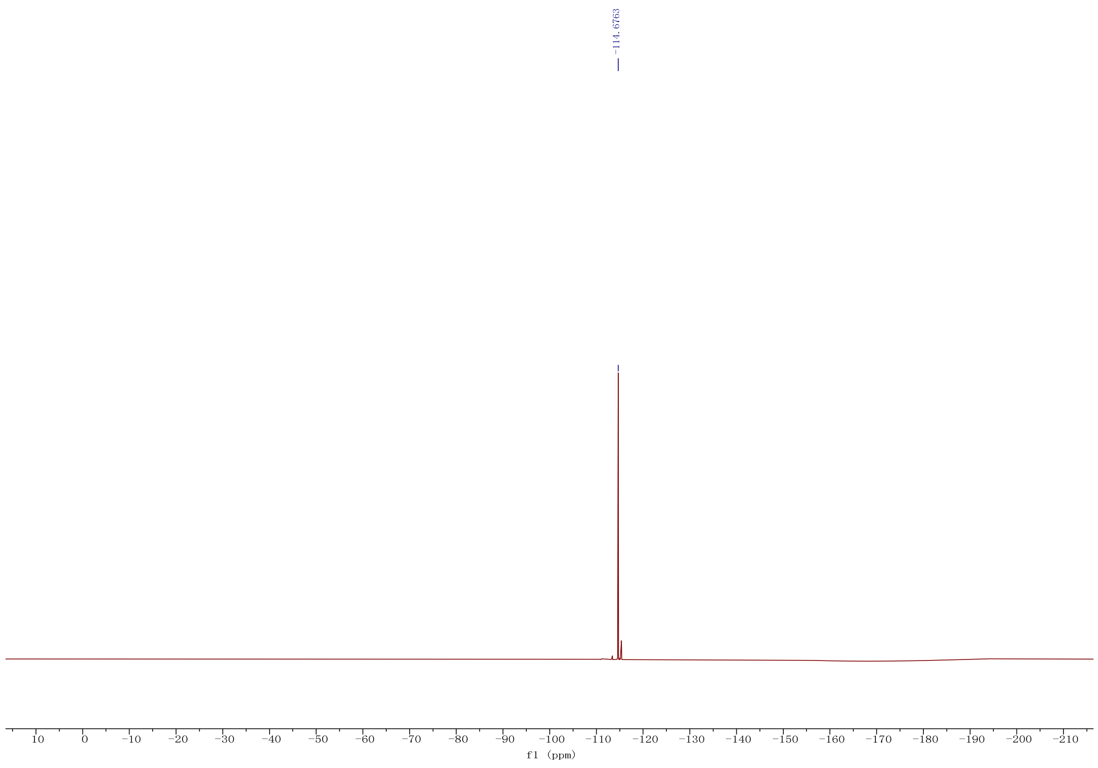
-114.6763
10 0 −10 −20 −30 −40 −50 −60 −70 −80 −90 −100 −110 −120 −130 −140 −150 −160 −170 −180 −190 −200 −210
f1 (ppm)

3ee

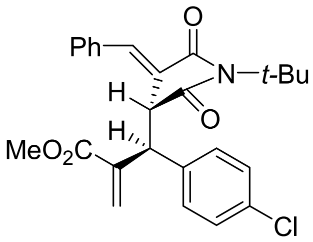
Ph
O
N
t-Bu
H
O
H
MeO2C
Cl

3ee

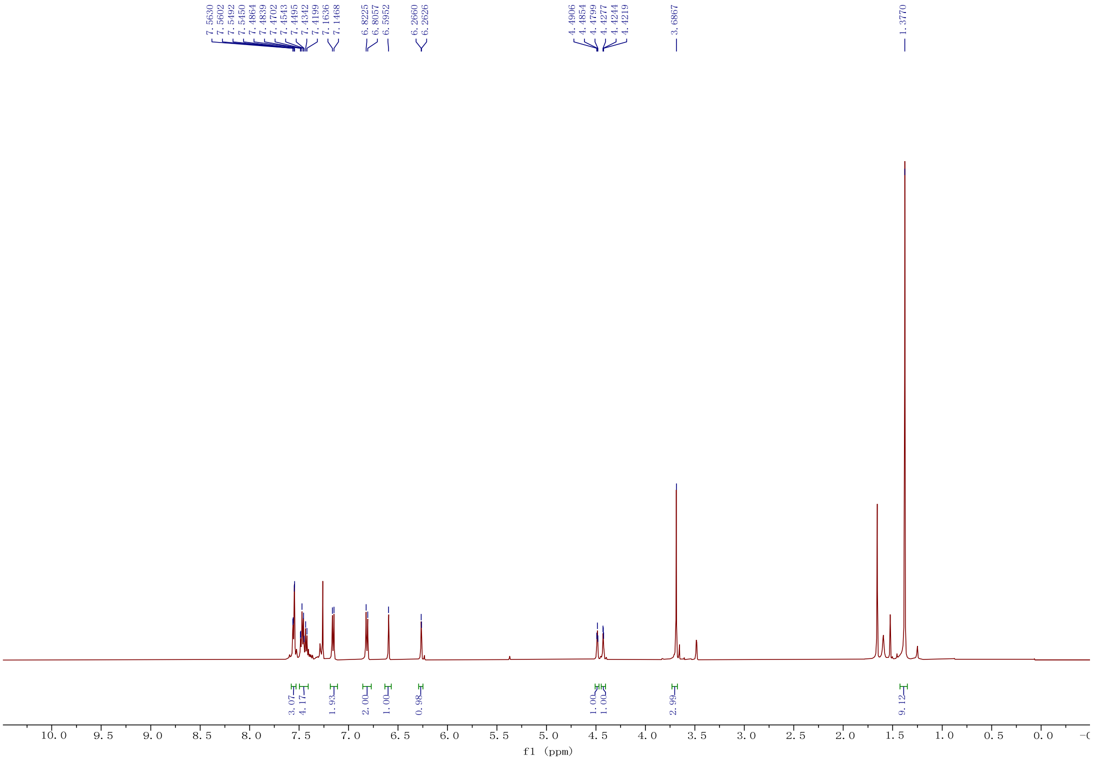
7.5630
7.5602
7.5492
7.5450
7.4864
7.4839
7.4702
7.4543
7.4495
7.4342
7.4199
7.1636
7.1468
6.8225
6.8057
6.5952
6.2660
6.2626
4.4906
4.4854
4.4799
4.4277
4.4244
4.4219
3.6867
1.3770
3.07
4.17
1.93
2.00
1.00
0.98
1.00
1.00
2.99
9.12
f1 (ppm)

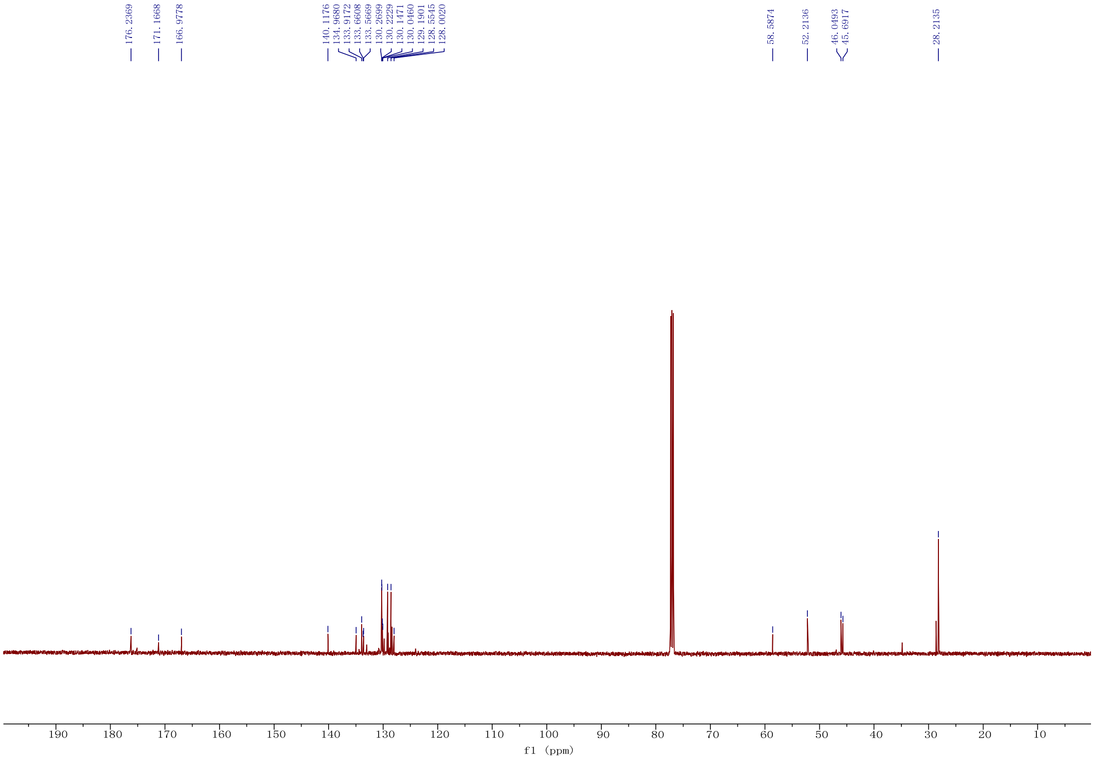
176.2369
171.1668
166.9778
140.1176
134.9680
133.9172
133.6608
133.5669
130.2699
130.2229
130.1471
130.0460
129.1901
128.5545
128.0020
58.5874
52.2136
46.0493
45.6917
28.2135
f1 (ppm)

3ef

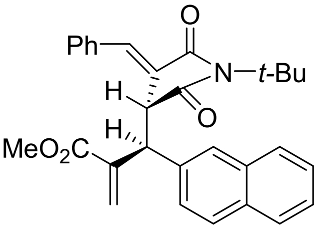

3ef

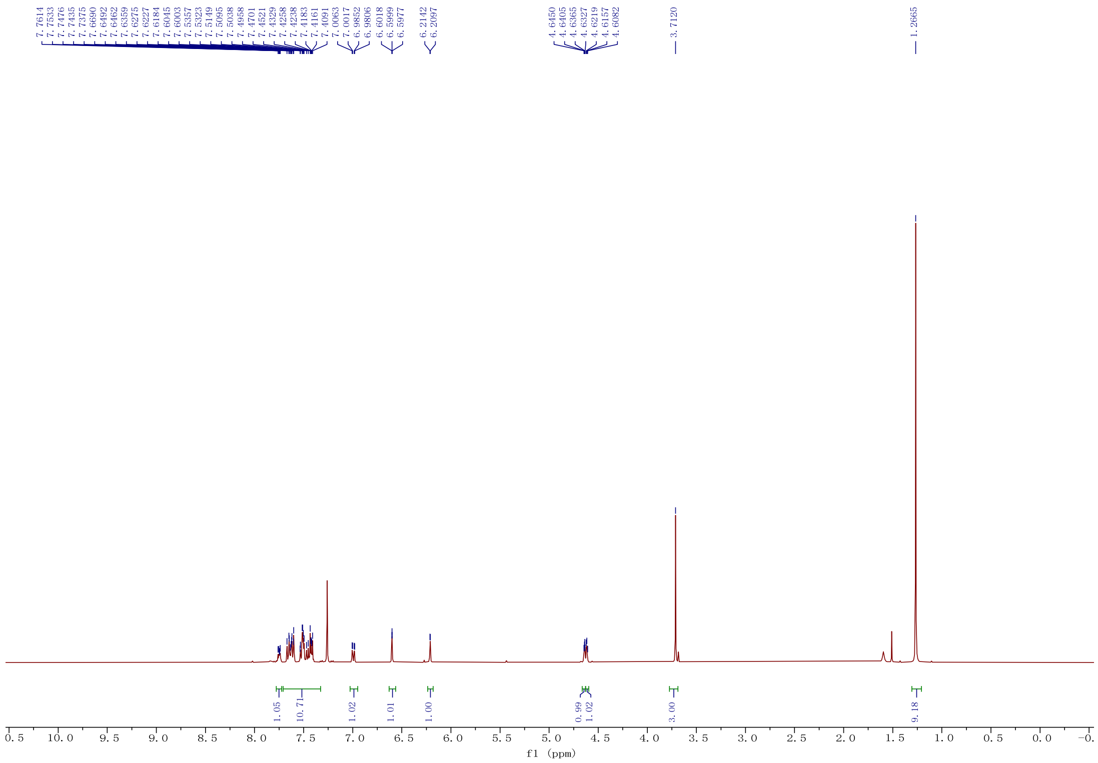

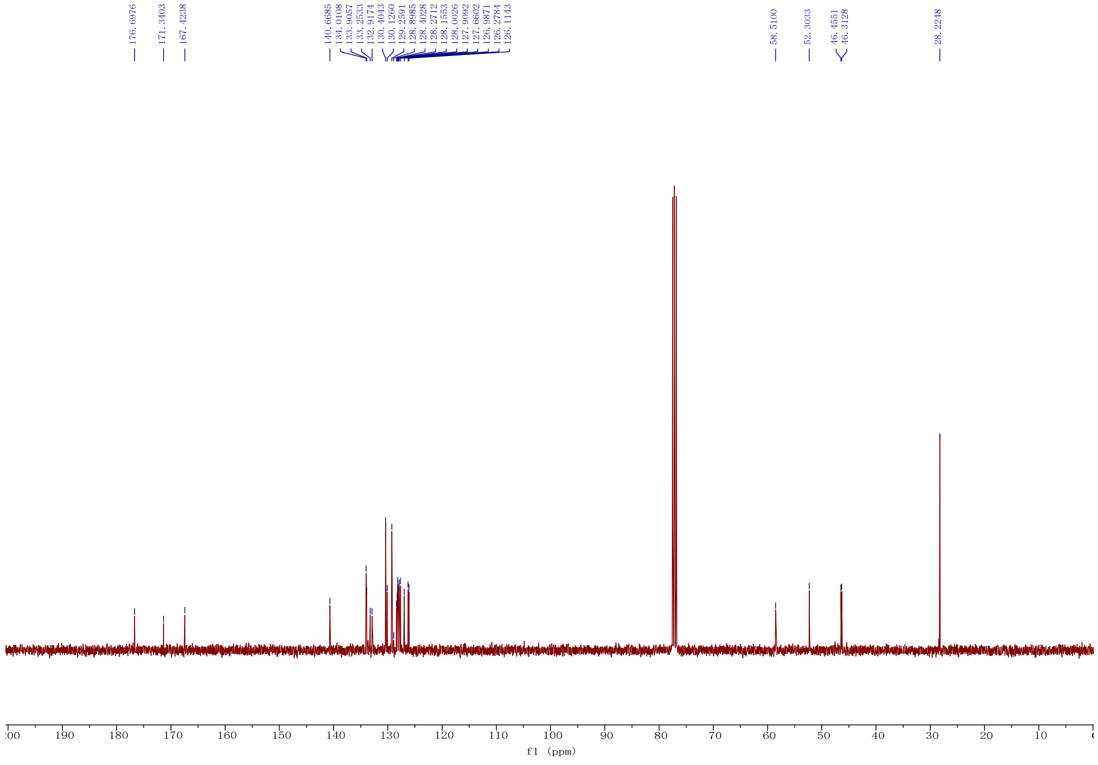

3eg

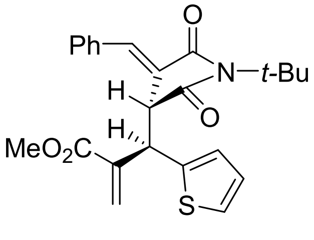
Ph
O
N—t-Bu
H
O
H
MeO2C
S

3eg

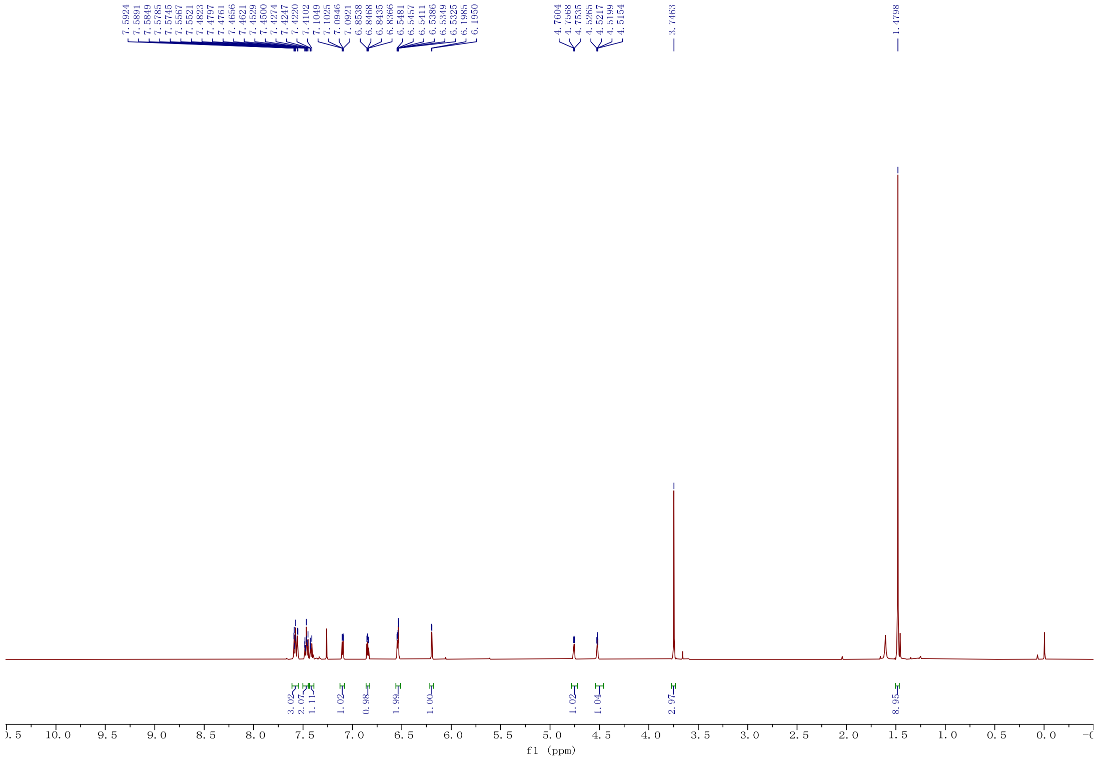
7.5924
7.5891
7.5849
7.5785
7.5745
7.5567
7.5521
7.4823
7.4797
7.4761
7.4656
7.4621
7.4529
7.4500
7.4274
7.4247
7.4220
7.4102
7.1049
7.1025
7.0946
7.0921
6.8538
6.8468
6.8435
6.8366
6.5481
6.5457
6.5411
6.5386
6.5349
6.5325
6.1985
6.1950
4.7604
4.7568
4.7535
4.5265
4.5217
4.5199
4.5154
3.7463
1.4798
3.02
2.07
1.11
1.02
0.98
1.99
1.00
1.02
1.04
2.97
8.95
f1 (ppm)

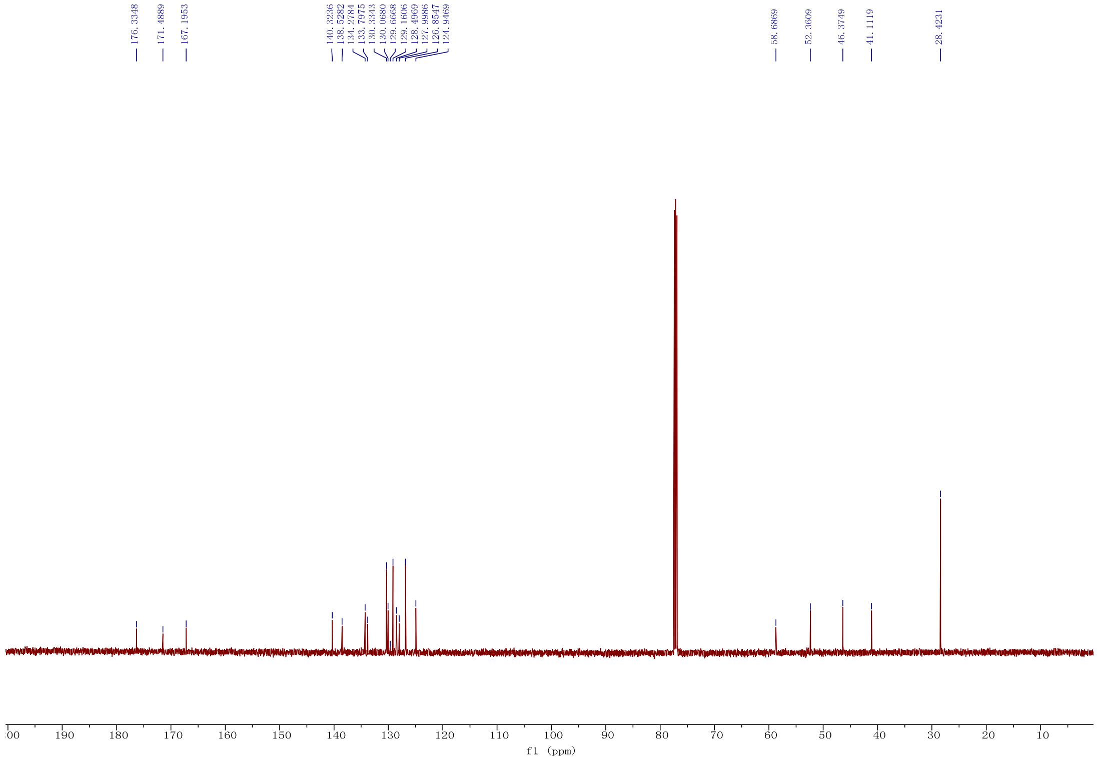
176.3348
171.4889
167.1953
140.3236
138.5282
134.2784
133.7975
130.3343
130.0680
129.6668
129.1606
128.4969
127.9986
126.8547
124.9469
58.6869
52.3609
46.3749
41.1119
28.4231
f1 (ppm)

3eh

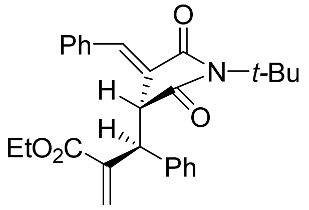
Ph
O
N—t-Bu
H
O
H
EtO2C
Ph

3eh

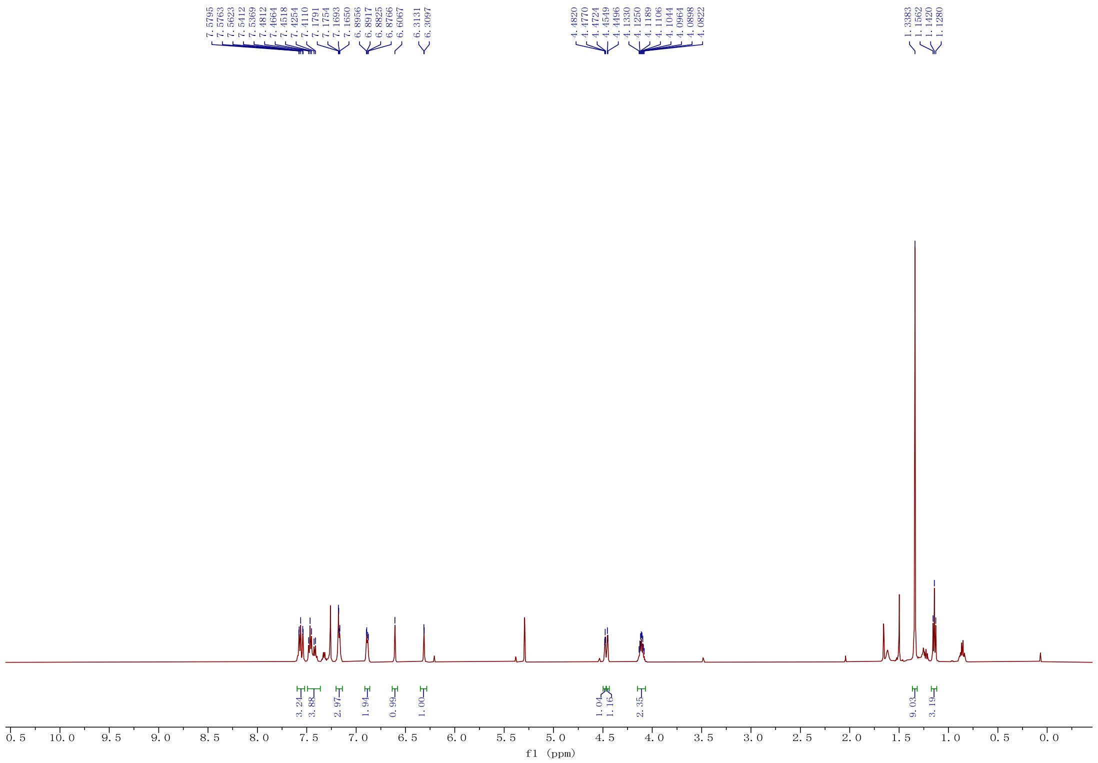
7.5795
7.5763
7.5623
7.5412
7.5369
7.4812
7.4664
7.4518
7.4254
7.4110
7.1791
7.1754
7.1693
7.1650
6.8956
6.8917
6.8825
6.8766
6.6067
6.3131
6.3097
4.4820
4.4770
4.4724
4.4549
4.4496
4.1330
4.1250
4.1189
4.1106
4.1044
4.0964
4.0898
4.0822
1.3383
1.1562
1.1420
1.1280
3.24
3.88
2.97
1.94
0.99
1.00
1.04
1.15
2.35
9.03
3.19
f1 (ppm)

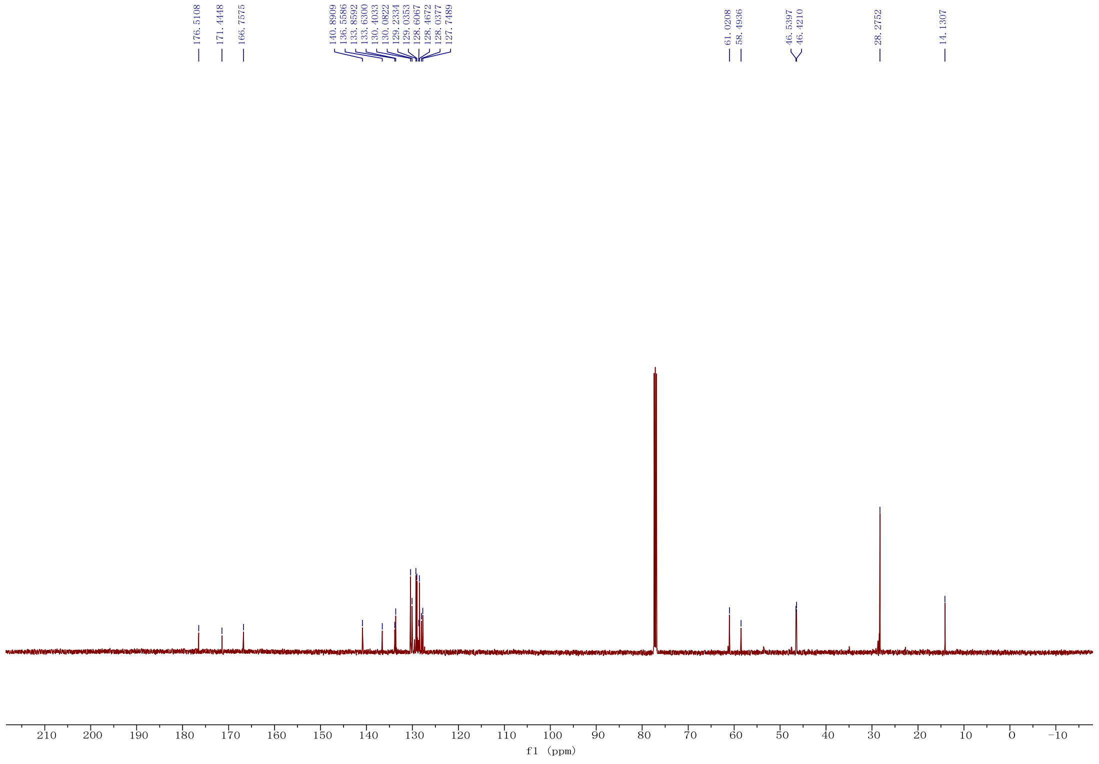
176.5108
171.4448
166.7575
140.8909
136.5586
133.8592
133.6300
130.4033
130.0822
129.2334
129.0353
128.6067
128.4672
128.0377
127.7489
61.0208
58.4936
46.5397
46.4210
28.2752
14.1307
f1 (ppm)

3ei

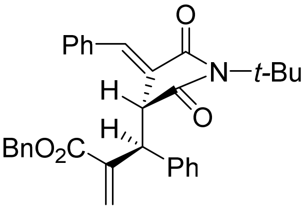

Ph
O
N—t-Bu
H
O
H
BnO2C
Ph

3ei

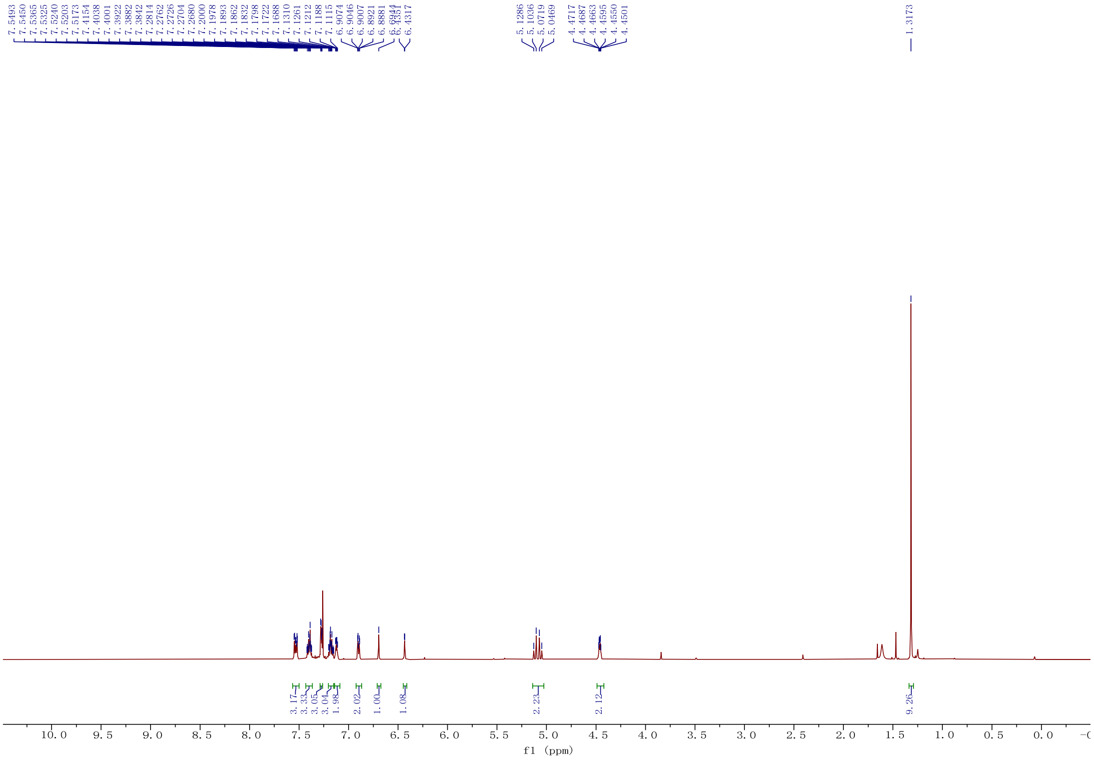

7.5493 7.5450 7.5365 7.5325 7.5240 7.5203 7.5173 7.4154 7.4038 7.4001 7.3922 7.3882 7.3812 7.2814 7.2762 7.2726 7.2704 7.2080 7.2000 7.1978 7.1893 7.1862 7.1832 7.1798 7.1722 7.1688 7.1310 7.1261 7.1212 7.1188 7.1115 6.9074 6.9046 6.9007 6.8921 6.8881 6.6944 6.4351 6.4317
5.1286 5.1036 5.0719 5.0469
4.4717 4.4687 4.4663 4.4595 4.4550 4.4501
1.3173
3.17 3.33 3.05 3.04 1.98 2.02 1.00 1.08 2.23 2.12 9.26
10.0 9.5 9.0 8.5 8.0 7.5 7.0 6.5 6.0 5.5 5.0 4.5 4.0 3.5 3.0 2.5 2.0 1.5 1.0 0.5 0.0
f1 (ppm)

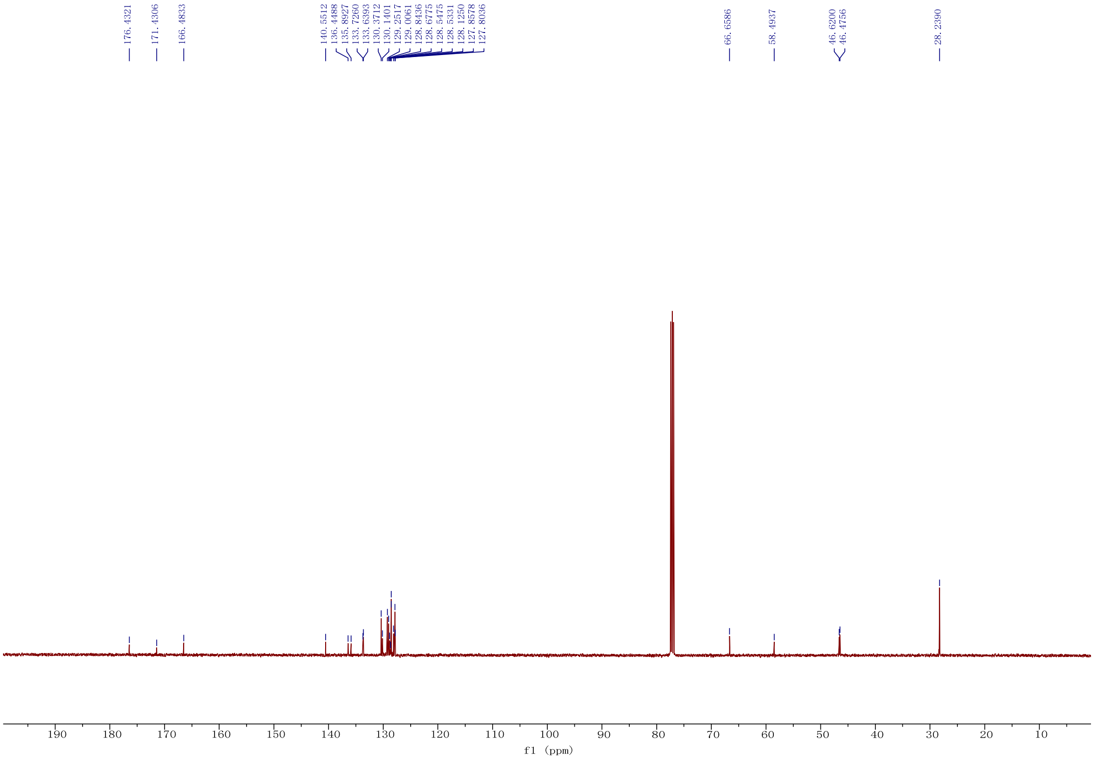

176.4321 171.4306 166.4833
140.5512 136.4488 135.8927 133.7260 133.6293 130.3712 130.1401 129.2517 129.0061 128.8136 128.6775 128.5475 128.5331 128.1250 127.8578 127.8036
66.6586 58.4937 46.6200 46.4756 28.2390
190 180 170 160 150 140 130 120 110 100 90 80 70 60 50 40 30 20 10
f1 (ppm)

3ej

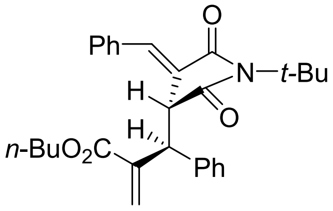

3ej

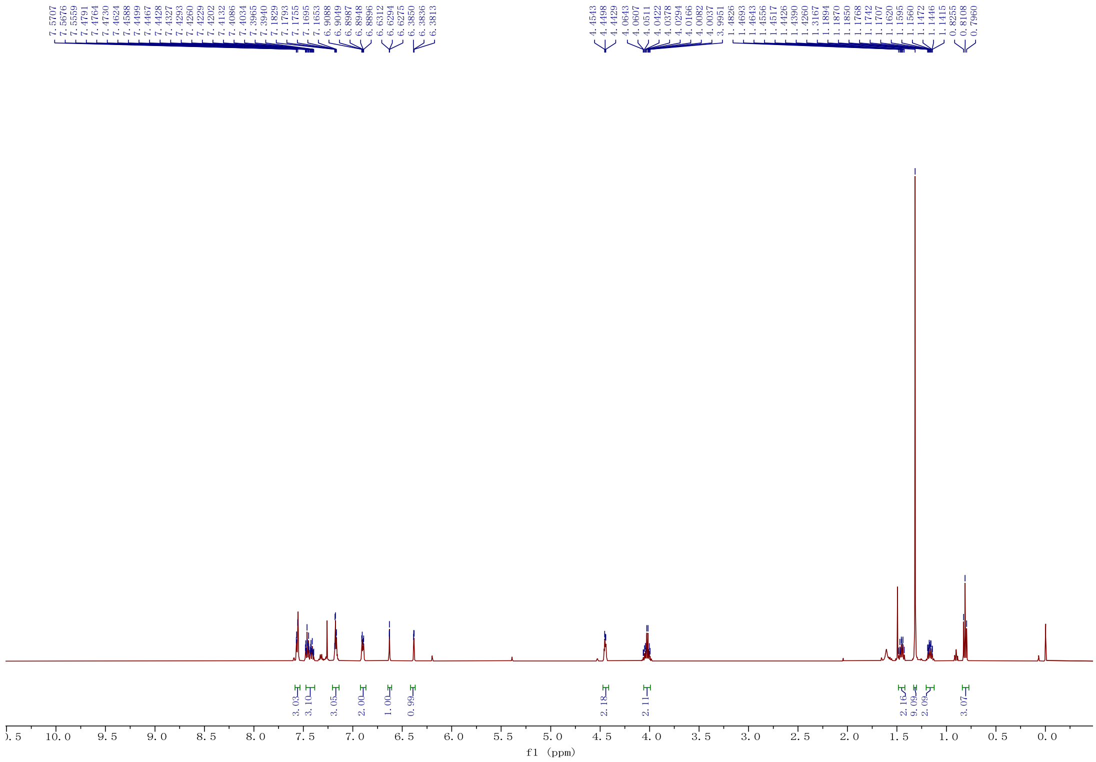

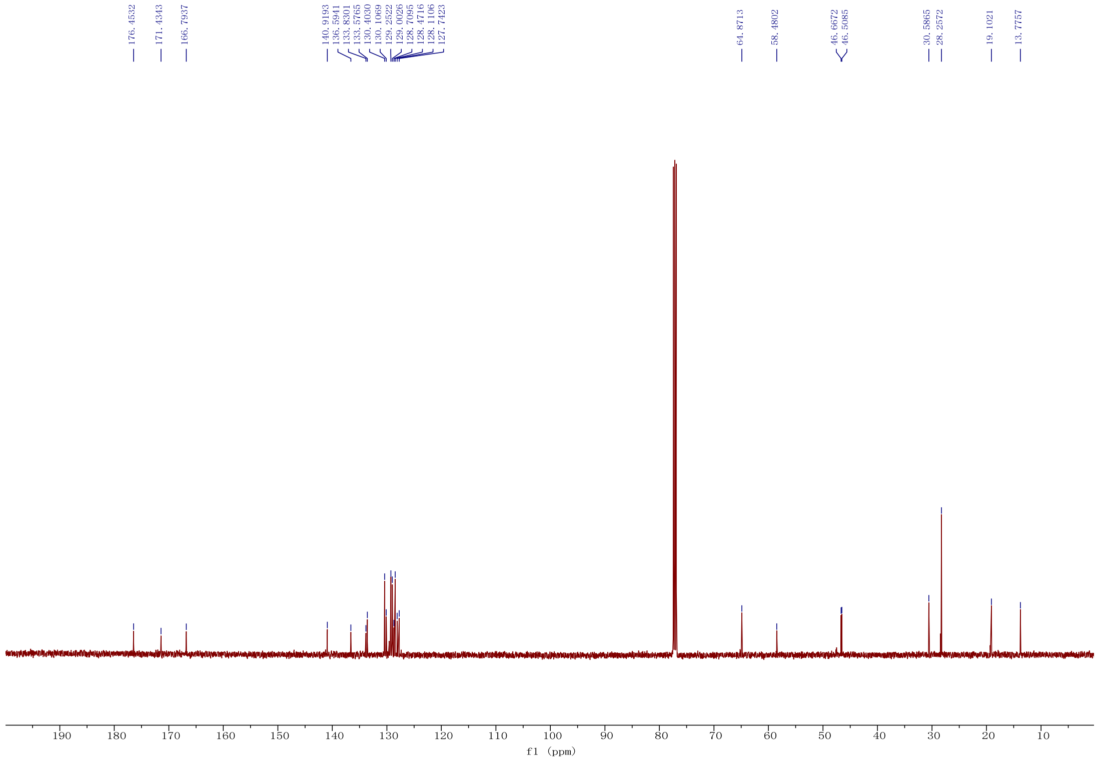

3ek

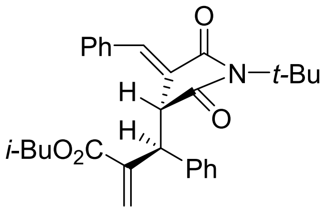

Ph
O
N—t-Bu
H
O
H
i-BuO2C
Ph

3ek

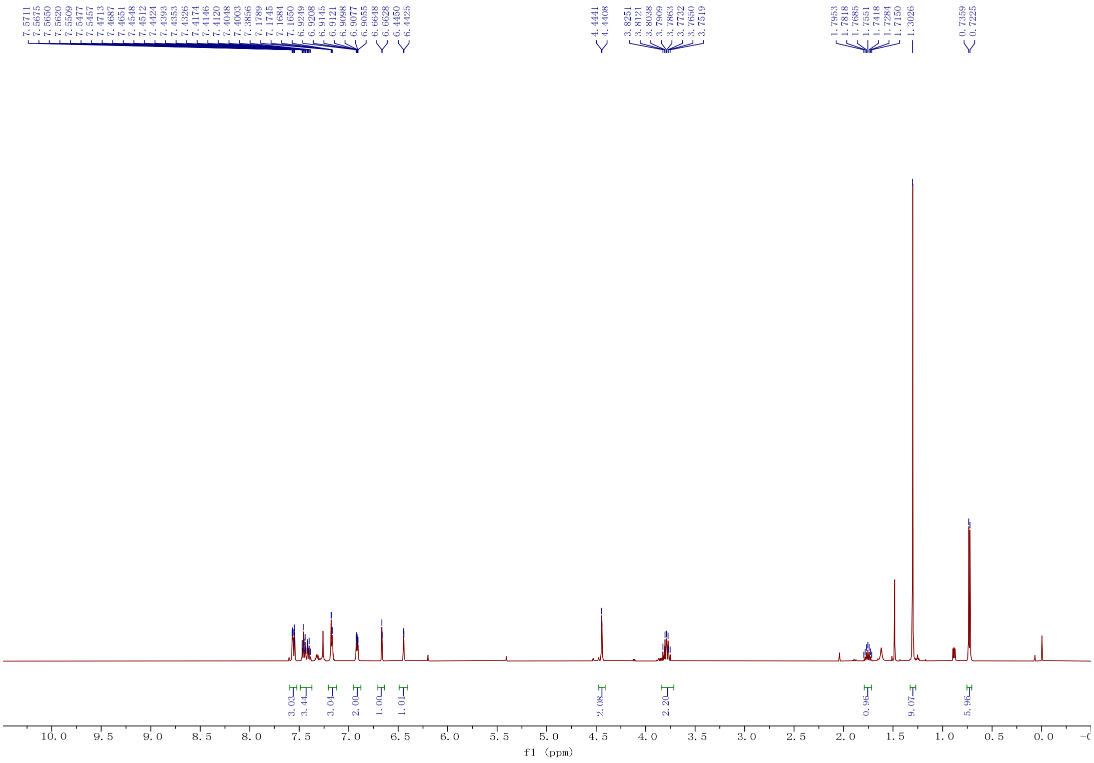

7.5711
7.5675
7.5650
7.5620
7.5509
7.5477
7.5457
7.4713
7.4687
7.4651
7.4548
7.4512
7.4424
7.4393
7.4353
7.4326
7.4174
7.4146
7.4120
7.4048
7.4003
7.3856
7.1789
7.1745
7.1684
7.1650
6.9249
6.9208
6.9145
6.9121
6.9098
6.9077
6.9055
6.6648
6.6628
6.4450
6.4425
4.4441
4.4408
3.8251
3.8121
3.8038
3.7909
3.7863
3.7732
3.7650
3.7519
1.7953
1.7818
1.7685
1.7551
1.7418
1.7284
1.7150
1.3026
0.7359
0.7225
3.03
3.44
3.04
2.00
1.00
1.01
2.08
2.20
0.96
9.07
5.96
10.0 9.5 9.0 8.5 8.0 7.5 7.0 6.5 6.0 5.5 5.0 4.5 4.0 3.5 3.0 2.5 2.0 1.5 1.0 0.5 0.0
f1 (ppm)

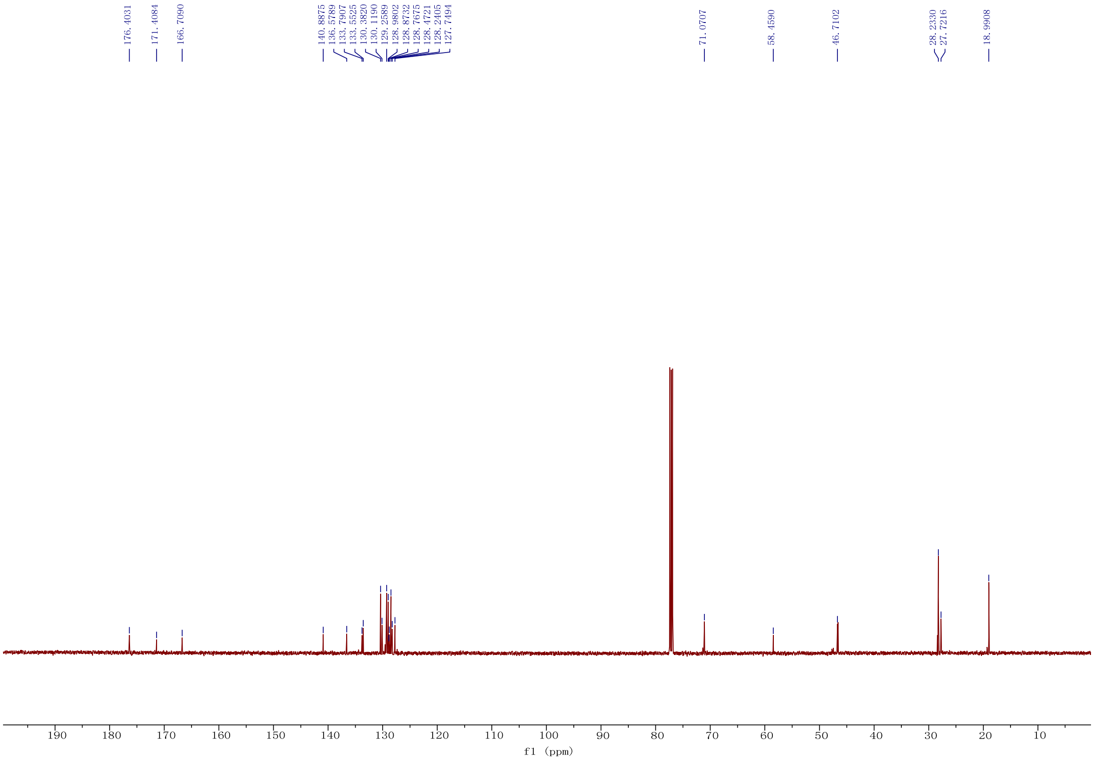

176.4031
171.4084
166.7090
140.8875
136.5789
133.7907
133.5525
130.3820
130.1190
129.2589
128.9802
128.8732
128.7675
128.4721
128.2405
127.7494
71.0707
58.4590
46.7102
28.2330
27.7216
18.9908
190 180 170 160 150 140 130 120 110 100 90 80 70 60 50 40 30 20 10
f1 (ppm)

VII. Copies of Chiral HPLC analysis

3aa-racmic

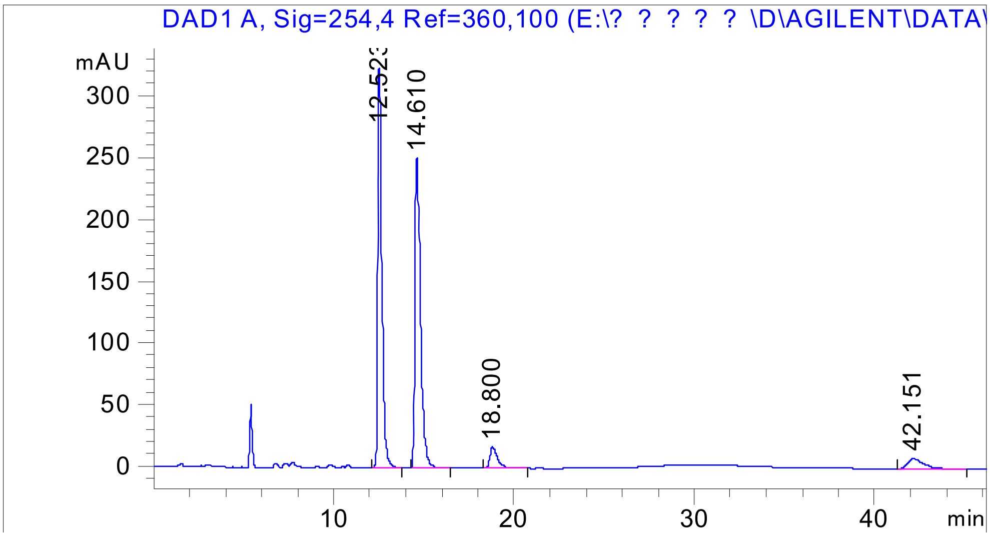

#	Time	Area	Height	Width	Area%	Symmetry
1	12.523	5296	323.6	0.247	45.664	0.572
2	14.61	5296	251.4	0.3178	45.664	0.474
3	18.8	509.5	17.4	0.4368	4.393	0.479
4	42.151	496.4	8.1	0.9191	4.280	0.509

3aa-chiral

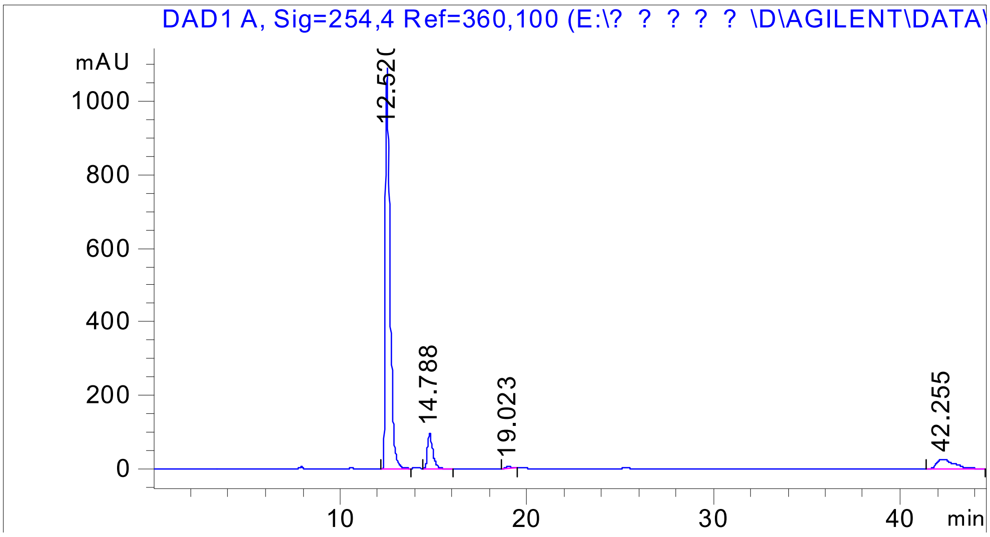

#	Time	Area	Height	Width	Area%	Symmetry
1	12.52	18383.7	1089.4	0.2549	82.954	0.502
2	14.788	1953	98.2	0.302	8.812	0.58
3	19.023	135.3	5.8	0.3709	0.610	0.79
4	42.255	1689.5	25.7	0.9562	7.624	0.425

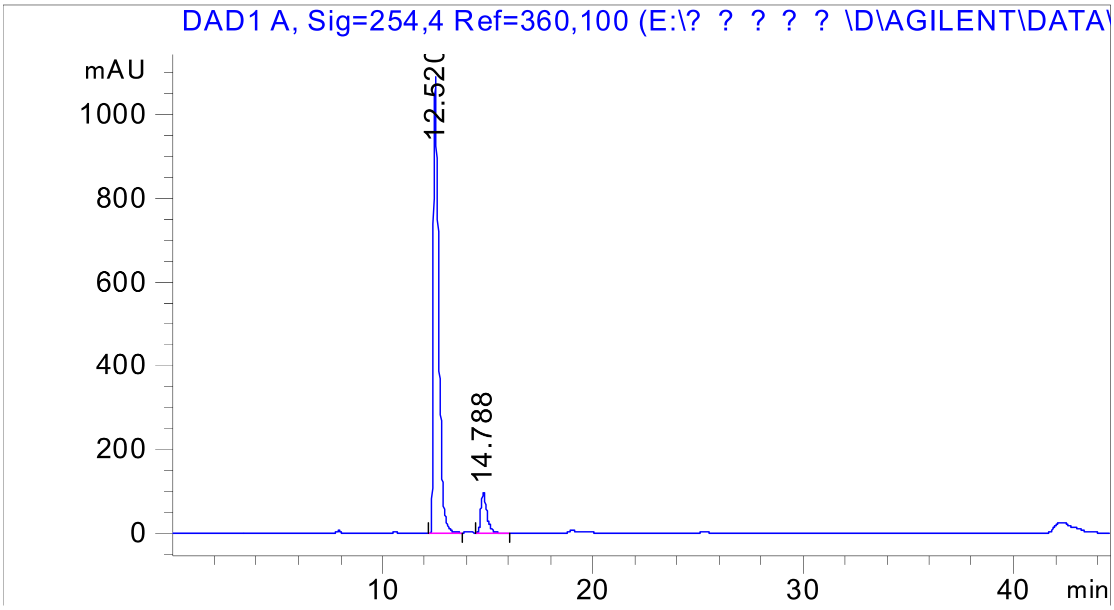

#	Time	Area	Height	Width	Area%	Symmetry
1	12.52	18383.7	1089.4	0.2549	90.397	0.502
2	14.788	1953	98.2	0.302	9.603	0.58

3ba-racmic

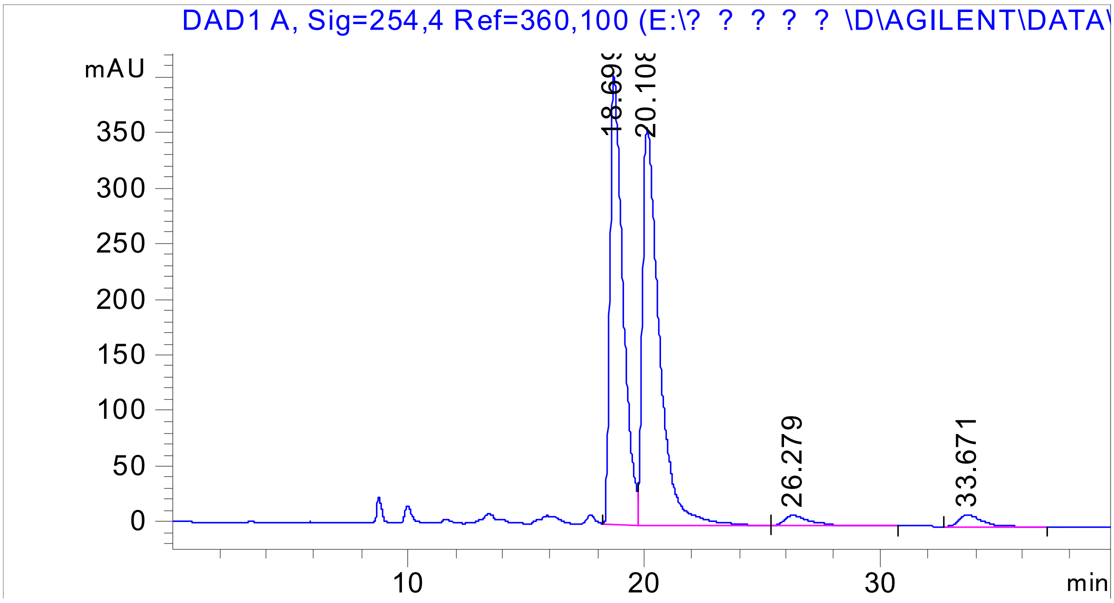

#	Time	Area	Height	Width	Area%	Symmetry
1	18.699	15608.3	403.4	0.5814	45.295	0.406
2	20.108	17496.2	356.6	0.7204	50.774	0.338
3	26.279	659.2	8.7	1.0637	1.913	0.431
4	33.671	695.3	10.3	0.9704	2.018	0.532

3ba-chiral

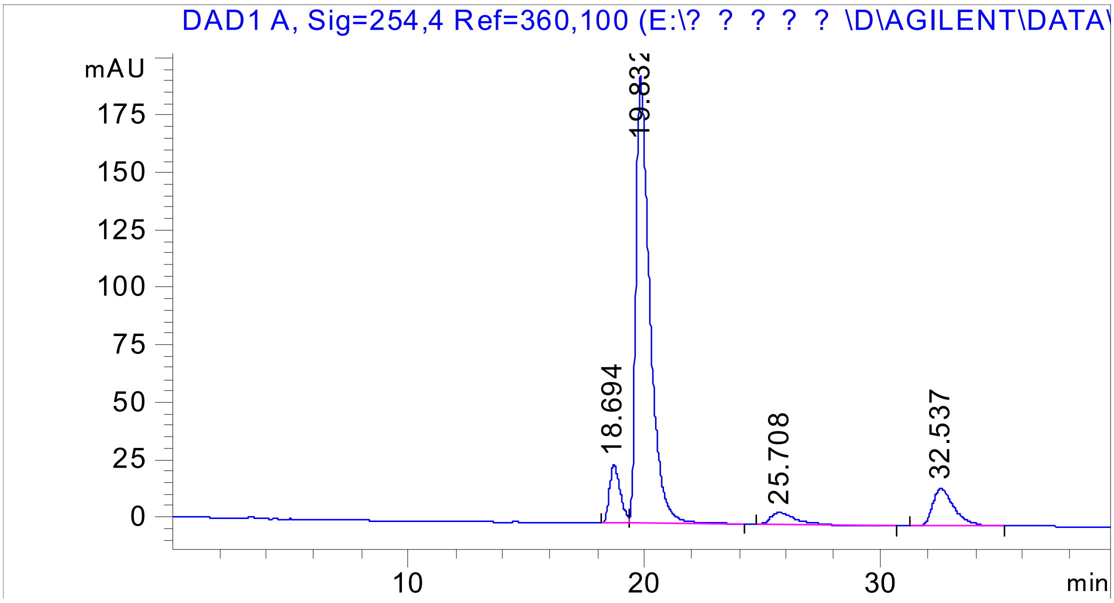

#	Time	Area	Height	Width	Area%	Symmetry
1	18.694	831.1	25.6	0.5012	8.123	0.648
2	19.832	7972.2	195.1	0.6047	77.911	0.451
3	25.708	397.8	5.4	1.0469	3.887	0.481
4	32.537	1031.3	16.2	0.9381	10.079	0.563

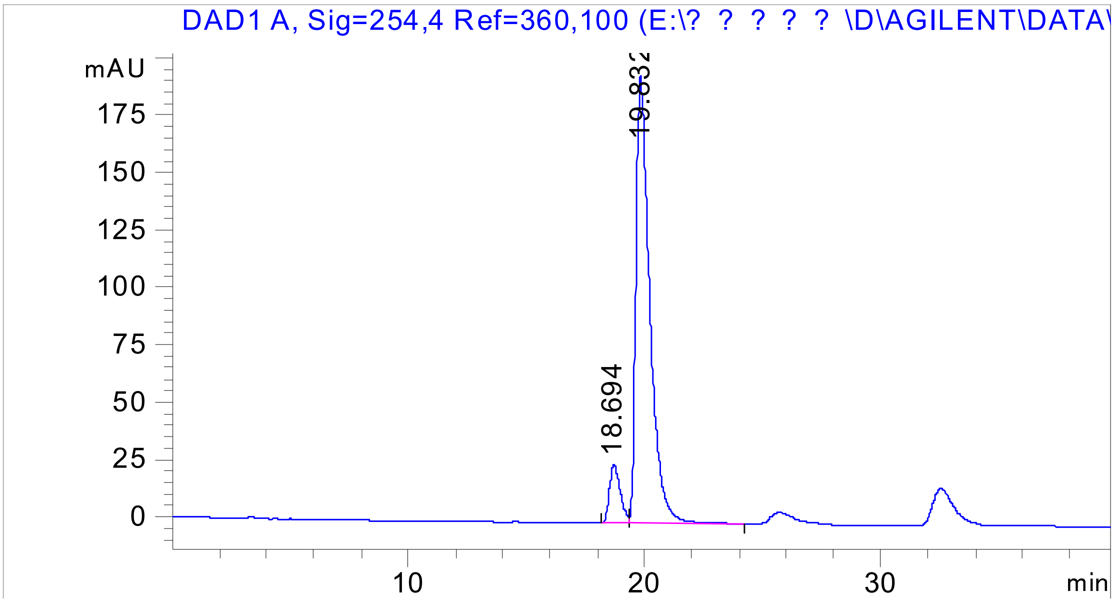

#	Time	Area	Height	Width	Area%	Symmetry
1	18.694	831.1	25.6	0.5012	9.441	0.648
2	19.832	7972.2	195.1	0.6047	90.559	0.451

3ca-racmic

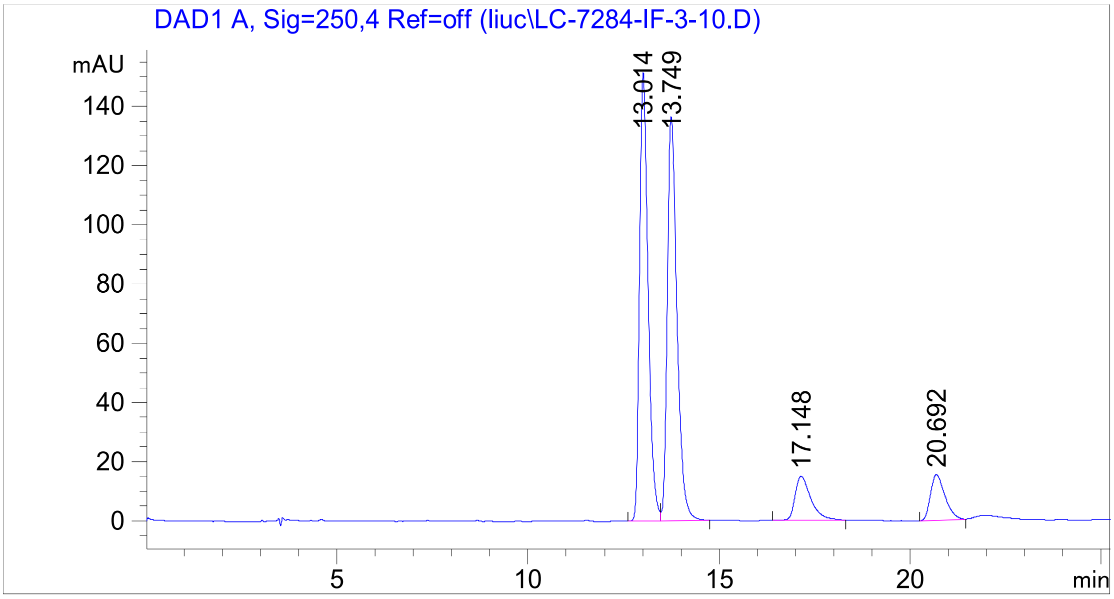

#	Time	Area	Height	Width	Area%	Symmetry
1	13.014	2346.2	151.5	0.2367	42.193	0.668
2	13.749	2384.3	136.4	0.2659	42.878	0.625
3	17.148	425	14.8	0.435	7.644	0.559
4	20.692	405	15.3	0.4059	7.284	0.646

3ca-chiral

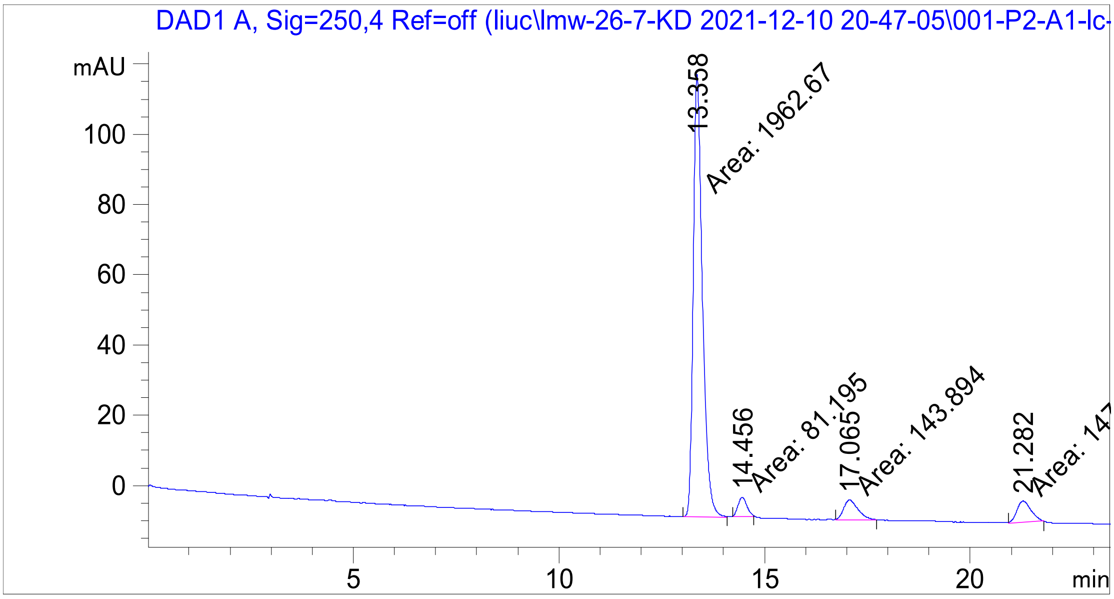

#	Time	Area	Height	Width	Area%	Symmetry
1	13.358	1962.7	126	0.2596	84.029	0.669
2	14.456	81.2	5.5	0.247	3.476	0.848
3	17.065	143.9	5.8	0.4159	6.161	0.651
4	21.282	148	6.1	0.4038	6.334	0.739

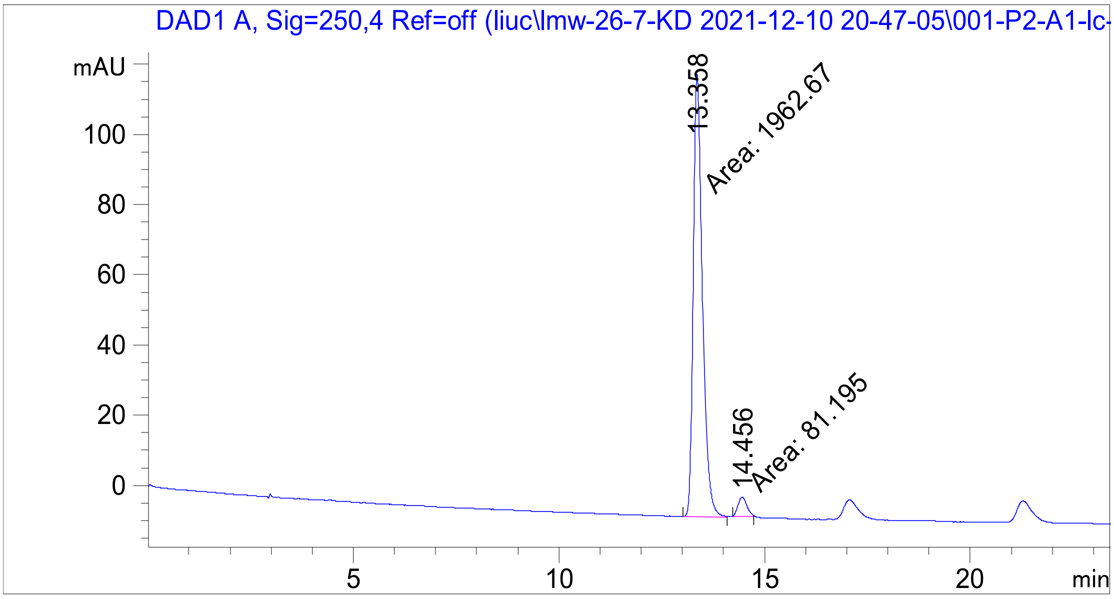

#	Time	Area	Height	Width	Area%	Symmetry
1	13.358	1962.7	126	0.2596	96.027	0.669
2	14.456	81.2	5.5	0.247	3.973	0.848

3da-racmic

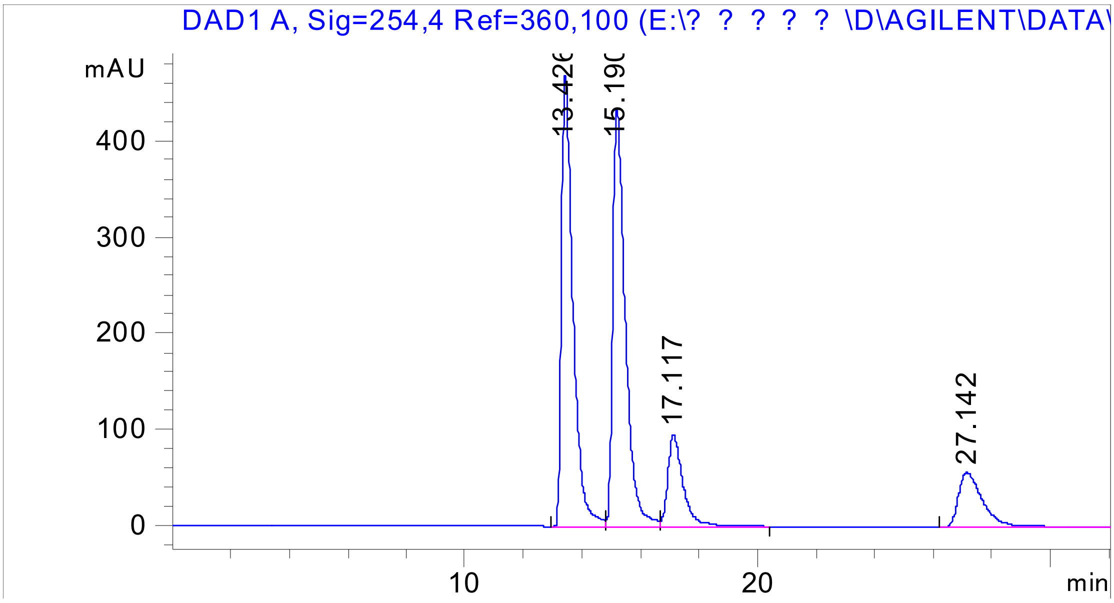

#	Time	Area	Height	Width	Area%	Symmetry
1	13.426	12792.9	468.2	0.4008	39.520	0.429
2	15.19	12975.7	432	0.4444	40.084	0.453
3	17.117	3455.1	95.6	0.5336	10.674	0.525
4	27.142	3147.3	56.6	0.9261	9.723	0.514

3da-chiral

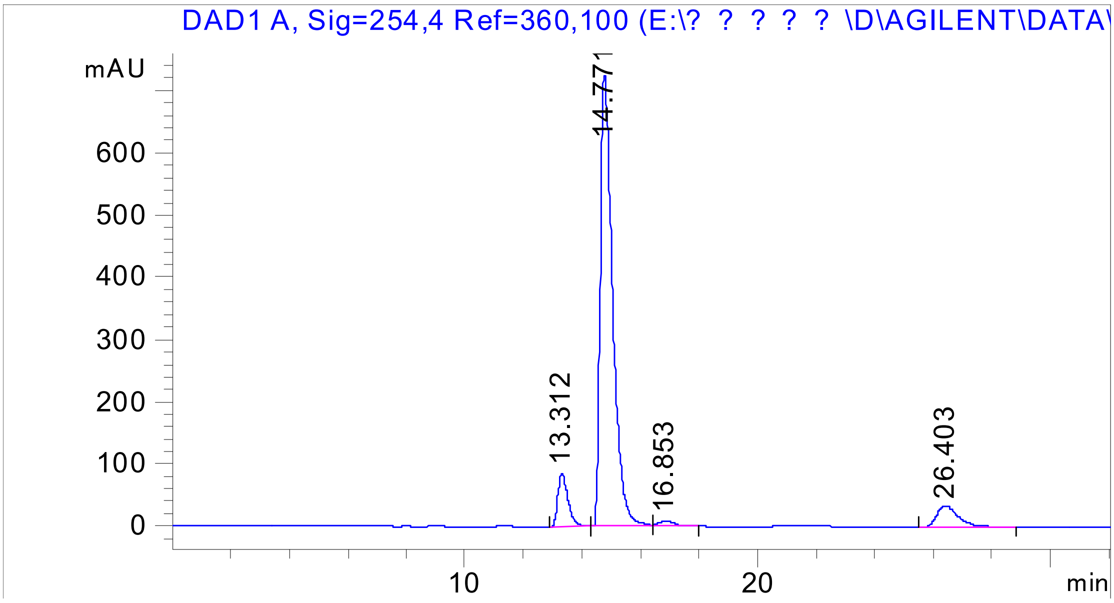

#	Time	Area	Height	Width	Area%	Symmetry
1	13.312	2037.8	85.9	0.3628	8.112	0.62
2	14.771	21132	722.9	0.437	84.122	0.491
3	16.853	220.1	7.1	0.4783	0.876	0.632
4	26.403	1730.7	34	0.7707	6.890	0.576

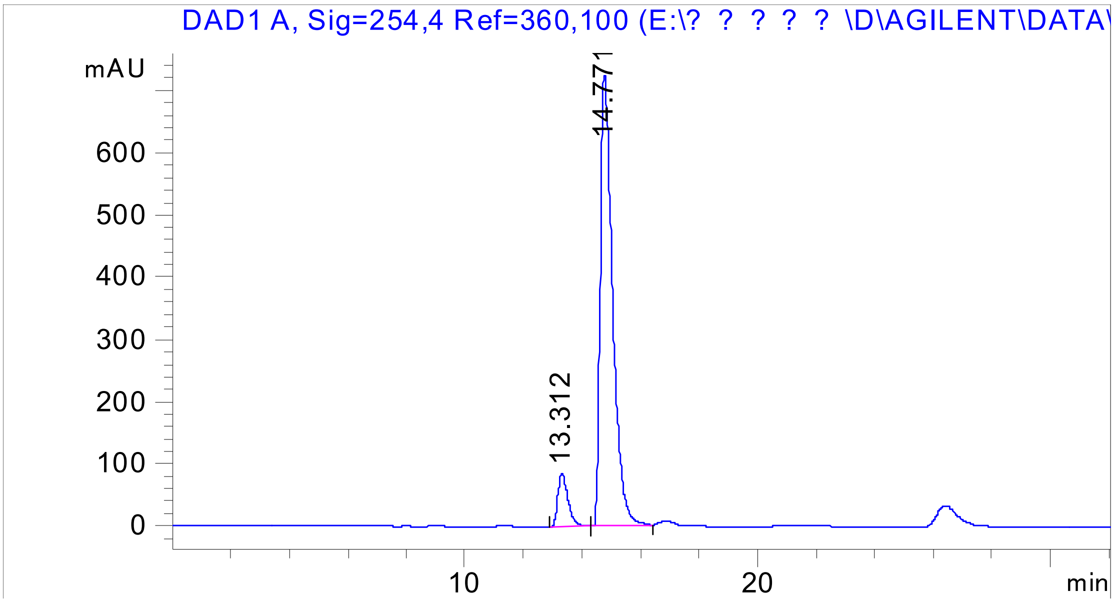

#	Time	Area	Height	Width	Area%	Symmetry
1	13.312	2037.8	85.9	0.3628	8.795	0.62
2	14.771	21132	722.9	0.437	91.205	0.491

3ea-racmic

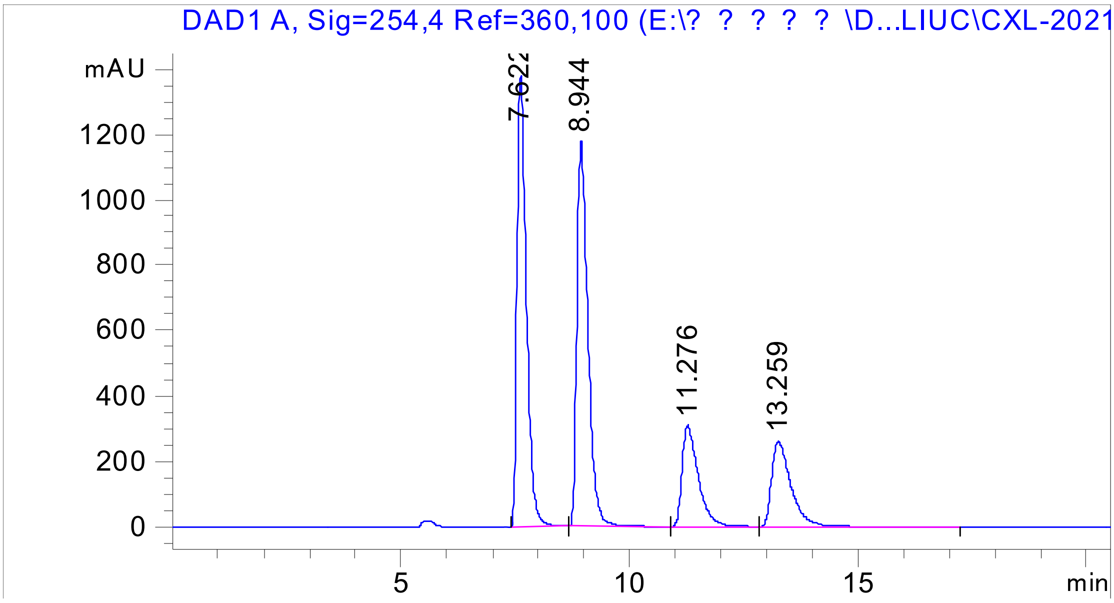

#	Time	Area	Height	Width	Area%	Symmetry
1	7.622	19601.2	1378.9	0.2178	35.871	0.562
2	8.944	19660.8	1179.3	0.2525	35.980	0.555
3	11.276	7666.6	309	0.3714	14.030	0.489
4	13.259	7715.2	261	0.4409	14.119	0.483

3ea-chiral

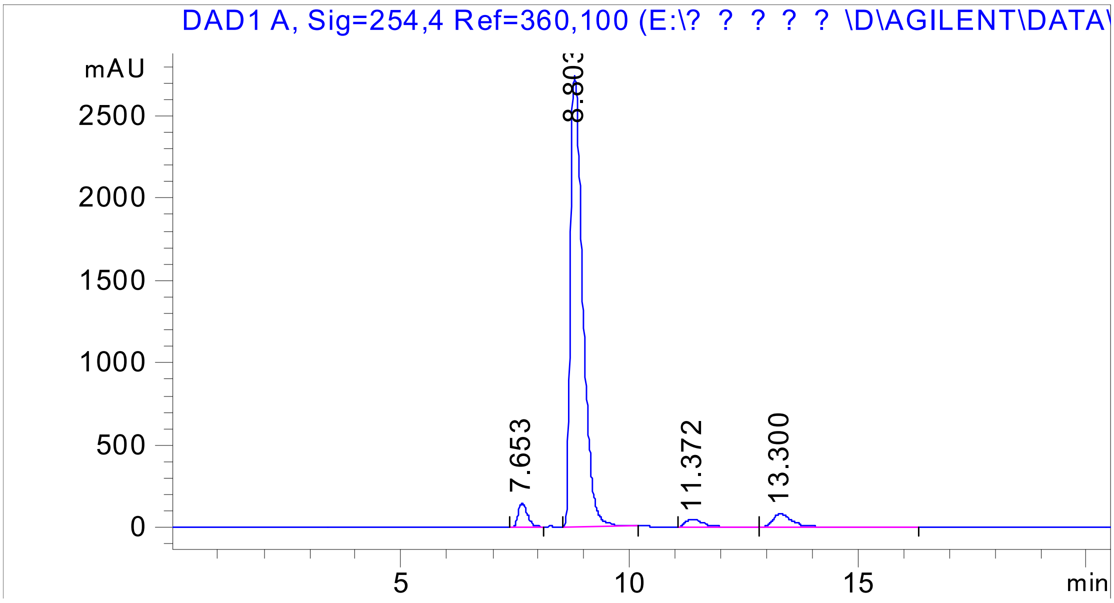

#	Time	Area	Height	Width	Area%	Symmetry
1	7.653	2048.5	150.4	0.2109	3.581	0.654
2	8.803	51552.9	2741.1	0.2834	90.108	0.466
3	11.372	1332.8	51	0.3968	2.330	0.529
4	13.3	2277.8	78.5	0.4364	3.981	0.546

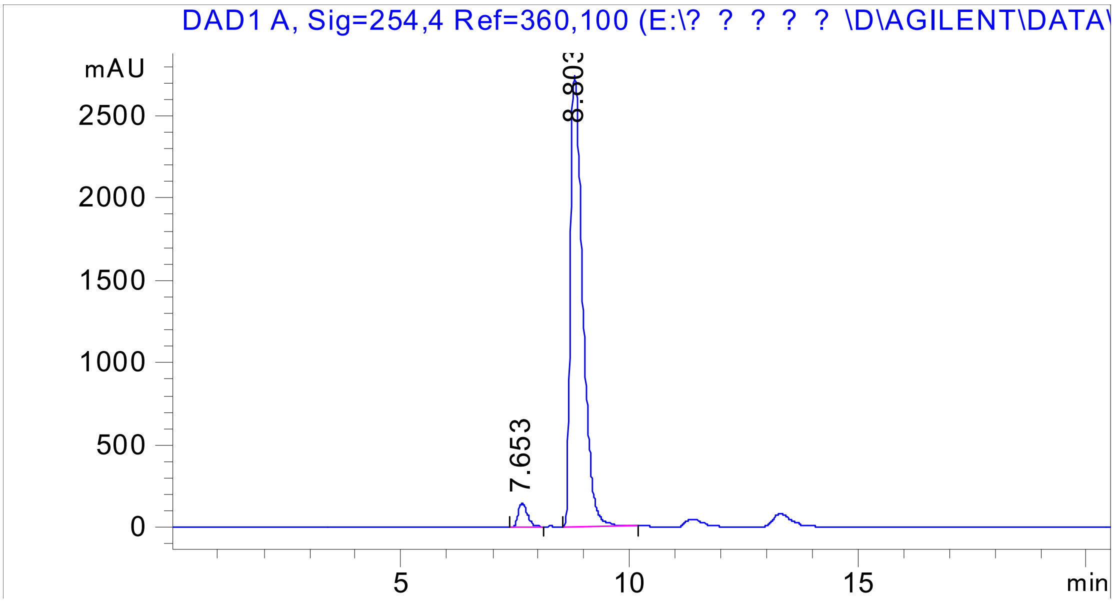

#	Time	Area	Height	Width	Area%	Symmetry
1	7.653	2048.5	150.4	0.2109	3.822	0.654
2	8.803	51552.9	2741.1	0.2834	96.178	0.466

3fa-racmic

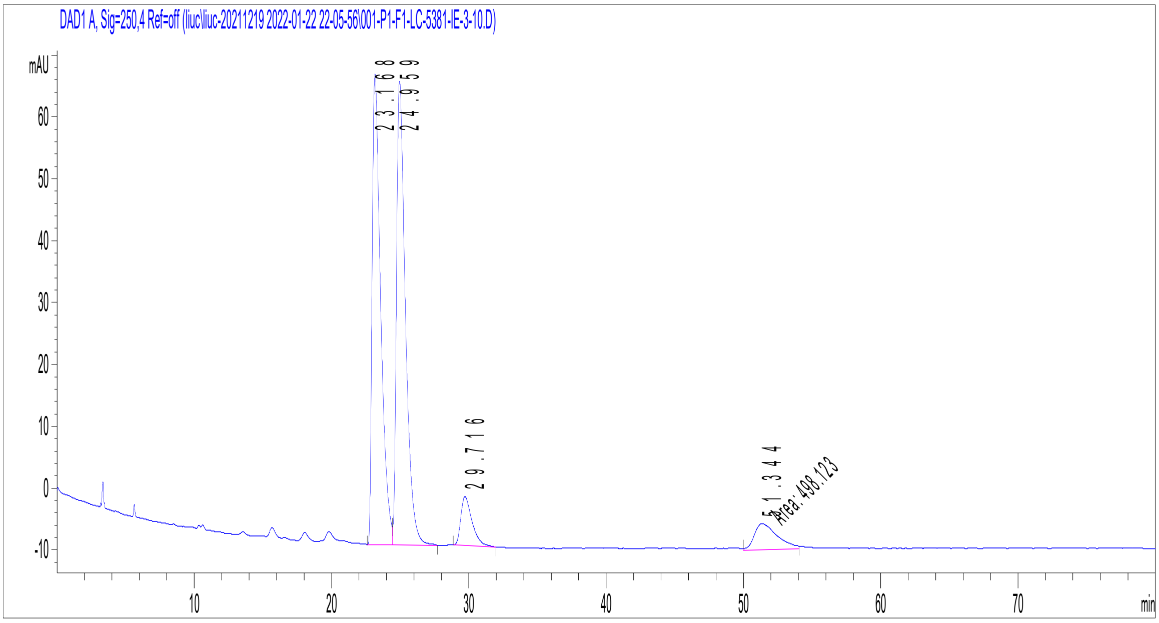

#	Time	Area	Height	Width	Area%	Symmetry
1	23.168	3303.5	76.1	0.6543	43.143	0.467
2	24.959	3400.7	75	0.6752	44.412	0.489
3	29.716	454.7	7.9	0.8013	5.939	0.54
4	51.344	498.1	4.3	1.953	6.505	0.505

3fa-chiral

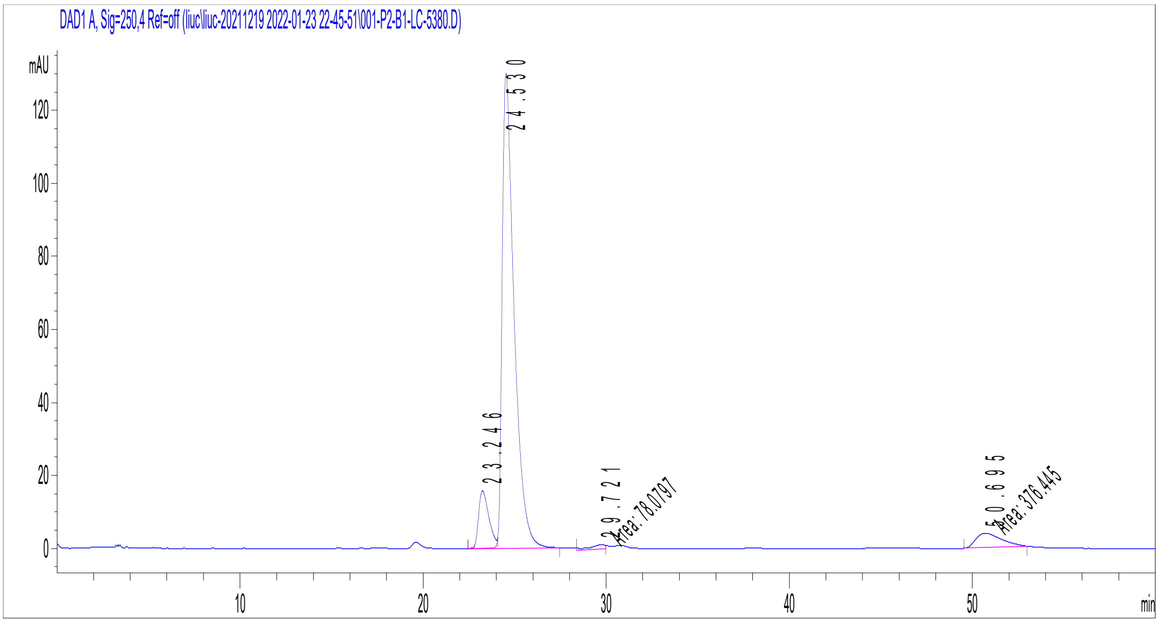

#	Time	Area	Height	Width	Area%	Symmetry
1	23.246	616.8	15.8	0.5926	8.876	0.595
2	24.53	5877.8	129.9	0.6738	84.583	0.757
3	29.721	78.1	1.3	1.0258	1.124	3.231
4	50.695	376.4	3.9	1.5992	5.417	0.547

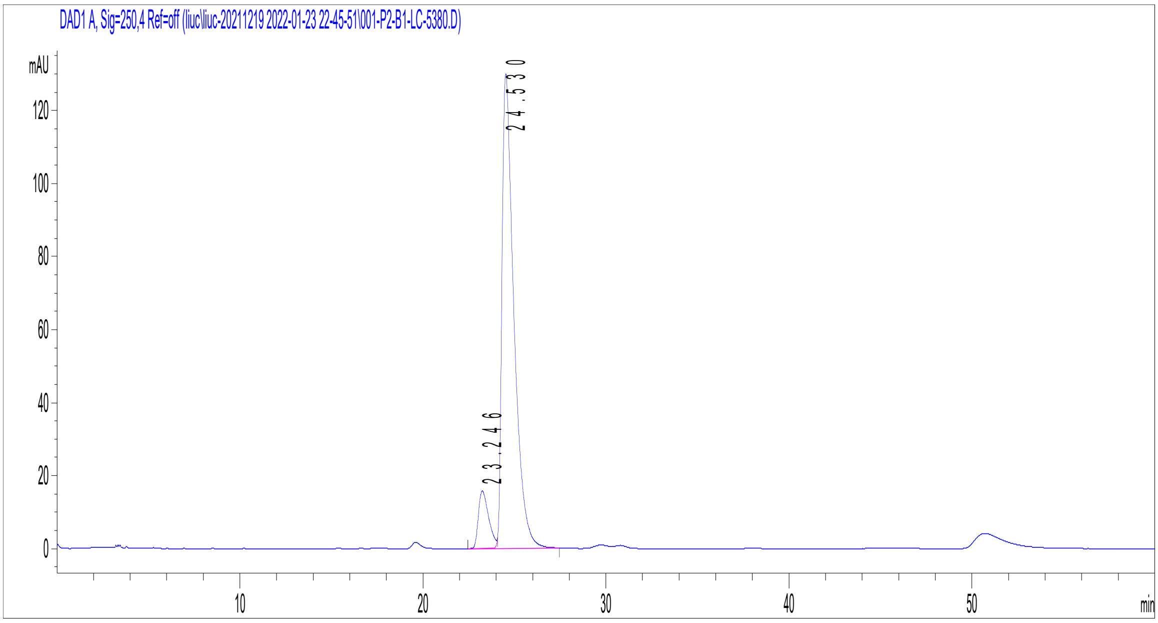

#	Time	Area	Height	Width	Area%	Symmetry
1	23.246	616.8	15.8	0.5926	9.497	0.595
2	24.53	5877.8	129.9	0.6738	90.503	0.757

3ga-racmic

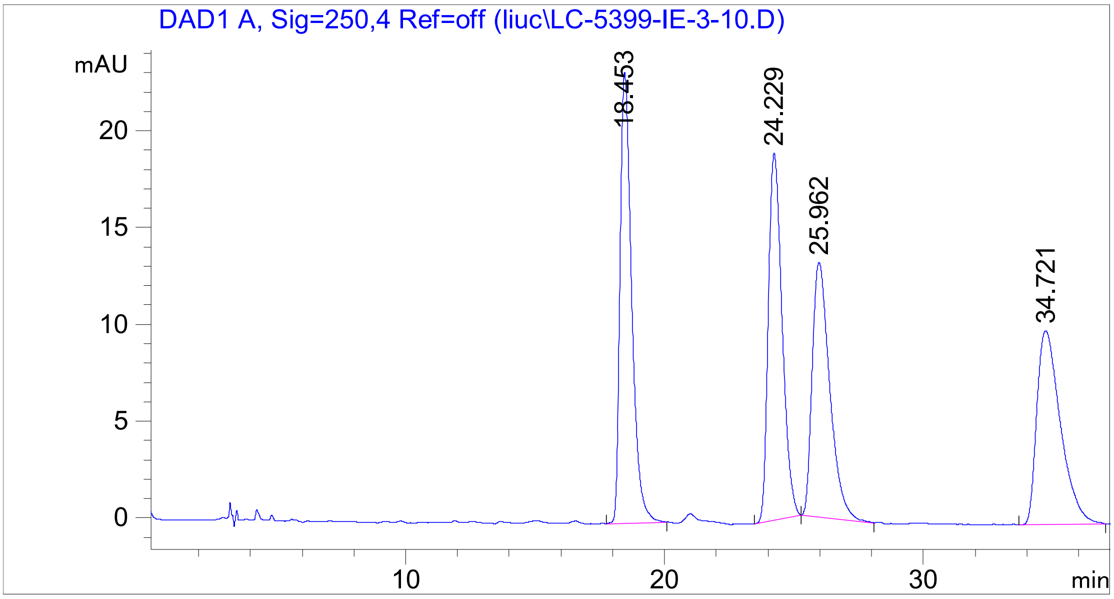

#	Time	Area	Height	Width	Area%	Symmetry
1	18.453	726.3	23.3	0.4758	27.060	0.595
2	24.229	701	19	0.5751	26.116	0.679
3	25.962	620.5	13.2	0.7098	23.115	0.59
4	34.721	636.4	10	0.9166	23.708	0.56

3ga-chiral

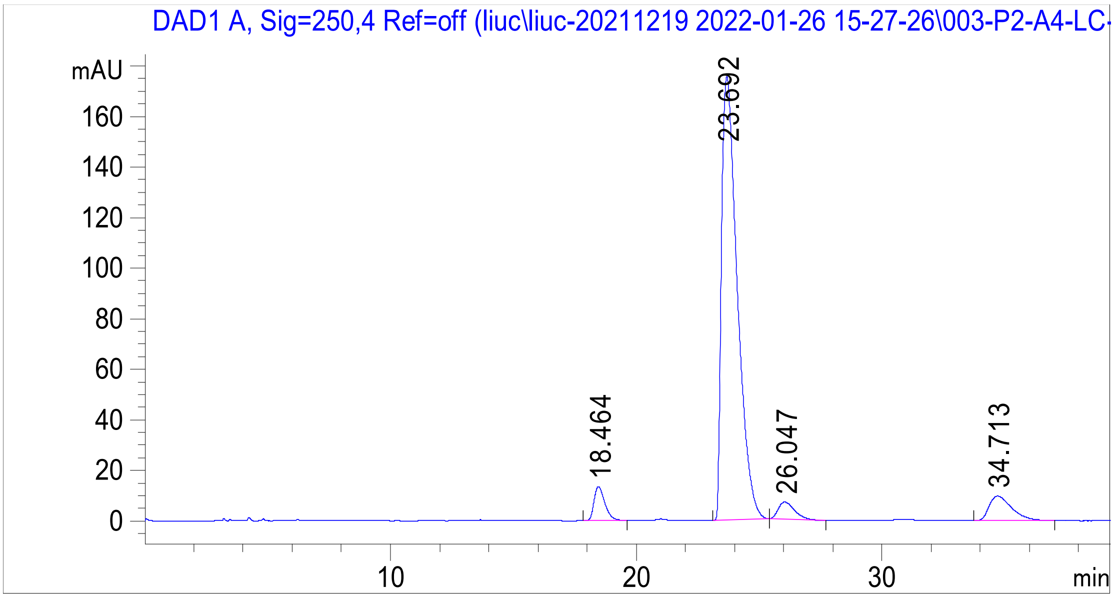

#	Time	Area	Height	Width	Area%	Symmetry
1	18.464	412.2	13.5	0.4673	4.498	0.638
2	23.692	7814.9	175.6	0.6753	85.290	0.463
3	26.047	315.4	6.8	0.6853	3.442	0.619
4	34.713	620.3	9.7	0.9221	6.770	0.565

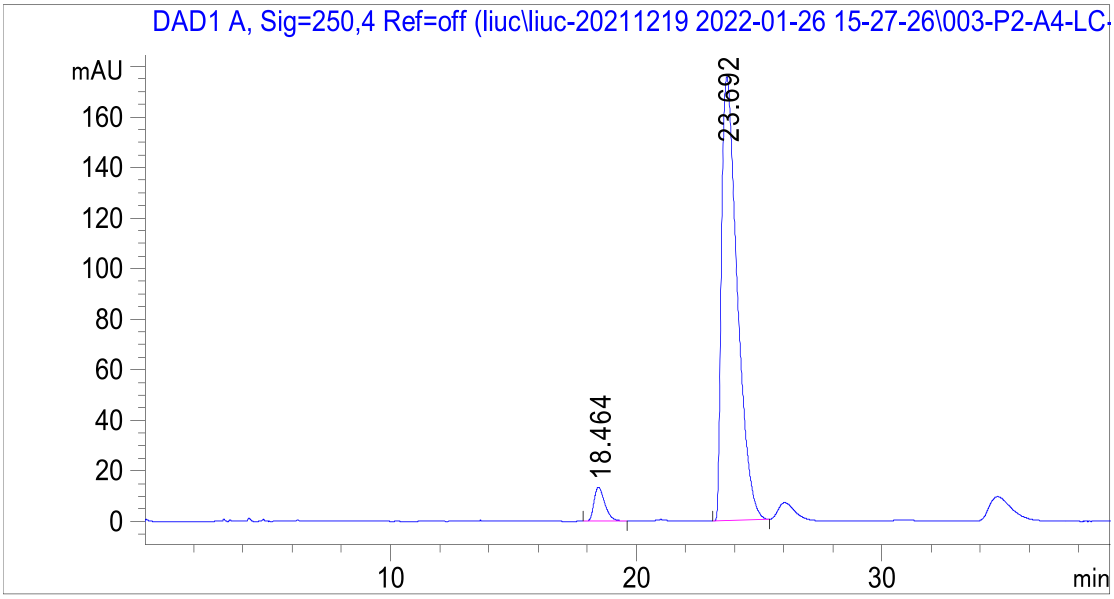

#	Time	Area	Height	Width	Area%	Symmetry
1	18.464	412.2	13.5	0.4673	5.010	0.638
2	23.692	7814.9	175.6	0.6753	94.990	0.463

3ha-racmic

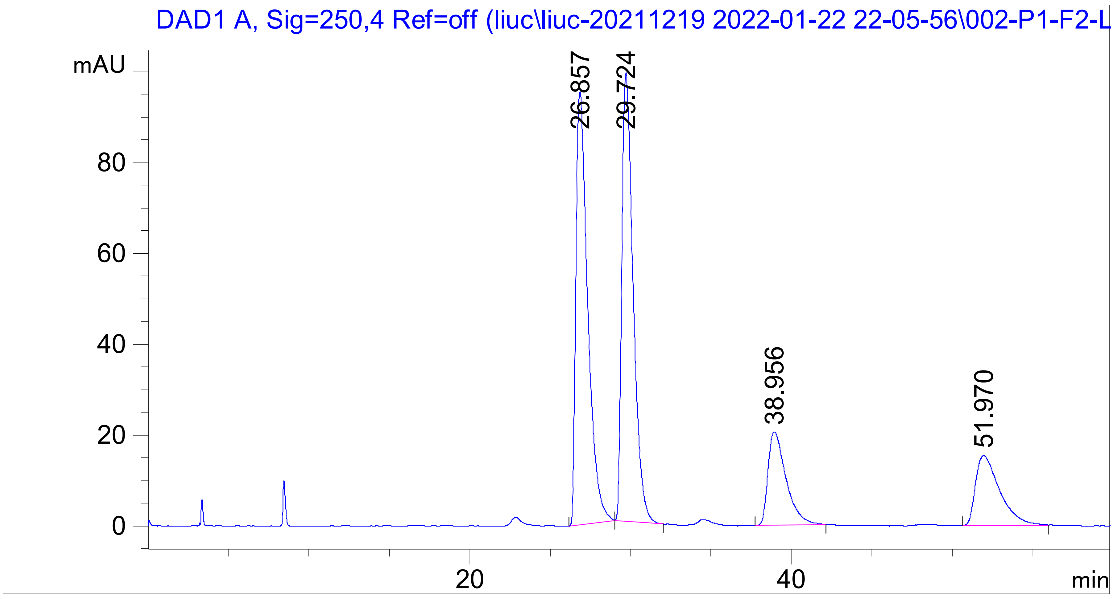

#	Time	Area	Height	Width	Area%	Symmetry
1	26.857	4844.6	95.1	0.764	37.608	0.461
2	29.724	4831.9	98.7	0.7431	37.509	0.534
3	38.956	1604.8	20.6	1.1263	12.457	0.512
4	51.97	1600.8	15.4	1.3908	12.426	0.471

3ha-chiral

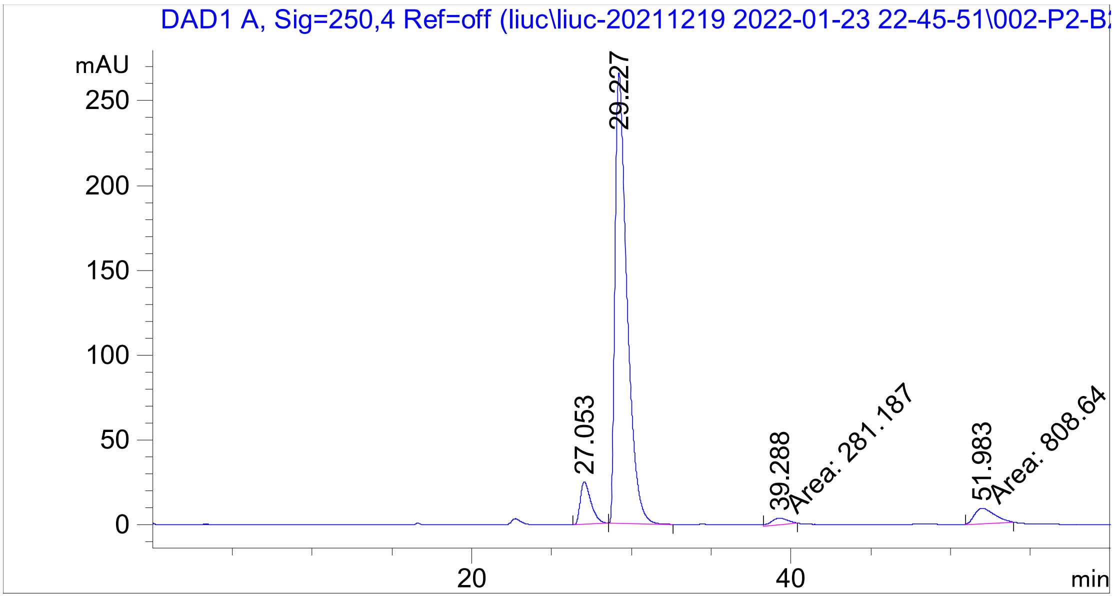

#	Time	Area	Height	Width	Area%	Symmetry
1	27.053	1167.4	25.1	0.6942	7.248	0.553
2	29.227	13849.4	265.7	0.7781	85.986	0.445
3	39.288	281.2	3.9	0.8494	1.746	1.034
4	51.983	808.6	9.1	1.4758	5.021	0.604

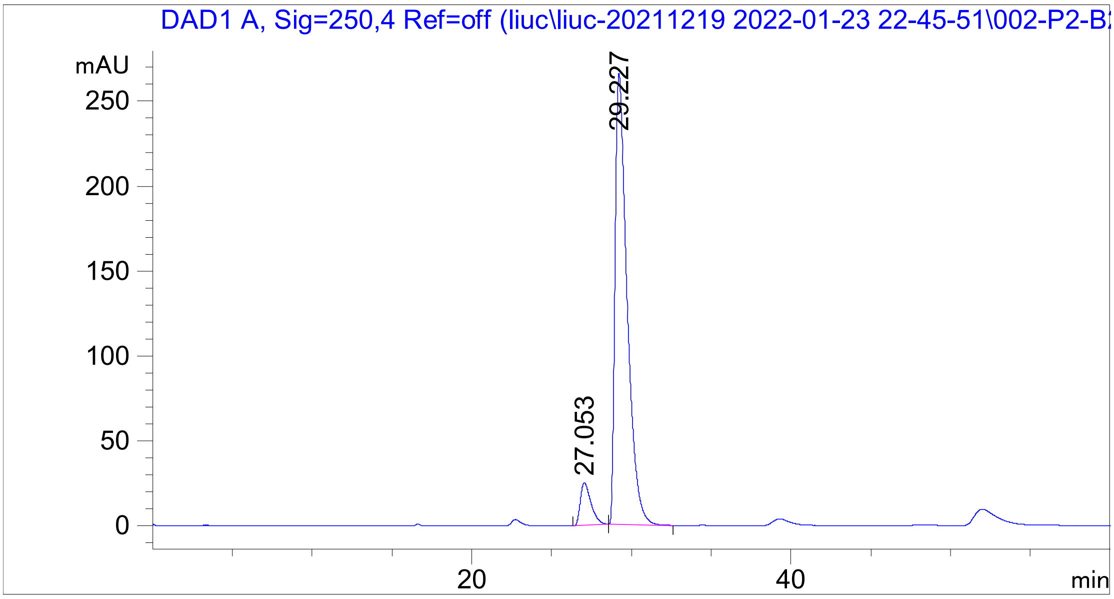

#	Time	Area	Height	Width	Area%	Symmetry
1	27.053	1167.4	25.1	0.6942	7.774	0.553
2	29.227	13849.4	265.7	0.7781	92.226	0.445

3ia-racmic

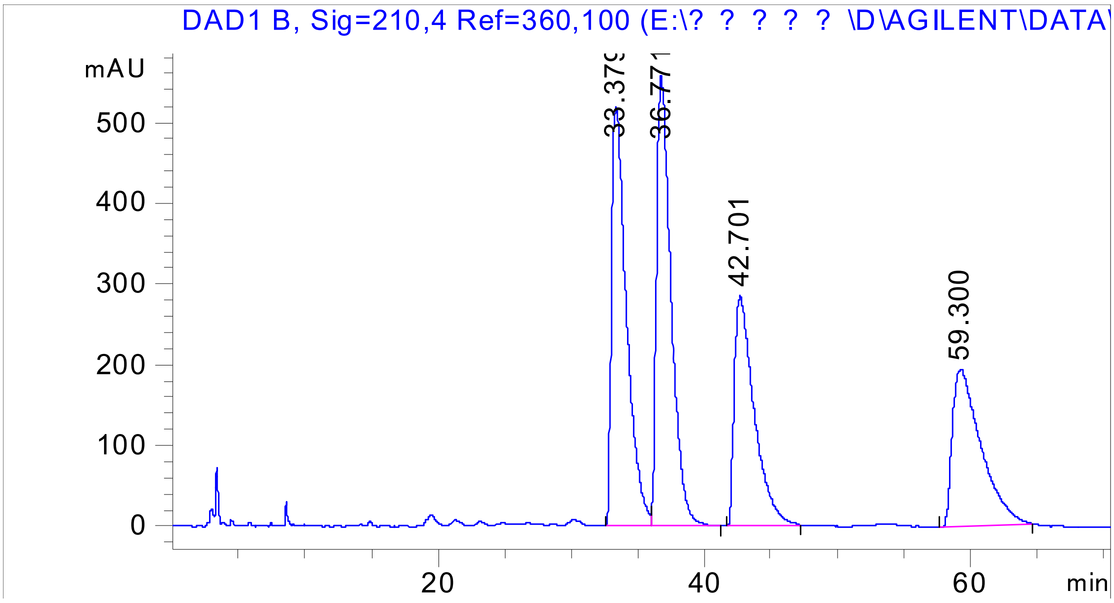

#	Time	Area	Height	Width	Area%	Symmetry
1	33.378	15491	196.4	1.1562	29.304	0.407
2	36.77	16021.3	211.4	1.1322	30.307	0.438
3	42.702	10638.6	102.5	1.4944	20.125	0.373
4	59.300	10712.3	70.2	2.1457	20.264	0.371

3ia-chiral

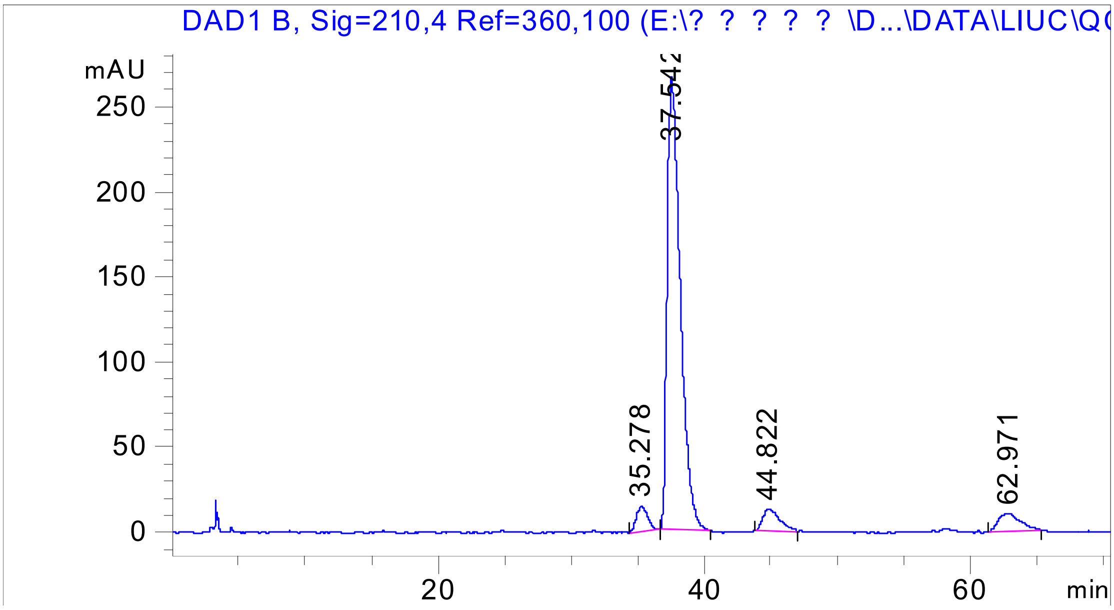

#	Time	Area	Height	Width	Area%	Symmetry
1	35.278	330.9	5.5	0.8741	3.953	0.716
2	37.542	6916.1	100.1	1.0321	82.620	0.462
3	44.822	491.8	5.1	1.5962	5.875	0.585
4	62.971	632.2	4.8	1.5591	7.552	0.637

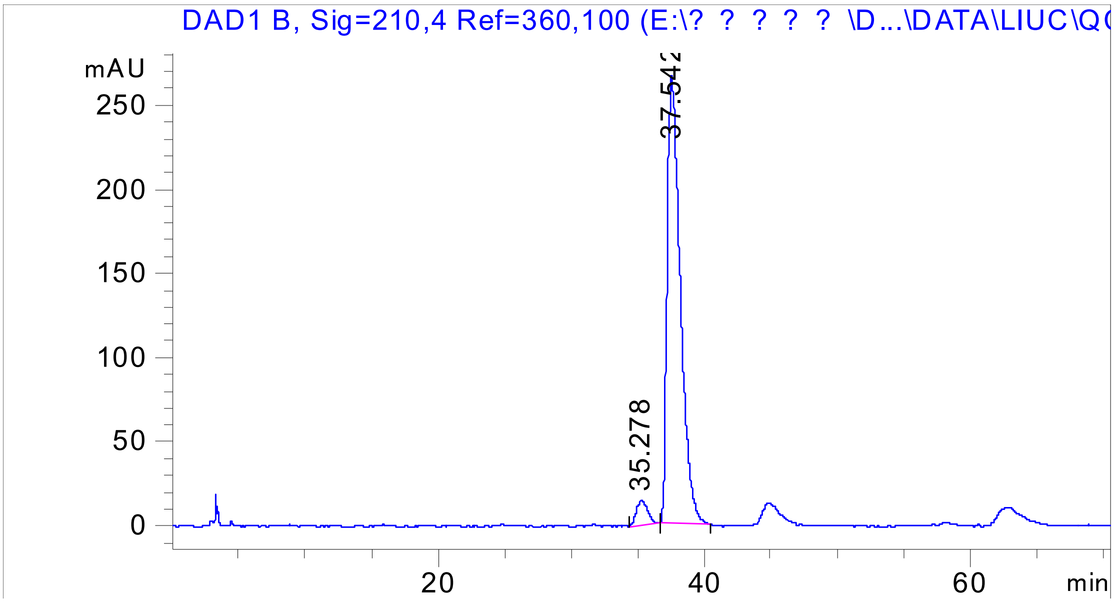

#	Time	Area	Height	Width	Area%	Symmetry
1	35.278	330.9	5.5	0.8741	4.566	0.716
2	37.542	6916.1	100.1	1.0321	95.434	0.462

3ja-racmic

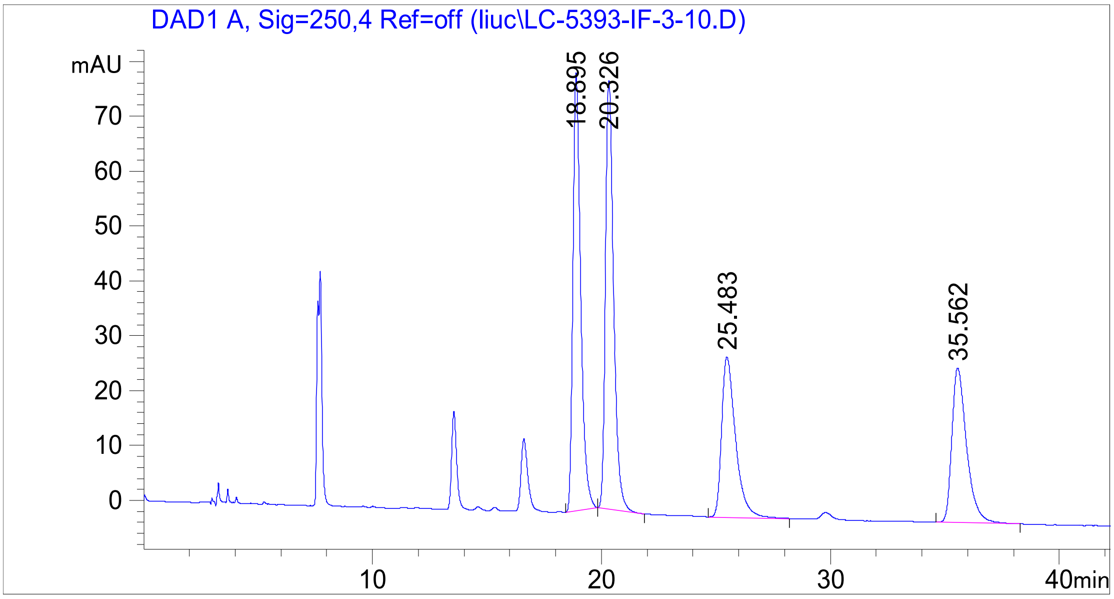

#	Time	Area	Height	Width	Area%	Symmetry
1	18.895	1883.9	79.8	0.3573	29.821	0.644
2	20.326	1933.8	78.1	0.377	30.612	0.663
3	25.483	1234	29.2	0.6334	19.533	0.568
4	35.562	1265.7	28.2	0.6782	20.035	0.602

3ja-chiral

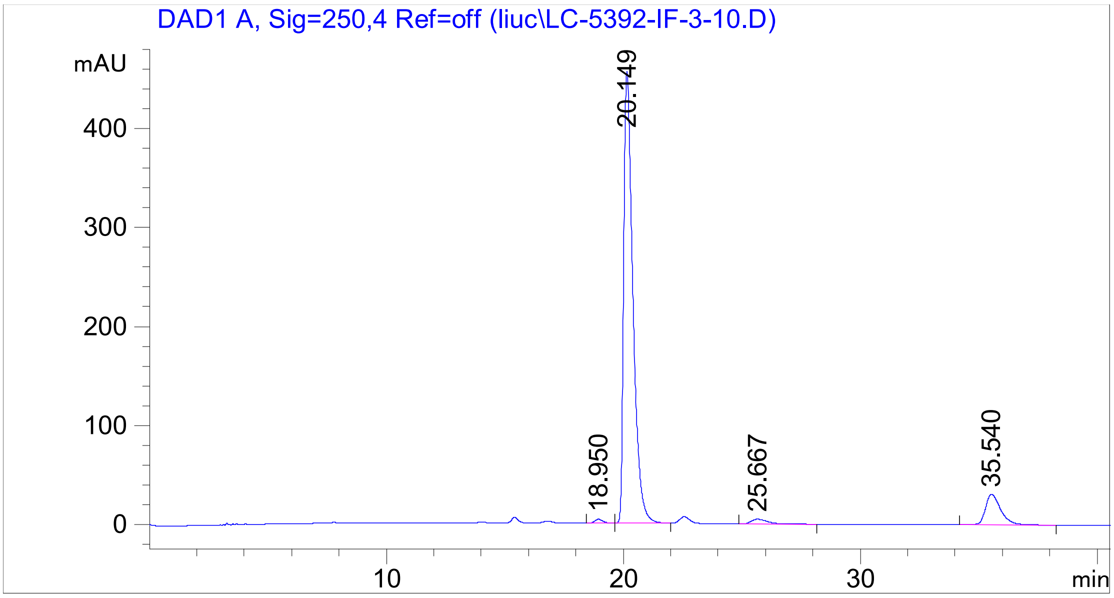

#	Time	Area	Height	Width	Area%	Symmetry
1	18.95	96.2	4	0.3613	0.688	0.755
2	20.149	12257.8	455.6	0.4059	87.667	0.479
3	25.667	246.7	4.8	0.7106	1.764	0.534
4	35.54	1381.6	30.9	0.6699	9.881	0.636

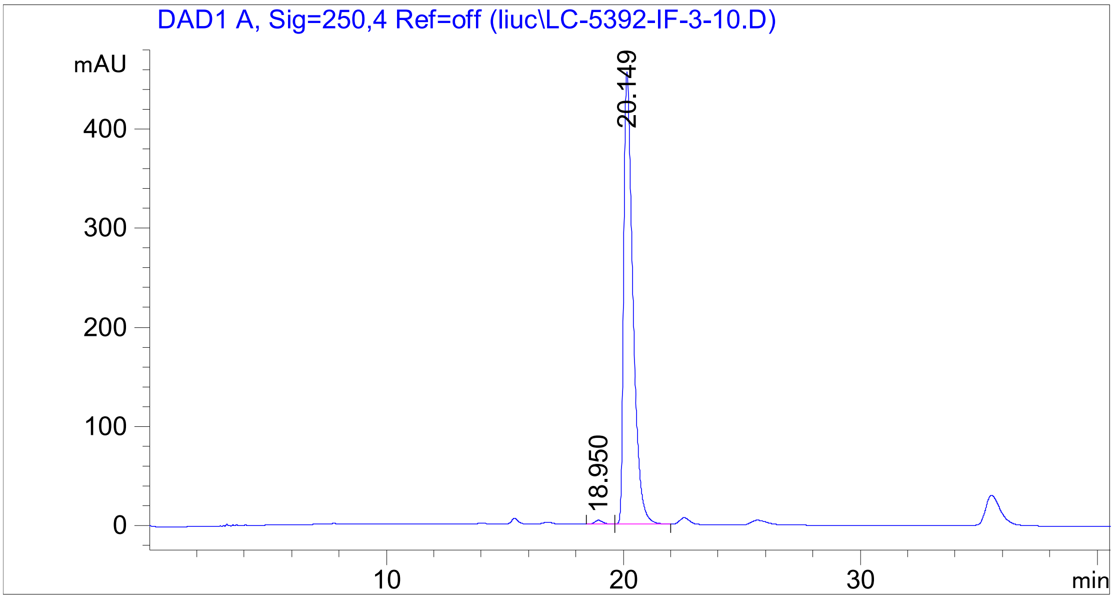

#	Time	Area	Height	Width	Area%	Symmetry
1	18.95	96.2	4	0.3613	0.779	0.755
2	20.149	12257.8	455.6	0.4059	99.221	0.479

3ka-racmic

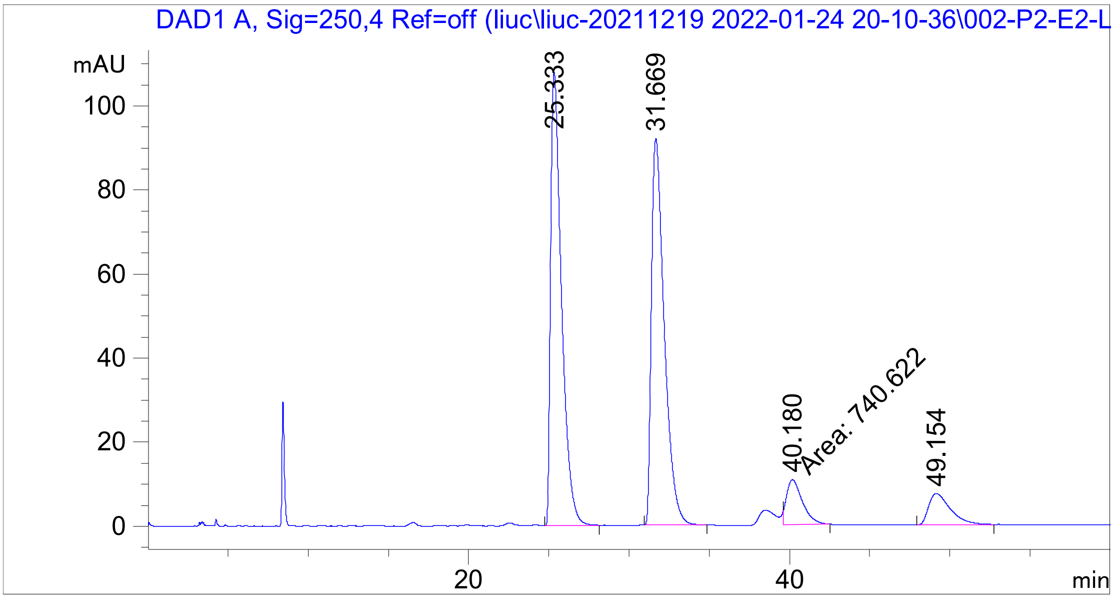

#	Time	Area	Height	Width	Area%	Symmetry
1	25.333	5114.7	107.9	0.7073	43.761	0.442
2	31.669	5130.8	92	0.8366	43.898	0.474
3	40.18	740.6	10.8	1.1458	6.337	0.542
4	49.154	701.8	7.5	1.1129	6.005	0.515

3ka-chiral

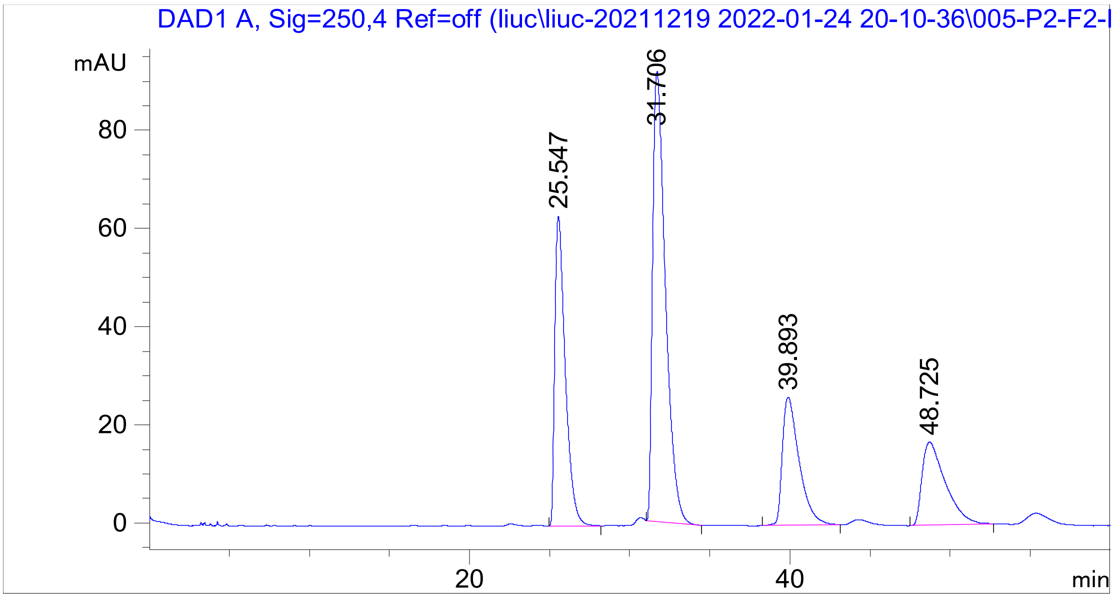

#	Time	Area	Height	Width	Area%	Symmetry
1	25.547	2867.2	63.1	0.6821	24.731	0.469
2	31.706	5103.6	91.8	0.8368	44.021	0.473
3	39.893	1914.9	26.1	1.0774	16.517	0.487
4	48.725	1707.8	17	1.3733	14.731	0.436

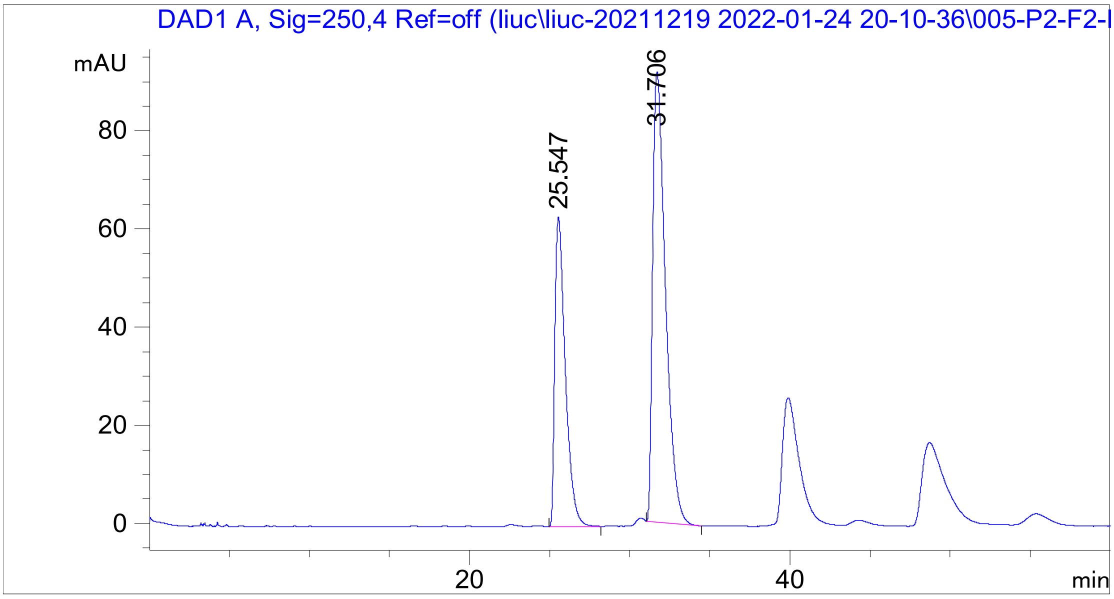

#	Time	Area	Height	Width	Area%	Symmetry
1	25.547	2867.2	63.1	0.6821	35.971	0.469
2	31.706	5103.6	91.8	0.8368	64.029	0.473

3la-racmic

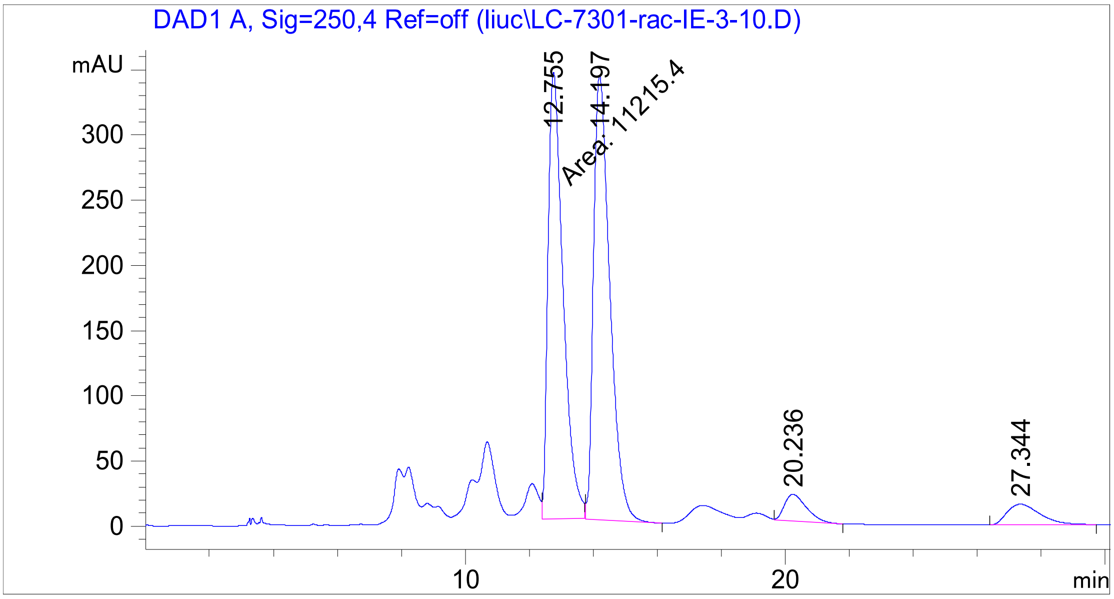

#	Time	Area	Height	Width	Area%	Symmetry
1	12.755	11215.4	342.6	0.5456	44.173	0.54
2	14.197	12081.4	340.7	0.5477	47.584	0.552
3	20.236	979.5	20.5	0.6978	3.858	0.584
4	27.344	1113.2	15.8	0.9752	4.385	0.622

3la-chiral

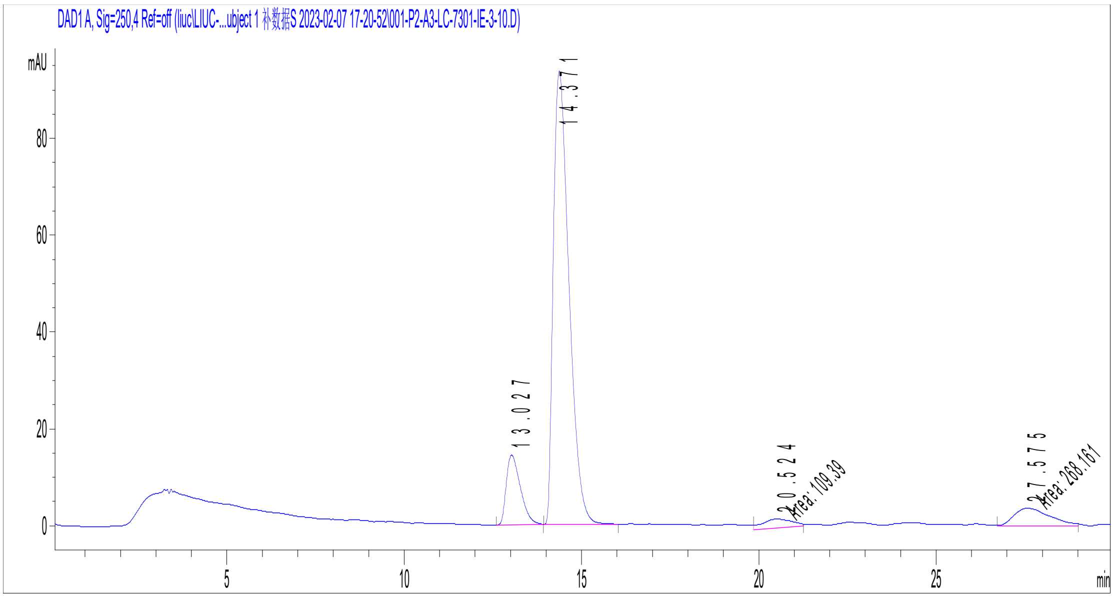

#	Time	Area	Height	Width	Area%	Symmetry
1	13.027	399.1	14.4	0.4249	10.930	0.578
2	14.371	2874.4	93.7	0.4781	78.729	0.568
3	20.524	109.4	1.9	0.9766	2.996	1.05
4	27.575	268.2	3.7	1.1943	7.345	0.587

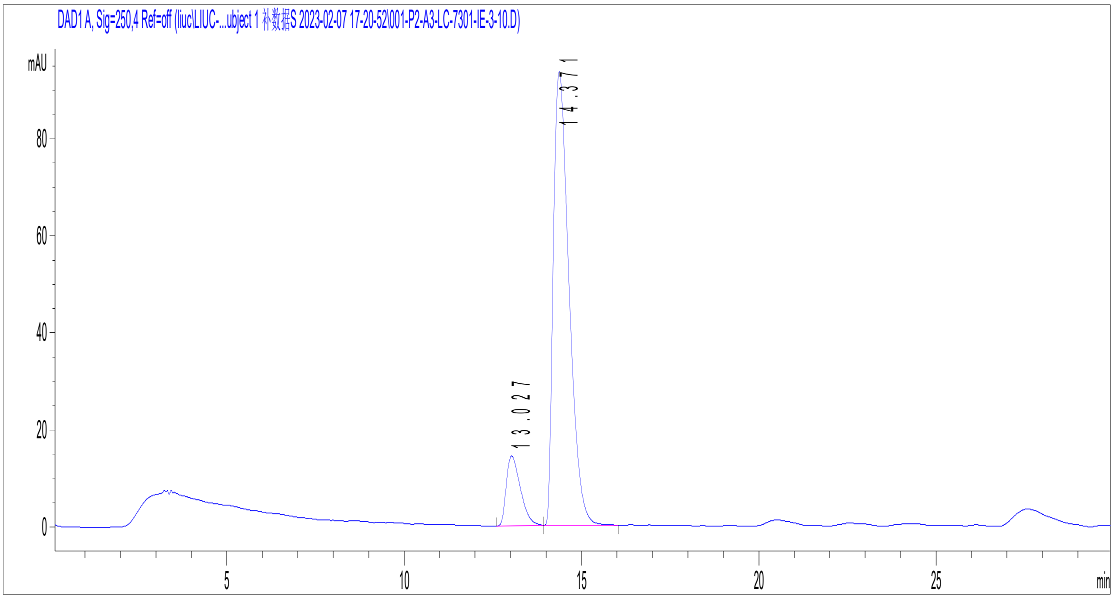

#	Time	Area	Height	Width	Area%	Symmetry
1	13.027	399.1	14.4	0.4249	12.191	0.578
2	14.371	2874.4	93.7	0.4781	87.809	0.568

3ma-racmic

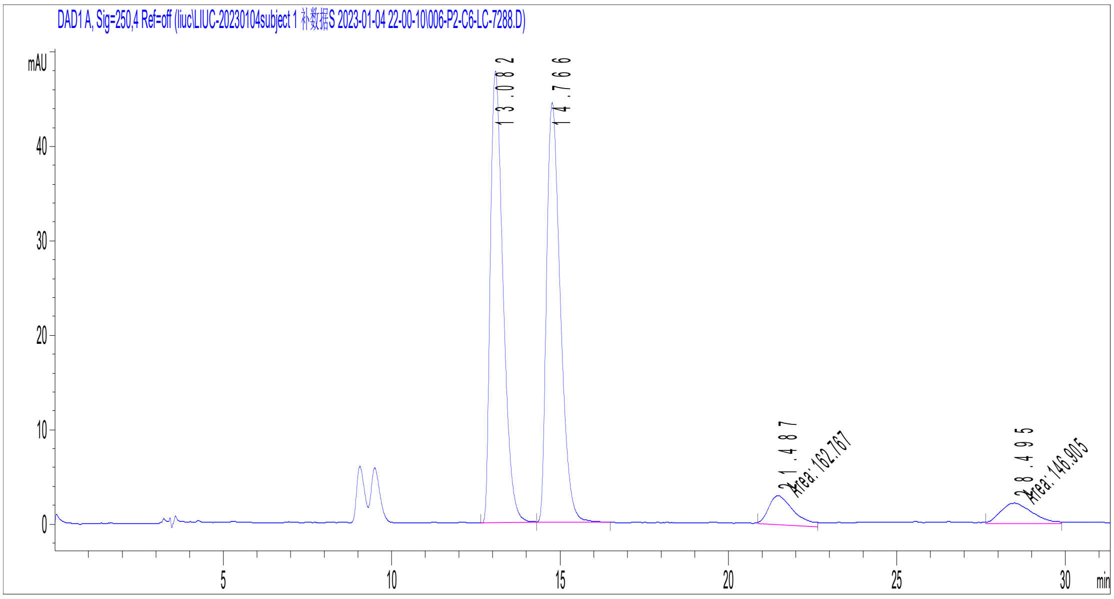

#	Time	Area	Height	Width	Area%	Symmetry
1	13.082	1256.1	47.8	0.4104	44.452	0.618
2	14.766	1259.9	44.4	0.441	44.589	0.633
3	21.487	162.8	3.1	0.8706	5.760	0.596
4	28.495	146.9	2.2	1.114	5.199	0.754

3ma-chiral

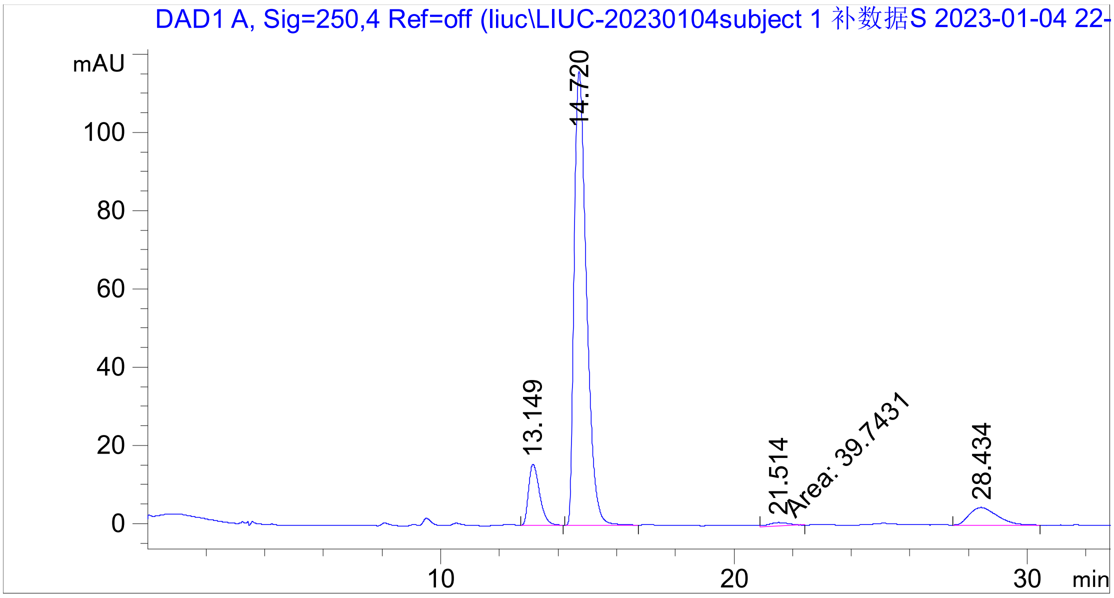

#	Time	Area	Height	Width	Area%	Symmetry
1	13.149	407.9	15.6	0.404	9.913	0.64
2	14.72	3371.8	115.9	0.4554	81.943	0.613
3	21.514	39.7	8.5E-1	0.7762	0.966	0.997
4	28.434	295.4	4.5	0.7808	7.179	0.719

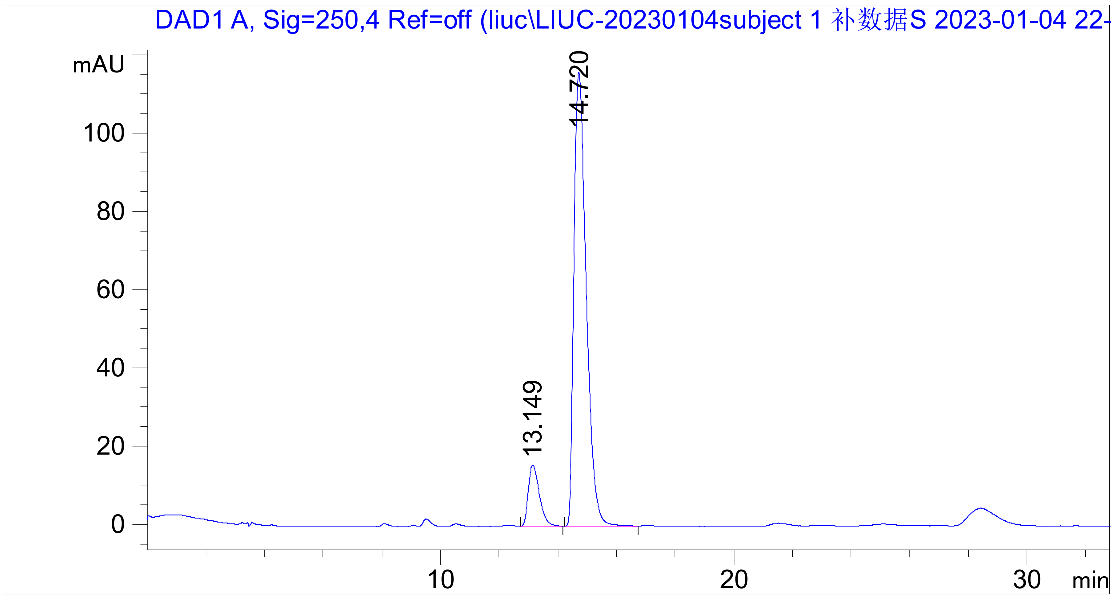

#	Time	Area	Height	Width	Area%	Symmetry
1	13.149	407.9	15.6	0.404	10.792	0.64
2	14.72	3371.8	115.9	0.4554	89.208	0.613

3na-racmic

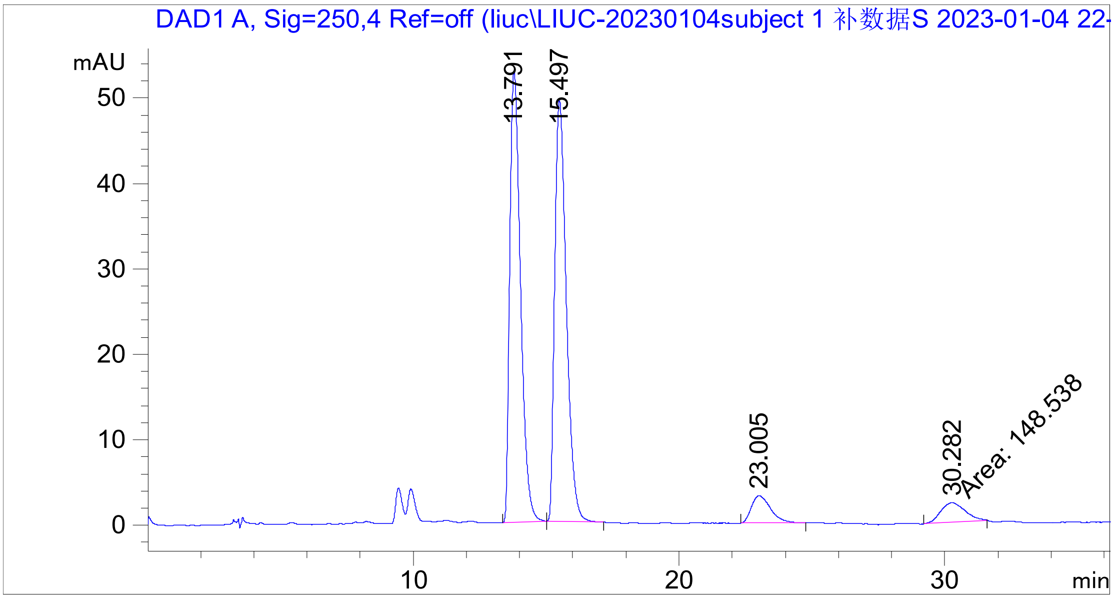

#	Time	Area	Height	Width	Area%	Symmetry
1	13.791	1485.9	52.8	0.438	45.276	0.616
2	15.497	1486.9	49.3	0.4701	45.307	0.627
3	23.005	160.5	3.2	0.6057	4.891	0.619
4	30.282	148.5	2.3	1.0743	4.526	0.863

3na-chiral

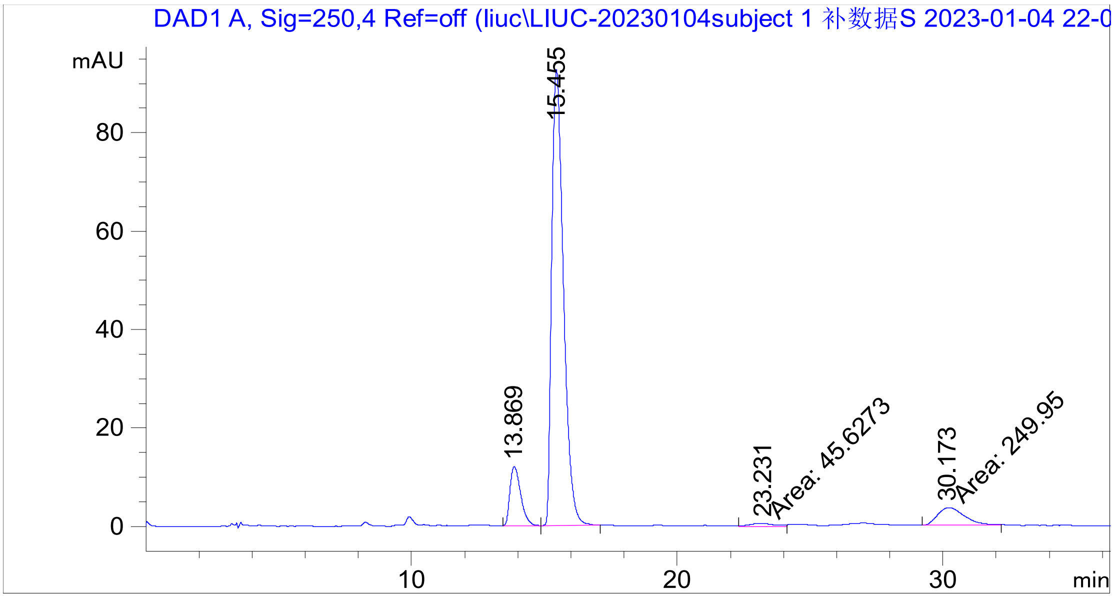

#	Time	Area	Height	Width	Area%	Symmetry
1	13.869	338.3	12	0.434	9.742	0.633
2	15.455	2839.1	92.6	0.4775	81.747	0.613
3	23.231	45.6	6.8E-1	1.1136	1.314	1.119
4	30.173	249.9	3.5	1.1759	7.197	0.572

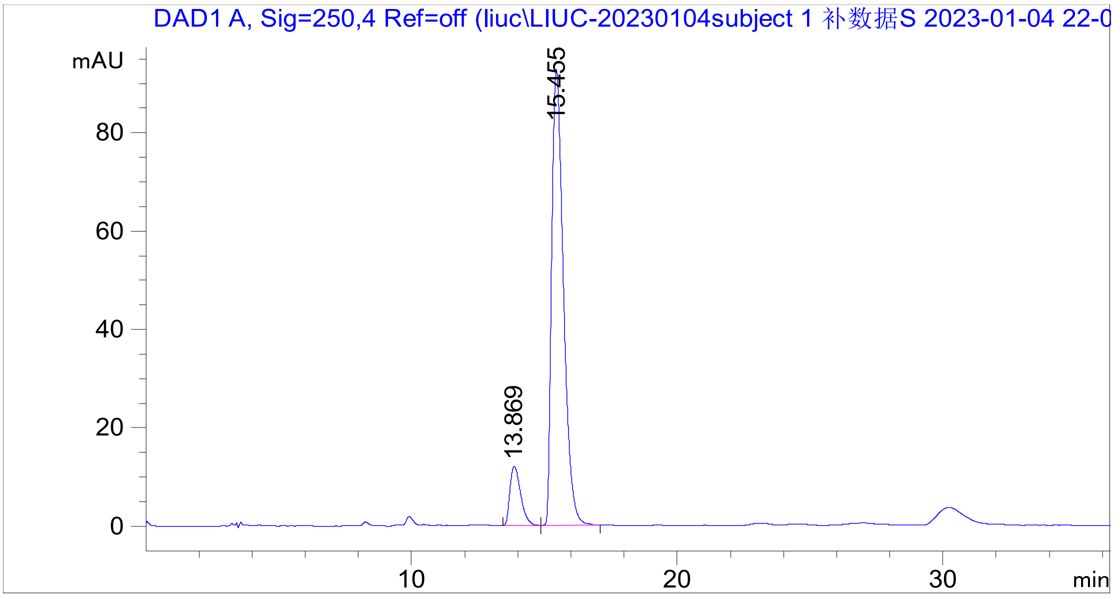

#	Time	Area	Height	Width	Area%	Symmetry
1	13.869	338.3	12	0.434	10.648	0.633
2	15.455	2839.1	92.6	0.4775	89.352	0.613

3oa-racmic

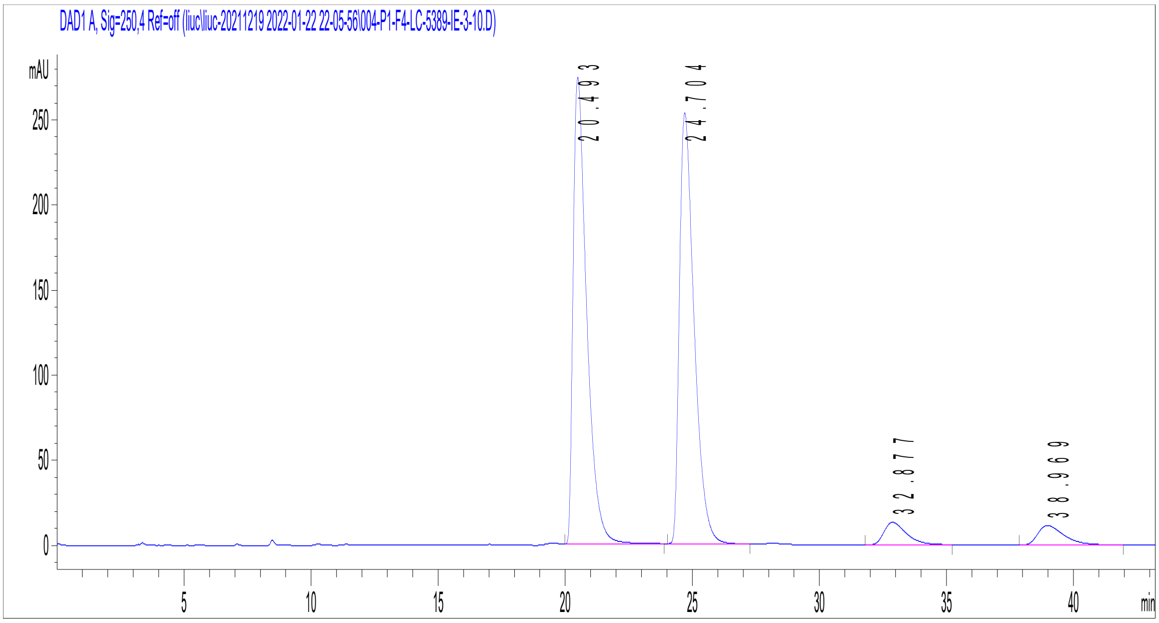

#	Time	Area	Height	Width	Area%	Symmetry
1	20.493	10202.2	274.4	0.5577	46.353	0.472
2	24.704	10155.8	253.1	0.6122	46.143	0.53
3	32.877	821	13.3	0.8965	3.730	0.576
4	38.969	830.6	11.5	0.9953	3.774	0.539

3oa-chiral

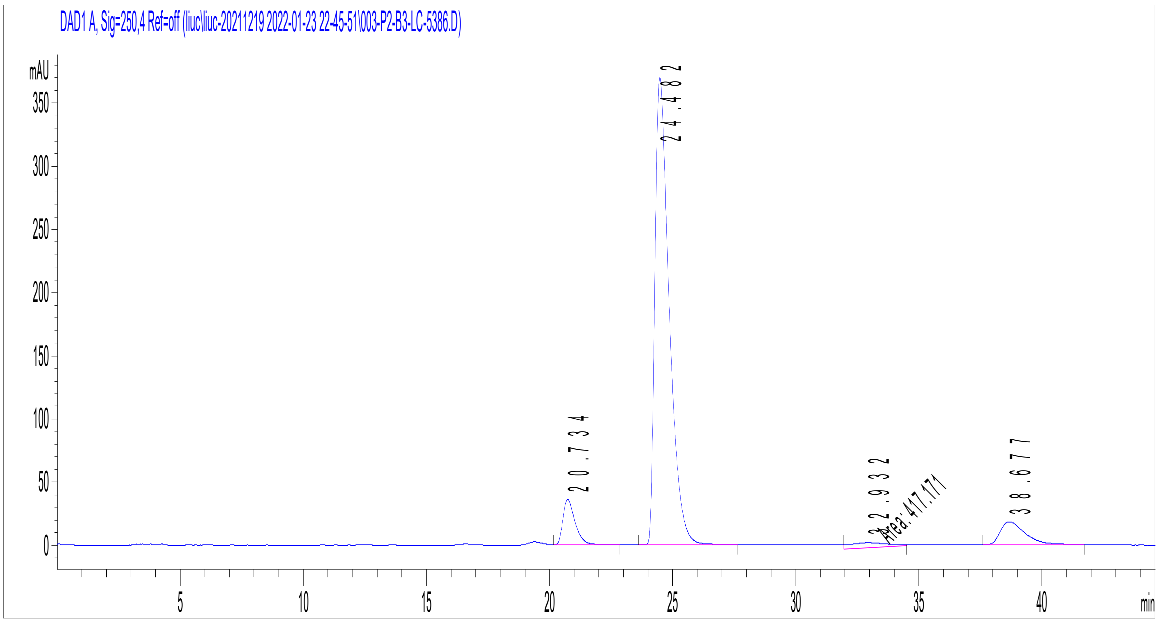

#	Time	Area	Height	Width	Area%	Symmetry
1	20.734	1246.7	36.1	0.5229	6.954	0.578
2	24.482	14963.3	370	0.6118	83.459	0.492
3	32.932	417.2	4.2	1.1535	2.327	1.107
4	38.677	1301.8	18.3	1.0124	7.261	0.529

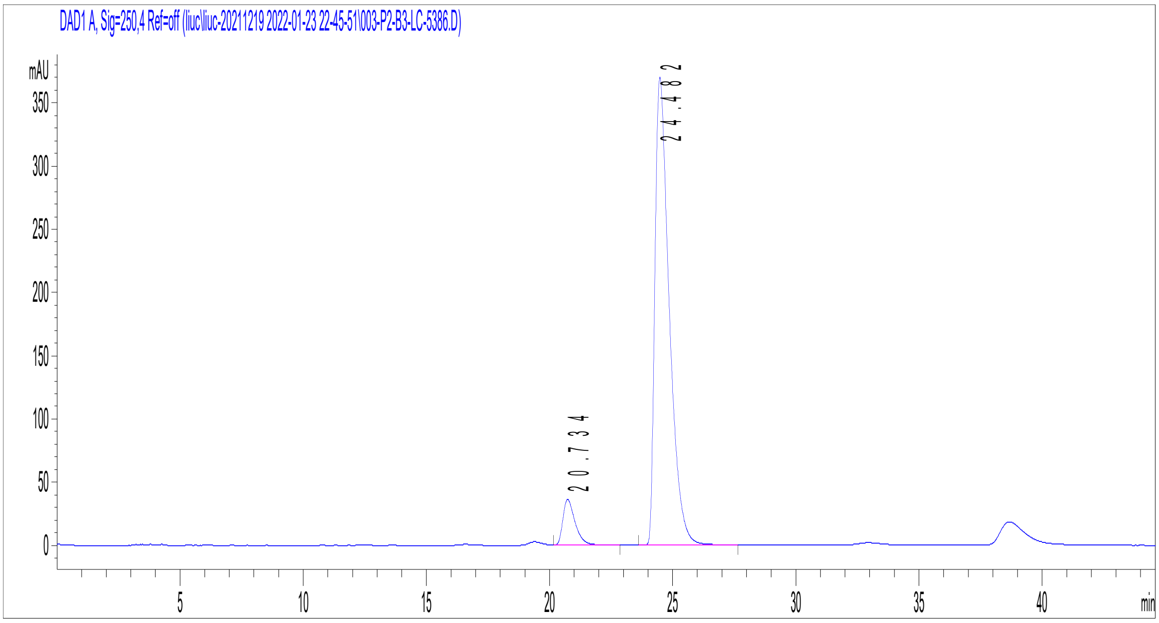

#	Time	Area	Height	Width	Area%	Symmetry
1	20.734	1246.7	36.1	0.5229	7.691	0.578
2	24.482	14963.3	370	0.6118	92.309	0.492

3eb-racmic

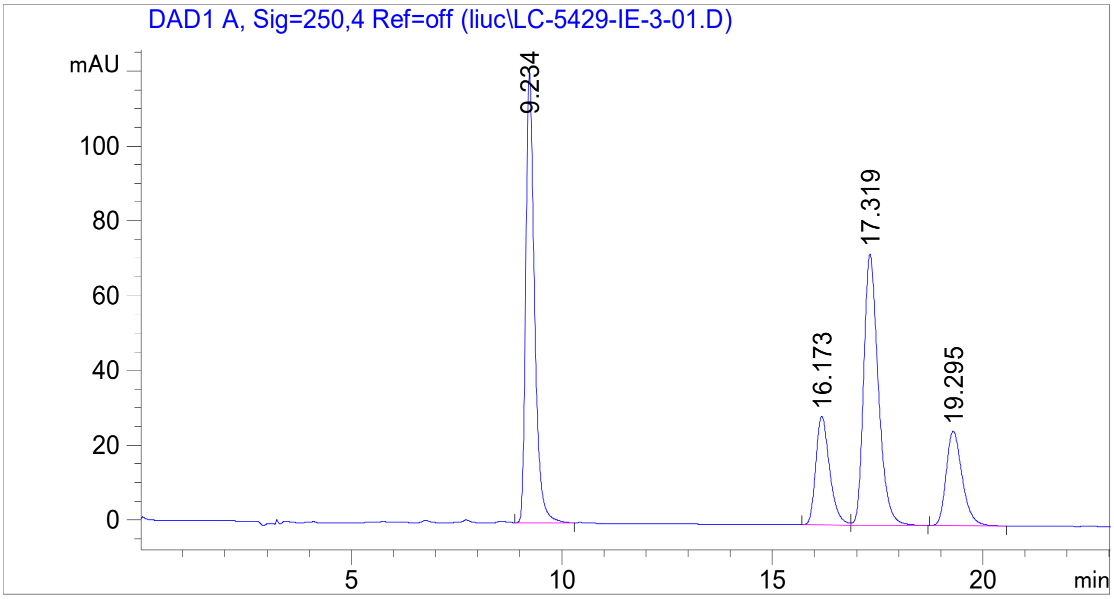

#	Time	Area	Height	Width	Area%	Symmetry
1	9.234	1740.2	120.5	0.2185	36.142	0.661
2	16.173	663.2	29	0.3484	13.774	0.704
3	17.319	1744.1	72.4	0.3691	36.224	0.704
4	19.295	667.3	25.2	0.4065	13.860	0.731

3eb-racmic

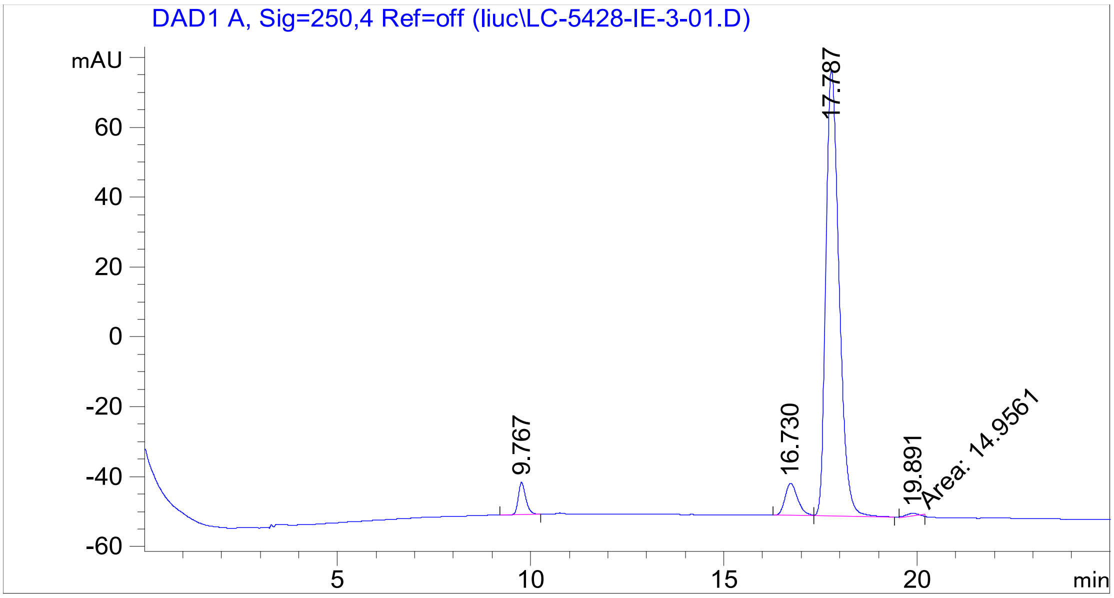

#	Time	Area	Height	Width	Area%	Symmetry
1	9.767	123.5	9.3	0.2037	3.593	0.737
2	16.73	207.1	9.2	0.343	6.025	0.748
3	17.787	3026.3	127.8	0.3643	88.035	0.684
4	19.891	80.7	2	0.6603	2.347	0.589

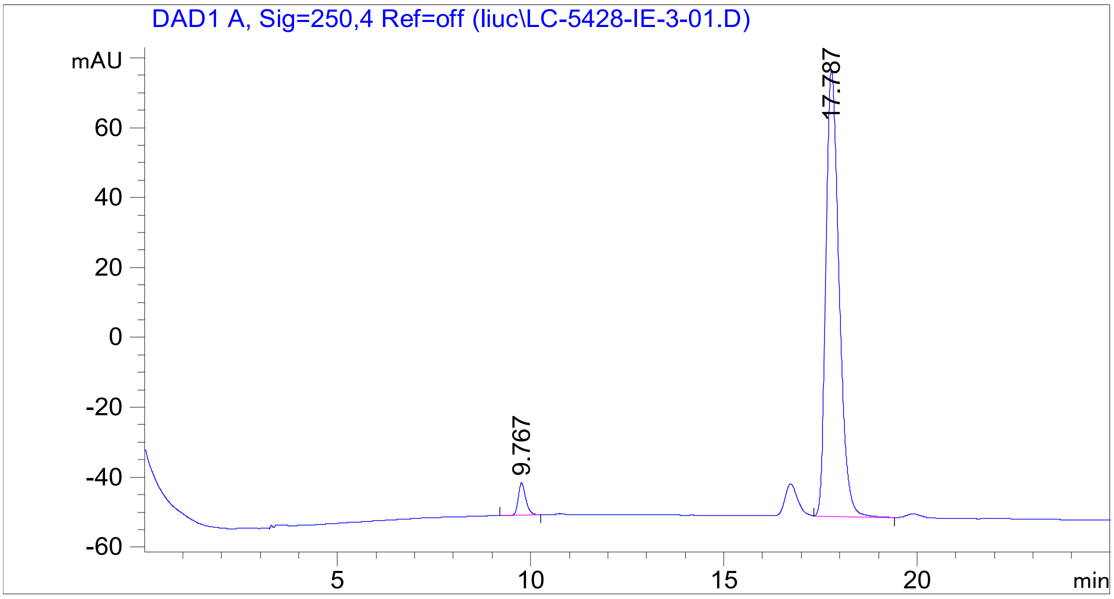

#	Time	Area	Height	Width	Area%	Symmetry
1	9.767	123.5	9.3	0.2037	3.922	0.737
2	17.787	3026.3	127.8	0.3643	96.078	0.684

3ec-racmic

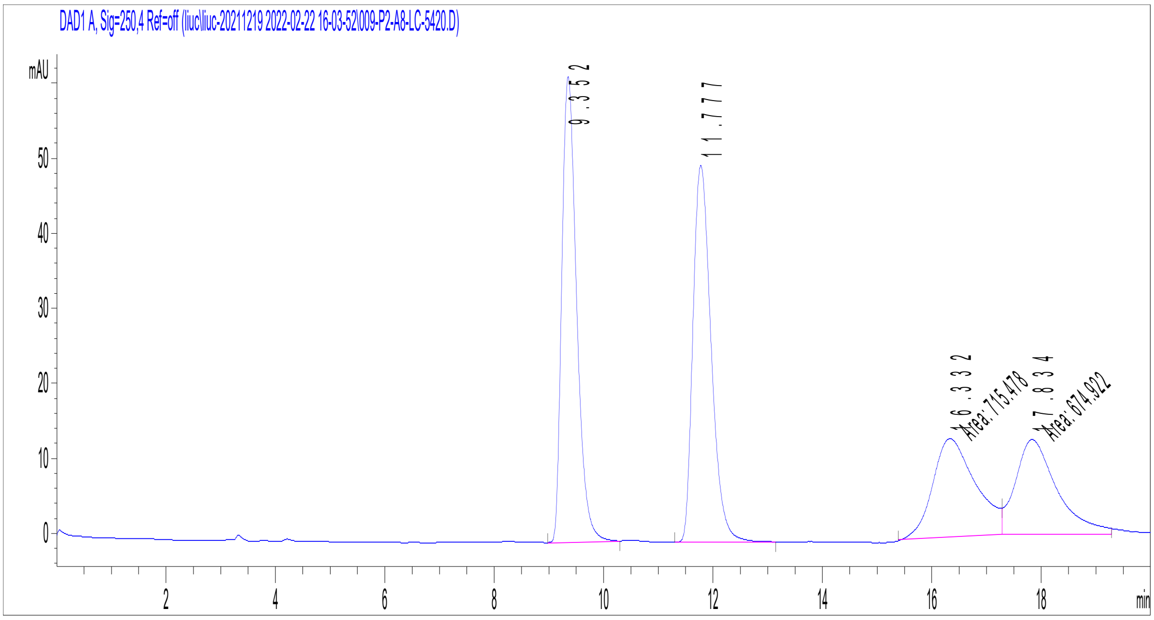

#	Time	Area	Height	Width	Area%	Symmetry
1	9.352	1164.2	62	0.2931	31.366	0.688
2	11.777	1157.2	50.2	0.3589	31.176	0.704
3	16.332	715.5	13.1	0.9086	19.276	0.657
4	17.834	674.9	12.7	0.8869	18.183	0.642

3ec-chiral

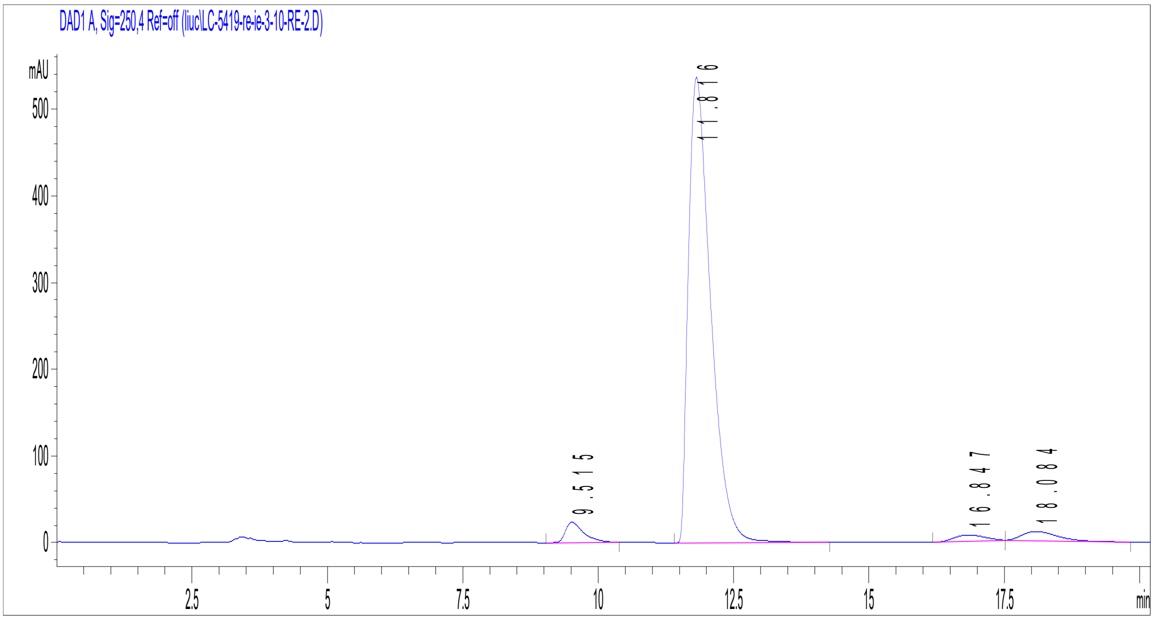

#	Time	Area	Height	Width	Area%	Symmetry
1	9. 515	548. 6	23. 5	0. 3525	3. 296	0. 508
2	11. 816	15334. 4	537. 3	0. 443	92. 117	0. 525
3	16. 847	285. 1	7. 3	0. 6106	1. 713	0. 958
4	18. 084	478. 6	10. 5	0. 6825	2. 875	0. 594

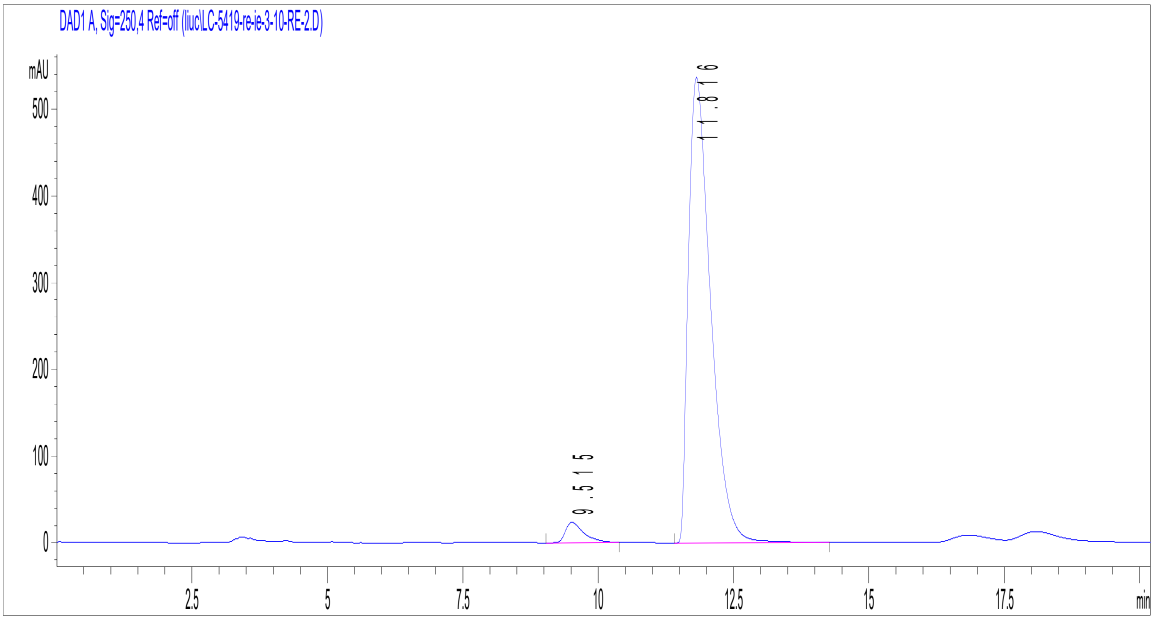

#	Time	Area	Height	Width	Area%	Symmetry
1	9. 515	548. 6	23. 5	0. 3525	3. 454	0. 508
2	11. 816	15334. 4	537. 3	0. 443	96. 546	0. 525

3ed-racmic

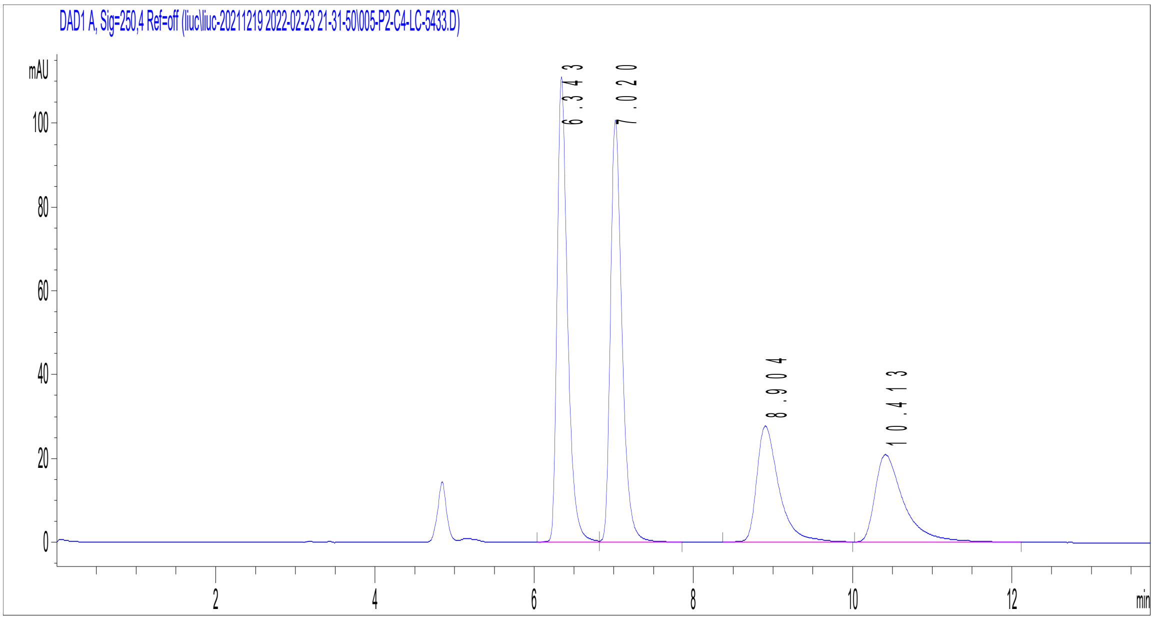

#	Time	Area	Height	Width	Area%	Symmetry
1	6.343	1013.1	111.1	0.1384	32.852	0.657
2	7.02	1014.3	100.9	0.1533	32.890	0.678
3	8.904	536.7	27.8	0.2894	17.402	0.584
4	10.413	519.8	20.8	0.3689	16.856	0.52

3ed-chiral

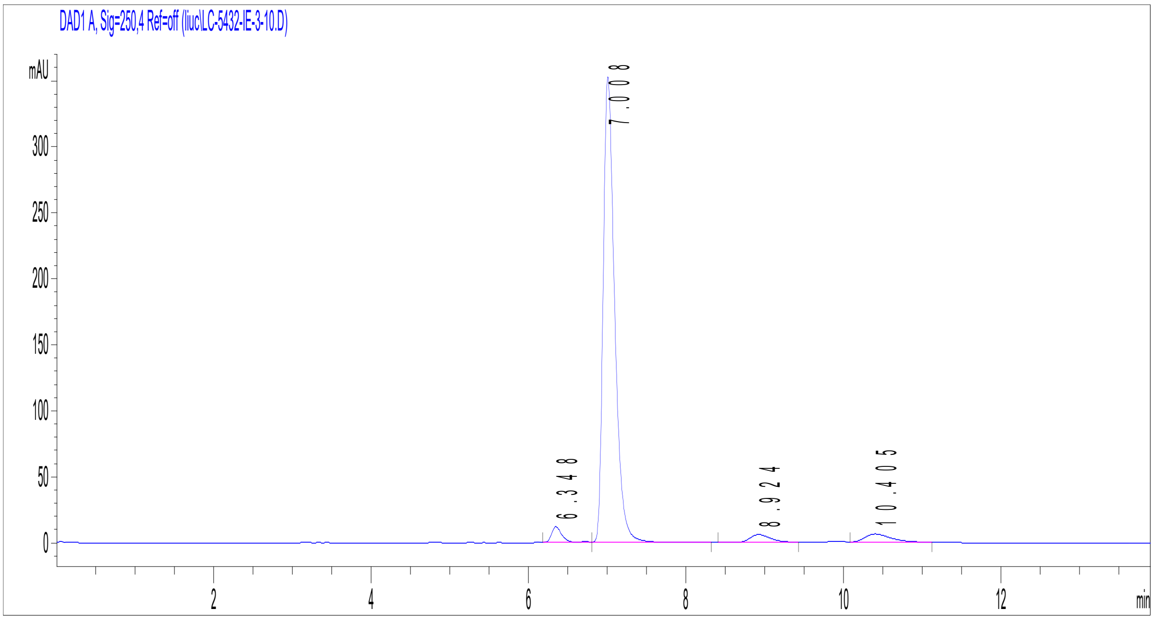

#	Time	Area	Height	Width	Area%	Symmetry
1	6.348	110.4	11.9	0.1404	2.815	0.625
2	7.008	3566.5	352.9	0.1539	90.979	0.632
3	8.924	105.9	5.9	0.2735	2.702	0.751
4	10.405	137.3	6.1	0.3396	3.503	0.604

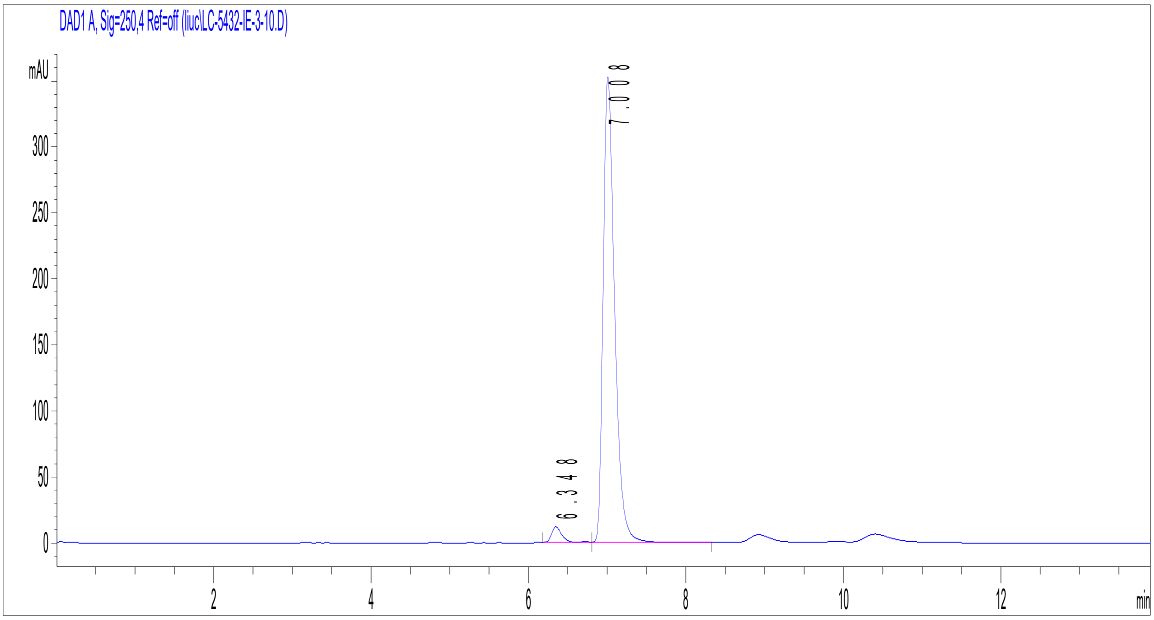

#	Time	Area	Height	Width	Area%	Symmetry
1	6.348	110.4	11.9	0.1404	3.002	0.625
2	7.008	3566.5	352.9	0.1539	96.998	0.632

3ee-racmic

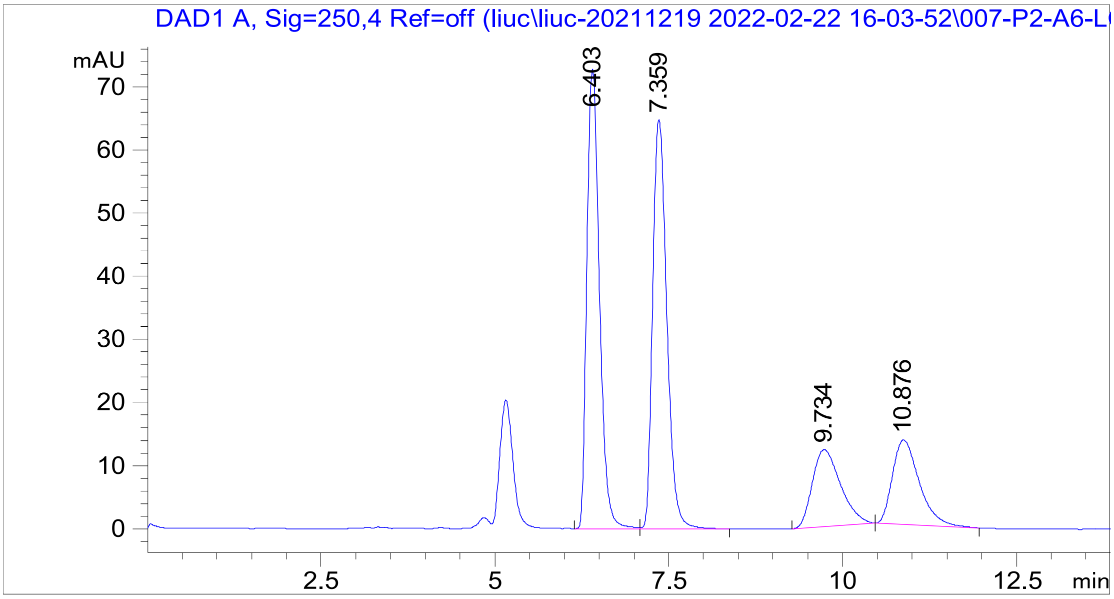

#	Time	Area	Height	Width	Area%	Symmetry
1	6.403	913.5	72.7	0.1965	36.027	0.726
2	7.359	916.8	64.8	0.2231	36.159	0.733
3	9.734	345.3	12.2	0.4357	13.620	0.684
4	10.876	359.9	13.4	0.4096	14.194	0.658

3ee-chiral

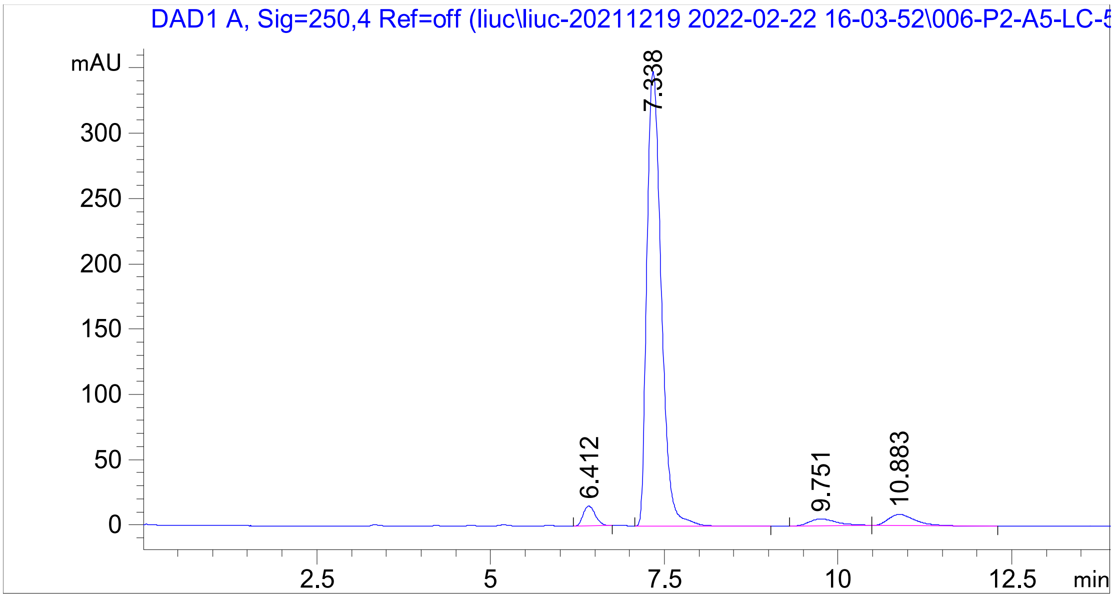

#	Time	Area	Height	Width	Area%	Symmetry
1	6. 412	186. 7	15. 3	0. 1926	3. 353	0. 781
2	7. 338	5001. 2	348. 5	0. 2254	89. 835	0. 686
3	9. 751	149. 7	5. 2	0. 4409	2. 689	0. 658
4	10. 883	229. 5	8. 5	0. 4086	4. 123	0. 638

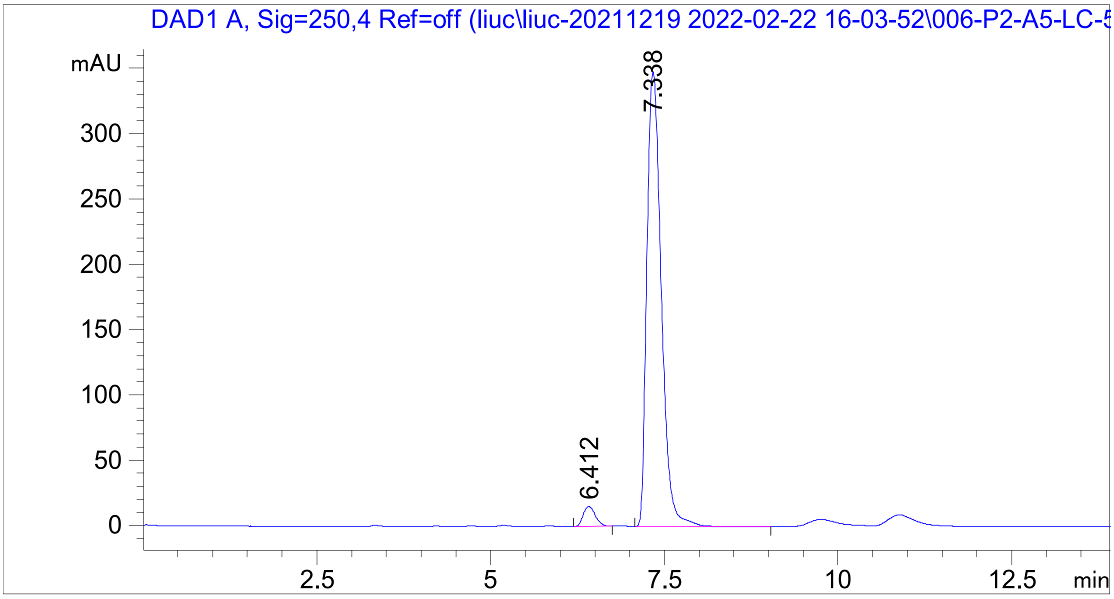

#	Time	Area	Height	Width	Area%	Symmetry
1	6. 412	186. 7	15. 3	0. 1926	3. 598	0. 781
2	7. 338	5001. 2	348. 5	0. 2254	96. 402	0. 686

3ef-racmic

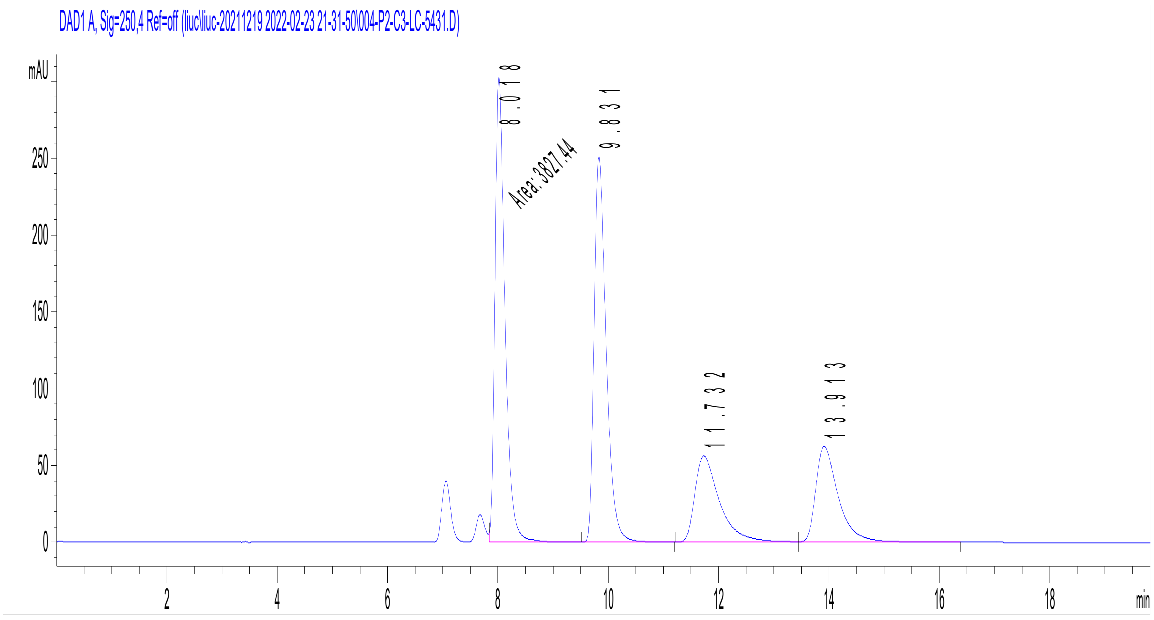

#	Time	Area	Height	Width	Area%	Symmetry
1	8.018	3827.4	303.1	0.2105	34.414	0.621
2	9.831	3810.9	251.3	0.231	34.265	0.615
3	11.732	1732.7	56	0.4629	15.580	0.52
4	13.913	1750.8	62.1	0.4227	15.742	0.546

3ef-chiral

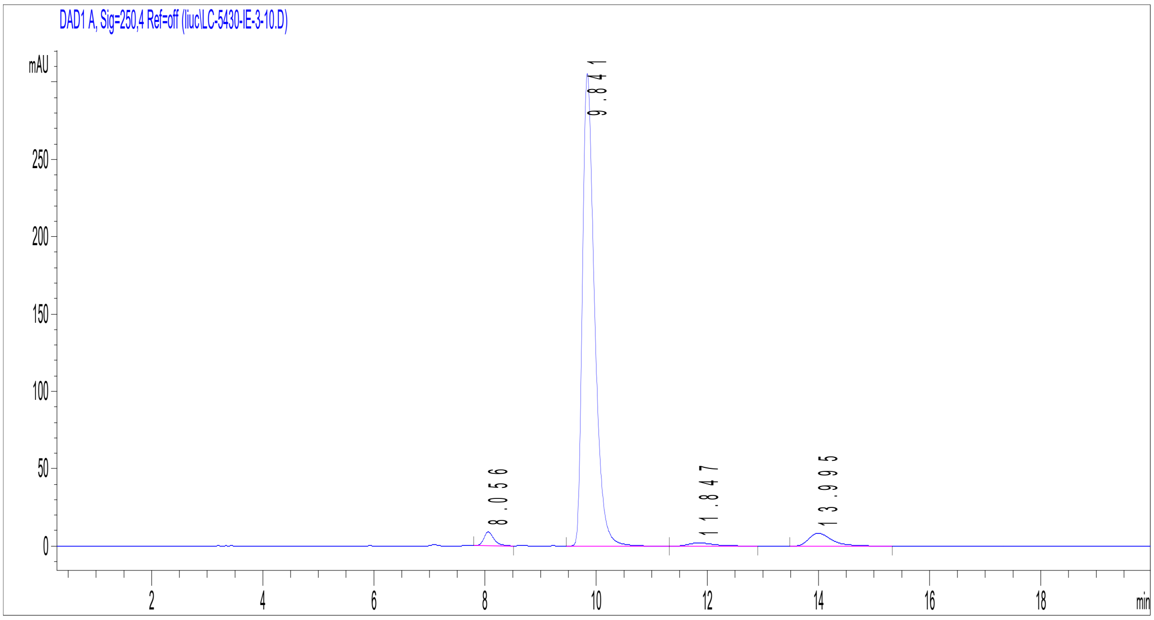

#	Time	Area	Height	Width	Area%	Symmetry
1	8.056	113.9	9	0.1922	2.256	0.692
2	9.841	4626.2	305.6	0.2326	91.625	0.613
3	11.847	64.1	2.1	0.4444	1.269	0.63
4	13.995	244.9	8.4	0.4468	4.850	0.617

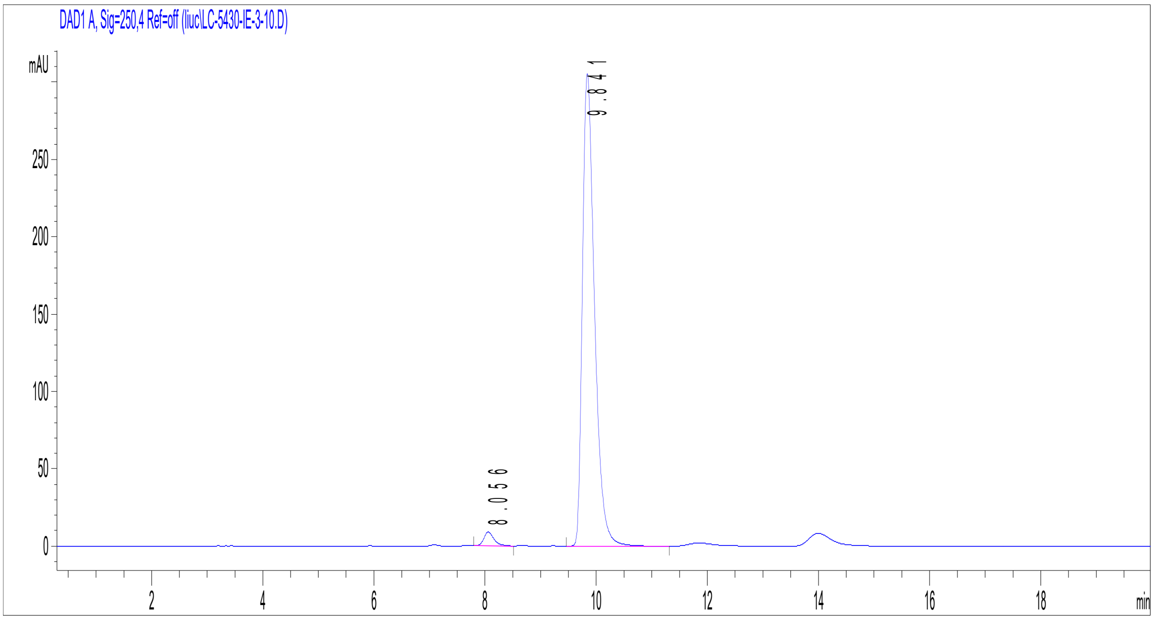

#	Time	Area	Height	Width	Area%	Symmetry
1	8.056	113.9	9	0.1922	2.403	0.692
2	9.841	4626.2	305.6	0.2326	97.597	0.613

3eg-racmic

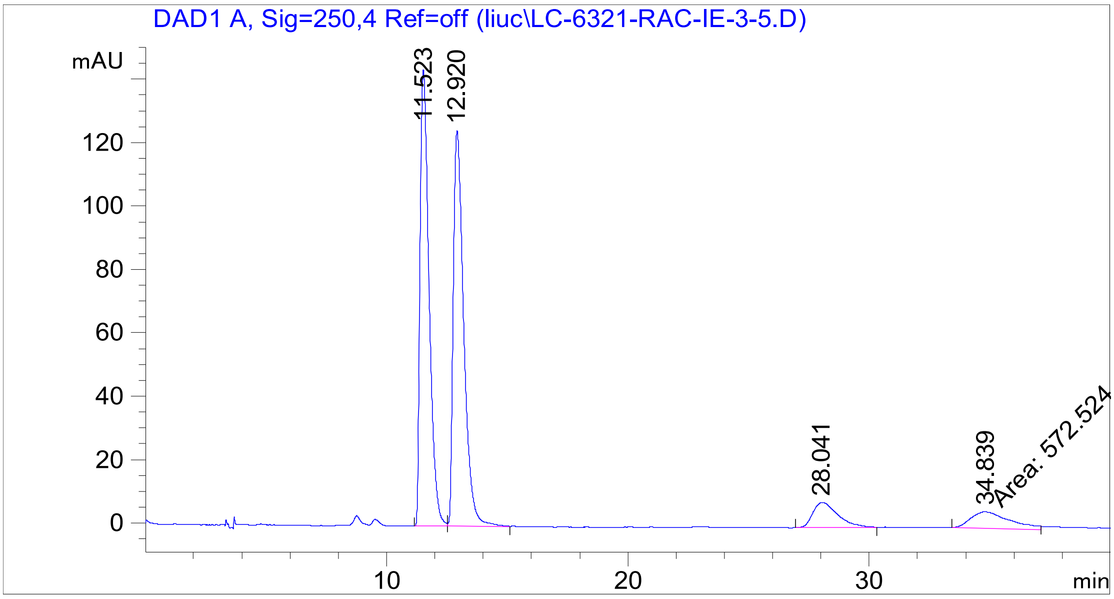

#	Time	Area	Height	Width	Area%	Symmetry
1	11.523	3742.1	143.8	0.4036	43.291	0.538
2	12.92	3764.6	124.7	0.4641	43.552	0.522
3	28.041	564.8	7.9	0.8565	6.534	0.565
4	34.839	572.5	5.1	1.8542	6.623	0.504

3eg-chiral

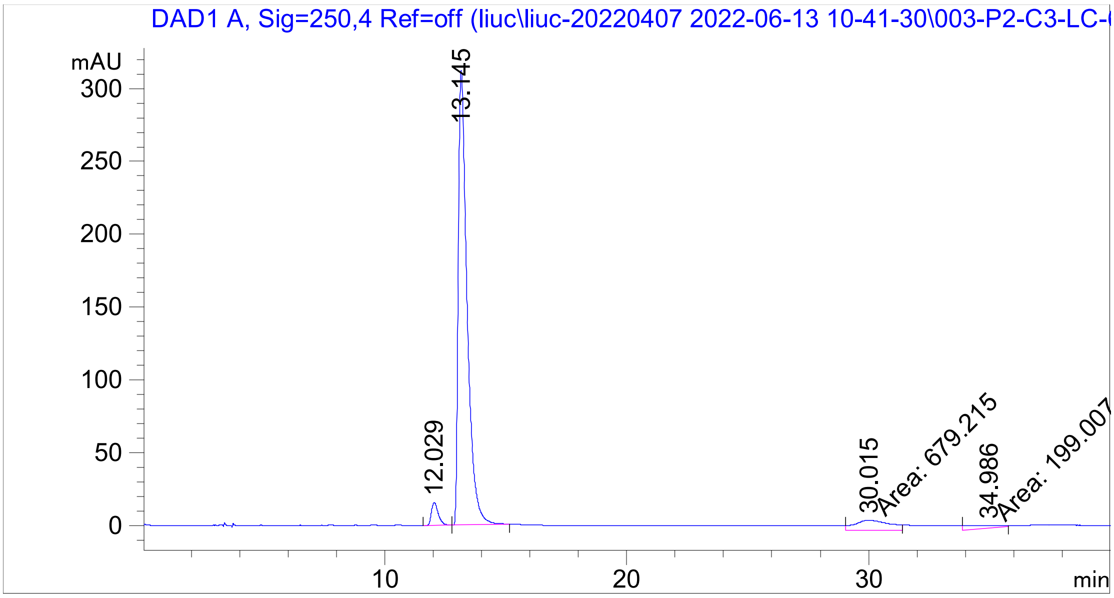

#	Time	Area	Height	Width	Area%	Symmetry
1	12.029	317.3	15.9	0.3047	3.471	0.646
2	13.145	7947.8	312.2	0.381	86.924	0.445
3	30.015	679.2	6.7	1.6892	7.429	0.659
4	34.986	199	1.6	1.4528	2.177	0

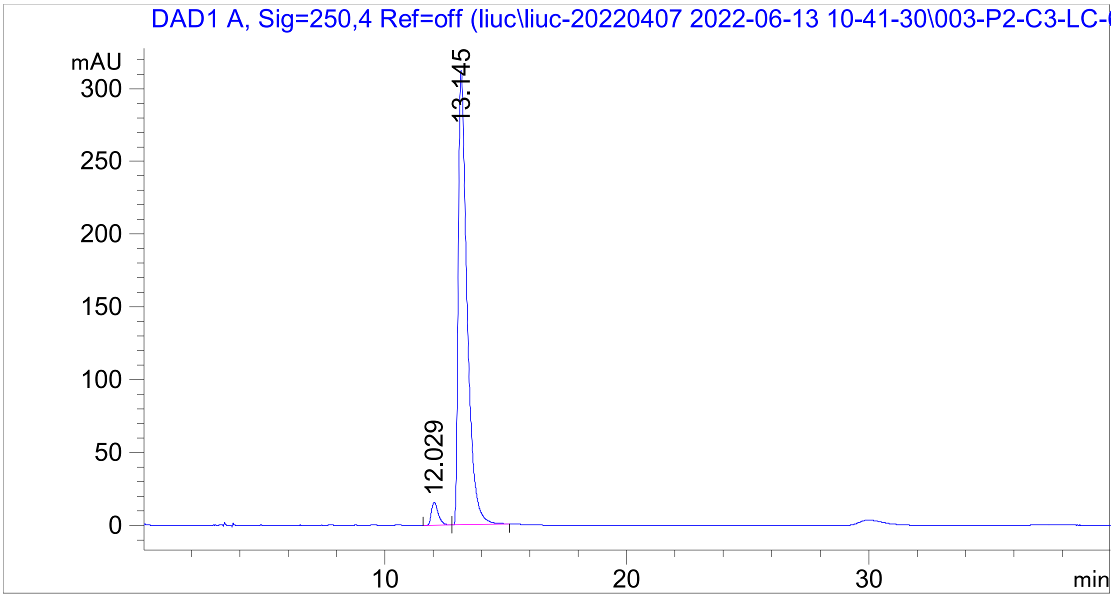

#	Time	Area	Height	Width	Area%	Symmetry
1	12.029	317.3	15.9	0.3047	3.839	0.646
2	13.145	7947.8	312.2	0.381	96.161	0.445

3eh-racmic

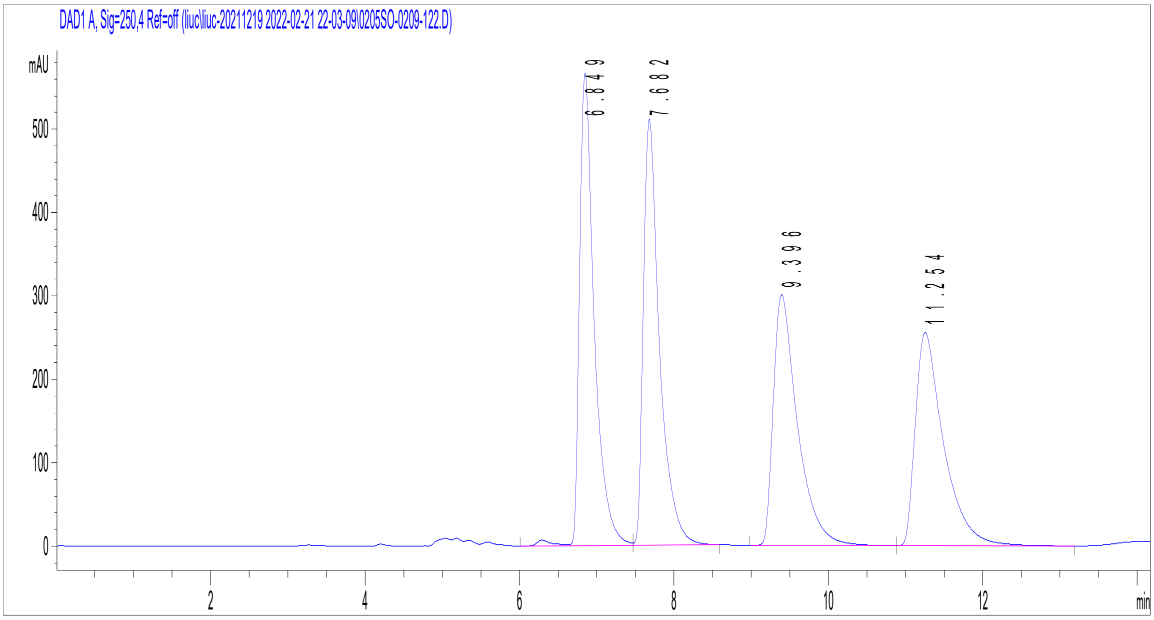

#	Time	Area	Height	Width	Area%	Symmetry
1	6.849	7561.8	566.5	0.1978	26.926	0.567
2	7.682	7454.9	511.2	0.2182	26.546	0.53
3	9.396	6544.9	301	0.3197	23.305	0.483
4	11.254	6521.6	255.9	0.3733	23.222	0.48

3eh-chiral

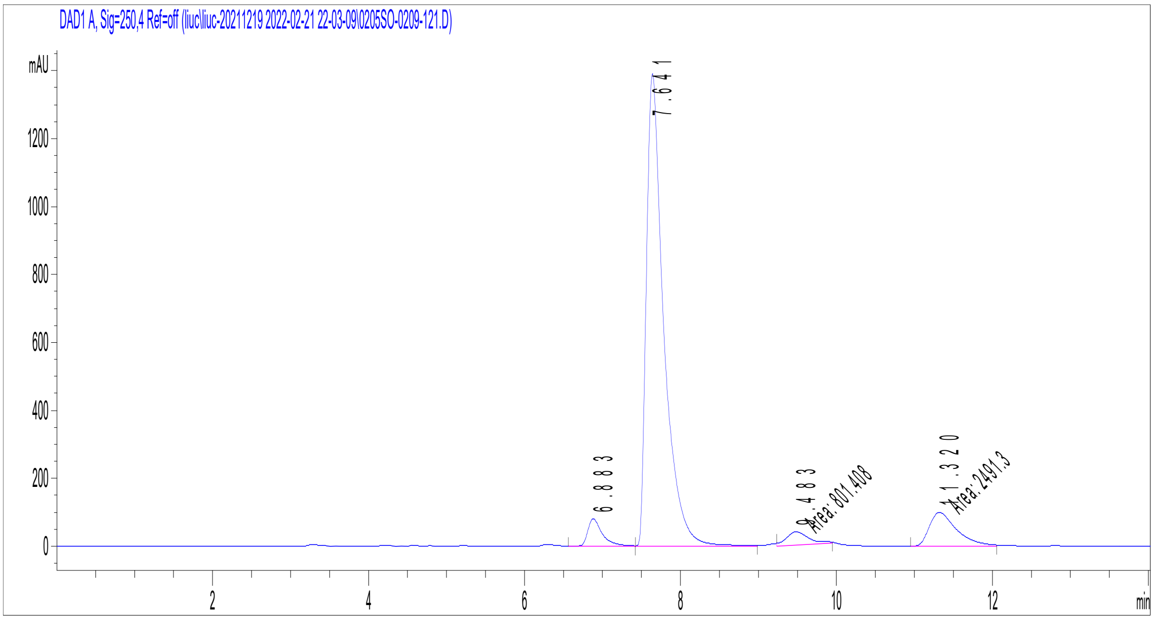

#	Time	Area	Height	Width	Area%	Symmetry
1	6.883	1009.8	79.9	0.1915	3.922	0.573
2	7.641	21447.1	1390.3	0.23	83.291	0.487
3	9.483	801.4	39.8	0.336	3.112	0.801
4	11.32	2491.3	100.6	0.4128	9.675	0.56

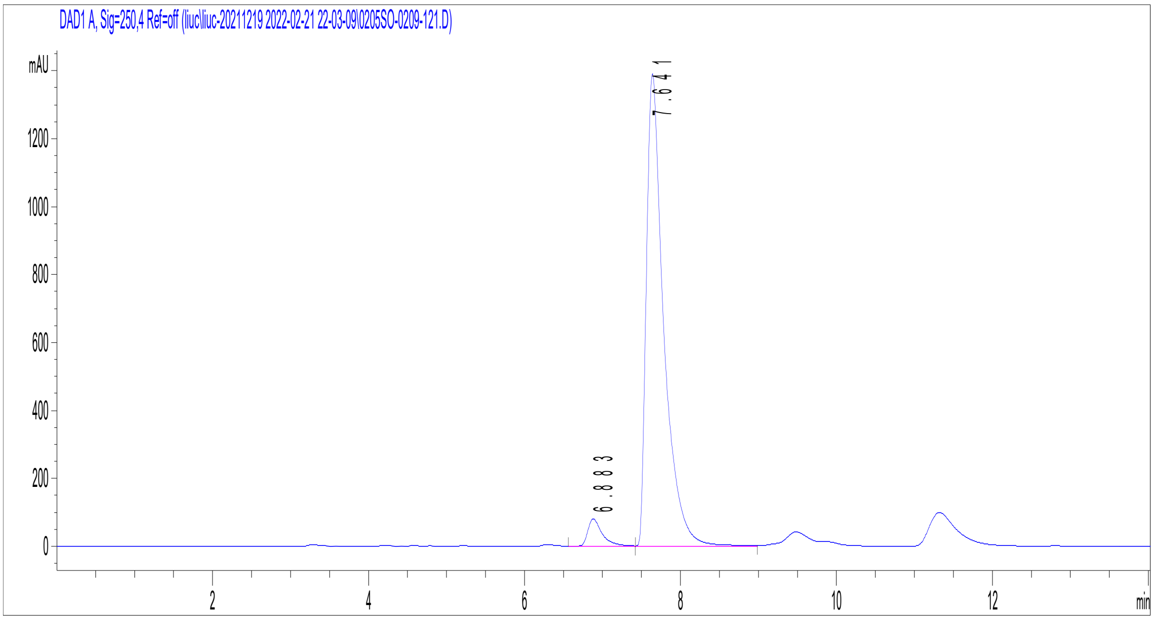

#	Time	Area	Height	Width	Area%	Symmetry
1	6.883	1009.8	79.9	0.1915	4.497	0.573
2	7.641	21447.1	1390.3	0.23	95.503	0.487

3ei-racmic

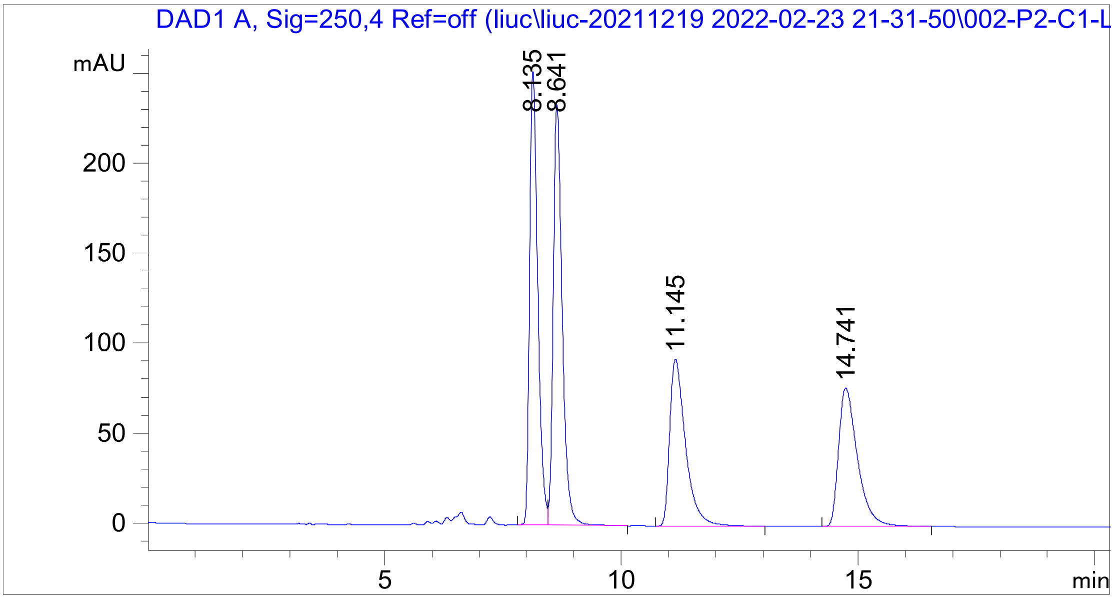

#	Time	Area	Height	Width	Area%	Symmetry
1	8. 135	3002. 7	252. 1	0. 183	28. 882	0. 65
2	8. 641	3095. 9	233. 9	0. 2024	29. 778	0. 644
3	11. 145	2156. 3	92. 8	0. 347	20. 740	0. 522
4	14. 741	2141. 7	77. 1	0. 4223	20. 600	0. 565

3ei-chiral

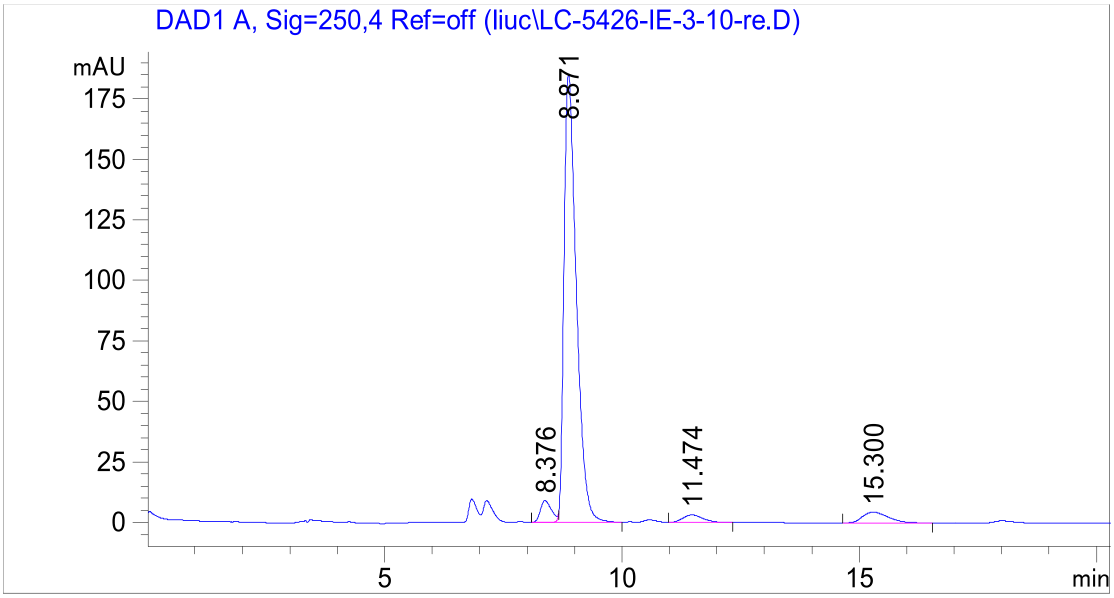

#	Time	Area	Height	Width	Area%	Symmetry
1	8.376	143.2	9	0.2439	3.806	0.672
2	8.871	3352.6	185.2	0.2752	89.095	0.673
3	11.474	91.1	3.1	0.4161	2.422	0.801
4	15.3	176	4.5	0.5447	4.676	0.657

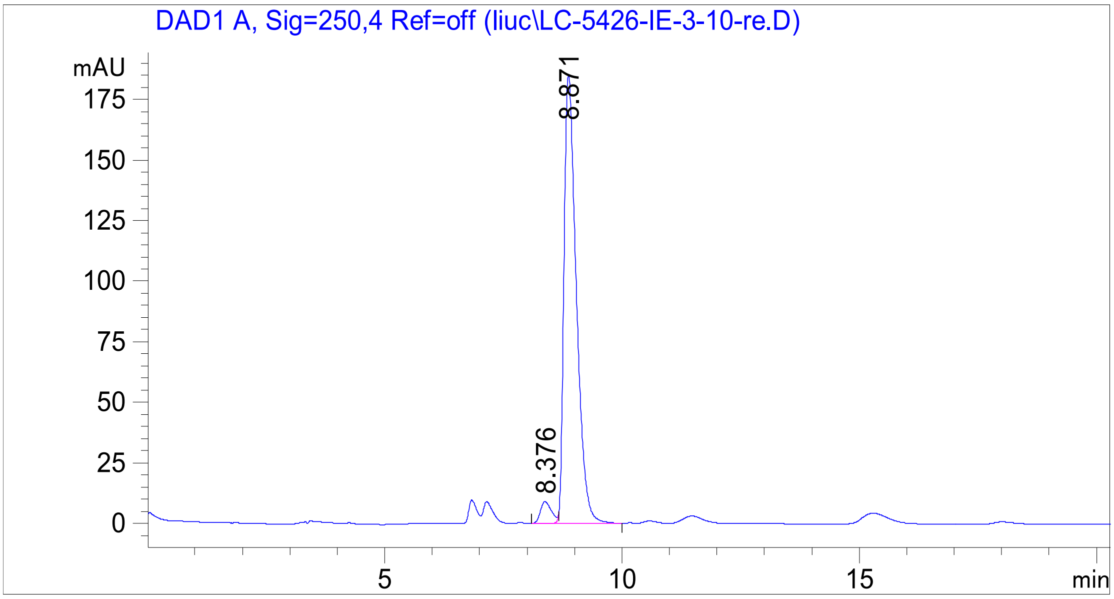

#	Time	Area	Height	Width	Area%	Symmetry
1	8.376	143.2	9	0.2439	4.097	0.672
2	8.871	3352.6	185.2	0.2752	95.903	0.673

3ej-racmic

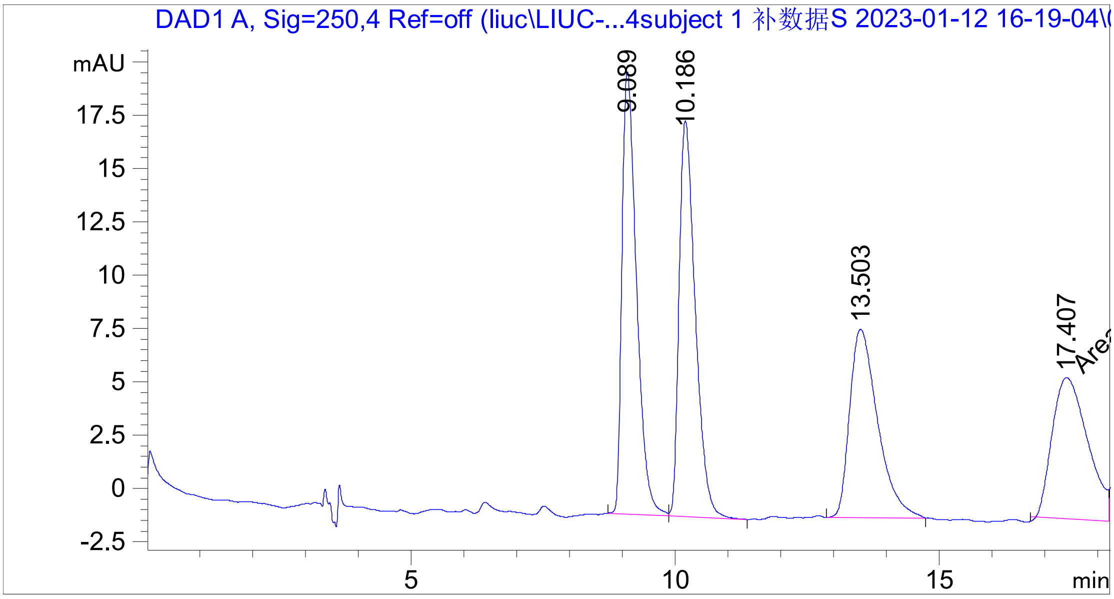

#	Time	Area	Height	Width	Area%	Symmetry
1	9.089	397.5	20.7	0.2916	27.613	0.548
2	10.186	396.2	18.6	0.325	27.523	0.579
3	13.503	325.6	8.8	0.548	22.619	0.591
4	17.407	320.2	6.8	0.7905	22.244	0.677

3ej-chiral

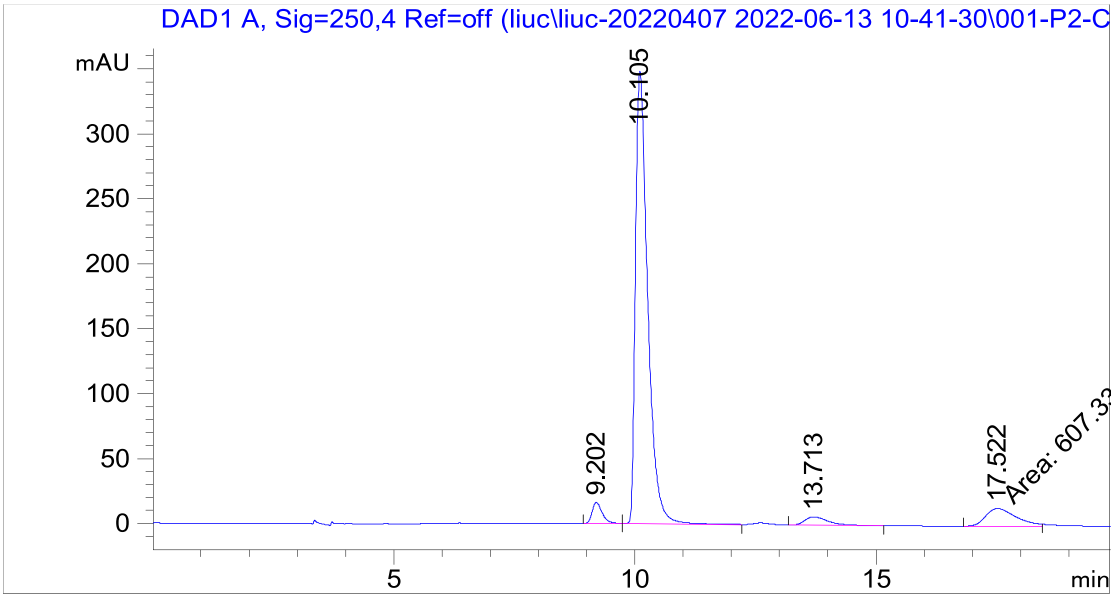

#	Time	Area	Height	Width	Area%	Symmetry
1	9.202	228.3	16	0.2203	3.182	0.683
2	10.105	6123.8	348.4	0.2671	85.358	0.512
3	13.713	214.8	6.5	0.4945	2.995	0.592
4	17.522	607.3	13.7	0.7373	8.466	0.604

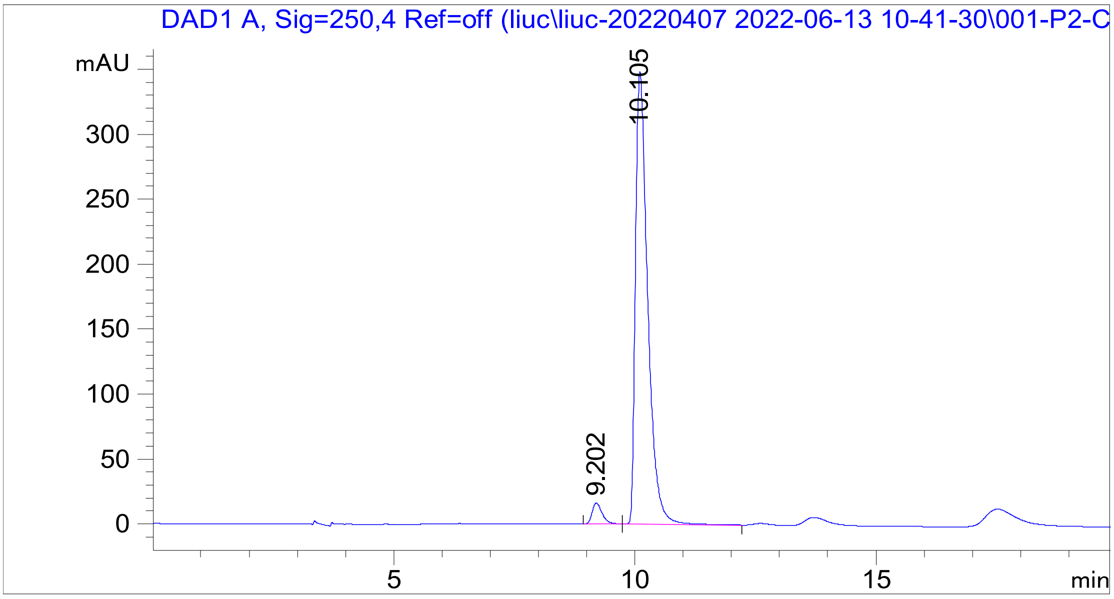

#	Time	Area	Height	Width	Area%	Symmetry
1	9.202	228.3	16	0.2203	3.594	0.683
2	10.105	6123.8	348.4	0.2671	96.406	0.512

3ek-racmic

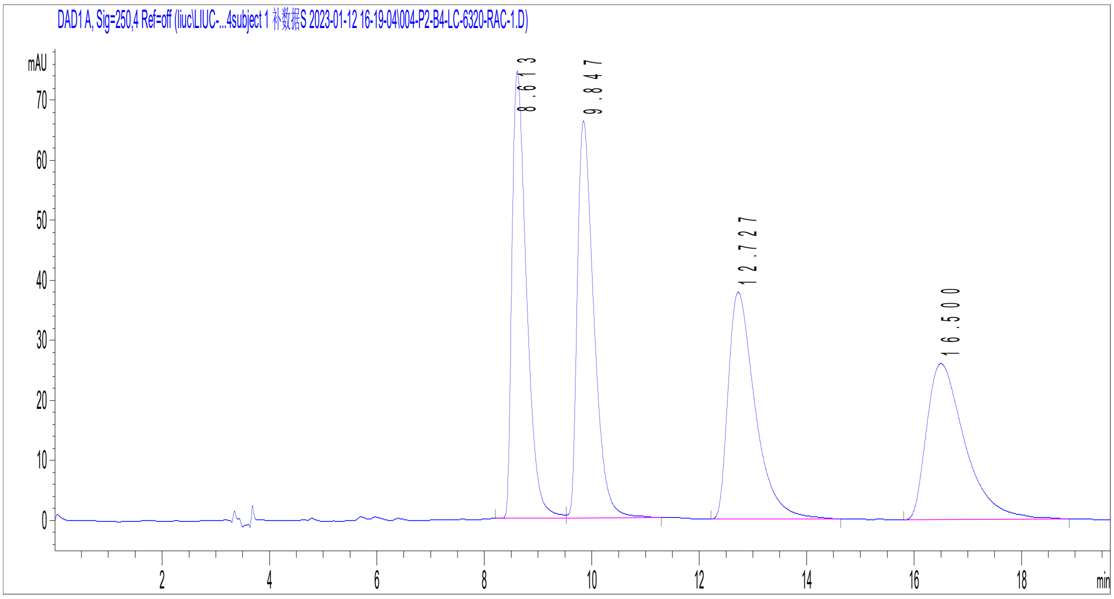

#	Time	Area	Height	Width	Area%	Symmetry
1	8.613	1404.2	74.5	0.2859	25.901	0.52
2	9.847	1412.9	66.2	0.327	26.061	0.548
3	12.727	1317.1	37.8	0.524	24.295	0.54
4	16.5	1287.2	26	0.7494	23.743	0.553

3ek-chiral

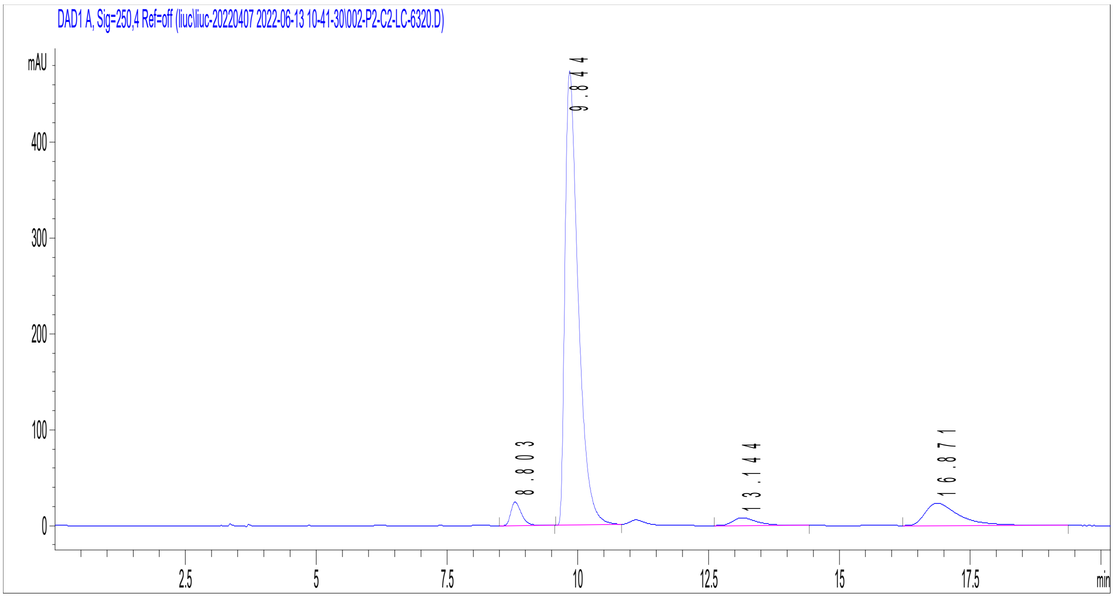

#	Time	Area	Height	Width	Area%	Symmetry
1	8.803	346.9	24.6	0.2163	3.470	0.642
2	9.844	8330.1	473.8	0.2651	83.311	0.492
3	13.144	262.8	8.1	0.4828	2.628	0.584
4	16.871	1059	23.3	0.6712	10.591	0.498

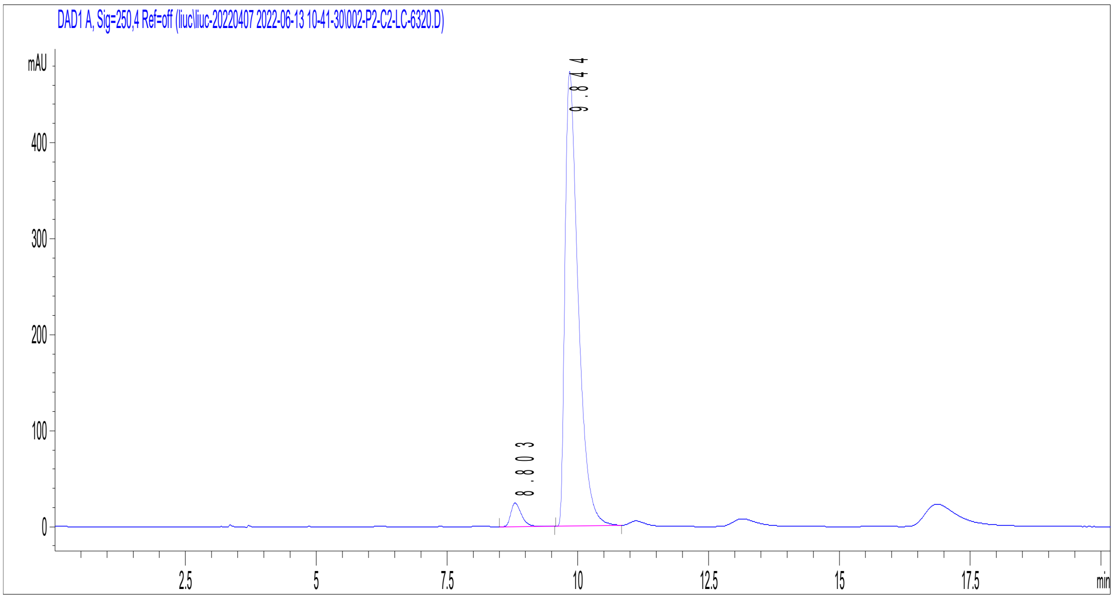

#	Time	Area	Height	Width	Area%	Symmetry
1	8.803	346.9	24.6	0.2163	3.998	0.642
2	9.844	8330.1	473.8	0.2651	96.002	0.492
